# Pyridine-enabled copper-promoted cross dehydrogenative coupling of C(sp^2^)–H and unactivated C(sp^3^)–H bonds†
†Electronic supplementary information (ESI) available: Experimental details including characterization data, copies of ^1^H, ^13^C NMR and NOESY spectra. See DOI: 10.1039/c5sc02143j


**DOI:** 10.1039/c5sc02143j

**Published:** 2015-07-20

**Authors:** Xuesong Wu, Yan Zhao, Haibo Ge

**Affiliations:** a Department of Chemistry and Chemical Biology , Indiana University-Purdue University Indianapolis , Indianapolis , Indiana 46202 , USA . Email: geh@iupui.edu

## Abstract

The pyridine-enabled cross dehydrogenative coupling of sp^2^ C–H bonds of polyfluoroarenes and unactivated sp^3^ C–H bonds of amides was achieved *via* a copper-promoted process with good functional group compatibility. This reaction showed great site-selectivity by favoring the sp^2^ C–H bonds *ortho* to two fluoro atoms of arenes and the sp^3^ C–H bonds of α-methyl groups over those of the α-methylene, β- or γ-methyl groups of the aliphatic amides. Mechanistic studies revealed that sp^3^ C–H bond cleavage is an irreversible but not the rate-determining step, and the sp^2^ C–H functionalization of arenes appears precedent to the sp^3^ C–H functionalization of amides in this process.

## Abstract 2

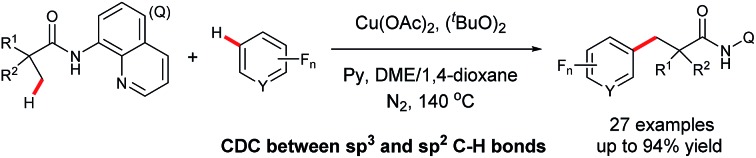
Pyridine-enabled cross dehydrogenative coupling of sp^2^ C–H bonds of polyfluoroarenes and unactivated sp^3^ C–H bonds of amides was achieved.

## Introduction

**Scheme 1:**
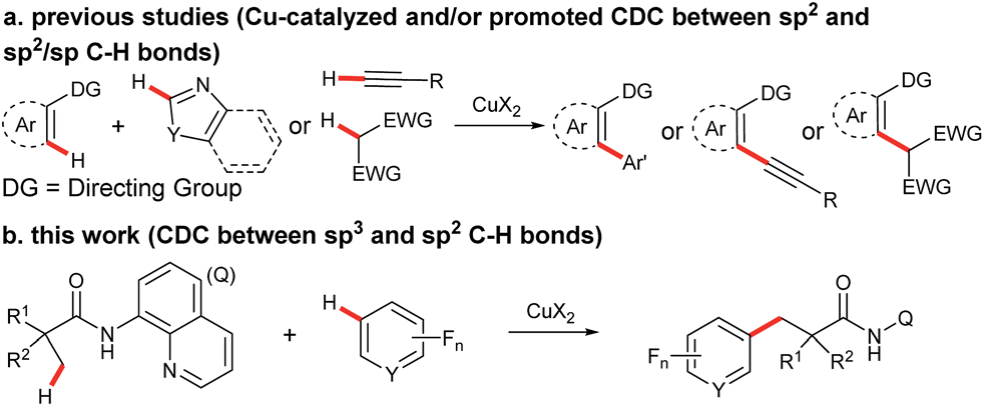
Copper-promoted cross dehydrogenative coupling (CDC) reactions.

Transition metal-promoted direct functionalization of unactivated C–H bonds is a highly valuable approach for the selective construction of C–C bonds, and considerable efforts have been devoted into this research area over the past couple of decades.^1^ Within this reaction category, ligand-assisted cross dehydrogenative coupling (CDC) is of current interest, and significant progress has been achieved in recent years.^2^ Compared with the conventional cross coupling reactions, this method enables the direct manipulation of aromatic and aliphatic C–H bonds by obviating the pre-installation of the functional groups. Moreover, the ligand acts as a directing group to ensure the high site-selectivity. In the process, a noble metal species such as palladium, rhodium, or ruthenium is often employed as a catalyst. From an economical point of view, avoidance of the use of the precious catalyst in the process would be highly desirable. Towards this effort, Miura and co-workers reported the first copper-promoted cross dehydrogenative coupling of 2-phenylpyridines and benzoxazoles in 2011 (Scheme 1a).^3^ It was then found that azine-*N*-oxides, benzamides, indoles, naphthylamines, and 2-pyridones were also effective substrates.^4^ Despite being a highly efficient method for the construction of C–C bonds, this process does not allow for the site-selective direct functionalization of unactivated sp^3^ bonds coupling with an arene bearing a directing group, thus restricting the product diversity.^5^,^6^ Inspired by the bidentate directing group-assisted unactivated sp^3^ C–H bond activation process developed by Daugulis' group,^7^ we envisaged that attachment of a bidentate directing group to an aliphatic acid may potentially overcome this drawback.^8^ With this design, we have examined and report here the copper-promoted cross dehydrogenative coupling of aliphatic amides^9^ and polyfluoroarenes^10^ (Scheme 1b), which provides an efficient access to alkyl-substituted perfluoroarenes, an important structural motif in pharmaceuticals and agrochemicals.^11^ It is worth mentioning that this is the first example of ligand-directed copper-promoted cross dehydrogenative coupling reaction by employing polyfluoroarenes as the coupling partners.

## Results and discussion

Our investigation commenced with cross dehydrogenative coupling of 2-ethyl-2-methyl-*N*-(quinolin-8-yl)pentanamide (**1a**) and pentafluorobenzene (**2a**) in 1,4-dioxane with stoichiometric amounts of Cu(OAc)_2_ under atmospheric oxygen (Table 1). After an extensive screening of the bases, pyridine has proven to be optimal, affording the desired product **3a** in 22% yield, while all inorganic bases failed in the reaction (entries 1–7). Considering that pyridine could also act as a ligand in the process, and thus promote the reaction, a screening of nitrogen-containing potential bidentate ligands was further carried out. As shown in Table 1, several ligands such as TMEDA, 2,2′-bipyridine, and 1,10-phenanthroline could promote the process, but none of these molecules is as effective as pyridine (entries 8–10). Next, different copper sources were examined, and it was found that CuOAc was the only other species, but with less efficiency (entry 13). Then, the effects of an oxidant towards the reaction were examined, and it turned out that di-*tert*-butyl peroxide was optimal, providing **3a** in 44% yield (entry 17). It was also noted that a higher yield could be obtained under atmospheric nitrogen (entry 18). Following the above investigation, we carried out an extensive screening of the solvents, and the reaction was significantly improved with the solvent mixture of DME and 1,4-dioxane (entry 22). Furthermore, an excellent yield was observed with increased amounts of pyridine (entry 24). It was also noted that the reaction yield was dramatically decreased with reduced amounts of the copper species (entry 25), presumably due to the competitive coordination of pyridine or *tert*-butanolate released from di-*tert*-butyl peroxide to copper. Moreover, it is clear that pyridine is required for this process since no apparent product formation occurred in the absence of pyridine (entry 26). As expected, a high site-selectivity was observed with a preference for the C–H bond of the α-methyl over that of the α-methylene, β- or γ-methyl group,^9^ which is believed to arise from the steric effect and preference for the formation of a five-membered ring intermediate over the six- or seven-membered ring intermediate in the cyclometalation step.

**Table 1:** Optimization of reaction conditions^*a*^

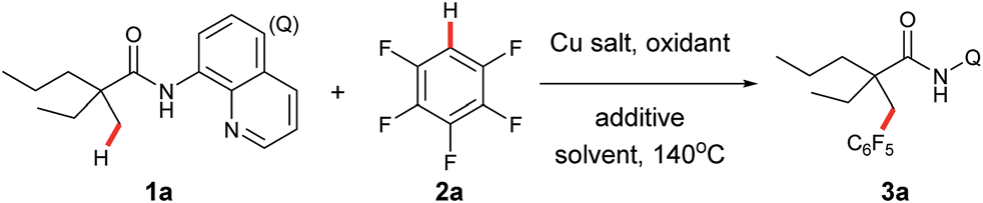					
Entry	Cu salt	Oxidant	Additive (eq.)	Solvent	Yield (%)^*b*^
1	Cu(OAc)_2_		K_2_CO_3_ (2)	1,4-Dioxane	0
2	Cu(OAc)_2_		K_2_HPO_4_ (2)	1,4-Dioxane	0
3	Cu(OAc)_2_		PhCO_2_Na (2)	1,4-Dioxane	0
4	Cu(OAc)_2_		Et_3_N (2)	1,4-Dioxane	<5
5	Cu(OAc)_2_		Py (2)	1,4-Dioxane	22
6	Cu(OAc)_2_		2,6-Lutidine (2)	1,4-Dioxane	14
7	Cu(OAc)_2_		DMAP (2)	1,4-Dioxane	17
8	Cu(OAc)_2_		TMEDA (1)	1,4-Dioxane	6
9	Cu(OAc)_2_		2,2′-Dipyridyl (1)	1,4-Dioxane	16
10	Cu(OAc)_2_		1,10-Phen (1)	1,4-Dioxane	<5
11	CuCl_2_		Py (2)	1,4-Dioxane	0
12	CuBr_2_		Py (2)	1,4-Dioxane	0
13	CuOAc		Py (2)	1,4-Dioxane	<5
14	CuBr		Py (2)	1,4-Dioxane	0
15	Cu(OAc)_2_	Ag_2_O	Py (2)	1,4-Dioxane	25
16	Cu(OAc)_2_	TBHP	Py (2)	1,4-Dioxane	10
17	Cu(OAc)_2_	(^*t*^BuO)_2_	Py (2)	1,4-Dioxane	44
18^*c*^	Cu(OAc)_2_	(^*t*^BuO)_2_	Py (2)	1,4-Dioxane	59
19^*c*^	Cu(OAc)_2_	(^*t*^BuO)_2_	Py (2)	DME	46
20^*c*^	Cu(OAc)_2_	(^*t*^BuO)_2_	Py (2)	THF	40
21^*c*^	Cu(OAc)_2_	(^*t*^BuO)_2_	Py (2)	Toluene	15
22^*c*^	Cu(OAc)_2_	(^*t*^BuO)_2_	Py (2)	DME–1,4-dioxane (7 : 3)	84
23^*c*^	Cu(OAc)_2_	(^*t*^BuO)_2_	Py (1)	DME–1,4-dioxane (7 : 3)	50
24^*c*^	Cu(OAc)_2_	(^*t*^BuO)_2_	Py (3)	DME–1,4-dioxane (7 : 3)	96 (92)
25^*c*^ ^*d*^	Cu(OAc)_2_	(^*t*^BuO)_2_	Py (3)	DME–1,4-dioxane (7 : 3)	63
26^*c*^	Cu(OAc)_2_	(^*t*^BuO)_2_	—	DME–1,4-dioxane (7 : 3)	0

^*a*^Reaction conditions: **1a** (0.3 mmol), **2a** (0.6 mmol), Cu salt (0.3 mmol), oxidant (0.75 mmol), additive, 1.0 mL of solvent, 140 °C, 16 h.

^*b*^Yields and conversions are based on **1a**, determined by ^1^H-NMR using dibromomethane as the internal standard. Isolated yield is in parenthesis.

^*c*^Under N_2_ atmosphere.

^*d*^Cu(OAc)_2_ (0.15 mmol). Q = 8-quinolinyl.

Under the optimized conditions, the scope with respect to fluoroarenes was examined. As shown in Table 2, tetrafluorobenzenes bearing a methoxyl, bromo, cyano, or trifluoromethyl group were compatible with the process (**3b–e**). Additionally, higher yields were observed with substrates substituted by an electron-withdrawing group, presumably due to the increased reactivity of the aromatic C–H bonds from the increased acidity of these bonds and/or increased electronegativity of a copper intermediate which facilitates the sp^3^ C–H bond activation.^4^ Furthermore, 1,2,3,5-tetrafluorobenzene, 1,2,4,5-tetrafluorobenzene, and trifluorobenzenes with an additional electron-withdrawing group were effective substrates (**3f–i**). Not surprisingly, low reactivity was observed with 1,3,5-trifluorobenzene, 1,2,4-trifluorobenzene, and 3,5-difluorobenzonitrile. Delightfully, successful couplings of these substrates were achieved at an elevated reaction temperature (**3j–l**). Interestingly, a high regioselectivity was also observed in these cases by favoring the C–H bonds between the two C–F bonds, the most acidic C–H bonds.^10^ Furthermore, fluoropyridines were also effective coupling partners for this process (**3m** and **3n**). Unfortunately, other heteroaromatic substrates such as benzoxazoles, thiophenes, indoles *etc.* failed to provide any desired products under the current conditions.

**Table 2:** Scope of fluoroarenes^*a*^ ^*b*^

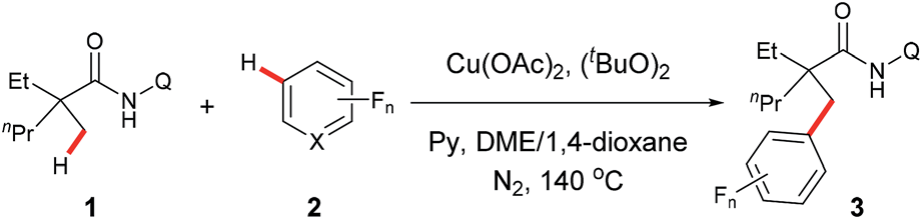
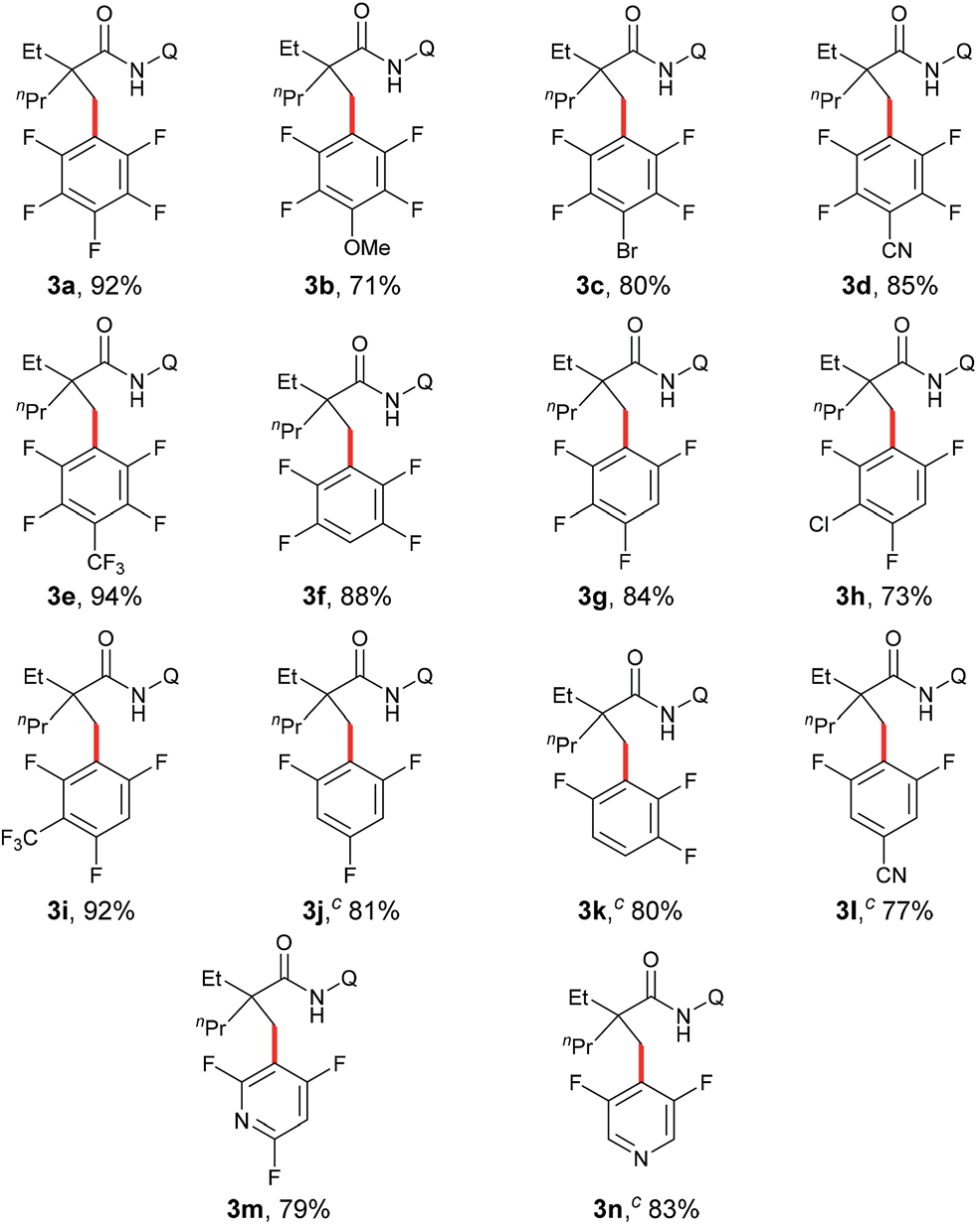

^*a*^Reaction conditions: **1a** (0.3 mmol), **2** (0.6 mmol), Cu(OAc)_2_ (0.3 mmol), (^*t*^BuO)_2_ (0.75 mmol), Py (0.9 mmol), DME/1,4-dioxane (v/v = 7 : 3, 1.0 mL), 140 °C, 16 h.

^*b*^Isolated yield.

^*c*^ **2** (1.2 mmol), run at 160 °C.

Next, the substrate scope study of aliphatic amides was investigated (Table 3). As expected, 2,2-disubstituted propanamides bearing either the linear or cyclic chains provided the corresponding desired products in good yields with good functional group compatibility. In addition, a predominant preference for functionalizing the C–H bonds of the α-methyl over those of the α-methylene, β- or γ-methyl groups, was observed in all cases, presumably due to the steric effect.^9^ Notably, both mono- and di-pentafluoro-substituted coupling products were obtained with 2,2-dimethyl butanamide and trifluropropanamide (**3w** and **3x**). Interestingly, only the mono-coupling products were observed with α-phthalimide and α-sulfone-substituted amides, which is believed to be due to the steric effects (**3y** and **3z**). Not surprisingly, both mono- and bis-coupling products were isolated with *N*-(quinolin-8-yl)pivalamide (**3aa**). Moreover, it was found that a tertiary α-carbon is necessary for this reaction since amides **4** and **5** failed to provide the corresponding desired products. Unfortunately, functionalization of secondary β-sp^3^ carbons was not successful (**6** and **7**).

**Table 3:** Scope of amides^*a*^ ^*b*^

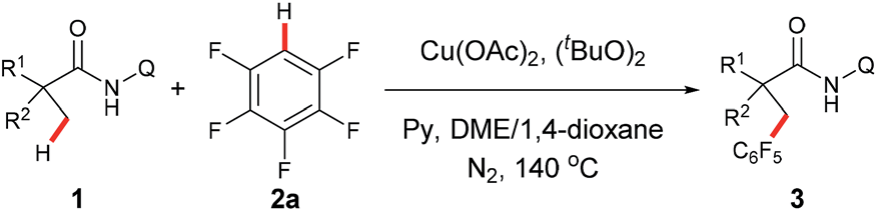
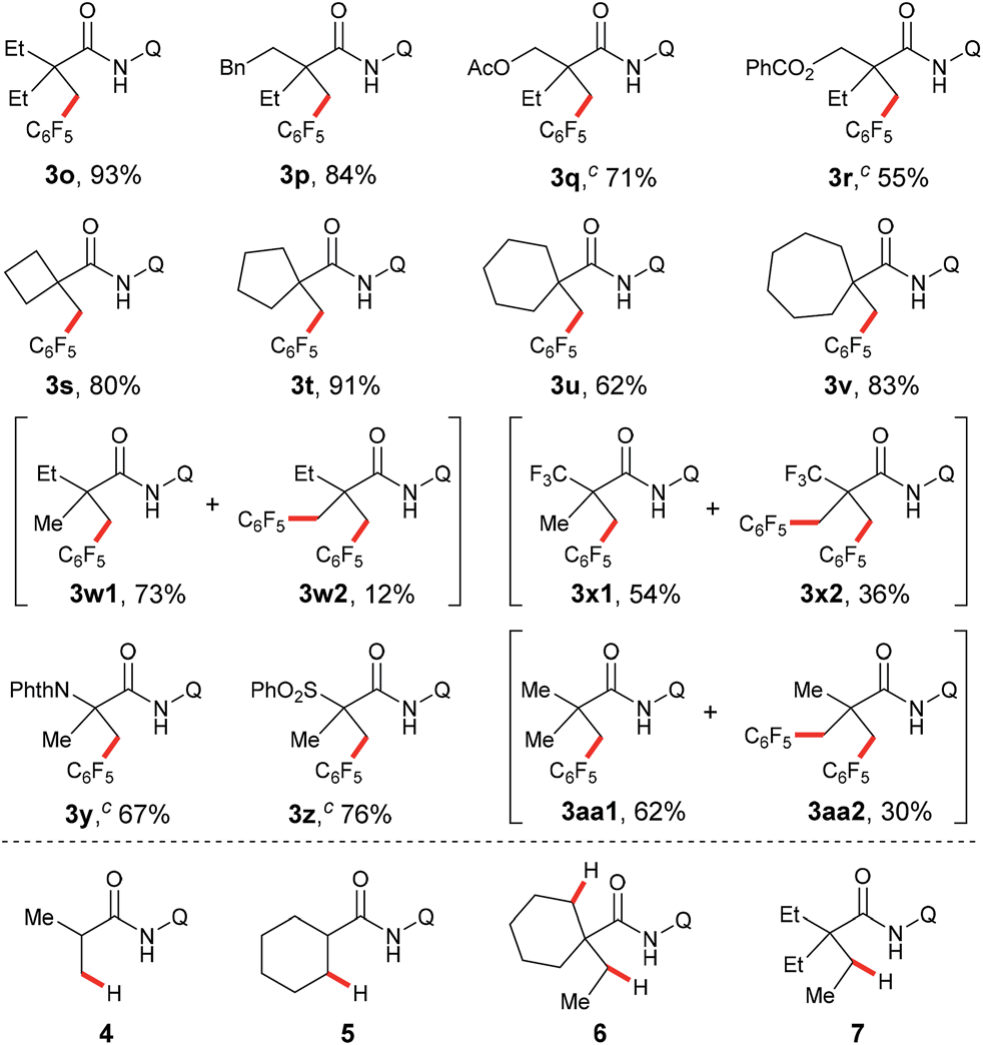

^*a*^Reaction conditions: **1** (0.3 mmol), **2a** (0.6 mmol), Cu(OAc)_2_ (0.3 mmol), (^*t*^BuO)_2_ (0.75 mmol), Py (0.9 mmol), DME/1,4-dioxane (v/v = 7 : 3, 1.0 mL), 140 °C, 16 h.

^*b*^Isolated yield.

^*c*^Run at 160 °C.

**Scheme 2:**
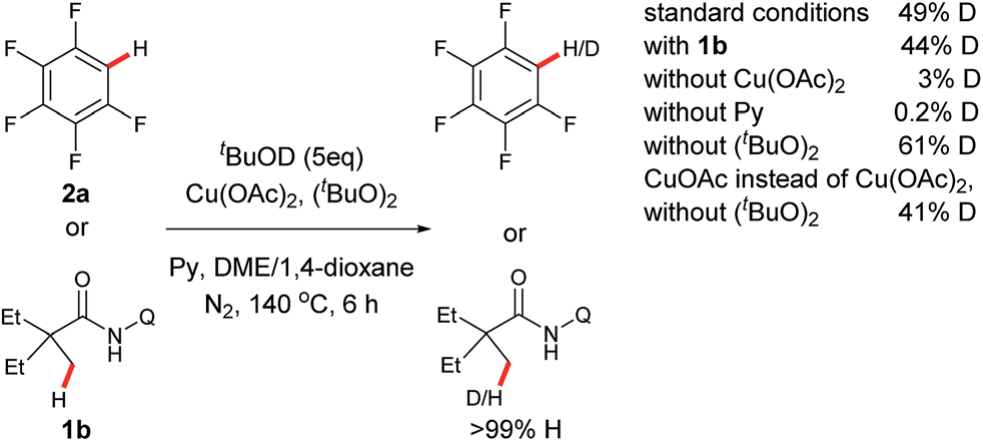
Deuterium labeling experiments.

To gain some mechanistic insights into this reaction, a series of deuterium-labeling experiments were carried out (Scheme 2). Considering that ^*t*^BuOH is generated from (^*t*^BuO)_2_ as a byproduct in the process, stoichiometric amounts of ^*t*^BuOD was added to the reaction system for this study. It was noted that an apparent H/D exchange occurred with pentafluorobenzene (**2a**) with or without 2-ethyl-2-methyl-*N*-(quinolin-8-yl)butanamide (**1b**), indicating that C–H bond cleavage of fluorobenzene is a reversible step. Furthermore, either a small or trace amount of [D]-**2a** was observed in the absence of Cu(OAc)_2_ or pyridine, while the obvious H–D scrambling occurred without (^*t*^BuO)_2_. It should be mentioned that H–D scrambling could also be promoted by CuOAc instead of Cu(OAc)_2_ in the absence of (^*t*^BuO)_2_. These results suggest that the copper species promotes the sp^2^ C–H bond cleavage with the pyridine as a base and ligand to facilitate the process, and a pyridine-coordinated aryl copper^II^ or aryl copper^I^ intermediate may be involved in the reaction.4e–i In the study, it was found that H/D exchange did not happen with **1b** in the absence of **2a**, indicating that an aryl copper intermediate may be involved in the sp^3^ C–H bond cleavage step of the amide.

**Scheme 3:**
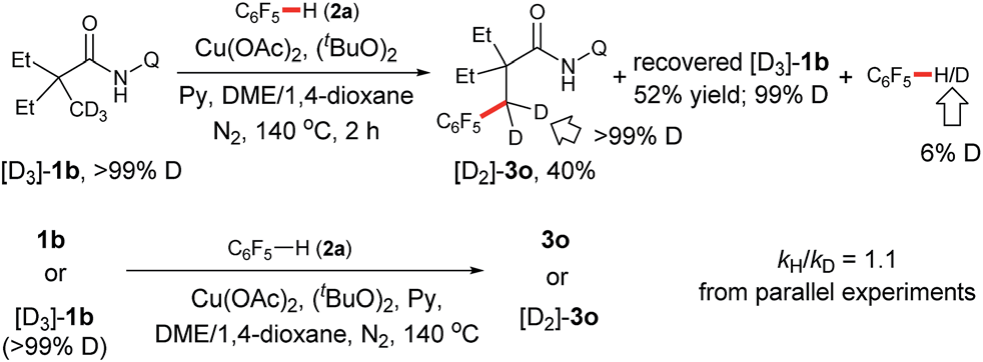
Deuterium labeling experiments of amides.

We further carried out deuterium-labeling experiments with [D_3_]-**1b**. As shown in Scheme 3, a H/D exchange was not observed with either this substrate or the product, suggesting that sp^3^ C–H bond cleavage is an irreversible step. In addition, a second order kinetic isotope effect was observed with **1b** in the process, indicating that cyclometalation of the amide is not the rate-limiting step.

**Scheme 4:**
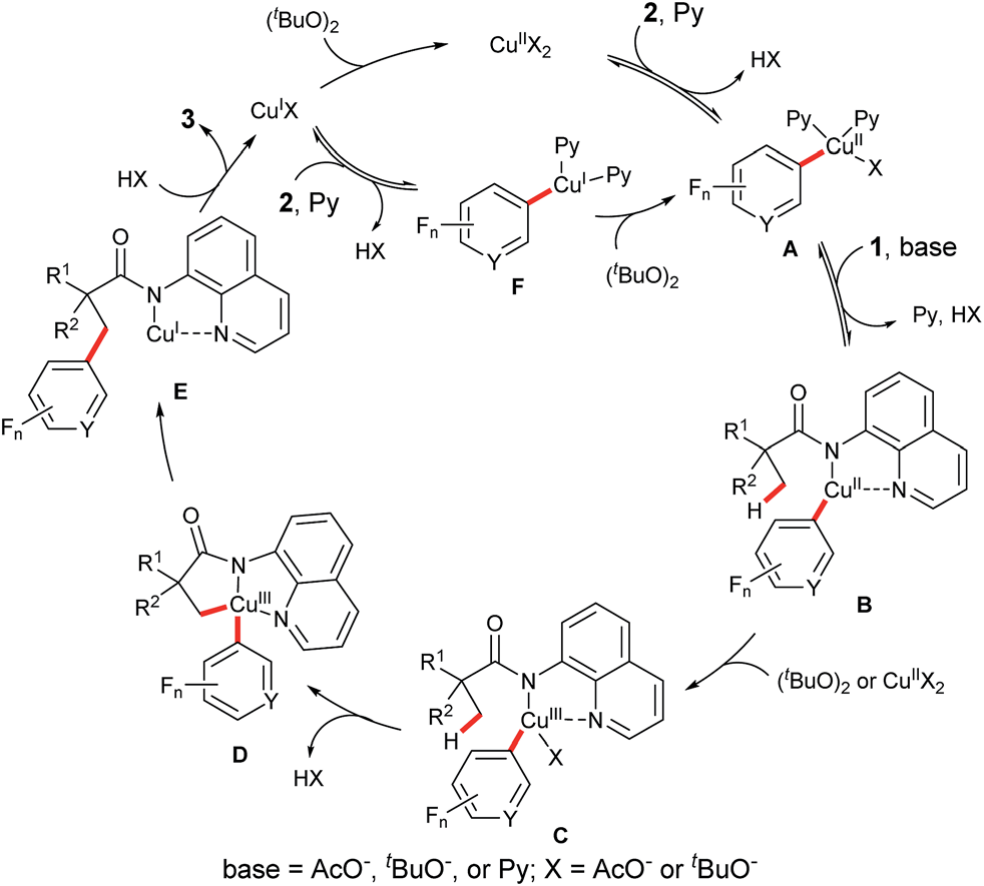
Plausible reaction mechanism.

On the basis of the above observed results and the previous reports,^4^,^9^,^12^ a plausible mechanism for this reaction is proposed (Scheme 4). It is believed that this process begins with the reversible C–H cupration of a fluoro(hetero)arene with Cu(OAc)_2_ in the presence of pyridine. Coordination of amide **1** to this Cu^II^ species followed by a ligand exchange step gives rise to the Cu^II^ intermediate **B**. Subsequent oxidation of the Cu^II^ species **B** generates the Cu^III^ intermediate **C**, which undergoes an intramolecular cyclometalation step to provide the Cu^III^ complex **D**. Reductive elimination of this intermediate followed by a ligand dissociation process affords the product **3** and a Cu^I^ species. Oxidation of the Cu^I^ species by (^*t*^BuO)_2_ regenerates the Cu^II^ species. Alternatively, on the basis of the observations in Scheme 2, the cupration of fluoro(hetero)arene **2** could take place with the Cu^I^ species, providing the Cu^I^ intermediate **F** which could then be oxidized to the Cu^II^ intermediate **A** by (^*t*^BuO)_2_.

## Conclusions

In summary, copper-promoted pyridine-enabled cross dehydrogenative coupling of aromatic sp^2^ C–H bonds and unactivated aliphatic sp^3^ C–H bonds was developed with high efficiency and good functional group tolerance. In this process, high regioselectivity was observed with sp^2^ C–H bond functionalization, favoring an sp^2^ C–H bond between two C–F bonds of (hetero)arenes. In addition, a predominant preference for functionalizing the sp^3^ C–H bonds of α-methyl groups over those of the α-methylene, β- or γ-methyl groups was observed with aliphatic amides. Mechanistic studies suggested that sp^2^ C–H bond cleavage is a reversible step while sp^3^ C–H bond cleavage is an irreversible but not the rate-limiting step. Interestingly, it was also found that sp^3^ C–H bond cleavage is dependent on sp^2^ C–H bond cleavage. The detailed mechanistic studies and potential synthetic applications of this process are currently under investigation in our laboratory.

## Supplementary Material

Supplementary information
