# Supplementary material for: Pyridine-enabled copper-promoted cross dehydrogenative coupling of C(sp2)–H and unactivated C(sp3)–H bonds
Source: Chem Sci. 2015 Jul 20;6(10):5978–83. doi: 10.1039/c5sc02143j (PMC5950755; doi:10.1039/c5sc02143j)
Supplement: Supplementary file 1 [file SC-006-C5SC02143J-s001.pdf]

Electronic Supplementary Material (ESI) for Chemical Science.
This journal is © The Royal Society of Chemistry 2015

Pyridine-Enabled Copper-Promoted Cross Dehydrogenative Coupling of C(sp^2)-H and C(sp^3)-H Bonds

Xuesong Wu, Yan Zhao, and Haibo Ge*

Table of Contents

General Information S2
Structures of Starting Materials S3
General Procedure for Copper-Promoted Cross Dehydrogenative Coupling of Amides S4
Analytical Data S4
Deuterium Labeling Experiment S20
Parallel KIE Experiment S22
References S23
^{1}H, ^{13}C and ^{19}F NMR Spectra S24

General Information

^{1}H, ^{13}C and ^{19}F NMR spectra were recorded on a Bruker 500 MHz NMR Fourier transform spectrometer (500 MHz and 125 MHz, respectively) using tetramethylsilane as an internal reference, and chemical shifts (δ) and coupling constants (J) were expressed in ppm and Hz, respectively. Infrared spectra were obtained using a Thermo Nicolet IR 330 spectrometer. Mass (MS) analysis were abtained using Agilent 1100 series LC/MSD system with Electrospray Ionization (ESI). All the solvents and commercially available reagents were purchased from commercial sources and used directly.

Starting materials **1a-d**, **1f-n**, [D_3]**-1b** and **4-7** were prepared according to literature procedures.[1,2] **1e** were prepared from **1b** based on reported reaction protocol.[2]

Structures of Starting Materials

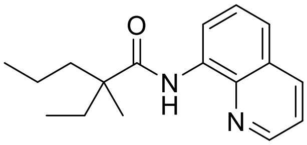

1a

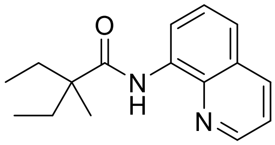

1b

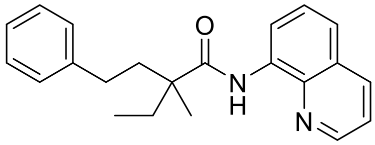

1c

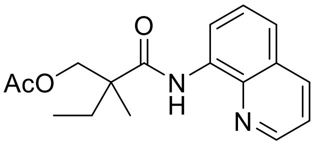

1d

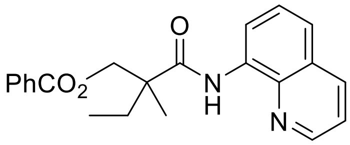

1e

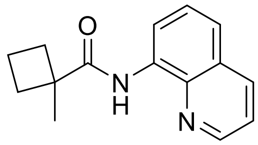

1f

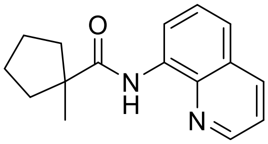

1g

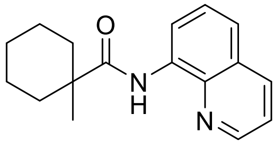

1h

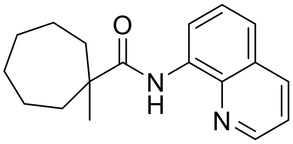

1i

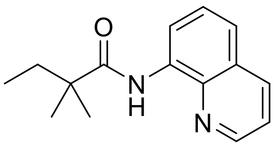

1j

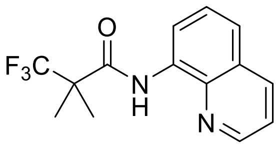

1k

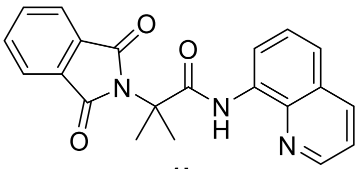

1l

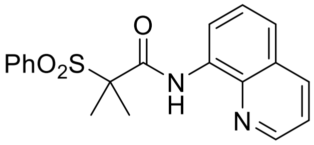

1m

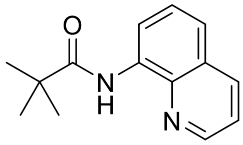

1n

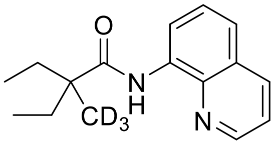

$[D_3]$-**1b**

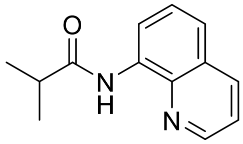

4

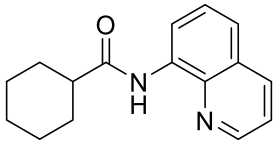

5

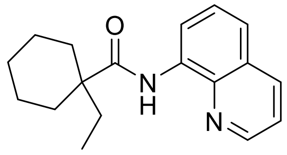

6

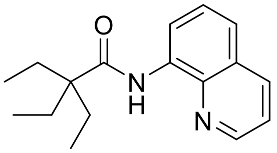

7

General Procedure for Copper-Promoted Cross Dehydrogenative Coupling of Amides

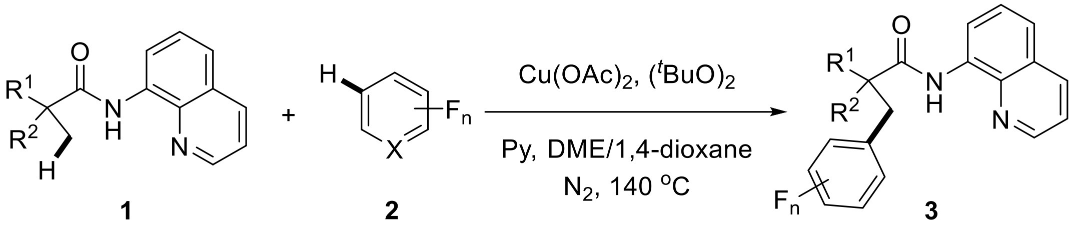

A 50 mL Schlenk tube was charged with amide (**1**, 0.30 mmol), $Cu(OAc)_2$ (54.5 mg, 0.30 mmol), DME/1,4-dioxane (7:3, v/v, 1.0 mL), pyridine (72 μL, 71 mg, 0.90 mmol), *tert*-butyl peroxide (138 μL, 110 mg, 0.75 mmol) and fluoroarene (0.60 mmol) in sequence under N_2 atmosphere. The tube was capped with a Teflon screw cap, and stirred at 140 °C for 16h. Then the reaction mixture was cooled to room temperature, diluted with EtOAc (30 mL). The organic layer was washed with 5% aqueous NaOH (10 mL), water (20 mL), brine (10 mL), dried over anhydrous sodium sulfate, and concentrated under reduced pressure. The residue was purified by flash column chromatography on silica gel, eluting with CH_2Cl_2/Hexane (1:2 ~ 3:1, v/v), to afford corresponding product **3**.

Analytical Data

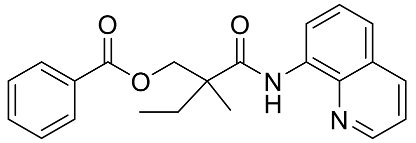

1e

Compound **1e**, colorless oil, R_f = 0.33 (hexane/EtOAc 4:1). ^{1}H NMR (500 MHz, $CDCl_3$) δ 10.50 (brs, 1H), 8.83 (dd, J = 7.4, 1.5 Hz, 1H), 8.57 (dd, J = 4.2, 1.7 Hz, 1H), 8.15 (dd, J = 8.3, 1.6 Hz, 1H), 8.08 (dd, J = 8.3, 1.2 Hz, 2H), 7.56 – 7.49 (m, 3H), 7.41 (dd, J = 8.3, 4.2 Hz, 1H), 7.36 – 7.31 (m, 2H), 4.58 (d, J = 11.2 Hz, 1H), 4.50 (d, J = 11.2 Hz, 1H), 2.10 – 2.01 (m, 1H), 1.88 – 1.78 (m, 1H), 1.53 (s, 3H), 1.04 (t, J = 7.5 Hz, 3H); ^{13}C NMR (125 MHz, $CDCl_3$) δ 173.7, 166.5, 148.3, 138.9, 136.4, 134.7, 133.1, 130.1, 130.0, 128.4, 128.1, 127.6, 121.7 (2C), 116.7, 69.9, 47.9, 29.5, 19.7, 8.8; IR (neat) ν 3359, 2968, 1722, 1682, 1529, 1487, 1424, 1384, 1325, 1270, 1111, 826, 791, 711; Ms (ESI): m/z = 363.2 $[M+H]^+$.

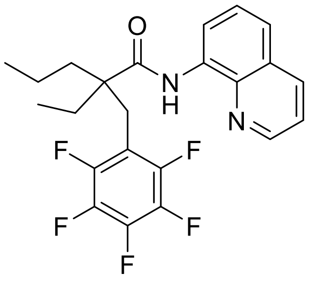

3a

Compound **3a**, colorless oil, yield: 92%, R_f = 0.55 (hexane/EtOAc 5:1). ^{1}H NMR (500 MHz, $CDCl_3$) δ 10.06 (brs, 1H), 8.76 – 8.71 (m, 2H), 8.12 (dd, *J* = 8.3, 1.6 Hz, 1H), 7.55 – 7.40 (m, 3H), 3.11 – 3.03 (m, 2H), 1.97 – 1.88 (m, 1H), 1.87 – 1.79 (m, 2H), 1.76 – 1.68 (m, 1H), 1.50 – 1.39 (m, 2H), 1.05 (t, *J* = 7.4 Hz, 3H), 1.00 (t, *J* = 7.3 Hz, 3H); ^{13}C NMR (125 MHz, $CDCl_3$) δ 173.3, 148.2, 145.9 (dm, *J* = 244.9 Hz), 140.0 (dm, *J* = 250.8 Hz), 138.8, 137.4 (dm, *J* = 249.0 Hz), 136.4, 134.3, 128.0, 127.6, 121.6, 121.5, 116.6, 111.7 (m), 51.7, 36.4, 29.6, 27.1, 17.6, 14.7, 8.7; ^{19}F NMR (471 MHz, $CDCl_3$) δ -139.6 (dd, *J* = 22.6, 7.5 Hz, 2F), -156.7 (t, *J* = 20.9 Hz, 1F), -163.2 (m, 2F); IR (neat) ν 3357, 2964, 2875, 1683, 1520, 1424, 1385, 1325, 1263, 1124, 1022, 959, 908, 826, 791, 737; Ms (ESI): *m*/*z* = 437.2 $[M+H]^+$.

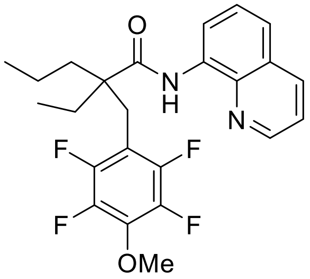

3b

Compound **3b**, white solid, yield: 71%, R_f = 0.45 (hexane/EtOAc 5:1). ^{1}H NMR (500 MHz, $CDCl_3$) δ 10.06 (brs, 1H), 8.76 – 8.72 (m, 2H), 8.14 (dd, *J* = 8.3, 1.6 Hz, 1H), 7.56 – 7.52 (m, 1H), 7.48 (dd, *J* = 8.3, 1.3 Hz, 1H), 7.43 (dd, *J* = 8.2, 4.2 Hz, 1H), 3.95 (t, *J* = 1.1 Hz, 3H), 3.12 – 3.02 (m, 2H), 1.95 – 1.87 (m, 1H), 1.85 – 1.77 (m, 2H), 1.74 – 1.66 (m, 1H), 1.50 – 1.40 (m, 2H), 1.04 (t, *J* = 7.4 Hz, 3H), 0.99 (t, *J* = 7.3 Hz, 3H); ^{13}C NMR (125 MHz, $CDCl_3$) δ 173.7, 148.2, 146.2 (dm, *J* = 243.0 Hz), 140.8 (dm, *J* = 244.9 Hz), 138.8, 137.0 (m), 136.4, 134.5, 128.0, 127.7, 121.6, 121.4, 116.6, 109.9 (m), 62.1, 51.7, 36.7, 29.5, 27.2, 17.7, 14.8, 8.8; ^{19}F NMR (471 MHz, $CDCl_3$) δ -141.3 (dd, *J* = 21.7, 8.3 Hz, 2F), -159.0 (dd, *J* = 21.7, 8.3 Hz, 2F); IR (neat) ν 3357, 2963, 2874, 1682, 1529, 1489, 1385, 1325, 1131, 1015, 960, 826, 792, 733; Ms (ESI): *m*/*z* = 449.2 $[M+H]^+$.

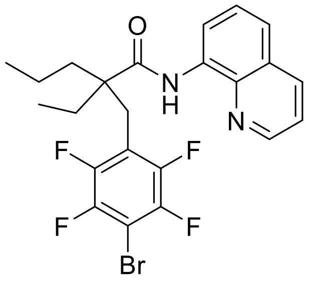

3c

Compound **3c**, colorless oil, yield: 80%, R_f = 0.55 (hexane/EtOAc 5:1). ^{1}H NMR (500 MHz, $CDCl_3$) δ 10.03 (brs, 1H), 8.73 – 8.70 (m, 2H), 8.15 (dd, J = 8.3, 1.6 Hz, 1H), 7.56 – 7.48 (m, 2H), 7.43 (dd, J = 8.2, 4.2 Hz, 1H), 3.14 – 3.06 (m, 2H), 1.97 – 1.89 (m, 1H), 1.87 – 1.79 (m, 2H), 1.77 – 1.69 (m, 1H), 1.50 – 1.39 (m, 2H), 1.05 (t, J = 7.4 Hz, 3H), 1.00 (t, J = 7.3 Hz, 3H); ^{13}C NMR (125 MHz, $CDCl_3$) δ 173.3, 148.3, 146.0 (dm, J = 246.8 Hz), 144.8 (dm, J = 245.0 Hz), 138.8, 136.7 (m), 136.4, 134.3, 128.0, 127.6, 121.7, 121.5, 166.6, 98.1 (m), 51.8, 36.5, 30.2, 27.2, 17.7, 14.8, 8.7; ^{19}F NMR (471 MHz, $CDCl_3$) δ -134.6 (m, 2F), -138.2 (dd, J = 21.5, 8.6 Hz, 2F); IR (neat) ν 3356, 2964, 2874, 1678, 1529, 1485, 1385, 1325, 1261, 1142, 948, 909, 826, 791, 733; Ms (ESI): m/z = 497.1 $[M+H]^+$.

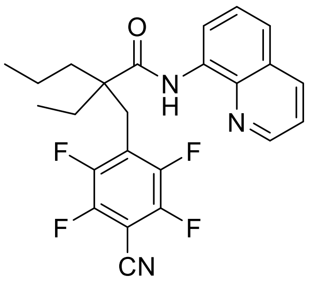

3d

Compound **3d**, colorless oil, yield: 85%, R_f = 0.36 (hexane/EtOAc 5:1). ^{1}H NMR (500 MHz, $CDCl_3$) δ 10.06 (brs, 1H), 8.75 (dd, J = 4.2, 1.7 Hz, 1H), 8.68 (dd, J = 7.0, 1.9 Hz, 1H), 8.18 (dd, J = 8.3, 1.6 Hz, 1H), 7.57 – 7.51 (m, 2H), 7.48 (dd, J = 8.3, 4.2 Hz, 1H), 3.18 – 3.10 (m, 2H), 2.00 – 1.91 (m, 1H), 1.90 – 1.81 (m, 2H), 1.78 – 1.71 (m, 1H), 1.52 – 1.36 (m, 2H), 1.06 (t, J = 7.4 Hz, 3H), 1.02 (t, J = 7.3 Hz, 3H); ^{13}C NMR (125 MHz, $CDCl_3$) δ 172.7, 147.3, 146.9 (dm, J = 259.8 Hz), 145.7 (dm, J = 247.0 Hz), 138.8, 136.6, 134.1, 128.0, 127.7, 124.9 (m), 121.8 (2C), 116.7, 107.7, 92.7 (m), 52.2, 36.4, 31.0, 27.1, 17.7, 14.8, 8.7; ^{19}F NMR (471 MHz, $CDCl_3$) δ -133.7 (m, 2F), -136.4 (m, 2F); IR (neat) ν 3361, 2965, 2875, 2246, 1675, 1530, 1491, 1385, 1325, 1298, 956, 826, 792, 738; Ms (ESI): m/z = 444.2 $[M+H]^+$.

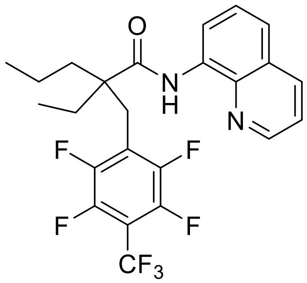

3e

Compound **3e**, colorless oil, yield: 94%, R_f = 0.54 (hexane/EtOAc 5:1). ^{1}H NMR (500 MHz, $CDCl_3$) δ 10.06 (brs, 1H), 8.73 – 8.70 (m, 2H), 8.16 (dd, J = 8.3, 1.6 Hz, 1H), 7.56 – 7.49 (m, 2H), 7.44 (dd, J = 8.3, 4.2 Hz, 1H), 3.18 – 3.10 (m, 2H), 1.99 – 1.90 (m, 1H), 1.89 – 1.81 (m, 2H), 1.78 – 1.71 (m, 1H), 1.52 – 1.39 (m, 2H), 1.06 (t, J = 7.4 Hz, 3H), 1.01 (t, J = 7.3 Hz, 3H); ^{13}C NMR (125 MHz, $CDCl_3$) δ 173.0, 148.3, 146.1 (dm, J = 246.1 Hz), 143.9 (dm, J = 257.4 Hz), 138.8, 136.5, 134.2, 128.0, 127.6, 122.0 (m), 121.7, 121.6, 120.9 (q, J = 269.6 Hz), 116.6, 108.6 (m), 52.0, 36.5, 30.4, 27.2, 17.7, 14.8, 8.7; ^{19}F NMR (471 MHz, $CDCl_3$) δ -56.3 (t, J = 21.4 Hz, 3F), -137.77 (m, 2F), -141.9 (m, 2F); IR (neat) ν 3357, 2965, 2876, 1683, 1530, 1492, 1331, 1147, 960, 826, 792, 715; Ms (ESI): m/z = 487.1 $[M+H]^+$.

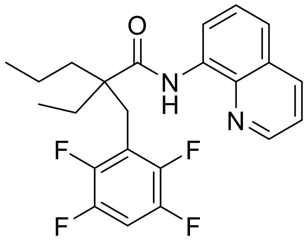

3f

Compound **3f**, colorless oil, yield: 88%, R_f = 0.52 (hexane/EtOAc 5:1). ^{1}H NMR (500 MHz, $CDCl_3$) δ 10.08 (brs, 1H), 8.76 – 8.72 (m, 2H), 8.14 (dd, J = 8.3, 1.6 Hz, 1H), 7.56 – 7.41 (m, 3H), 6.90 – 6.82 (m, 1H), 3.18 – 3.10 (m, 2H), 1.96 – 1.88 (m, 1H), 1.87 – 1.78 (m, 2H), 1.76 – 1.68 (m, 1H), 1.50 – 1.41 (m, 2H), 1.05 (t, J = 7.4 Hz, 3H), 0.99 (t, J = 7.3 Hz, 3H); ^{13}C NMR (125 MHz, $CDCl_3$) δ 173.6, 148.2, 145.7 (dm, J = 246.6 Hz), 138.9, 136.4, 134.5, 128.0, 127.7, 121.6, 121.4, 117.7 (m), 116.6, 104.5 (m), 51.8, 36.8, 29.9, 27.3, 17.7, 14.8, 8.8; ^{19}F NMR (471 MHz, $CDCl_3$) δ -140.1 (m, 4F); IR (neat) ν 3357, 3054, 2964, 2875, 1683, 1505, 1386, 1325, 1253, 1171, 938, 826, 792, 738; Ms (ESI): m/z = 419.2 $[M+H]^+$.

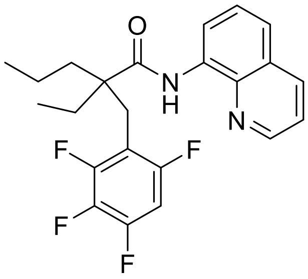

3g

Compound **3g**, colorless oil, yield: 84%, R_f = 0.52 (hexane/EtOAc 5:1). ^{1}H NMR (500 MHz, $CDCl_3$) δ 10.04 (brs, 1H), 8.75 – 8.71 (m, 2H), 8.15 (dd, J = 8.3, 1.6 Hz, 1H), 7.56 – 7.47 (m, 2H), 7.43 (dd, J = 8.2, 4.2 Hz, 1H), 6.62 – 6.55 (m, 1H), 3.09 – 3.00 (m, 2H), 1.95 – 1.87 (m, 1H), 1.85 – 1.77 (m, 2H), 1.74 – 1.67 (m, 1H), 1.48 – 1.39 (m, 2H), 1.04 (t, J = 7.4 Hz, 3H), 0.99 (t, J = 7.3 Hz, 3H); ^{13}C NMR (125 MHz, $CDCl_3$) δ 173.7, 156.3 (dm, J = 244.0 Hz), 150.8 (dm, J = 247.6 Hz), 149.4 (dm, J = 248.0 Hz), 148.2, 138.8, 137.2 (dm, J = 246.3 Hz), 136.4, 134.5, 128.0, 127.6, 121.6, 121.4, 116.5, 111.6 (m), 100.3 (m), 51.6, 36.5, 29.5, 27.1, 17.6, 14.8, 8.7; ^{19}F NMR (471 MHz, $CDCl_3$) δ -114.6 (t, J = 10.0 Hz, 1F), -132.2 (d, J = 18.9 Hz, 1F), -134.8 (m, 1F), -165.7 (m, 1F); IR (neat) ν 3357, 3052, 2964, 2874, 1678, 1528, 1385, 1325, 1254, 1114, 1052, 909, 826, 791, 734; Ms (ESI): m/z = 419.2 $[M+H]^+$.

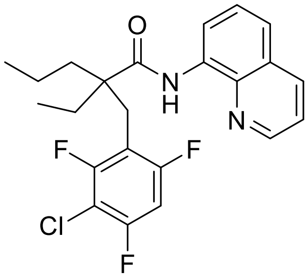

3h

Compound **3h**, colorless oil, yield: 73%, R_f = 0.50 (hexane/EtOAc 5:1). ^{1}H NMR (500 MHz, $CDCl_3$) δ 9.99 (brs, 1H), 8.74 (dd, J = 7.6, 1.3 Hz, 1H), 8.69 (dd, J = 4.2, 1.7 Hz, 1H), 8.14 (dd, J = 8.3, 1.6 Hz, 1H), 7.56 – 7.40 (m, 3H), 6.63 – 6.57 (m, 1H), 3.08 – 2.98 (m, 2H), 1.96 – 1.86 (m, 1H), 1.85 – 1.77 (m, 2H), 1.75 – 1.67 (m, 1H), 1.49 – 1.39 (m, 2H), 1.04 (t, J = 7.4 Hz, 3H), 1.00 (t, J = 7.2 Hz, 3H). ^{13}C NMR (125 MHz, $CDCl_3$) δ 173.7, 159.9 (dm, J = 247.1 Hz), 158.2 (dm, J = 248.1 Hz), 157.3 (dm, J = 248.1 Hz), 148.2, 138.8, 136.4, 134.5, 128.0, 127.6, 121.6, 121.4, 116.5, 111.4 (m), 105.9 (m), 100.4 (m), 51.7, 36.4, 29.8, 27.0, 17.6, 14.9, 8.8; ^{19}F NMR (471 MHz, $CDCl_3$) δ -110.0 (m, 2F), -113.1 (m, 1F); IR (neat) ν 3357, 3053, 2963,

2874, 1682, 1527, 1486, 1436, 1385, 1149, 1047, 909, 826, 791, 736; Ms (ESI): *m*/*z* = 435.1 $[M+H]^+$.

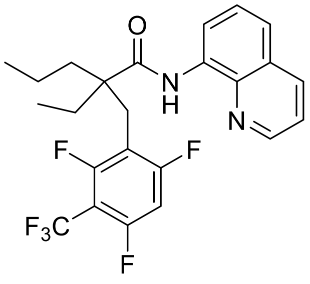

3i

Compound **3i**, colorless oil, yield: 92%, R_f = 0.51 (hexane/EtOAc 3:1). ^{1}H NMR (500 MHz, $CDCl_3$) δ 10.01 (brs, 1H), 8.73 – 8.68 (m, 2H), 8.14 (dd, *J* = 8.3, 1.6 Hz, 1H), 7.55 – 7.47 (m, 2H), 7.42 (dd, *J* = 8.2, 4.2 Hz, 1H), 6.63 – 6.57 (m, 1H), 3.06 – 2.98 (m, 2H), 1.97 – 1.88 (m, 1H), 1.87 – 1.78 (m, 2H), 1.75 – 1.68 (m, 1H), 1.50 – 1.38 (m, 2H), 1.04 (t, *J* = 7.4 Hz, 3H), 1.00 (t, *J* = 7.3 Hz, 3H); ^{13}C NMR (125 MHz, $CDCl_3$) δ 173.4, 164.0 (dm, *J* = 253.1 Hz), 159.8 (dm, *J* = 257.0 Hz), 159.0 (dm, *J* = 257.8 Hz), 148.2, 138.8, 136.4, 134.4, 128.0, 127.6, 121.6, 121.5 (q, *J* = 272.1 Hz), 121.5, 116.5, 112.0 (m), 104.4 (m), 101.0 (m), 51.7, 36.2, 29.6, 26.9, 17.6, 14.8, 8.7; ^{19}F NMR (471 MHz, $CDCl_3$) δ -56.5 (t, *J* = 22.8 Hz, 3F), -101.3 (q, *J* = 9.1 Hz, 1F), -107.95 (m, 1F), -110.7 (m, 1F); IR (neat) ν 3357, 2964, 2875, 1676, 1634, 1530, 1487, 1450, 1307, 1139, 1054, 910, 826, 792, 735; Ms (ESI): *m*/*z* = 469.2 $[M+H]^+$.

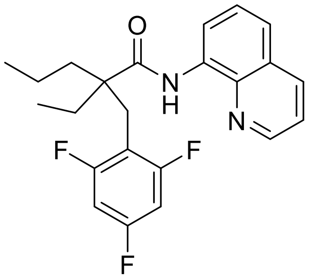

3j

Compound **3j**, colorless oil, yield: 81%, R_f = 0.48 (hexane/EtOAc 5:1). ^{1}H NMR (500 MHz, $CDCl_3$) δ 10.04 (brs, 1H), 8.76 (dd, *J* = 7.6, 1.1 Hz, 1H), 8.71 (dd, *J* = 4.2, 1.6 Hz, 1H), 8.13 (dd, *J* = 8.3, 1.6 Hz, 1H), 7.55 – 7.46 (m, 2H), 7.41 (dd, *J* = 8.2, 4.2 Hz, 1H), 6.52 – 6.46 (m, 2H), 3.07 – 2.98 (m, 2H), 1.95 – 1.86 (m, 1H), 1.85 – 1.77 (m, 2H), 1.73 – 1.66 (m, 1H), 1.48 – 1.39 (m, 2H), 1.03 (t, *J* = 7.4 Hz, 3H),

0.99 (t, J = 7.3 Hz, 3H); ^{13}C NMR (125 MHz, $CDCl_3$) δ 174.0, 162.3 (ddd, J = 248.4, 14.8, 11.6 Hz), 161.5 (dt, J = 247.8, 15.9 Hz). 148.2, 138.8, 136.3, 134.7, 128.0, 127.6, 121.6, 121.2, 116.4, 110.1 (m), 99.9 (m), 51.6, 36.5, 29.1, 27.1, 17.6, 14.8, 8.8; ^{19}F NMR (471 MHz, $CDCl_3$) δ -108.2 (t, J = 6.8 Hz, 2F), -110.6 (m, 1F); IR (neat) ν 3359, 3053, 2963, 2874, 1683, 1624, 1538, 1488, 1385, 1325, 1117, 1042, 1000, 840, 791, 736; Ms (ESI): m/z = 401.2 $[M+H]^+$.

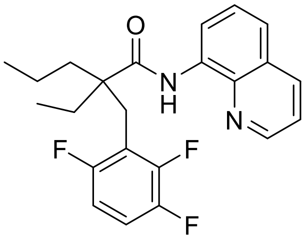

3k

Compound **3k**, colorless oil, yield: 80%, R_f = 0.47 (hexane/EtOAc 5:1). ^{1}H NMR (500 MHz, $CDCl_3$) δ 10.07 (brs, 1H), 8.76 (dd, J = 7.6, 1.3 Hz, 1H), 8.72 (dd, J = 4.2, 1.7 Hz, 1H), 8.13 (dd, J = 8.3, 1.6 Hz, 1H), 7.55 – 7.51 (m, 1H), 7.48 (dd, J = 8.3, 1.3 Hz, 1H), 7.41 (dd, J = 8.2, 4.2 Hz, 1H), 6.94 – 6.87 (m, 1H), 6.67 – 6.62 (m, 1H), 3.15 – 3.07 (m, 2H), 1.96 – 1.87 (m, 1H), 1.86 – 1.77 (m, 2H), 1.75 – 1.66 (m, 1H), 1.50 – 1.41 (m, 2H), 1.05 (t, J = 7.4 Hz, 3H), 0.99 (t, J = 7.3 Hz, 3H); ^{13}C NMR (125 MHz, $CDCl_3$) δ 173.9, 157.5 (dm, J = 242.5 Hz), 149.9 (dm, J = 247.4 Hz), 148.2, 147.2 (dm, J = 242.4 Hz), 138.9, 136.3, 134.6, 128.0, 127.6, 121.6, 121.3, 116.5, 116.2 (dd, J = 22.5, 16.9 Hz), 115.1 (dd, J = 18.9, 9.7 Hz), 110.18 (m), 51.8, 36.8, 29.6, 27.4, 17.7, 14.8, 8.8; ^{19}F NMR (471 MHz, $CDCl_3$) δ -116.2 (m, 1F), -134.3 (dd, J = 21.1, 8.5 Hz, 1F), -142.9 (m, 1F); IR (neat) ν 3359, 2963, 2874, 1679, 1529, 1493, 1385, 1325, 1243, 1145, 1029, 942, 825, 791, 737; Ms (ESI): m/z = 401.2 $[M+H]^+$.

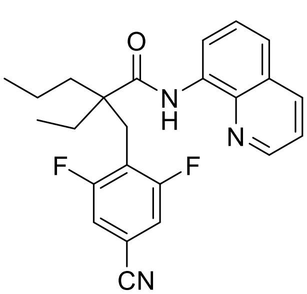

3l

Compound **3l**, colorless oil, yield: 77%, R_f = 0.45 (hexane/EtOAc 3:1). ^{1}H NMR (500 MHz, $CDCl_3$) δ 10.02 (brs, 1H), 8.72 (dd, *J* = 9.0, 2.7 Hz, 2H), 8.15 (dd, *J* = 8.2, 1.5 Hz, 1H), 7.56 – 7.48 (m, 2H), 7.44 (dd, *J* = 8.2, 4.2 Hz, 1H), 7.05 – 7.00 (m, 2H), 3.13 – 3.05 (m, 2H), 1.96 – 1.89 (m, 1H), 1.88 – 1.79 (m, 2H), 1.76 – 1.68 (m, 1H), 1.50 – 1.36 (m, 2H), 1.04 (t, *J* = 7.4 Hz, 3H), 1.00 (t, *J* = 7.2 Hz, 3H); ^{13}C NMR (125 MHz, $CDCl_3$) δ 173.3, 162.1 (d, *J* = 249.9 Hz), 162.0 (d, *J* = 249.9 Hz), 148.2, 138.8, 136.4, 134.4, 128.0, 127.6, 121.7, 121.5, 121.0 (t, *J* = 20.1 Hz), 116.8 (t, *J* = 3.4 Hz), 116.5, 115.2 (d, *J* = 22.8 Hz), 115.1 (d, *J* = 22.6 Hz), 111.8 (t, *J* = 12.3 Hz), 51.9, 36.4, 30.0, 27.1, 17.6, 14.8, 8.7; ^{19}F NMR (471 MHz, $CDCl_3$) δ -107.0 (d, J = 6.0 Hz, 2F); IR (neat) ν 3358, 3065, 2963, 2874, 2232, 1675, 1526, 1487, 1423, 1385, 1325, 1138, 1043, 972, 861, 826, 792, 736; Ms (ESI): *m*/*z* = 408.2 $[M+H]^+$.

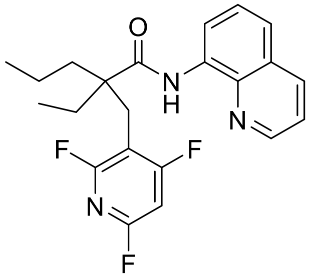

3m

Compound **3m**, pale yellow oil, yield: 79%, R_f = 0.38 (hexane/EtOAc 2:1). ^{1}H NMR (500 MHz, $CDCl_3$) δ 10.08 (brs, 1H), 8.75 – 8.71 (m, 2H), 8.15 (dd, *J* = 8.3, 1.6 Hz, 1H), 7.56 – 7.48 (m, 2H), 7.44 (dd, *J* = 8.2, 4.2 Hz, 1H), 6.40 (dd, *J* = 8.0, 2.1 Hz, 1H), 3.04 – 2.96 (m, 2H), 1.95 – 1.87 (m, 1H), 1.85 – 1.77 (m, 2H), 1.74 – 1.66 (m, 1H), 1.49 – 1.39 (m, 2H), 1.04 (t, *J* = 7.4 Hz, 3H), 1.00 (t, *J* = 7.3 Hz, 3H); ^{13}C NMR (125 MHz, $CDCl_3$) δ 173.5, 172.0 (dm, *J* = 261.3 Hz), 161.4 (dm, *J* = 242.1 Hz), 160.7 (dm, *J* = 241.9 Hz), 148.3, 138.9, 136.5, 134.4, 128.0, 127.7, 121.7, 121.5, 116.6, 105.9 (m), 95.0 (m), 51.7, 36.5, 29.0, 27.1, 17.6, 14.8, 8.7; ^{19}F NMR (471 MHz, $CDCl_3$) δ -66.3 (m, 1F), -67.7 (dd, *J* = 19.7, 13.4 Hz, 1F), -92.56 (m, 1F); IR (neat) ν 3357, 2963, 2874, 1675, 1616, 1528, 1472, 1414, 1385, 1142, 1018, 826, 792, 733; Ms (ESI): *m*/*z* = 402.2 $[M+H]^+$.

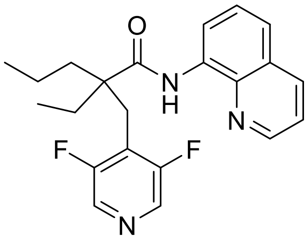

3n

Compound **3n**, pale yellow oil, yield: 83%, R_f = 0.33 (hexane/EtOAc 2:1). ^{1}H NMR (500 MHz, $CDCl_3$) δ 10.07 (brs, 1H), 8.75 – 8.70 (m, 2H), 8.17 (s, 2H), 8.14 (dd, *J* = 8.3, 1.6 Hz, 1H), 7.56 – 7.47 (m, 2H), 7.42 (dd, *J* = 8.2, 4.2 Hz, 1H), 3.17 – 3.09 (m, 2H), 1.94 – 1.86 (m, 1H), 1.85 – 1.76 (m, 2H), 1.75 – 1.67 (m, 1H), 1.50 – 1.40 (m, 2H), 1.05 (t, *J* = 7.4 Hz, 3H), 0.98 (t, *J* = 7.3 Hz, 3H); ^{13}C NMR (125 MHz, $CDCl_3$) δ 173.4, 158.8 (d, *J* = 257.9 Hz), 158.7 (d, *J* = 258.0 Hz), 148.3, 138.8, 136.4, 134.4, 134.0 (d, *J* = 24.6 Hz), 133.9 (d, *J* = 24.8 Hz), 128.0, 127.6, 122.8 (t, *J* = 17.3 Hz), 121.7, 121.5, 116.5, 51.8, 36.9, 28.9, 27.5, 17.6, 14.8, 8.7; ^{19}F NMR (471 MHz, $CDCl_3$) δ -126.3 (2F); IR (neat) ν 3357, 3049， 2963, 2874, 1682, 1527, 1486, 1421, 1385, 1325, 1140, 1040, 909, 826, 791, 733; Ms (ESI): *m*/*z* = 384.2 $[M+H]^+$.

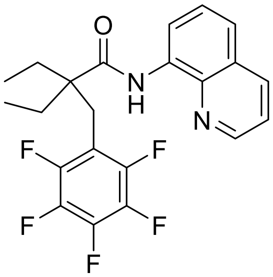

3o

Compound **3o**, colorless oil, yield: 93%, R_f = 0.53 (hexane/EtOAc 5:1). ^{1}H NMR (500 MHz, $CDCl_3$) δ 10.05 (brs, 1H), 8.75 – 8.71 (m, 2H), 8.14 (dd, *J* = 8.3, 1.6 Hz, 1H), 7.55 – 7.41 (m, 3H), 3.07 (s, 2H), 1.98 – 1.89 (m, 2H), 1.86 – 1.77 (m, 2H), 1.04 (t, *J* = 7.4 Hz, 6H); ^{13}C NMR (125 MHz, $CDCl_3$) δ 173.2, 148.2, 146.0 (dm, *J* = 244.8 Hz), 140.0 (dm, *J* = 250.8 Hz), 138.8, 138.4 (dm, *J* = 245.5 Hz), 136.4, 134.3, 128.0, 127.6, 121.6, 121.5, 116.6, 111.7 (m), 51.9, 29.4, 26.6, 8.6; ^{19}F NMR (471 MHz, $CDCl_3$) δ -139.3 (dd, *J* = 22.6, 7.7 Hz, 2F), -156.7 (t, *J* = 20.9 Hz, 1F), -163.2 (m, 2F); IR (neat) ν 3358, 3051, 2927, 2883, 1683, 1505, 1385, 1325, 1003, 949, 826, 792, 736; Ms (ESI): *m*/*z* = 423.1 $[M+H]^+$.

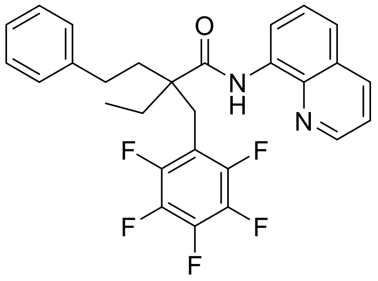

3p

Compound **3p**, pale yellow oil, yield: 84%, R_f = 0.51 (hexane/EtOAc 5:1). ^{1}H NMR (500 MHz, $CDCl_3$) δ 10.14 (brs, 1H), 8.76 – 8.73 (m, 2H), 8.17 (dd, J = 8.3, 1.6 Hz, 1H), 7.58 – 7.44 (m, 3H), 7.31 – 7.24 (m, 4H), 7.21 – 7.17 (m, 1H), 3.15 (dd, J = 32.0, 14.1 Hz, 2H), 2.79 – 2.66 (m, 2H), 2.19 – 2.12 (m, 1H), 2.08 – 1.97 (m, 2H), 1.95 – 1.87 (m, 1H), 1.14 (t, J = 7.4 Hz, 3H); ^{13}C NMR (125 MHz, $CDCl_3$) δ 172.9, 148.3, 146.0 (dm, J = 245.4 Hz), 141.9, 140.1 (dm, J = 251.4 Hz), 138.8, 137.5 (dm, J = 246.0 Hz), 136.5, 134.3, 128.6, 128.5, 128.1, 127.7, 126.2, 121.8, 121.7, 116.8, 111.4 (m), 51.7, 36.8, 30.9, 29.5, 27.1, 8.7; ^{19}F NMR (471 MHz, $CDCl_3$) δ -139.2 (dd, J = 22.6, 7.6 Hz, 2F), -156.2 (t, J = 20.9 Hz, 1F), -162.8 (m, 2F); IR (neat) ν 3357, 2942, 1682, 1521, 1503, 1424, 1385, 1325, 1124, 987, 826, 792, 757, 738, 699; Ms (ESI): m/z = 499.2 $[M+H]^+$.

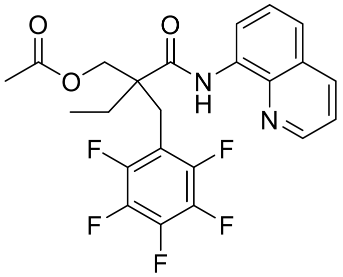

3q

Compound **3q**, white solid, yield: 71%, R_f = 0.44 (hexane/EtOAc 3:1). ^{1}H NMR (500 MHz, $CDCl_3$) δ 10.41 (brs, 1H), 8.76 – 8.73 (m, 2H), 8.18 (dd, J = 8.3, 1.6 Hz, 1H), 7.58 – 7.52 (m, 2H), 7.47 (dd, J = 8.3, 4.2 Hz, 1H), 4.43 (d, J = 11.9 Hz, 1H), 4.33 (d, J = 11.9 Hz, 1H), 3.29 (d, J = 14.2 Hz, 1H), 3.17 (d, J = 14.2 Hz, 1H), 2.23 (s, 3H), 2.05 – 1.96 (m, 1H), 1.80 – 1.72 (m, 1H), 1.02 (t, J = 7.4 Hz, 3H); ^{13}C NMR (125 MHz, $CDCl_3$) δ 171.0, 170.7, 148.3, 146.0 (dm, J = 248.1 Hz), 140.3 (dm, J = 255.9 Hz), 138.9, 137.6 (dm, J = 245.9 Hz), 136.6, 134.4, 128.1, 127.7, 122.0, 121.8, 117.1, 110.7 (m), 65.8, 51.3, 28.1, 26.9, 21.0, 8.9; ^{19}F NMR (471 MHz, $CDCl_3$) δ -139.5 (dd, J = 22.4, 7.6 Hz, 2F), -155.6 (t, J = 20.9 Hz, 1F), -162.4 (m, 2F); IR (neat)

ν 3327, 2924, 2361, 1750, 1683, 1522, 1503, 1382, 1227, 1123, 998, 969, 826, 792, 748; Ms (ESI): *m*/*z* = 467.1 $[M+H]^+$.

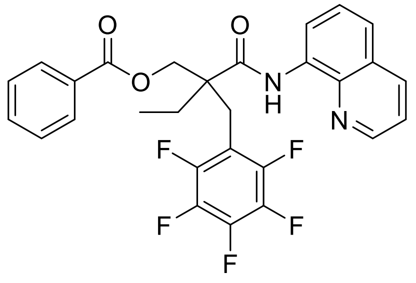

3r

Compound **3r**, white solid, yield: 55%, R_f = 0.47 (hexane/EtOAc 3:1). ^1H NMR (500 MHz, $CDCl_3$) δ 10.47 (brs, 1H), 8.76 (dd, *J* = 7.3, 1.6 Hz, 1H), 8.36 (dd, *J* = 4.2, 1.7 Hz, 1H), 8.15 – 8.09 (m, 3H), 7.60 – 7.51 (m, 3H), 7.45 – 7.41 (m, 2H), 7.37 (dd, *J* = 8.3, 4.2 Hz, 1H), 4.71 (d, *J* = 12.0 Hz, 1H), 4.64 (d, *J* = 12.0 Hz, 1H), 3.38 (d, *J* = 14.2 Hz, 1H), 3.29 (d, *J* = 14.2 Hz, 1H), 2.15 – 2.07 (m, 1H), 1.94 – 1.85 (m, 1H), 1.08 (t, *J* = 7.4 Hz, 3H); ^{13}C NMR (125 MHz, $CDCl_3$) δ 171.0, 166.3, 148.2, 146.0 (dm, *J* = 244.6 Hz), 140.5 (dm, *J* = 252.8 Hz), 138.8, 137.6 (dm, *J* = 244.4 Hz), 136.4, 134.3, 133.4, 130.0, 129.8, 128.6, 128.0, 127.6, 122.0, 121.7, 117.0, 110.7 (m), 66.2, 51.7, 28.5, 27.2, 9.0; ^{19}F NMR (471 MHz, $CDCl_3$) δ -139.4 (dd, *J* = 22.5, 7.7 Hz, 2F), -155.6 (t, *J* = 20.9 Hz, 1F), -162.3 (m, 2F); IR (neat) ν 3342, 2925, 2361, 1723, 1683, 1522, 1504, 1266, 1122, 984, 913, 826, 792, 743, 711; Ms (ESI): *m*/*z* = 529.2 $[M+H]^+$.

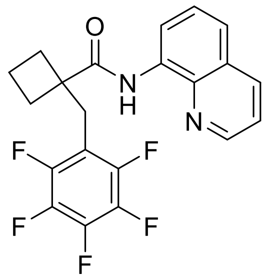

3s

Compound **3s**, colorless oil, yield: 80%, R_f = 0.48 (hexane/EtOAc 5:1). ^1H NMR (500 MHz, $CDCl_3$) δ 9.75 (brs, 1H), 8.72 – 8.69 (m, 2H), 8.16 (dd, *J* = 8.3, 1.6 Hz,

1H), 7.57 – 7.49 (m, 2H), 7.44 (dd, *J* = 8.3, 4.2 Hz, 1H), 3.31 (s, 2H), 2.67 – 2.60 (m, 2H), 2.29 – 2.22 (m, 2H), 2.11 – 2.02 (m, 1H), 2.00 – 1.91 (m, 1H); ^{13}C NMR (125 MHz, $CDCl_3$) δ 174.6, 148.2, 145.6 (dm, *J* = 247.0 Hz), 140.1 (dm, *J* = 251.0 Hz), 138.6, 137.5 (dm, *J* = 250.9 Hz), 136.5, 134.2, 128.0, 127.6, 121.7 (2C), 116.6, 111.4 (m), 50.7, 31.2, 30.2, 15.2; ^{19}F NMR (471 MHz, $CDCl_3$) δ -140.9 (dd, *J* = 22.7, 8.0 Hz, 2F), -156.3 (t, *J* = 20.9 Hz, 1F), -162.6 (m, 2F); IR (neat) ν 3349, 2925, 2853, 1684, 1521, 1502, 1486, 1424, 1324, 1123, 986, 826, 792, 734; Ms (ESI): *m*/*z* = 407.1 $[M+H]^+$.

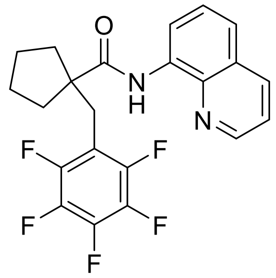

3t

Compound **3t**, colorless oil, yield: 91%, R_f = 0.52 (hexane/EtOAc 5:1). ^{1}H NMR (500 MHz, $CDCl_3$) δ 9.98 (brs, 1H), 8.73 – 8.69 (m, 2H), 8.15 (dd, *J* = 8.3, 1.6 Hz, 1H), 7.56 – 7.42 (m, 3H), 3.16 (s, 2H), 2.40 – 2.28 (m, 2H), 1.90 – 1.77 (m, 6H); ^{13}C NMR (125 MHz, $CDCl_3$) δ 174.0, 148.2, 145.7 (dm, *J* = 245.0 Hz), 140.1 (dm, *J* = 250.9 Hz), 138.6, 137.5 (dm, *J* = 251.1 Hz), 136.5, 134.4, 128.0, 127.6, 121.7, 121.6, 116.6, 111.9 (m), 57.5, 35.0, 31.1, 23.6; ^{19}F NMR (471 MHz, $CDCl_3$) δ -140.4 (dd, *J* = 22.7, 7.9 Hz, 2F), -156.6 (t, *J* = 20.9 Hz, 1F), -162.9 (m, 2F); IR (neat) ν 3354, 3050, 2961, 2878, 1682, 1521, 1424, 1385, 1324, 1123, 1022, 964, 826, 792, 757, 737; Ms (ESI): *m*/*z* = 421.1 $[M+H]^+$.

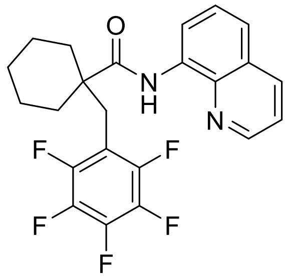

3u

Compound **3u**, colorless oil, yield: 62%, R_f = 0.53 (hexane/EtOAc 5:1). ^{1}H NMR (500 MHz, $CDCl_3$) δ 10.12 (brs, 1H), 8.75 – 8.71 (m, 2H), 8.17 (dd, *J* = 8.3, 1.6 Hz,

1H), 7.58 – 7.50 (m, 2H), 7.45 (dd, *J* = 8.3, 4.2 Hz, 1H), 2.99 (s, 2H), 2.42 – 2.32 (m, 2H), 1.80 – 1.72 (m, 2H), 1.70 – 1.63 (m, 1H), 1.54 – 1.45 (m, 4H), 1.34 – 1.27 (m, 1H); ^{13}C NMR (125 MHz, $CDCl_3$) δ 172.6, 148.3, 145.8 (dm, *J* = 237.4 Hz), 140.0 (dm, *J* = 250.9 Hz), 138.8, 137.4 (dm, *J* = 244.0 Hz), 136.5, 134.4, 128.0, 127.7, 121.7, 121.6, 116.8, 111.1 (m), 49.8, 38.9, 34.4, 25.8, 23.5; ^{19}F NMR (471 MHz, $CDCl_3$) δ -139.6 (dd, *J* = 22.7, 7.8 Hz, 2F), -156.6 (t, *J* = 20.9 Hz, 1F), -163.1 (m, 2F); IR (neat) ν 3358, 2932, 2861, 1727, 1683, 1521, 1503, 1423, 1386, 1324, 1124, 976, 945, 826, 791, 756; Ms (ESI): *m*/*z* = 435.1 $[M+H]^+$.

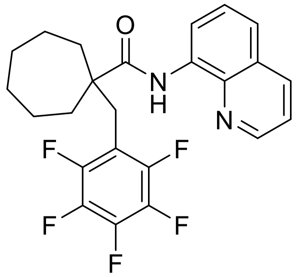

3v

Compound **3v**, colorless oil, yield: 83%, R_f = 0.53 (hexane/EtOAc 5:1). ^{1}H NMR (500 MHz, $CDCl_3$) δ 10.00 (brs, 1H), 8.75 – 8.70 (m, 2H), 8.16 (dd, *J* = 8.3, 1.6 Hz, 1H), 7.57 – 7.42 (m, 3H), 3.03 (s, 2H), 2.41 – 2.35 (m, 2H), 1.80 – 1.71 (m, 4H), 1.70 – 1.57 (m, 6H); ^{13}C NMR (125 MHz, $CDCl_3$) δ 173.9, 148.2, 145.8 (dm, *J* = 247.3 Hz), 140.1 (dm, *J* = 250.8 Hz), 138.8, 137.4 (dm, *J* = 250.5 Hz), 136.5, 134.5, 128.0, 127.7, 121.7, 121.5, 116.7, 111.5 (m), 52.2, 35.9, 34.5, 29.7, 23.6; ^{19}F NMR (471 MHz, $CDCl_3$) δ -139.7 (dd, *J* = 22.7, 7.8 Hz, 2F), -156.6 (t, *J* = 20.9 Hz, 1F), -163.1 (m, 2F); IR (neat) ν 3357, 2929, 2858, 1683, 1521, 1502, 1385, 1326, 1124, 989, 925, 826, 792, 736; Ms (ESI): *m*/*z* = 449.2 $[M+H]^+$.

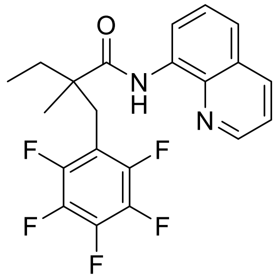

3w1

Compound **3w1**, white solid, yield: 73%, R_f = 0.50 (hexane/EtOAc 5:1). ^{1}H NMR (500 MHz, $CDCl_3$) δ 10.16 (brs, 1H), 8.78 – 8.75 (m, 2H), 8.17 (dd, *J* = 8.3, 1.6 Hz,

1H), 7.57 – 7.43 (m, 2H), 7.45 (dd, J = 8.3, 4.2 Hz, 1H), 3.16 – 3.08 (m, 2H), 2.14 – 2.06 (m, 1H), 1.60 – 1.52 (m, 1H), 1.42 (s, 3H), 1.00 (t, J = 7.4 Hz, 3H); ^{13}C NMR (125 MHz, $CDCl_3$) δ 173.7, 148.4, 145.8 (dm, J = 244.8 Hz), 140.1 (dm, J = 251.0 Hz), 138.9, 137.5 (dm, J = 252.4 Hz), 136.5, 134.4, 128.1, 127.6, 121.8, 121.7, 116.6, 111.6 (m), 48.8, 32.8, 31.8, 20.4, 9.4; ^{19}F NMR (471 MHz, $CDCl_3$) δ -139.3 (dd, J = 22.6, 7.8 Hz, 2F), -156.4 (t, J = 20.9 Hz, 1F), -162.8 (m, 2F); IR (neat) v 3360, 2971, 1683, 1521, 1502, 1424, 1385, 1324, 1123, 981, 826, 791, 756; Ms (ESI): m/z = 409.1 $[M+H]^+$.

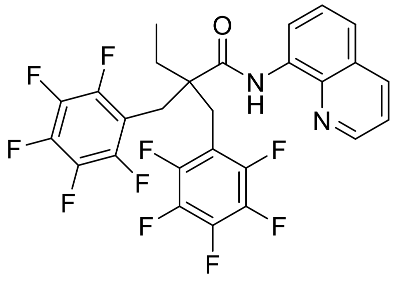

3w2

Compound **3w2**, white solid, yield: 12%, R_f = 0.54 (hexane/EtOAc 5:1). ^{1}H NMR (500 MHz, $CDCl_3$) δ 9.58 (brs, 1H), 8.73 (dd, J = 7.5, 1.4 Hz, 1H), 8.53 (dd, J = 4.2, 1.6 Hz, 1H), 8.16 (dd, J = 8.3, 1.6 Hz, 1H), 7.59 – 7.451 (m, 2H), 7.43 (dd, J = 8.3, 4.2 Hz, 1H), 3.28 (d, J = 14.2 Hz, 2H), 3.19 (d, J = 14.2 Hz, 2H), 1.78 (q, J = 7.2 Hz, 2H), 1.16 (t, J = 7.3 Hz, 3H); ^{13}C NMR (125 MHz, $CDCl_3$) δ 170.8, 148.0, 146.1 (dm, J = 248.0 Hz), 140.4 (dm, J = 251.9 Hz), 138.4, 137.7 (dm, J = 250.9 Hz), 136.6, 133.7, 128.0, 127.7, 122.0, 121.8, 116.7, 110.9 (m), 52.9, 28.7, 25.9, 9.0; ^{19}F NMR (471 MHz, $CDCl_3$) δ -138.3 (dd, J = 22.4, 7.3 Hz, 4F), -155.4 (t, J = 20.9 Hz, 2F), -162.4 (m, 4F); IR (neat) v 3346, 3052, 2981, 2946, 1683, 1505, 1424, 1386, 1325, 1122, 1096, 1009, 890, 826, 792, 739; Ms (ESI): m/z = 575.1 $[M+H]^+$.

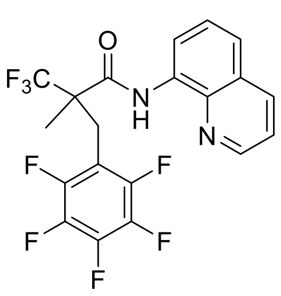

3x1

Compound **3x1**, colorless oil, yield: 54%, R_f = 0.55 (hexane/EtOAc 5:1). ^{1}H NMR (500 MHz, $CDCl_3$) δ 10.58 (brs, 1H), 8.79 (dd, J = 4.2, 1.7 Hz, 1H), 8.73 – 8.68 (m, 1H), 8.19 (dd, J = 8.3, 1.7 Hz, 1H), 7.59 – 7.54 (m, 2H), 7.48 (dd, J = 8.3, 4.2 Hz, 1H), 3.52 (d, J = 14.5 Hz, 1H), 3.39 (d, J = 14.5 Hz, 1H), 1.60 (s, 3H); ^{13}C NMR (125 MHz, $CDCl_3$) δ 165.3, 148.6, 146.0 (dm, J = 246.5 Hz), 140.8 (dm, J = 252.9 Hz), 138.8, 137.6 (dm, J = 251.5 Hz), 136.5, 133.9, 128.0, 127.5, 126.8 (q, J = 281.9 Hz), 122.6, 121.9, 117.1, 109.1 (m), 53.6 (q, J = 24.3 Hz), 26.7, 16.4; ^{19}F NMR (471 MHz, $CDCl_3$) δ -71.9 (s, 3F), -139.1 (m, 2F), -154.4 (t, J = 20.9 Hz, 1F), -161.9 (m, 2F); IR (neat) ν 3344, 1695, 1522, 1505, 1328, 1266, 1183, 1099, 992, 976, 826, 791, 756; Ms (ESI): m/z = 449.1 $[M+H]^+$.

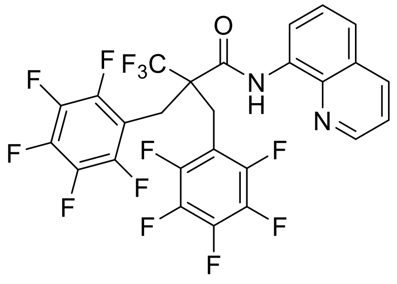

3x2

Compound **3x2**, colorless oil, yield: 36%, R_f = 0.57 (hexane/EtOAc 5:1). ^{1}H NMR (500 MHz, $CDCl_3$) δ 10.34 (brs, 1H), 8.73 – 8.68 (m, 1H), 8.62 (dd, J = 4.2, 1.6 Hz, 1H), 8.18 (dd, J = 8.3, 1.6 Hz, 1H), 7.60 – 7.56 (m, 2H), 7.45 (dd, J = 8.3, 4.2 Hz, 1H), 3.60 – 3.52 (m, 4H); ^{13}C NMR (125 MHz, $CDCl_3$) δ 163.2, 148.4, 146.2 (dm, J = 250.1 Hz), 141.0 (dm, J = 253.5 Hz), 138.7, 137.7 (dm, J = 250.4 Hz), 136.5, 133.6, 128.0, 127.5, 126.4 (q, J = 282.5 Hz), 122.9, 122.0, 117.6, 109.1 (m), 57.1 (q, J = 22.6 Hz), 26.5; ^{19}F NMR (471 MHz, $CDCl_3$) δ -66.5 (s, 3F), -138.1 (dd, J = 14.9, 5.8 Hz, 4F), -154.2 (t, J = 20.9 Hz, 2F), -162.2 (m, 4F); IR (neat) ν 3336, 2929, 1694, 1506, 1426, 1388, 1267, 1180, 1068, 955, 827, 792, 741; Ms (ESI): m/z = 615.1 $[M+H]^+$.

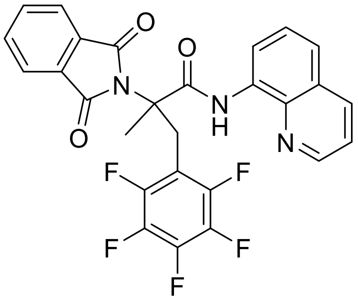

3y

Compound **3y**, white solid, yield: 67%, R_f = 0.38 (hexane/EtOAc 2:1). ^{1}H NMR (500 MHz, $CDCl_3$) δ 10.23 (brs, 1H), 8.72 (dd, J = 7.6, 1.1 Hz, 1H), 8.43 (dd, J = 4.2, 1.6 Hz, 1H), 8.11 (dd, J = 8.3, 1.5 Hz, 1H), 7.84 – 7.79 (m, 2H), 7.78 – 7.74 (m, 2H), 7.59 – 7.49 (m, 2H), 7.34 (dd, J = 8.2, 4.2 Hz, 1H), 3.92 (d, J = 14.5 Hz, 1H), 3.76 (d, J = 14.4 Hz, 1H), 2.13 (s, 3H); ^{13}C NMR (125 MHz, $CDCl_3$) δ 170.0, 168.9, 148.3, 146.1 (dm, J = 246.9 Hz), 140.5 (dm, J = 252.6 Hz), 138.7, 137.6 (dm, J = 251.0 Hz), 136.5, 134.6, 134.0, 131.6, 128.0, 127.5, 123.6, 122.0, 121.7, 116.8, 110.2 (m), 65.2, 28.3, 22.5; ^{19}F NMR (471 MHz, $CDCl_3$) δ -140.6 (dd, J = 21.8, 6.4 Hz, 2F), -154.4 (t, J = 20.9 Hz, 1F), -161.7 (m, 2F); IR (neat) ν 3338, 2924, 1716, 1696, 1522, 1502, 1321, 1124, 987, 909, 825, 792, 721; Ms (ESI): m/z = 526.1 $[M+H]^+$.

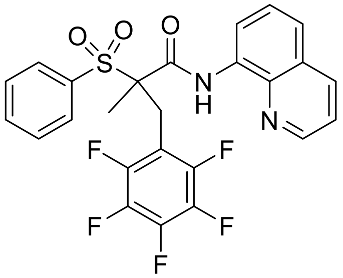

3z

Compound **3z**, yellow solid, yield: 76%, R_f = 0.49 (hexane/EtOAc 3:1). ^{1}H NMR (500 MHz, $CDCl_3$) δ 11.64 (brs, 1H), 8.98 (dd, J = 4.2, 1.6 Hz, 1H), 8.53 (dd, J = 7.6, 1.1 Hz, 1H), 8.22 (dd, J = 8.3, 1.6 Hz, 1H), 7.98 (dd, J = 8.4, 1.1 Hz, 2H), 7.64 – 7.58 (m, 2H), 7.55 – 7.50 (m, 2H), 7.47 – 7.43 (m, 2H), 3.97 (d, J = 14.2 Hz, 1H), 3.74 (d, J = 14.2 Hz, 1H), 1.59 (s, 3H); ^{13}C NMR (125 MHz, $CDCl_3$) δ 163.8, 149.1, 145.9 (dm, J = 251.4 Hz), 140.9 (dm, J = 253.1 Hz), 137.7 (dm, J = 251.9 Hz), 139.3, 136.4, 135.1, 134.8, 134.2, 130.7, 129.4, 128.2, 127.3, 122.8, 122.0, 117.4, 108.5 (m), 72.4, 26.6, 17.1; ^{19}F NMR (471 MHz, $CDCl_3$) δ -138.4 (dd, J = 21.8, 6.7 Hz, 2F), -153.8 (t, J = 20.9 Hz, 1F), -161.4 (m, 2F); IR (neat) ν 3273, 2964, 1684, 1522, 1505, 1325, 1308, 1146, 991, 912, 827, 792, 738; Ms (ESI): m/z = 521.1 $[M+H]^+$.

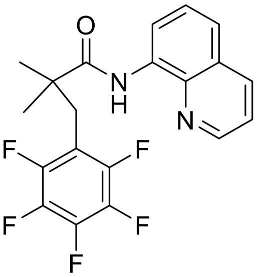

3aa1

Compound **3aa1**, white solid, yield: 62%, $R_f = 0.47$ (hexane/EtOAc 5:1). ^{1}H NMR (500 MHz, $CDCl_3$) δ 10.23 (brs, 1H), 8.78 – 8.74 (m, 2H), 8.17 (dd, J = 8.3, 1.5 Hz, 1H), 7.57 – 7.50 (m, 2H), 7.46 (dd, J = 8.2, 4.2 Hz, 1H), 3.14 (s, 2H), 1.45 (s, 6H); ^{13}C NMR (125 MHz, $CDCl_3$) δ 174.6, 148.4, 145.8 (dm, J = 242.9 Hz), 140.1 (dm, J = 251.1 Hz), 138.9, 137.6 (dm, J = 244.3 Hz), 136.5, 134.4, 128.1, 127.6, 121.8, 121.7, 116.6, 111.6 (m), 44.9, 33.1, 24.8; ^{19}F NMR (471 MHz, $CDCl_3$) δ -139.6 (dd, J = 22.6, 7.7 Hz, 2F), -156.2 (t, J = 20.8 Hz, 1F), -162.7 (m, 2F); IR (neat) ν 3360, 2971, 1683, 1521, 1504, 1424, 1385, 1325, 1123, 977, 917, 826, 792, 733; Ms (ESI): m/z = 395.1 $[M+H]^+$.

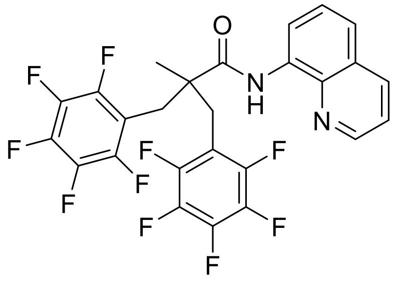

3aa2

Compound **3aa2**, colorless oil, yield: 30%, $R_f = 0.51$ (hexane/EtOAc 5:1). ^{1}H NMR (500 MHz, $CDCl_3$) δ 9.95 (brs, 1H), 8.73 (dd, J = 7.2, 1.7 Hz, 1H), 8.65 (dd, J = 4.2, 1.6 Hz, 1H), 8.17 (dd, J = 8.3, 1.6 Hz, 1H), 7.59 – 7.53 (m, 2H), 7.44 (dd, J = 8.3, 4.2 Hz, 1H), 3.36 (d, J = 14.0 Hz, 2H), 3.17 (d, J = 14.0 Hz, 2H), 1.42 (s, 3H); ^{13}C NMR (125 MHz, $CDCl_3$) δ 171.8, 148.3, 145.8 (dm, J = 248.1 Hz), 140.4 (dm, J = 251.6 Hz), 138.7, 137.7 (dm, J = 249.9 Hz), 136.6, 133.8, 128.0, 127.6, 122.2, 121.8, 117.0, 110.9 (m), 49.0, 31.9, 19.6; ^{19}F NMR (471 MHz, $CDCl_3$) δ -138.8 (dd, J = 22.4, 7.5 Hz, 4F), -155.2 (t, J = 20.9 Hz, 2F), -162.0 (m, 4F); IR (neat) ν 3355, 2976, 1684, 1522, 1503, 1424, 1386, 1325, 1121, 985, 944, 826, 792, 736; Ms (ESI): m/z = 561.1 $[M+H]^+$.

Deuterium Labeling Experiment

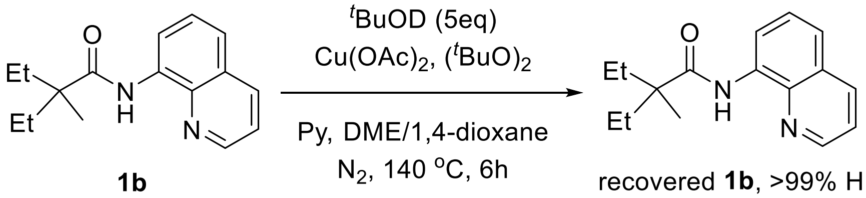

A 50 mL Schlenk tube was charged with amide **1b** (76.8 mg, 0.30 mmol), $Cu(OAc)_2$ (54.5 mg, 0.30 mmol), DME/1,4-dioxane (7:3, v/v, 1.0 mL), pyridine (72 μL, 71 mg, 0.90 mmol), *tert*-butyl peroxide (138 μL, 110 mg, 0.75 mmol) and *tert*-butan(ol-*d*) (99% D, 143 μL, 113 mg, 1.5 mmol) in sequence under N_2 atmosphere. The tube was capped with a Teflon screw cap, and stirred at 140 °C for 6h. Then the reaction mixture was cooled to room temperature, diluted with EtOAc (30 mL), filtered through a celite pad, and concentrated under reduced pressure. The residue was purified by flash column chromatography on silica gel, eluting with EtOAc/Hexane (1:30, v/v), to afford recovered **1b** (72.4 mg, 94% yield).

Recovered **1b**: ^{1}H NMR (500 MHz, $CDCl_3$) δ 10.22 (brs, 1H), 8.84 – 8.81 (m, 2H), 8.15 (dd, J = 8.3, 1.7 Hz, 1H), 7.55 – 7.42 (m, 3H), 1.93 – 1.85 (m, 2H), 1.67 – 1.59 (m, 2H), 1.35 (s, **3H**), 0.94 (t, J = 7.5 Hz, 6H).

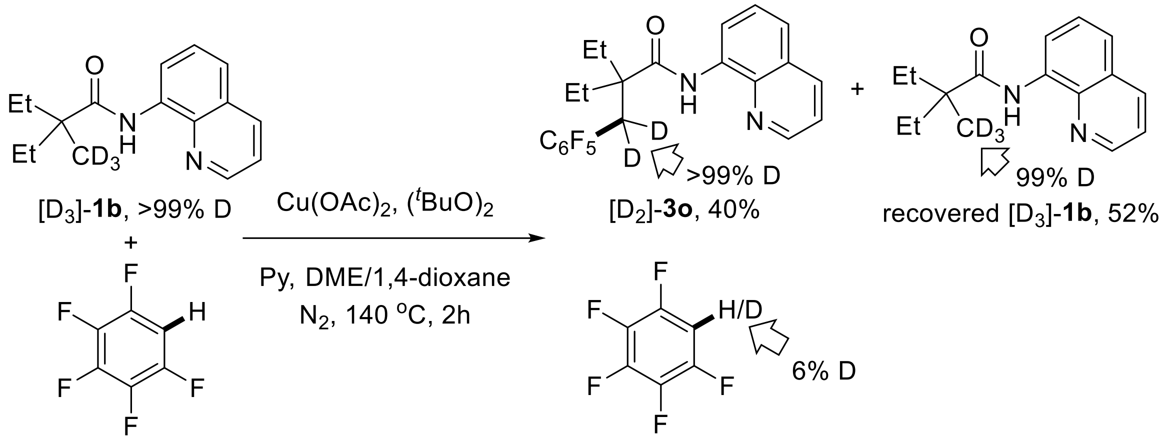

A 50 mL Schlenk tube was charged with [D_3]-**1b** (77.7 mg, 0.30 mmol), $Cu(OAc)_2$ (54.5 mg, 0.30 mmol), DME/1,4-dioxane (7:3, v/v, 1.0 mL), pyridine (72 μL, 71 mg, 0.90 mmol), *tert*-butyl peroxide (138 μL, 110 mg, 0.75 mmol) and pentafluorobenzene (67 μL, 101 mg, 0.60 mmol) in sequence under N_2 atmosphere. The tube was capped with a Teflon screw cap, and stirred at 140 °C for 2h. Then the reaction mixture was cooled to room temperature, and analyzed by GC-MS to obtain the rate of H/D exchange of pentafluorobenzene. The resultant mixture was diluted with EtOAc (30 mL), filtered through a celite pad, and concentrated under reduced pressure. The residue was purified by flash column chromatography on silica gel, eluting with CH_2Cl_2/Hexane (1:2, v/v), to afford [D_2]-**3o** (50.9 mg, 40% yield) and recovered [D_3]-**1b** (40.4 mg, 52% yield).

[D_2]-**3o**: ^{1}H NMR (500 MHz, $CDCl_3$) δ 10.03 (brs, 1H), 8.74 – 8.71 (m, 2H), 8.16 (dd, J = 8.3, 1.6 Hz, 1H), 7.57 – 7.49 (m, 2H), 7.45 (dd, J = 8.3, 4.2 Hz, 1H), 1.97 – 1.89 (m, 2H), 1.85 – 1.77 (m, 2H), 1.04 (t, J = 7.4 Hz, 6H); ^{13}C NMR (125 MHz, $CDCl_3$) δ 173.3, 148.2, 146.0 (dm, J = 244.8 Hz), 140.0 (dm, J = 250.6 Hz), 138.8,

137.4 (dm, J = 242.9 Hz), 136.5, 134.4, 128.0, 127.7, 121.7, 121.6, 116.7, 111.6 (m), 51.8, 26.6, 8.7; ^{19}F NMR (471 MHz, $CDCl_3$) δ -139.4 (dd, J = 22.6, 7.9 Hz, 2F), -156.6 (t, J = 20.9 Hz, 1F), -163.1 (m, 2F); IR (neat) ν 3361, 2971, 1683, 1521, 1496, 1424, 1385, 1325, 1009, 922, 826, 791, 757; Ms (ESI): m/z = 425.2 $[M+H]^+$.

Recovered $[D_3]$-**1b**: ^{1}H NMR (500 MHz, $CDCl_3$) δ 10.22 (brs, 1H), 8.84 – 8.81 (m, 2H), 8.15 (dd, J = 8.3, 1.6 Hz, 1H), 7.55 – 7.41 (m, 3H), 1.93 – 1.85 (m, 2H), 1.67 – 1.59 (m, 2H), 1.32 – 1.28 (m, **0.04H**), 0.94 (t, J = 7.5 Hz, 6H).

Parallel KIE Experiment

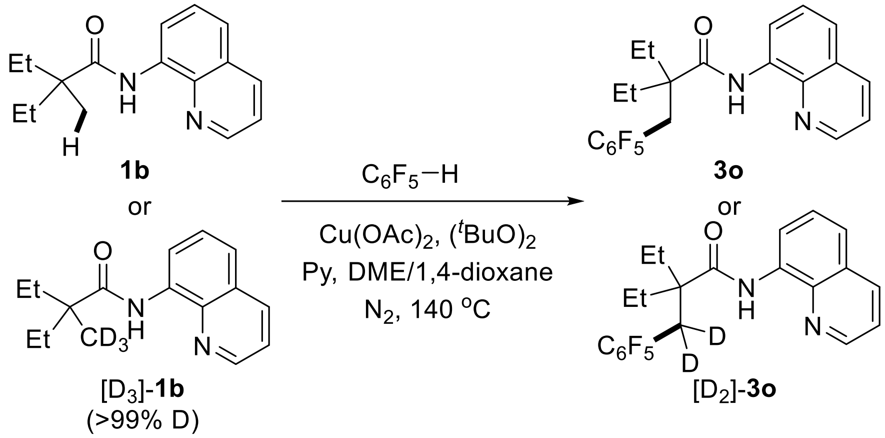

A 50 mL Schlenk tube was charged with **1b** (76.8 mg, 0.30 mmol) or $[D_3]$-**1b** (77.7 mg, 0.30 mmol), $Cu(OAc)_2$ (54.5 mg, 0.30 mmol), DME/1,4-dioxane (7:3, v/v, 1.0 mL), pyridine (72 μL, 71 mg, 0.90 mmol), *tert*-butyl peroxide (138 μL, 110 mg, 0.75 mmol) and pentafluorobenzene (67 μL, 101 mg, 0.60 mmol) in sequence under N_2 atmosphere. The tube was capped with a Teflon screw cap, and stirred at 140 °C for 2h. The reaction was stopped by rapid cooling in the indicated reaction period, and analyzed by GC using benzophenone as the internal standard. The average GC yield was calculated after calibrating the response of GC based on five runs of each reaction. The KIE value determined from two parallel reactions is 1.1.

Time (min)	10	20	30	40	50	60	70	80	90
Yield of **3o** (%)	0.1	0.8	3.3	6.8	8.2	14.8	20.9	28.5	48.0
Yield of $[D_2]$-**3o** (%)	0.1	1.1	2.2	4.2	10.2	12.6	19.5	32.0	38.5

References

1. (a) Tran, L. D.; Daugulis, O. *Angew. Chem. Int. Ed.* **2012**, *51*, 5188. (b) Shang, R.; Ilies, L.; Matsumoto, A.; Nakamura, E. *J. Am. Chem. Soc.* **2013**, *135*, 6030. (c) Wu, X.; Zhao, Y.; Ge, H. *J. Am. Chem. Soc.* **2014**, *136*, 1789. (d) Wu, X.; Zhao, Y.; Zhang, G.; Ge, H. *Angew. Chem. Int. Ed.* **2014**, *53*, 3706. (e) Wu, X.; Zhao, Y.; Ge, H. *Chem. Eur. J.* **2014**, *20*, 9530. (f) Wu, X.; Yang, K.; Zhao, Y.; Sun, H.; Li, G.; Ge, H. *Nat. Commun*. **2015**, *6*, 6462.
2. Wu, X.; Zhao, Y.; Ge, H. *Chem. Asian J*. **2014**, *9*, 2736.

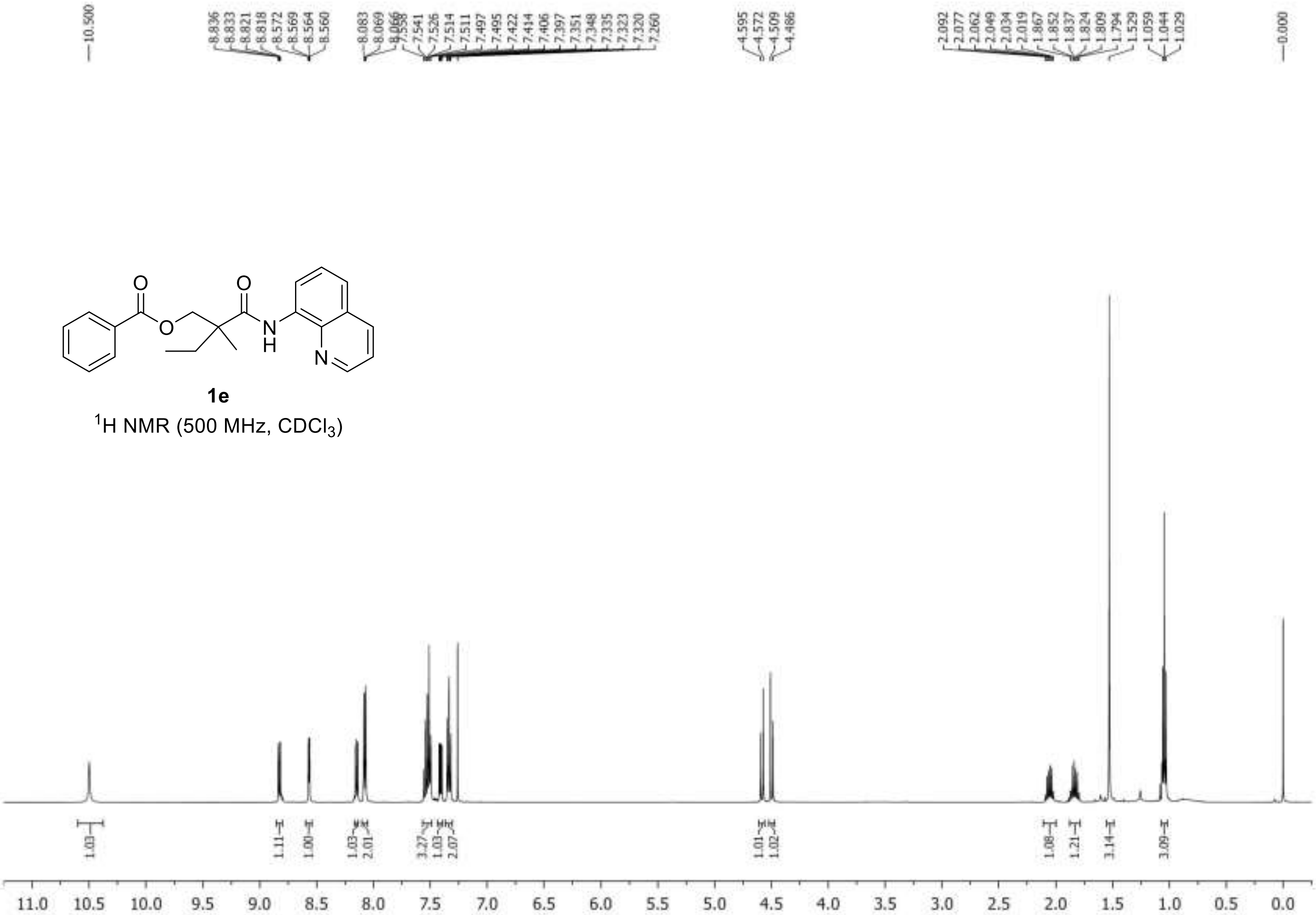

1e

^{1}H NMR (500 MHz, $CDCl_3$)

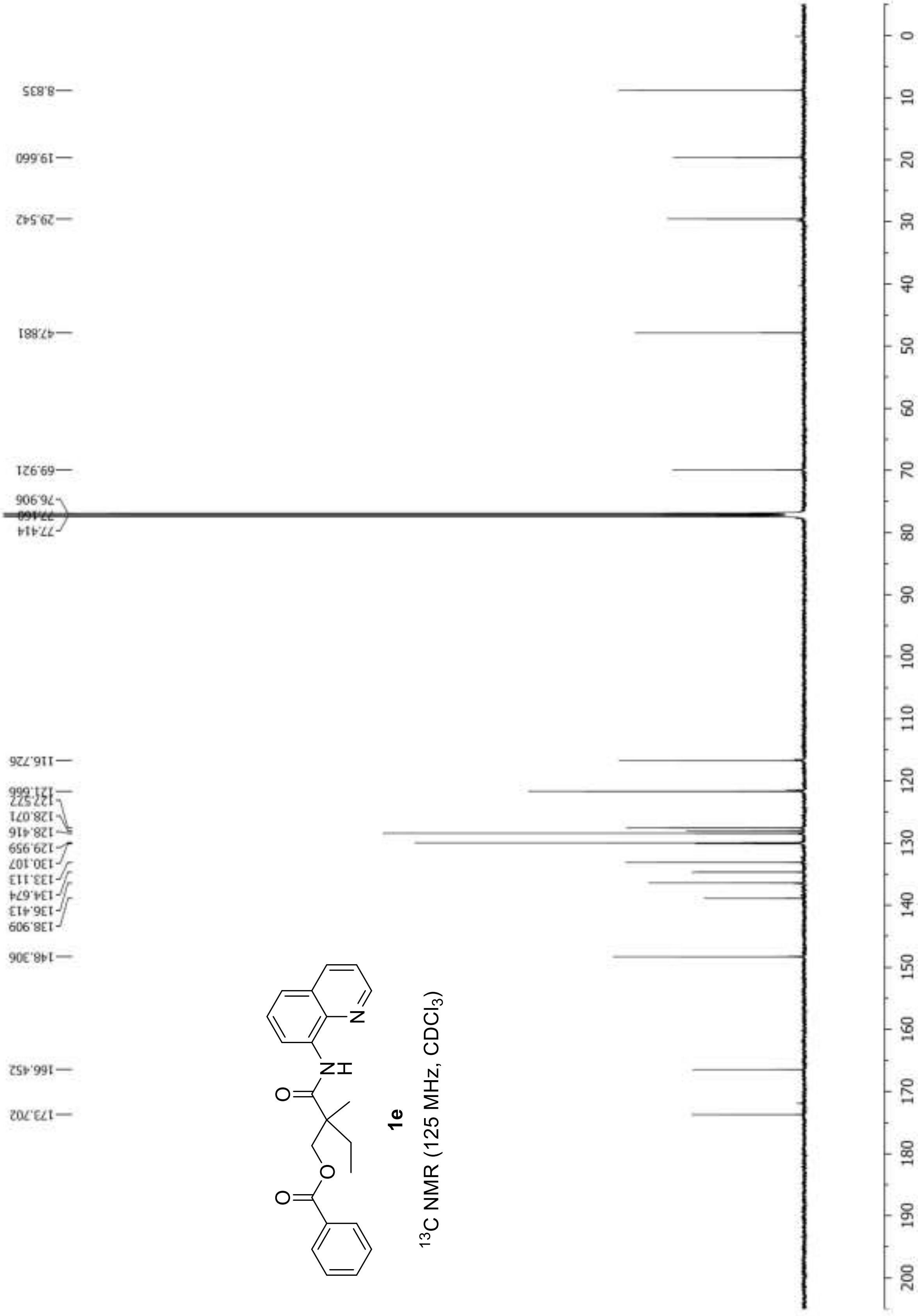

1e

^{13}C NMR (125 MHz, $CDCl_3$)

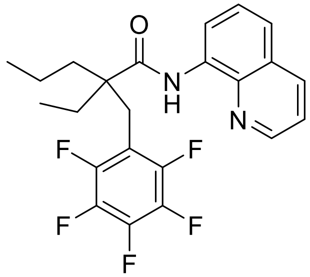

3a

^{1}H NMR (500 MHz, $CDCl_3$)

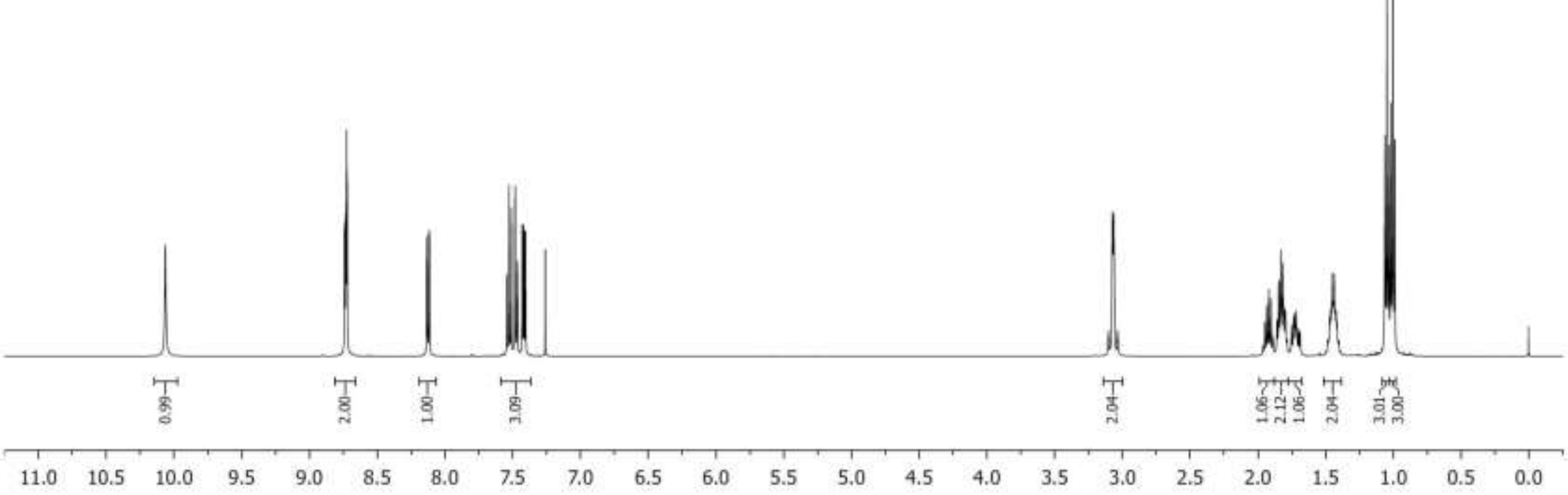

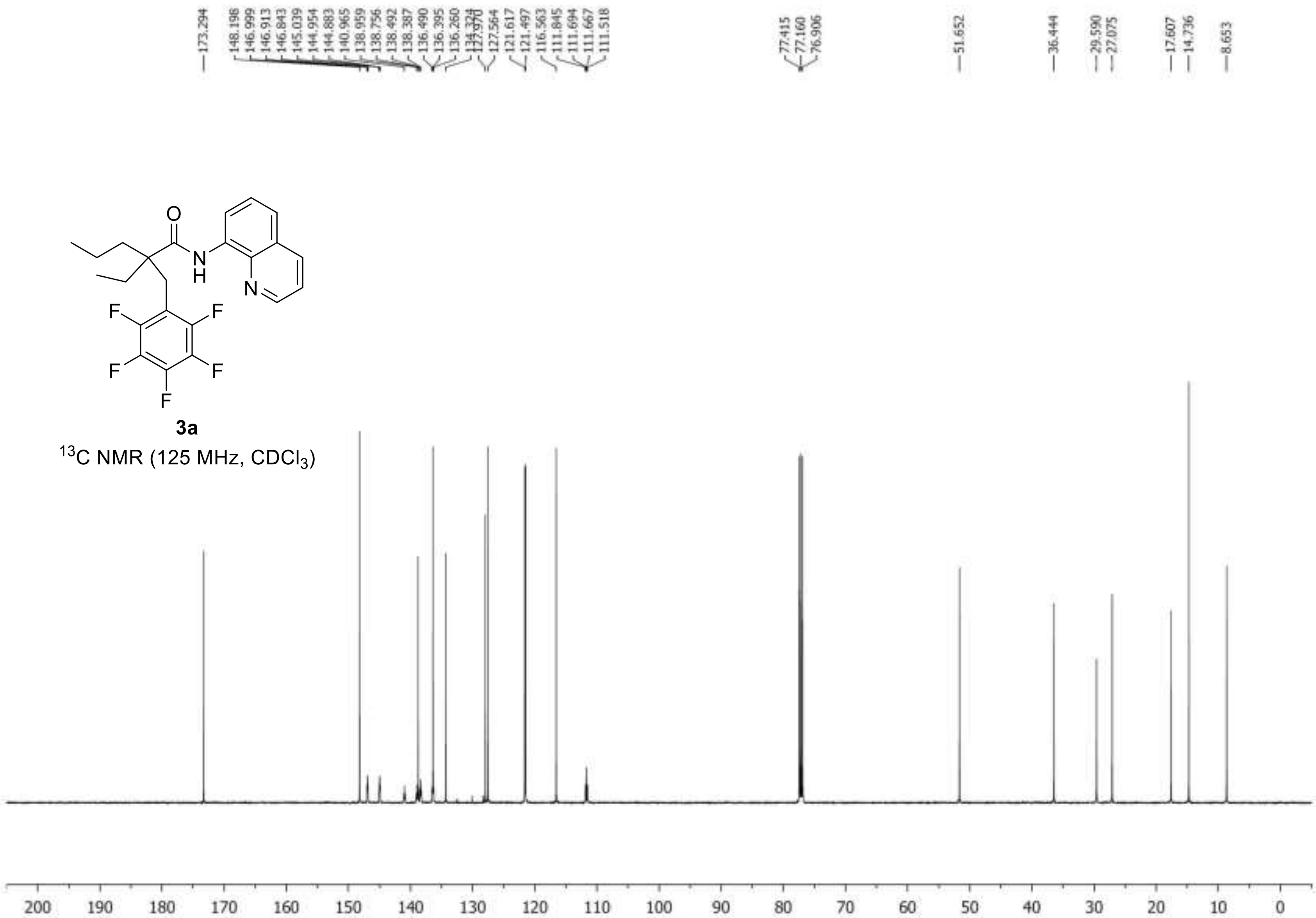

3a

^{13}C NMR (125 MHz, $CDCl_3$)

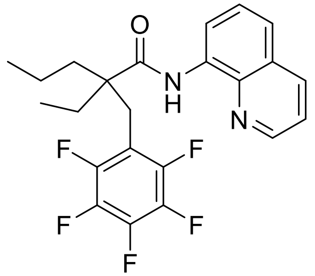

3a

^{19}F NMR (471 MHz, $CDCl_3$)

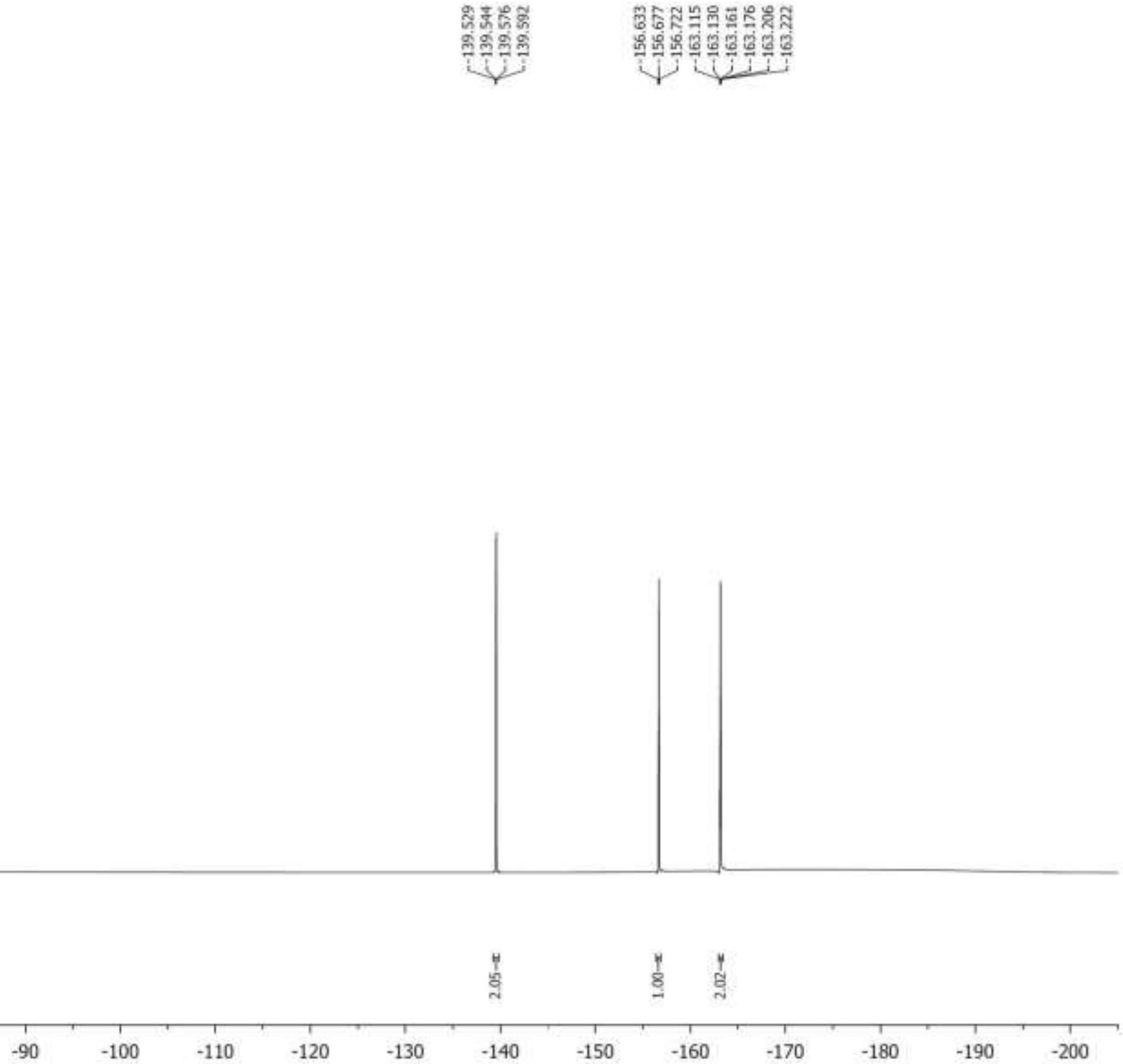

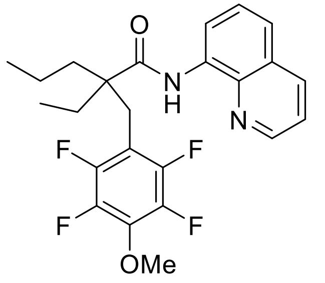

3b

^{1}H NMR (500 MHz, $CDCl_3$)

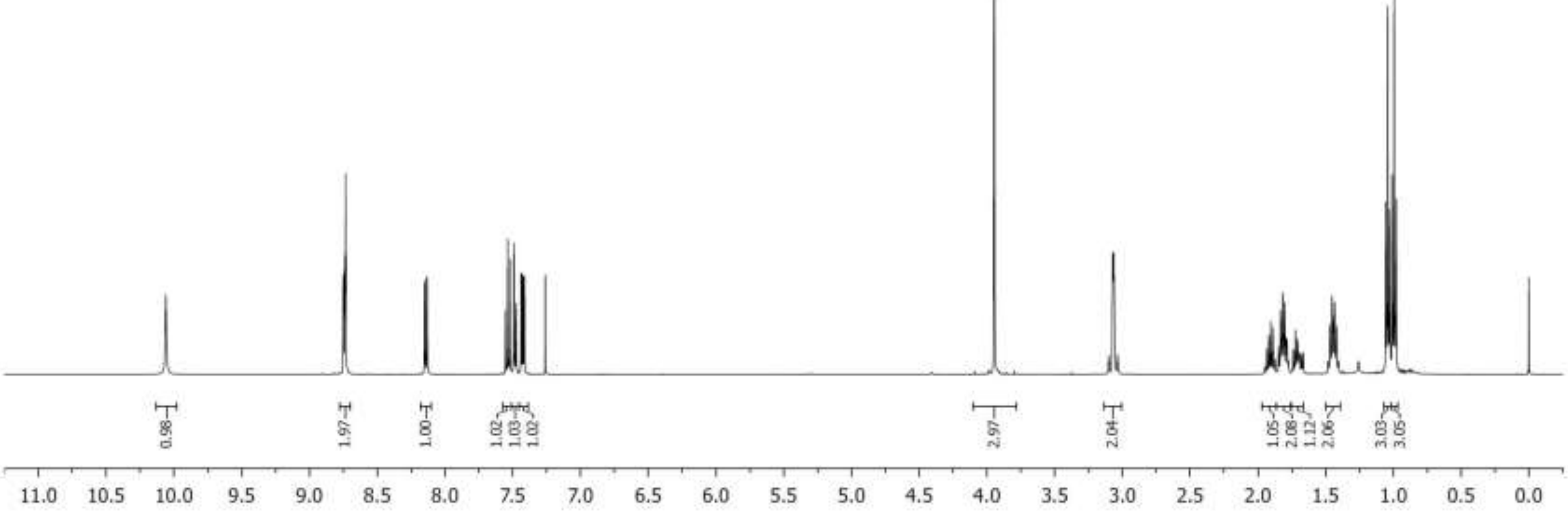

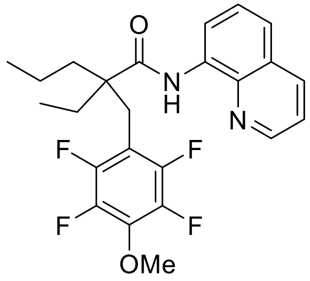

3b

^{13}C NMR (125 MHz, $CDCl_3$)

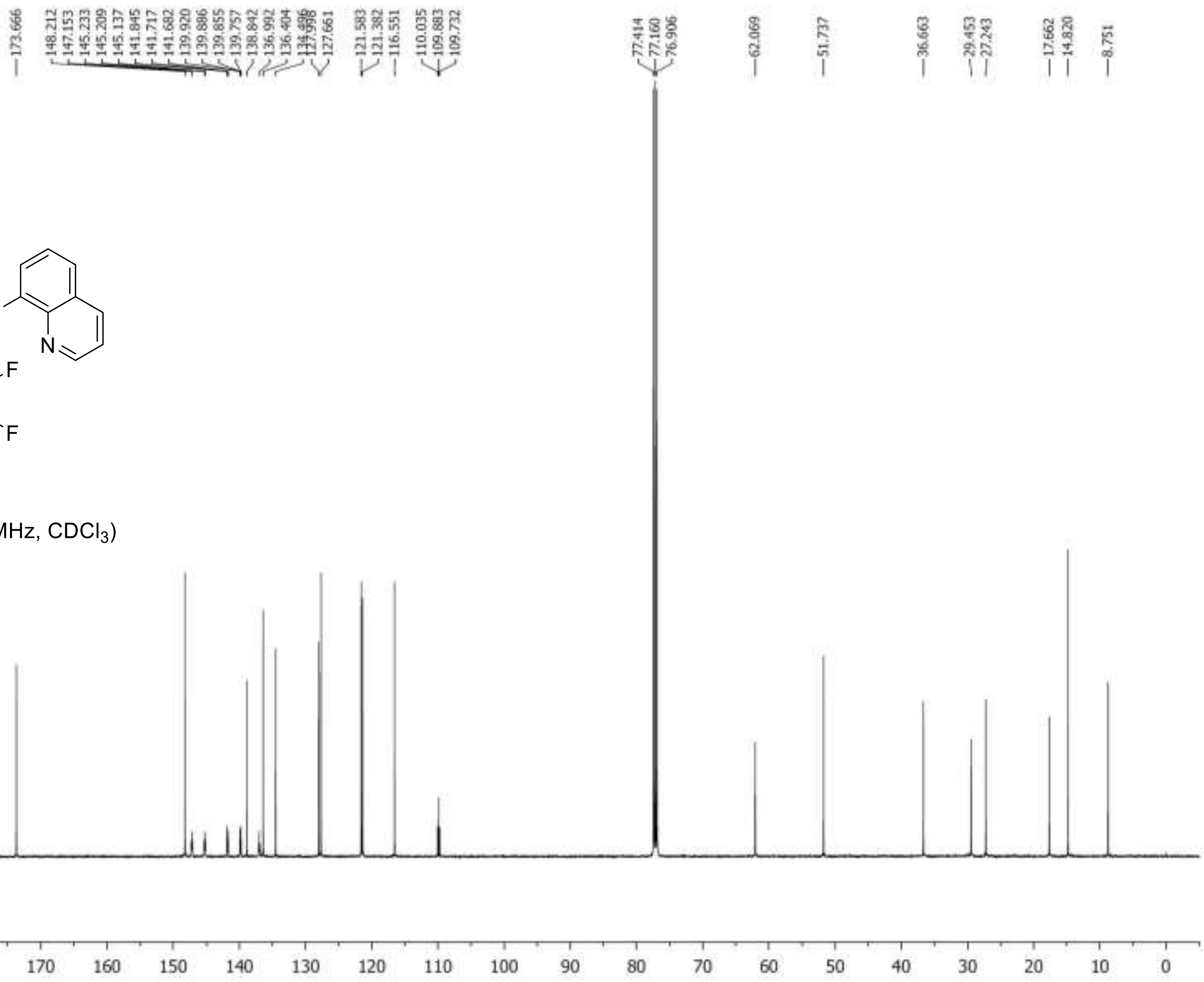

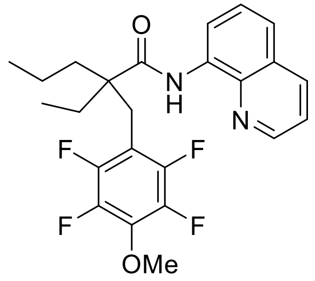

3b

^{19}F NMR (471 MHz, $CDCl_3$)

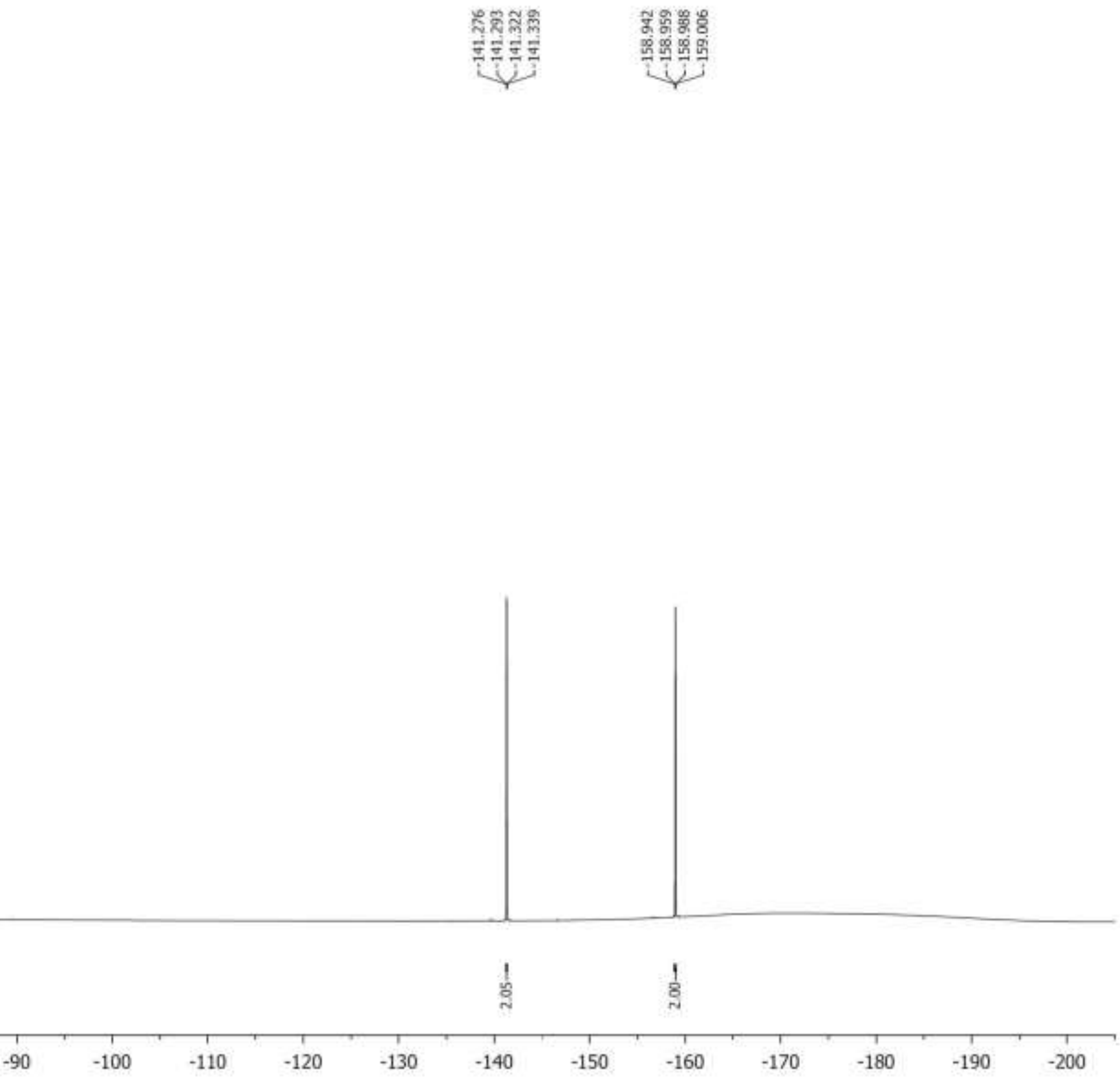

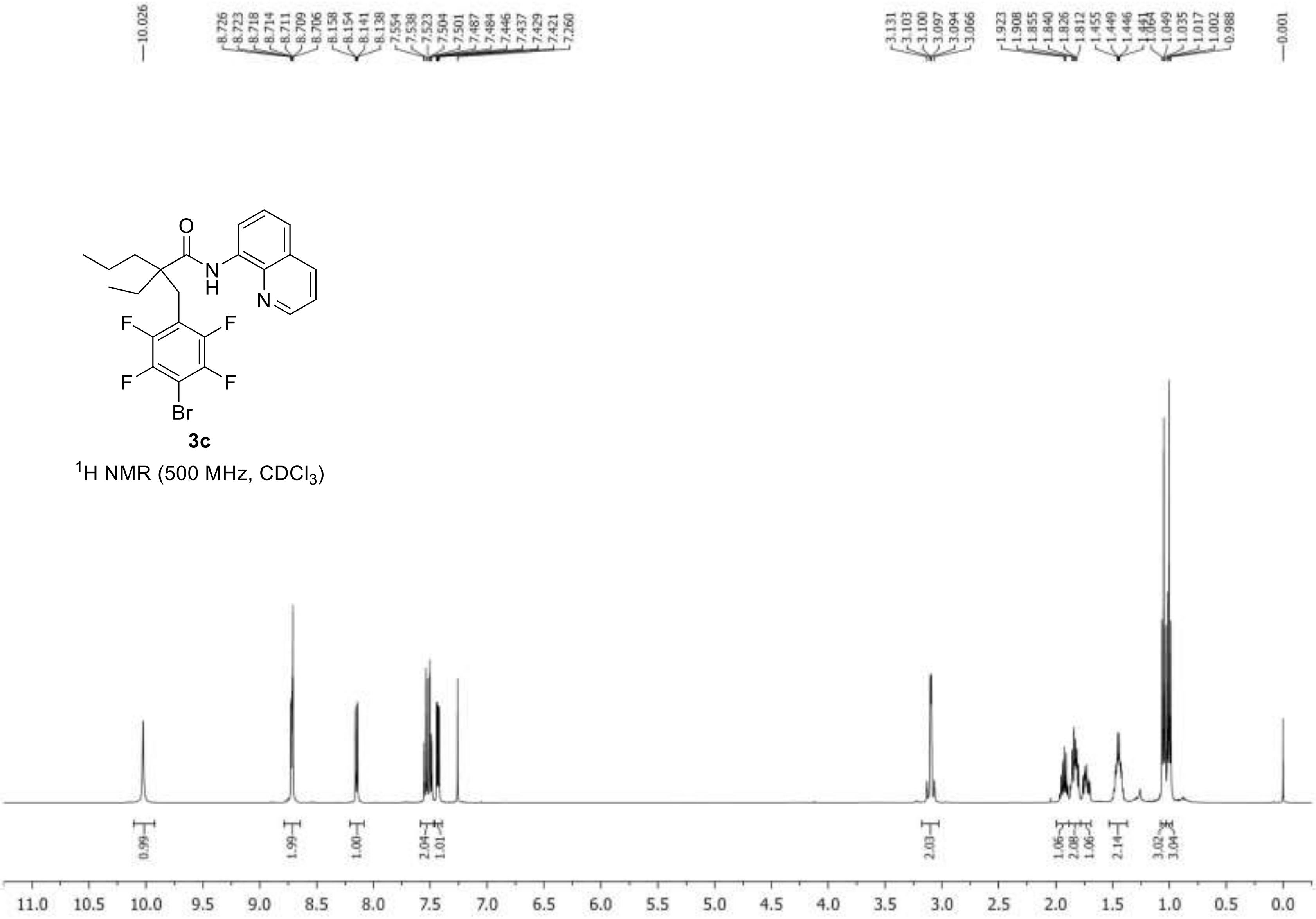

3c

^{1}H NMR (500 MHz, $CDCl_3$)

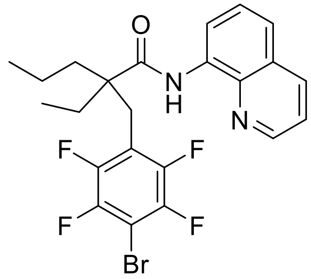

3c

^{13}C NMR (125 MHz, $CDCl_3$)

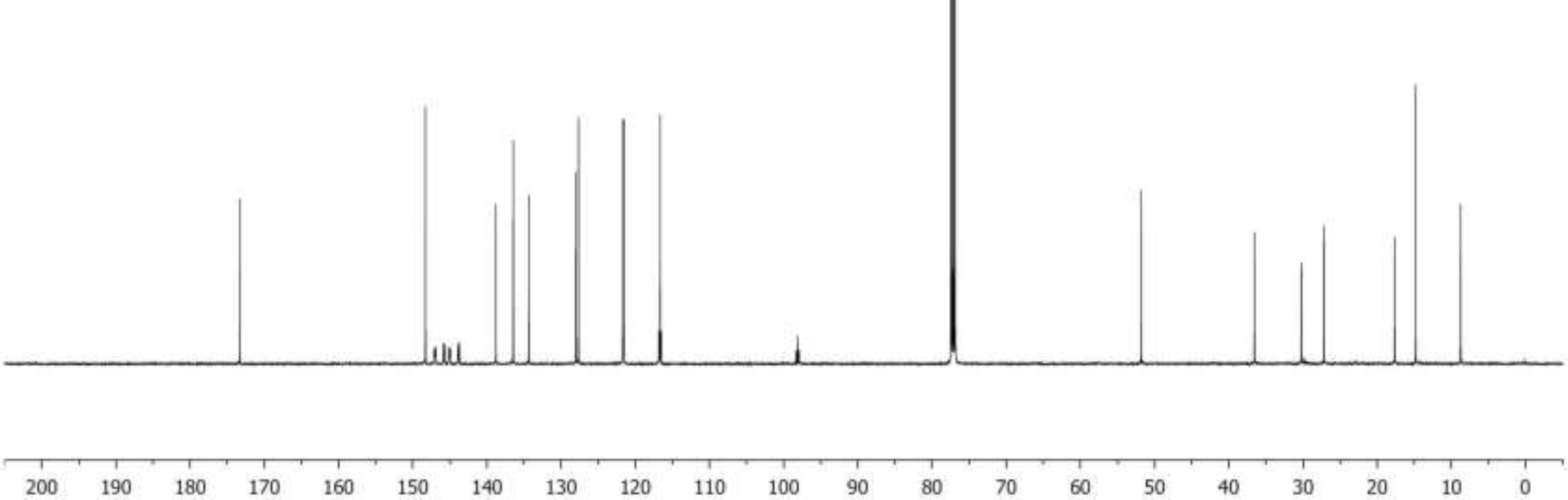

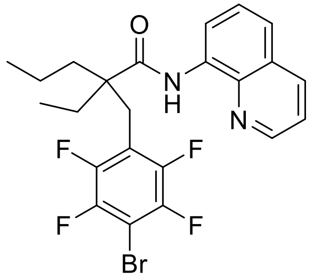

3c

^{19}F NMR (471 MHz, $CDCl_3$)

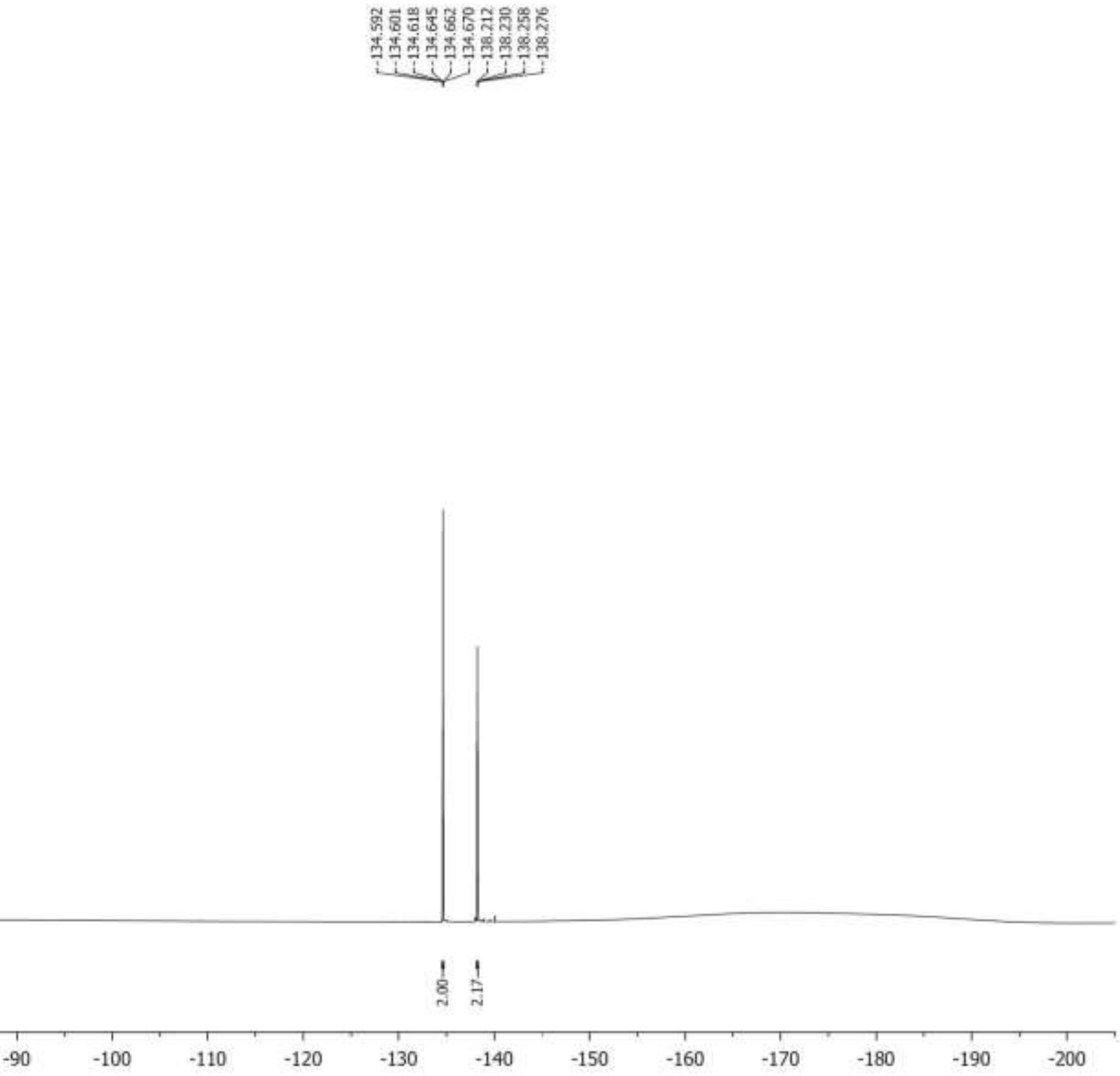

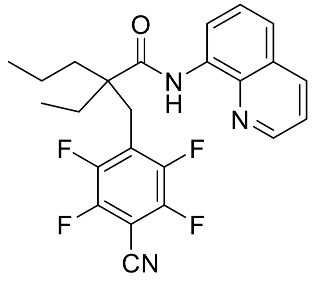

3d

^{1}H NMR (500 MHz, $CDCl_3$)

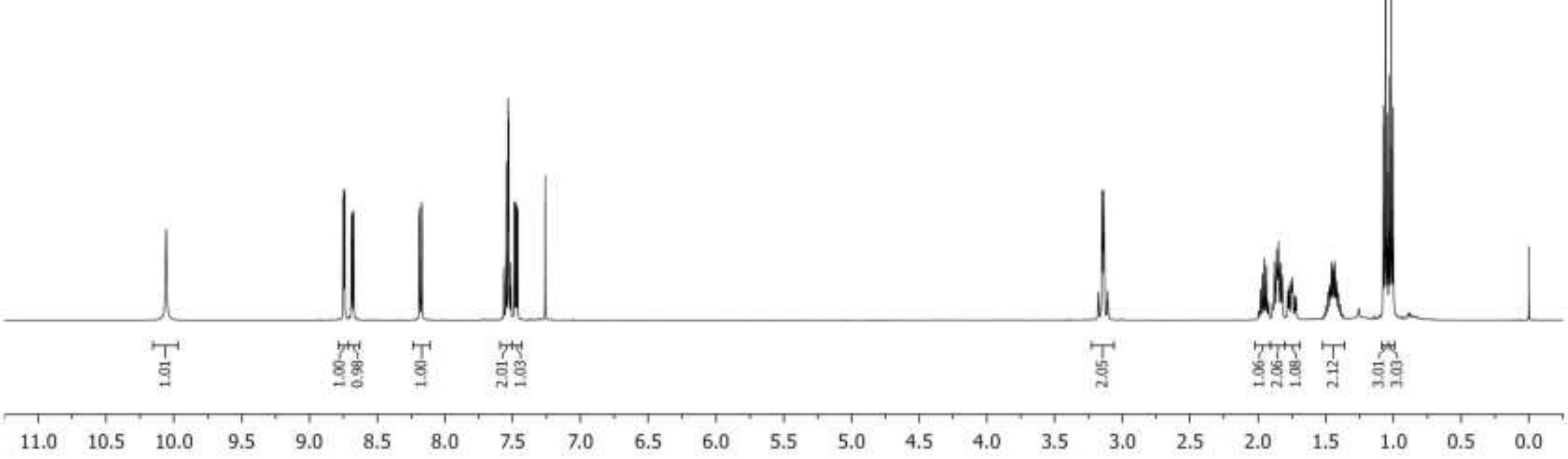

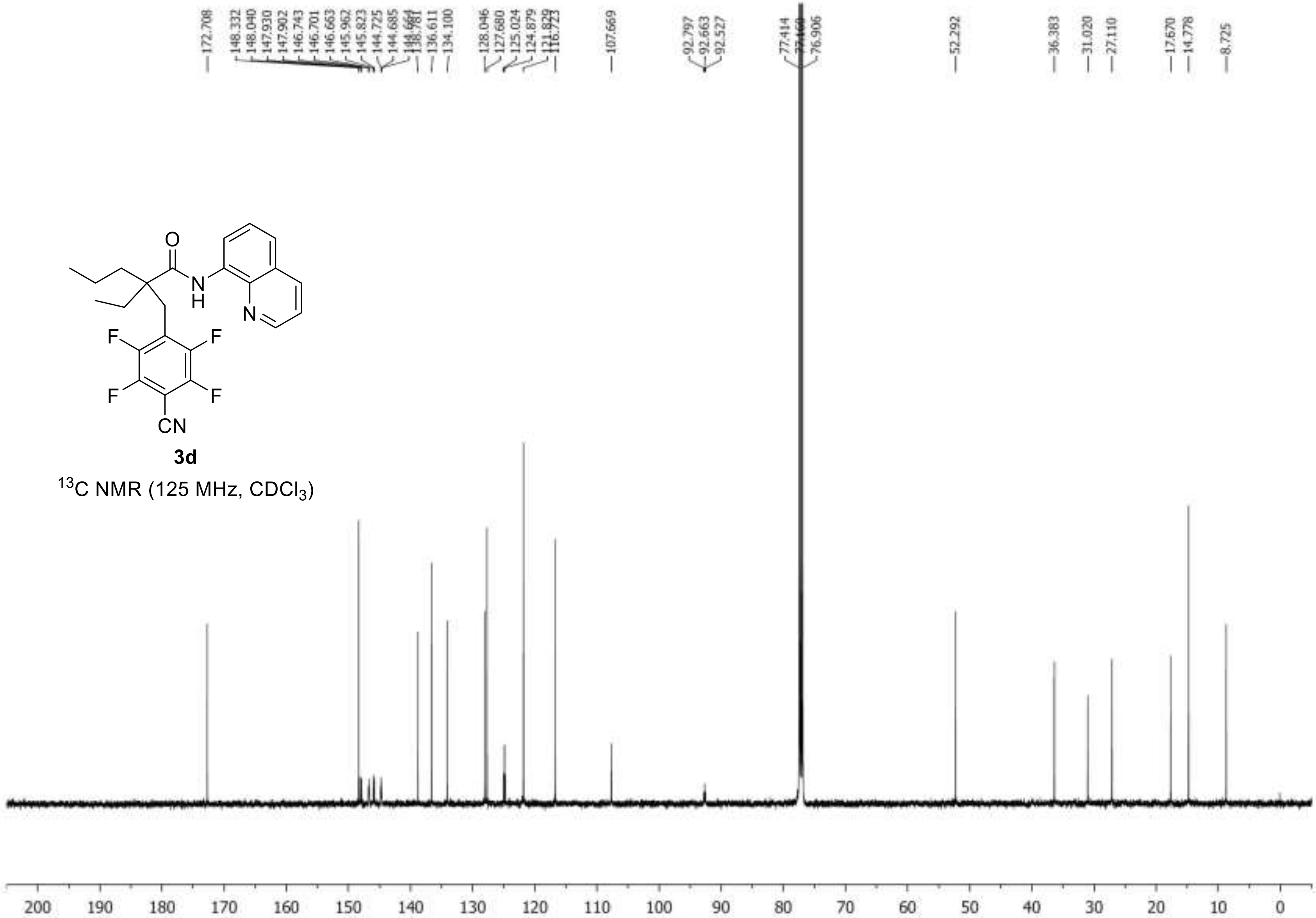

3d

^{13}C NMR (125 MHz, $CDCl_3$)

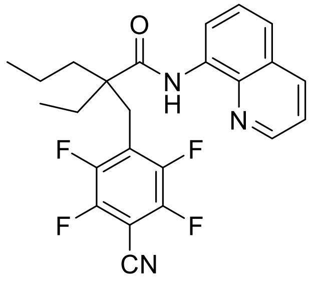

3d

^{19}F NMR (471 MHz, $CDCl_3$)

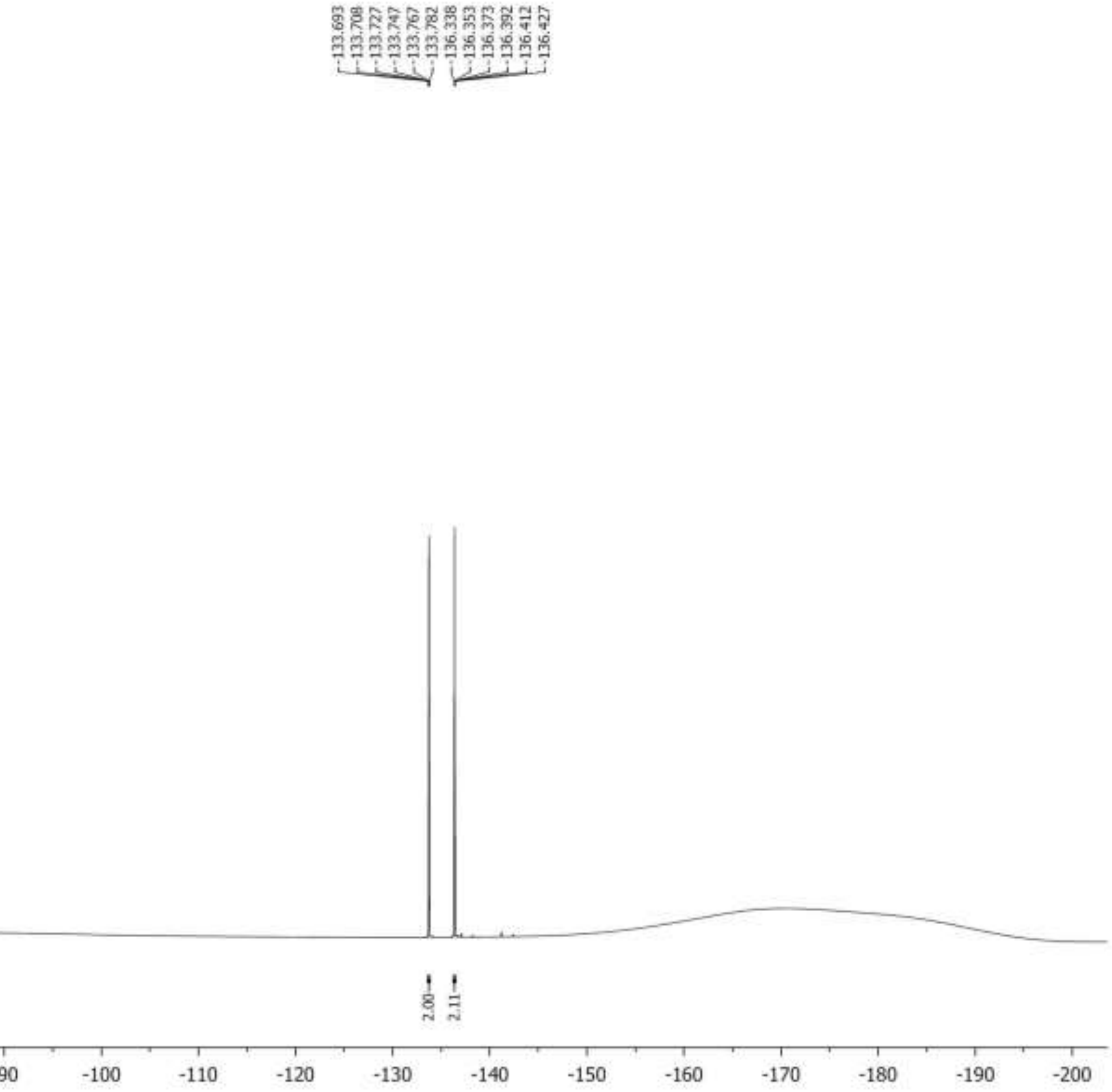

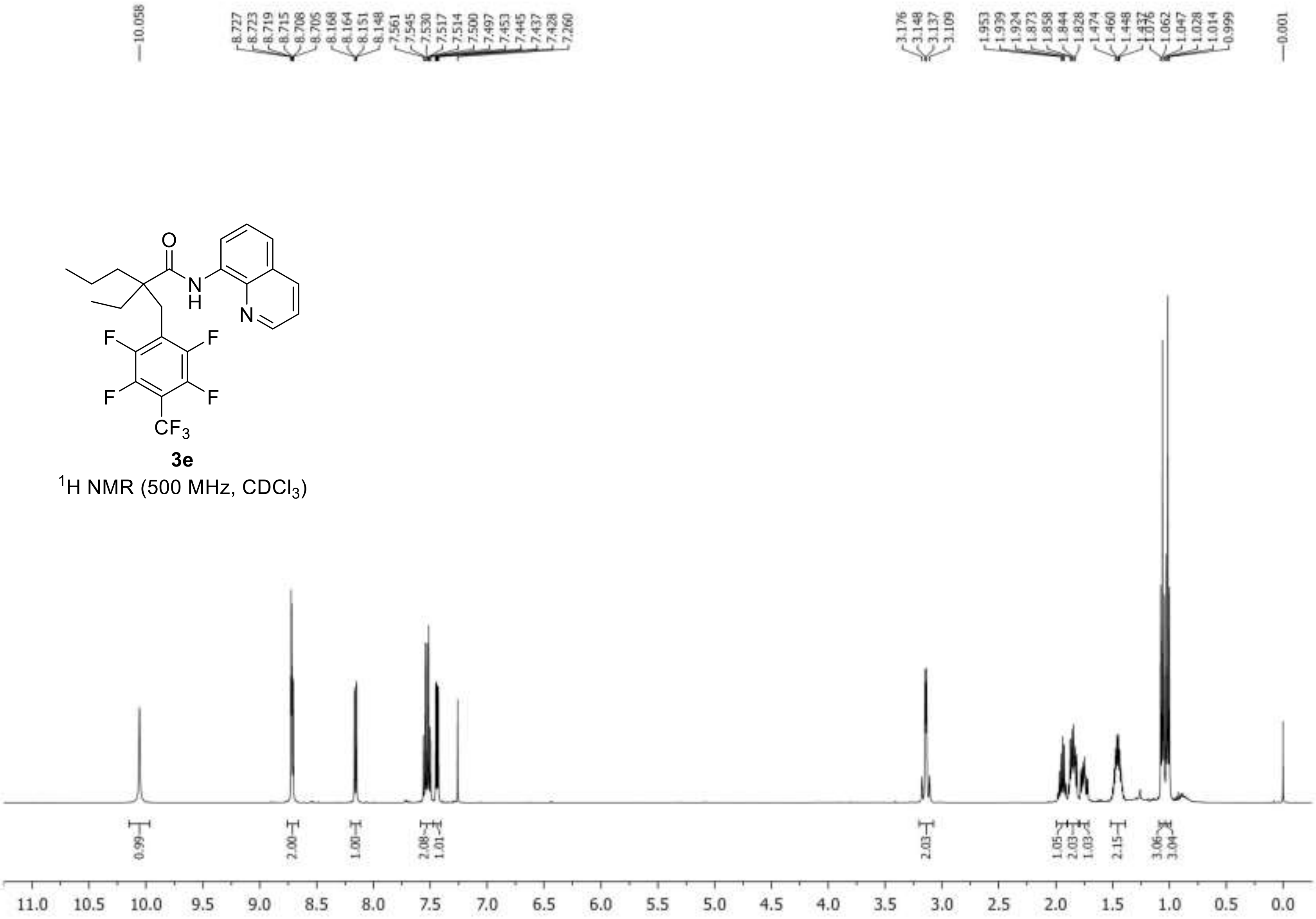

3e

^{1}H NMR (500 MHz, $CDCl_3$)

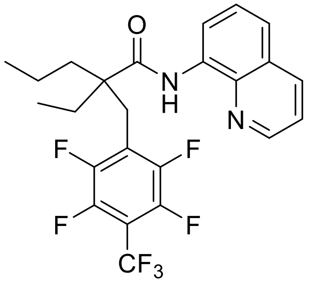

3e

^{13}C NMR (125 MHz, $CDCl_3$)

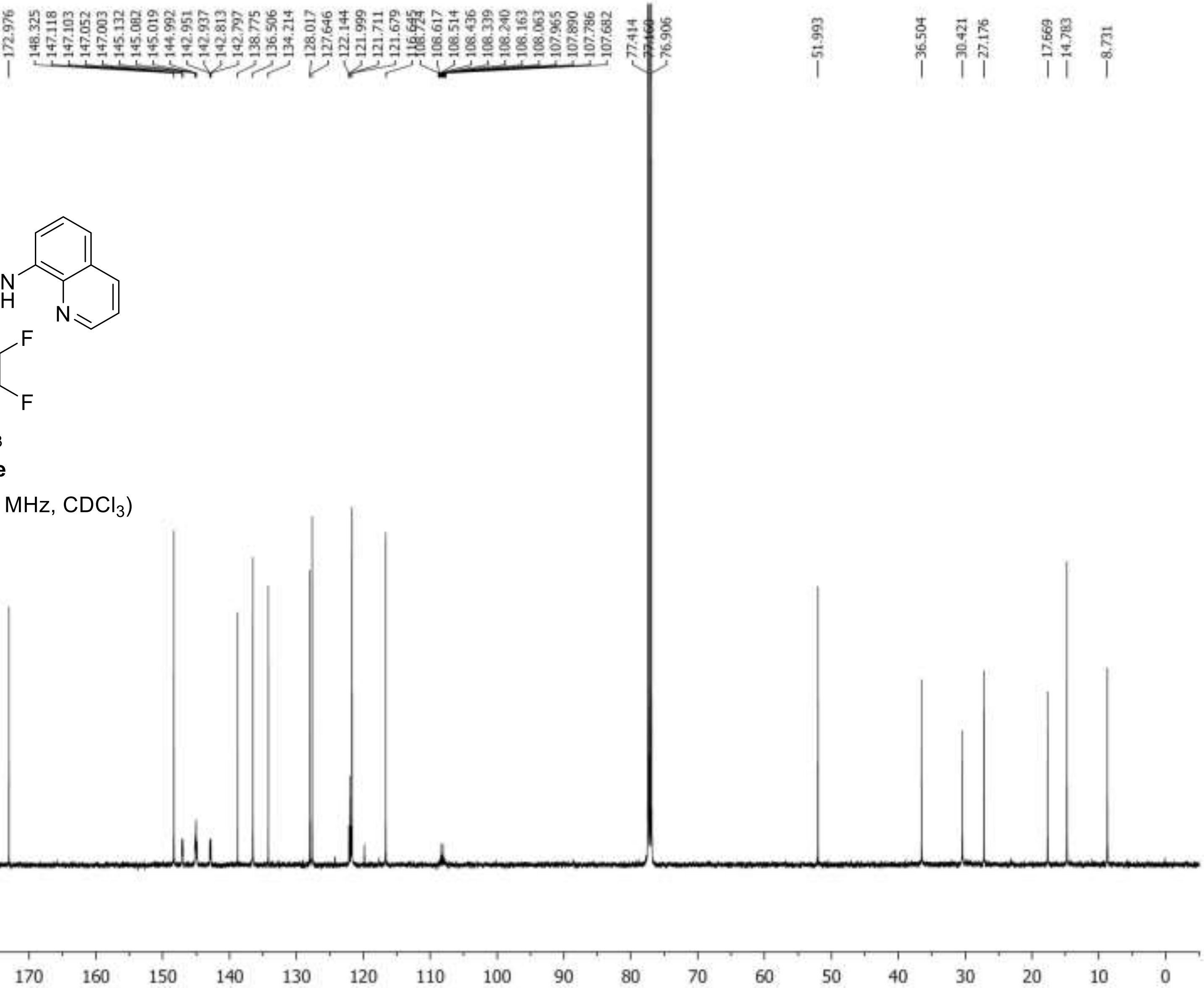

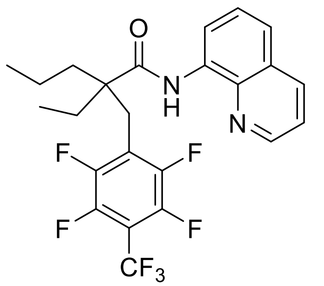

3e

^{19}F NMR (471 MHz, $CDCl_3$)

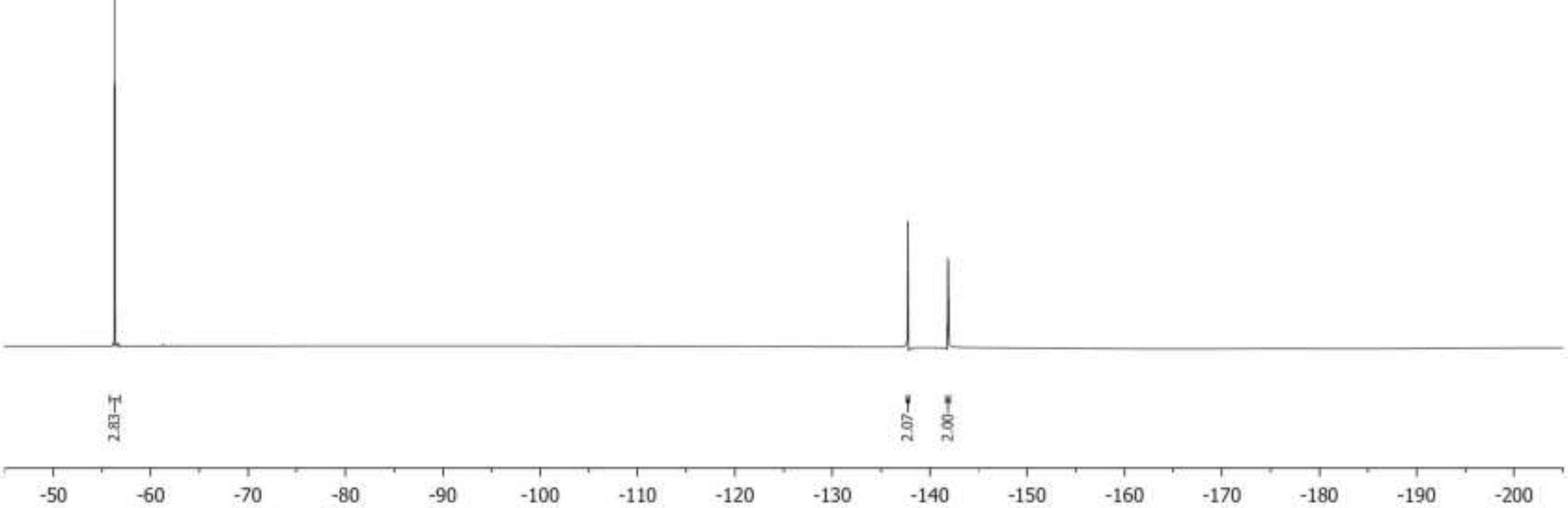

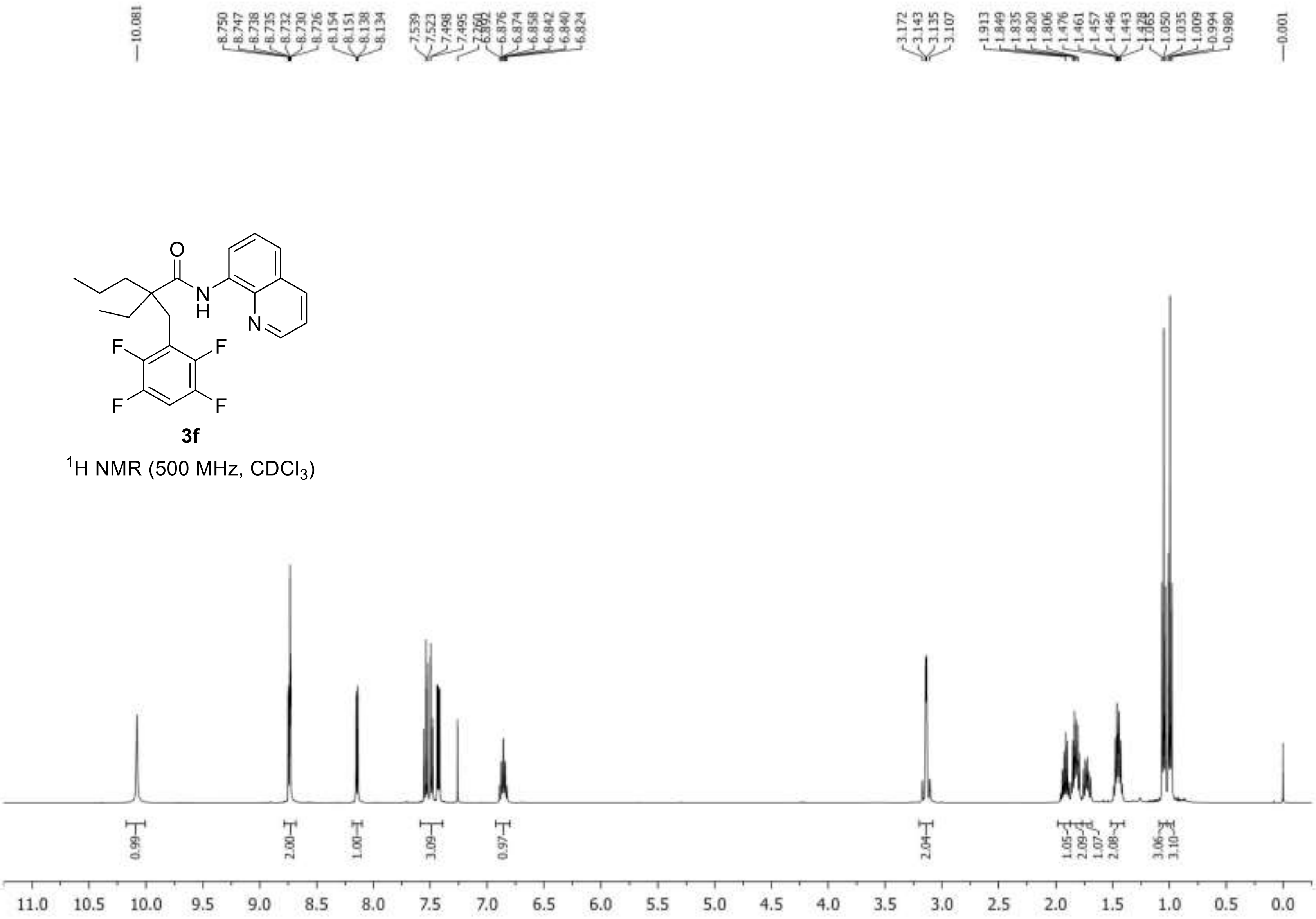

3f

^{1}H NMR (500 MHz, $CDCl_3$)

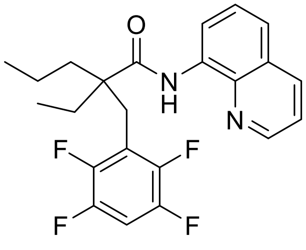

3f

^{13}C NMR (125 MHz, $CDCl_3$)

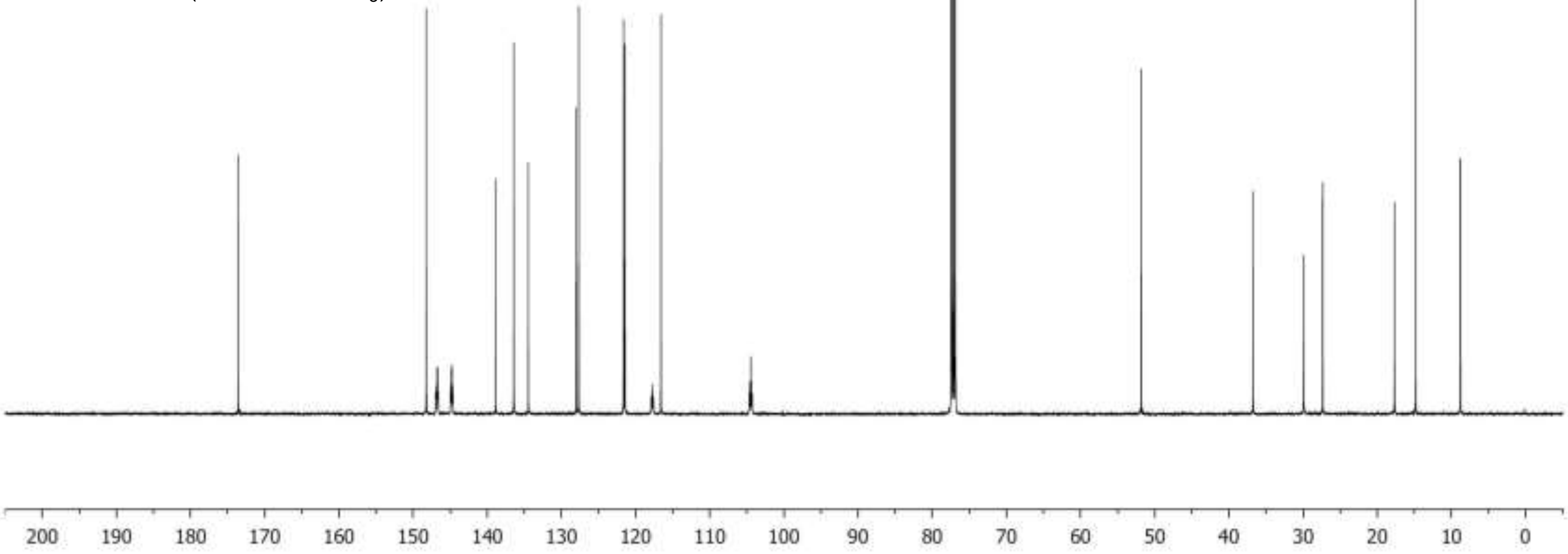

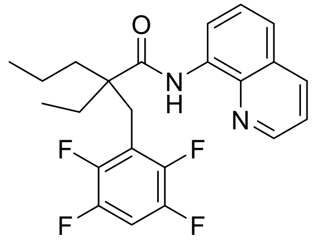

3f

^{19}F NMR (471 MHz, $CDCl_3$)

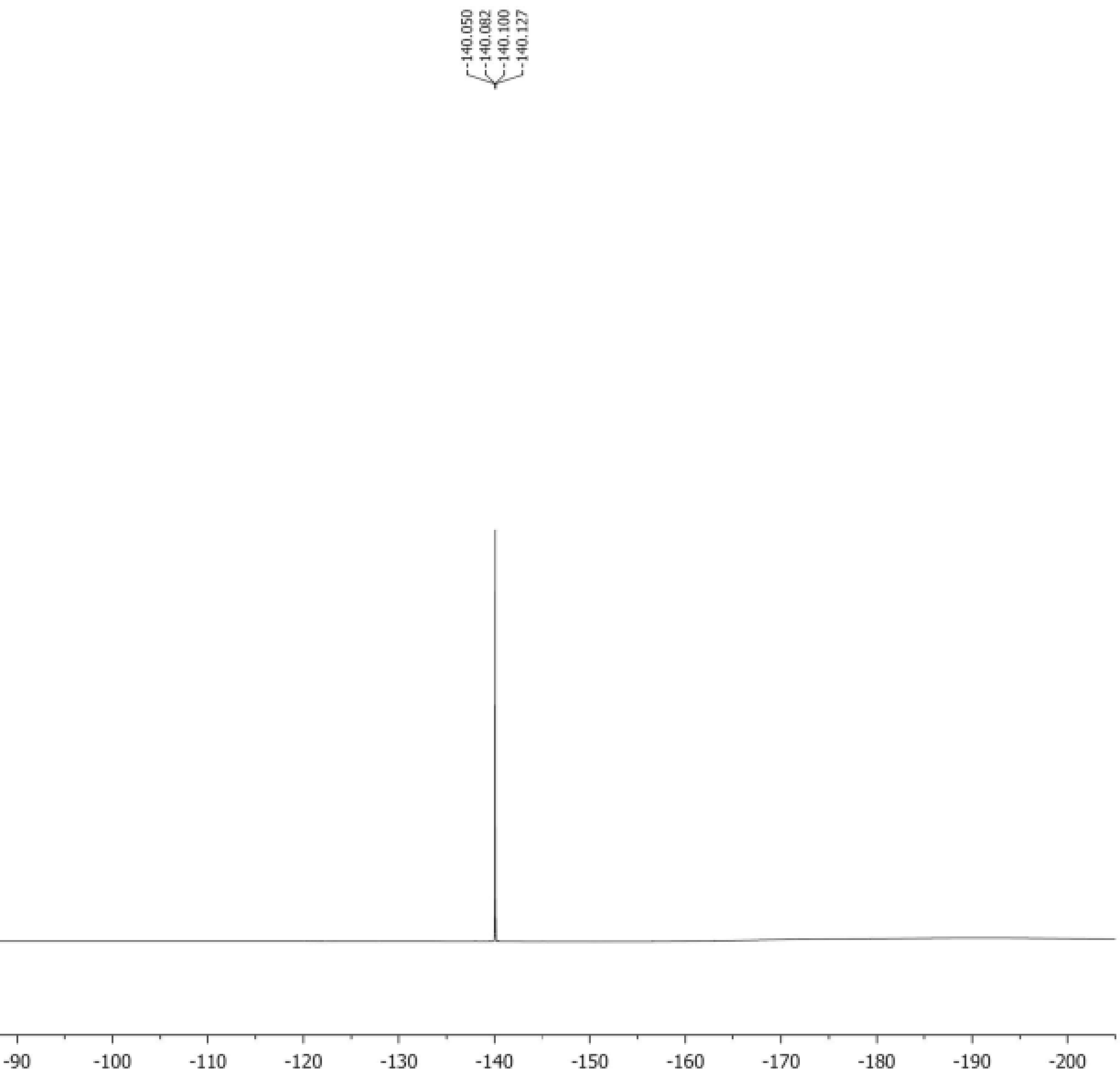

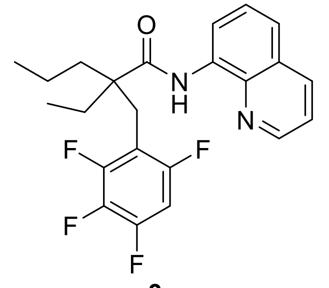

3g

^{1}H NMR (500 MHz, $CDCl_3$)

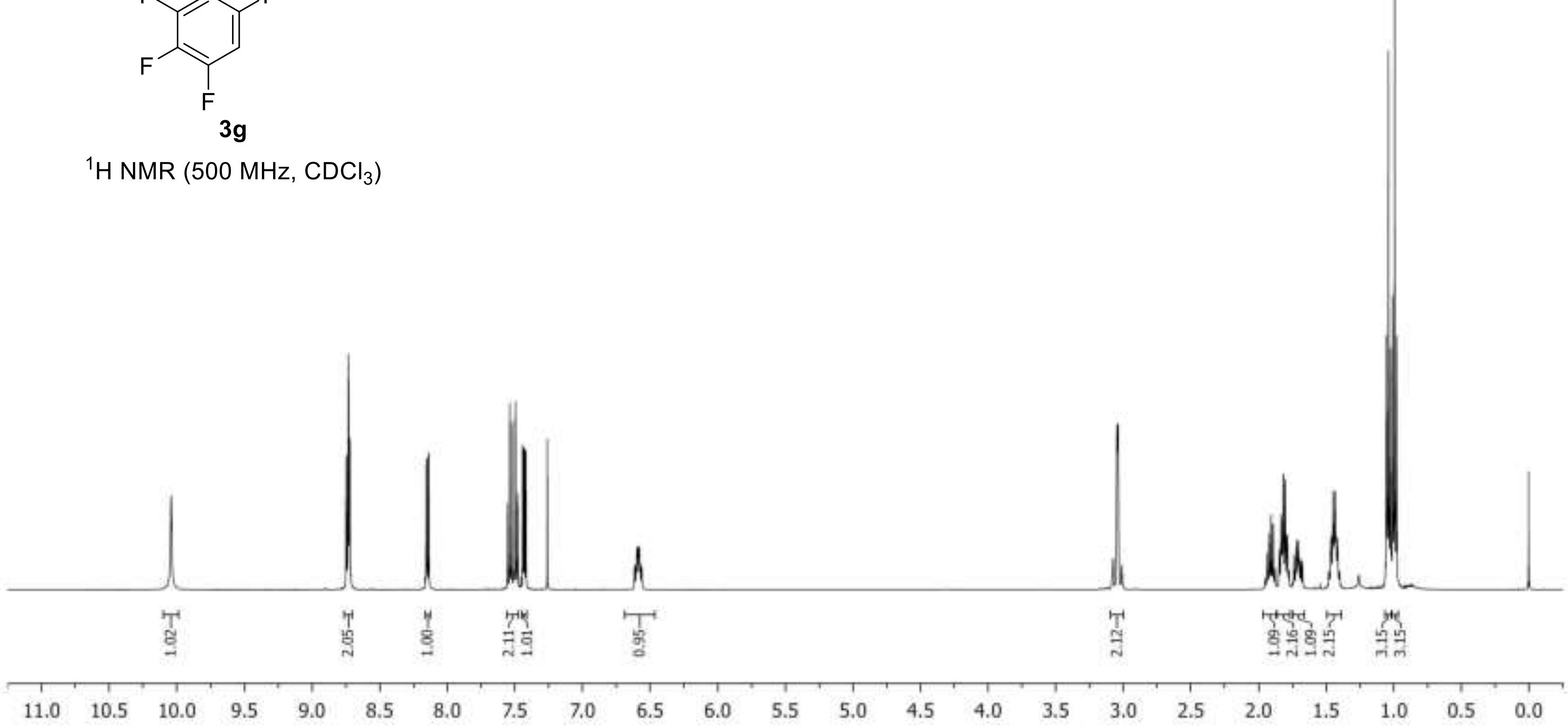

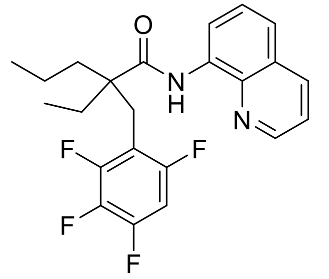

3g

^{13}C NMR (125 MHz, $CDCl_3$)

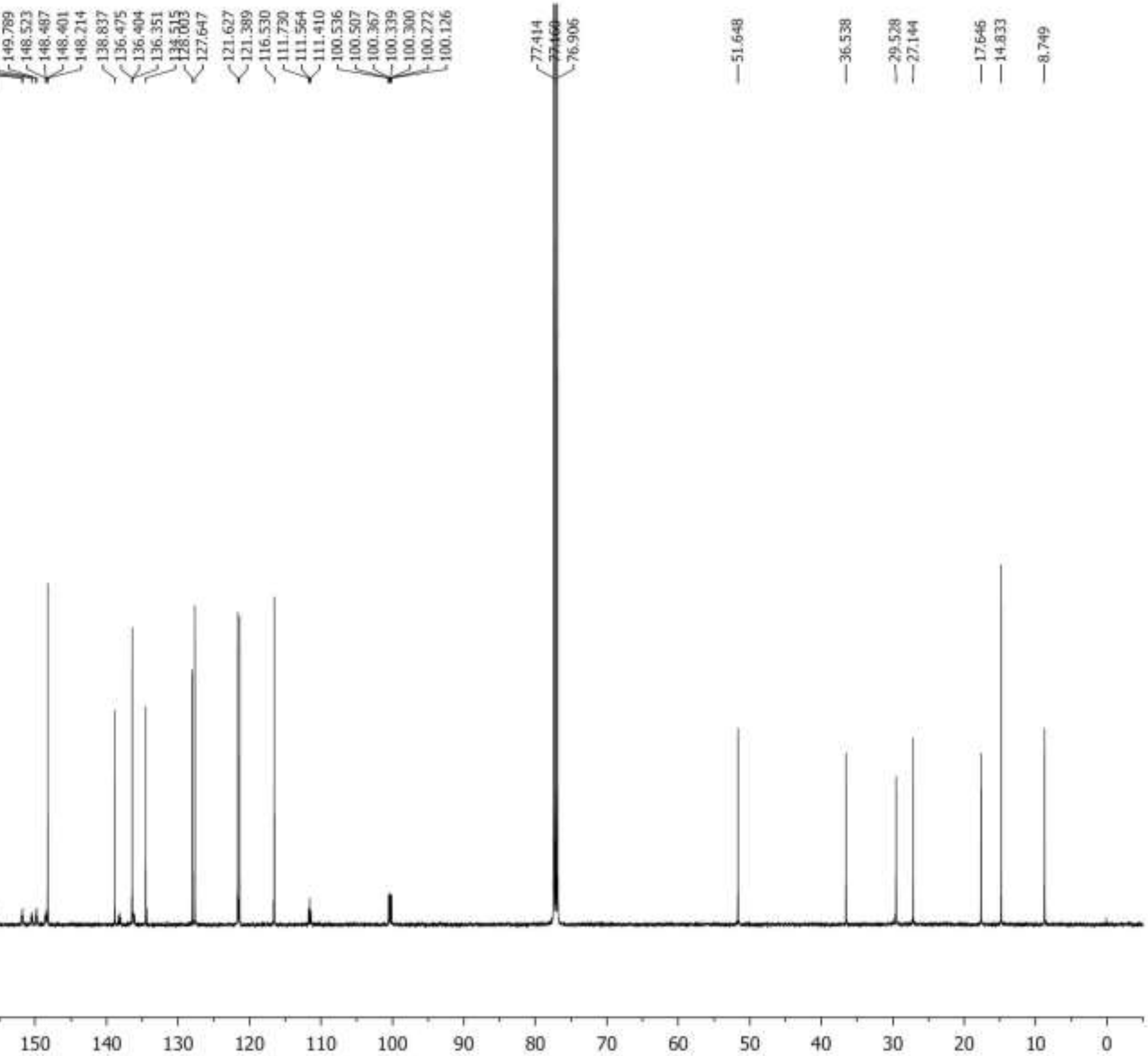

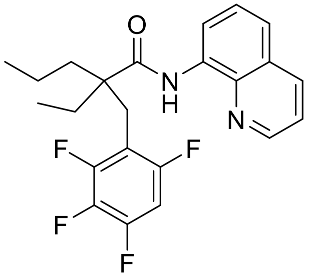

3g

^{19}F NMR (471 MHz, $CDCl_3$)

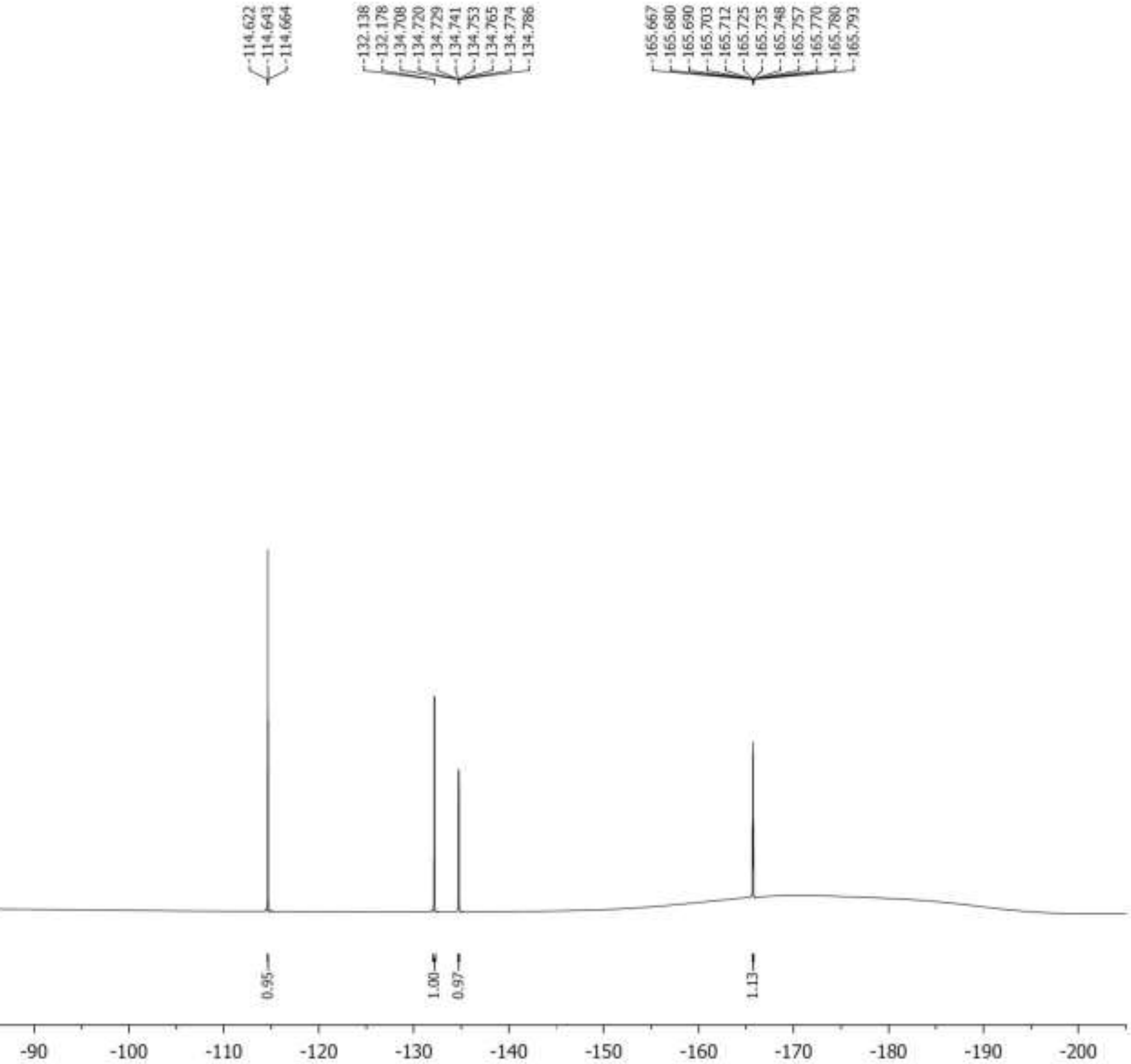

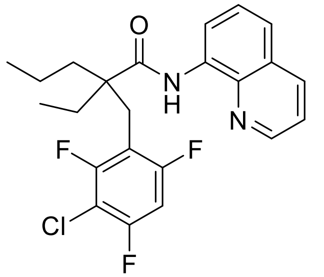

3h

^{1}H NMR (500 MHz, $CDCl_3$)

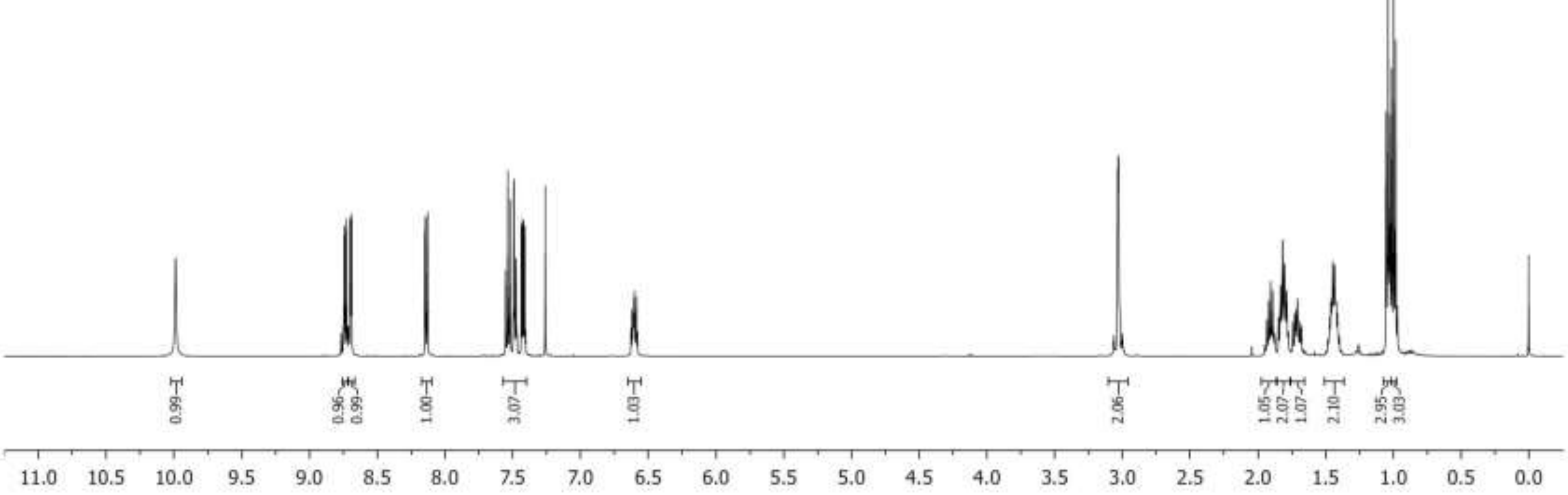

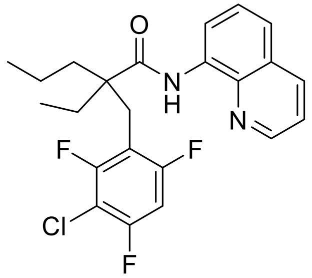

3h

^{13}C NMR (125 MHz, $CDCl_3$)

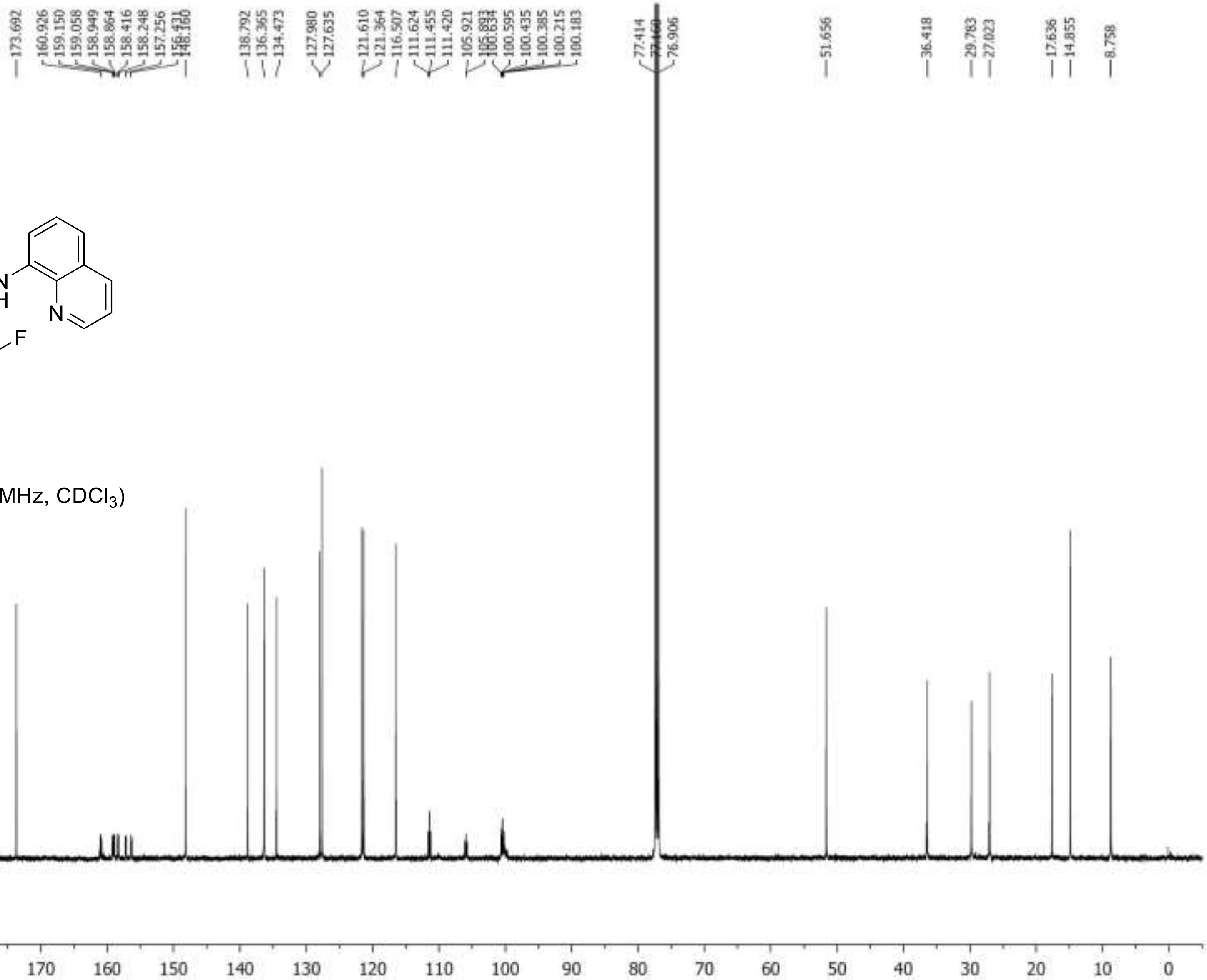

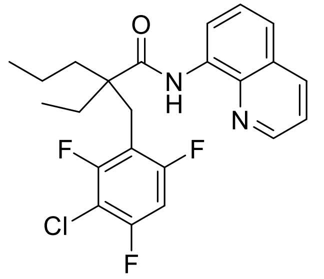

3h

^{19}F NMR (471 MHz, $CDCl_3$)

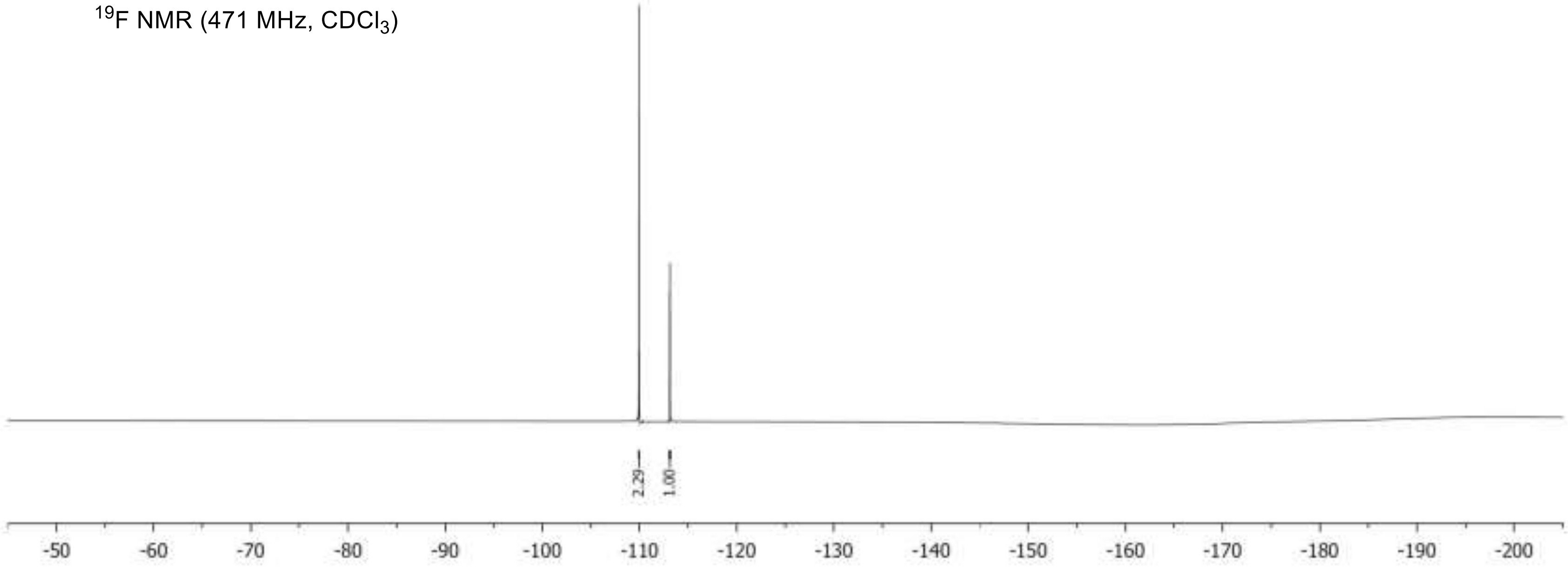

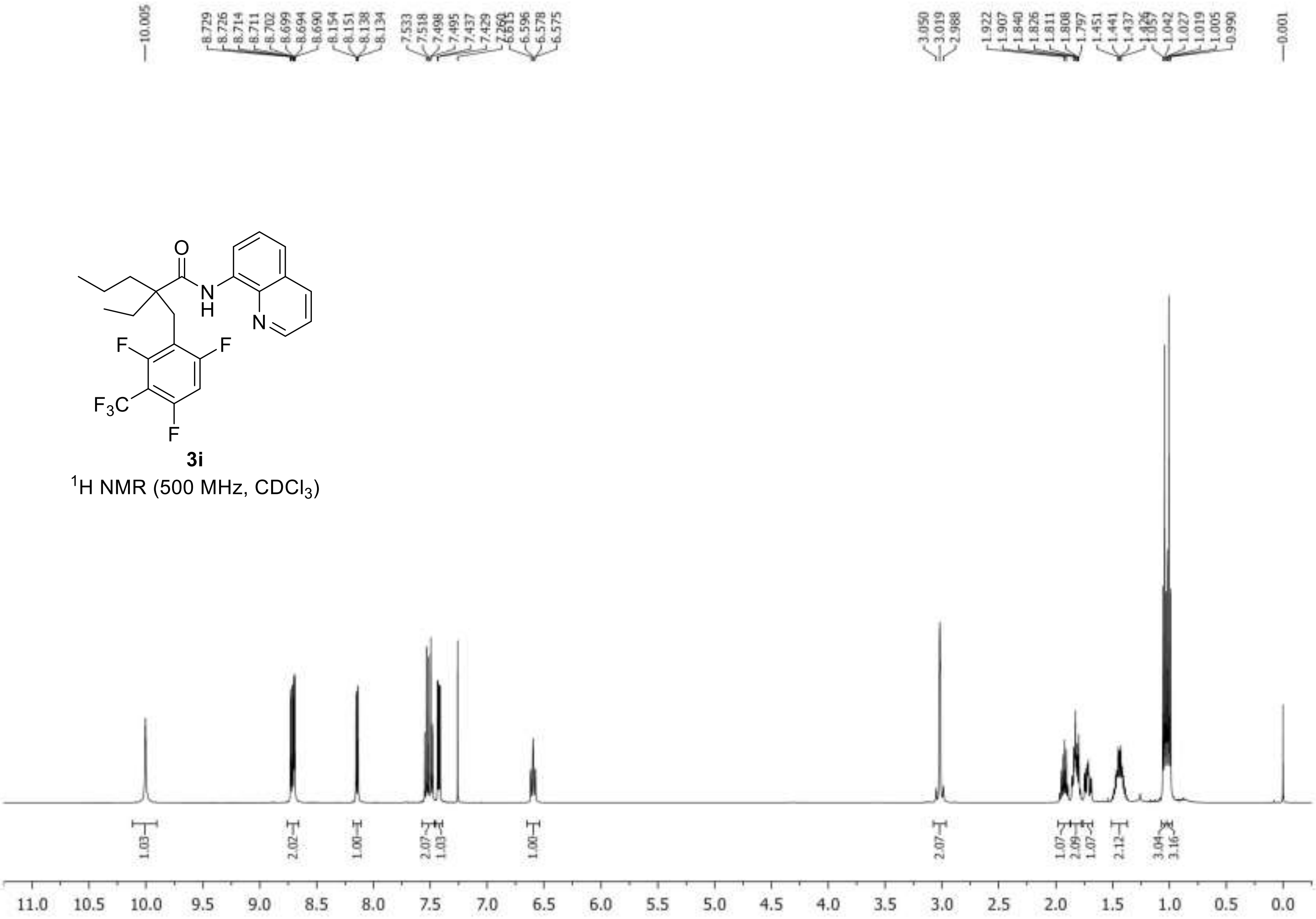

3i

^{1}H NMR (500 MHz, $CDCl_3$)

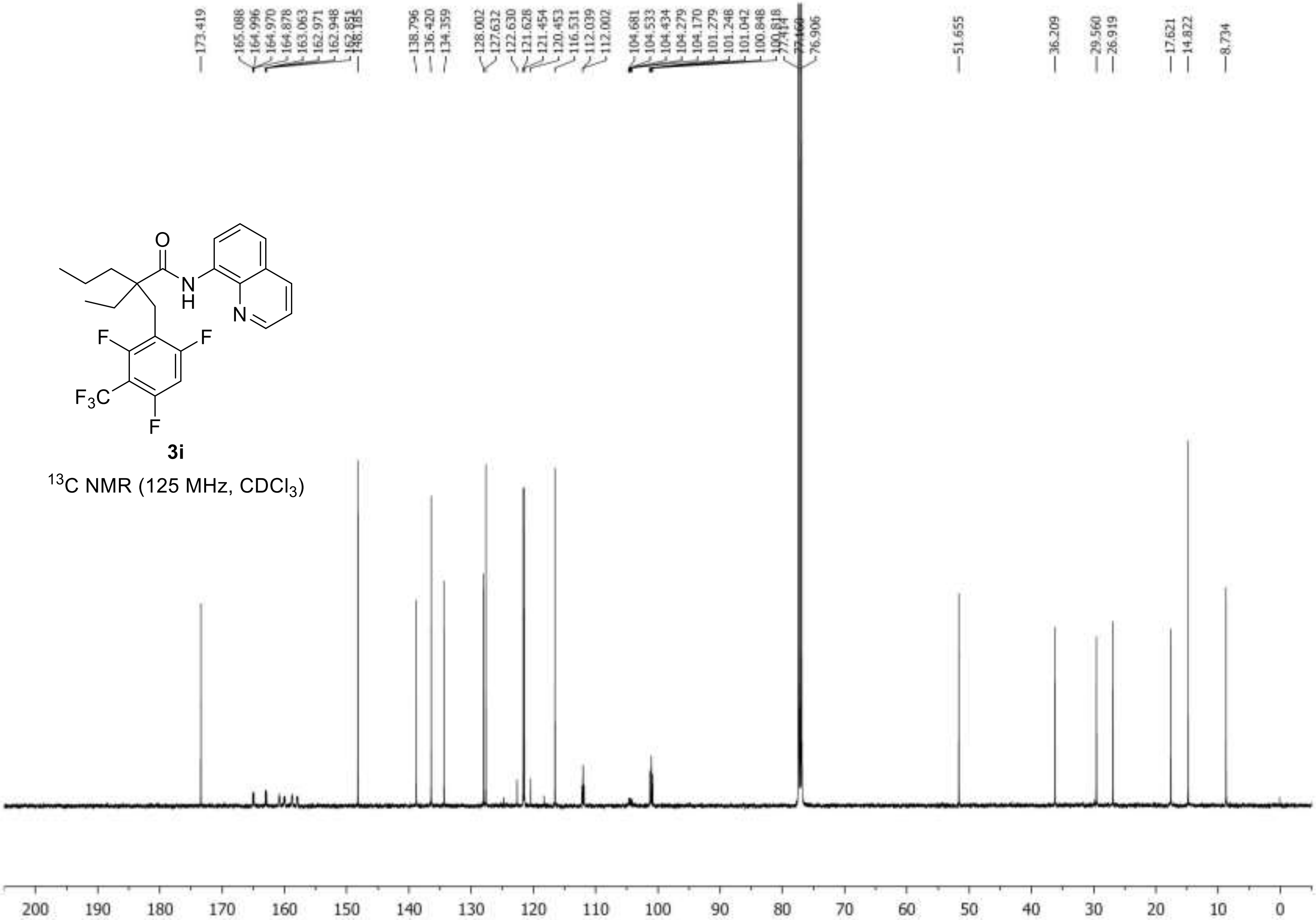

3i

^{13}C NMR (125 MHz, $CDCl_3$)

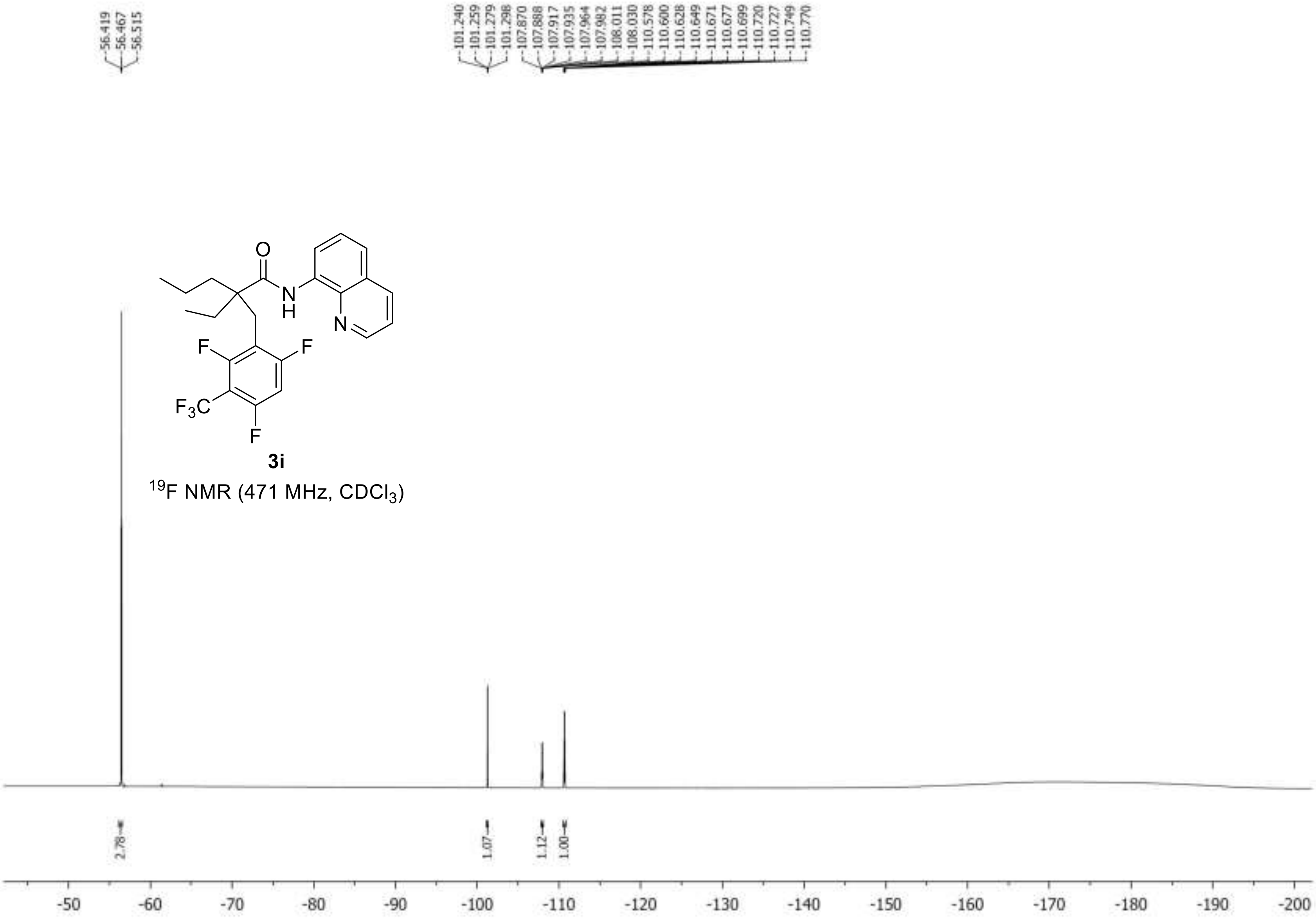

3i

^{19}F NMR (471 MHz, $CDCl_3$)

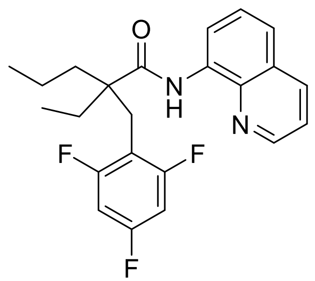

3j

^{1}H NMR (500 MHz, $CDCl_3$)

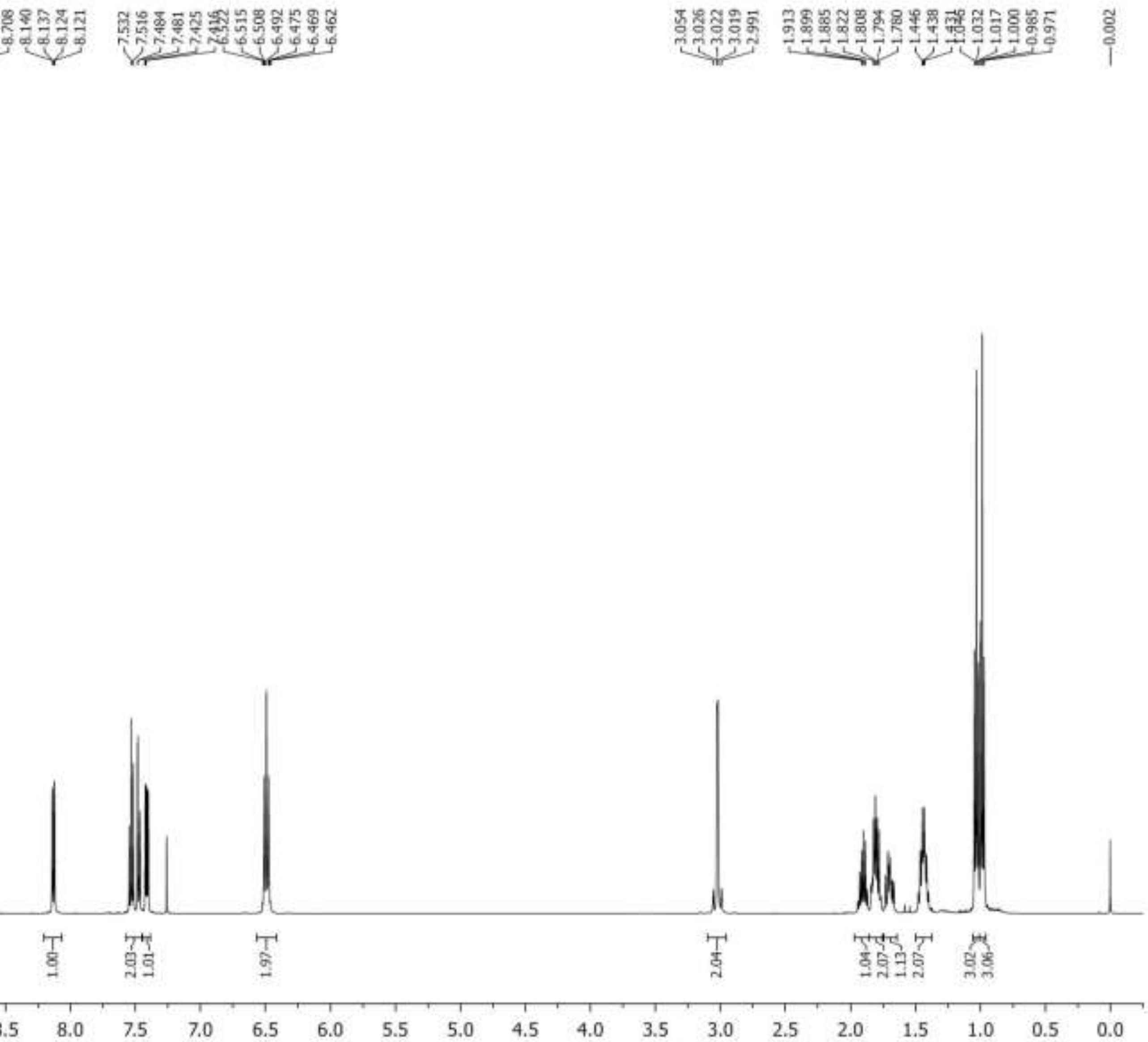

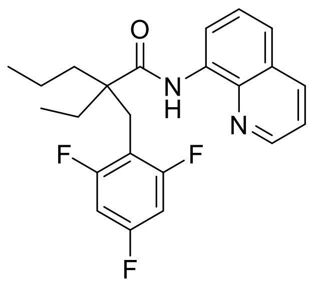

3j

^{13}C NMR (125 MHz, $CDCl_3$)

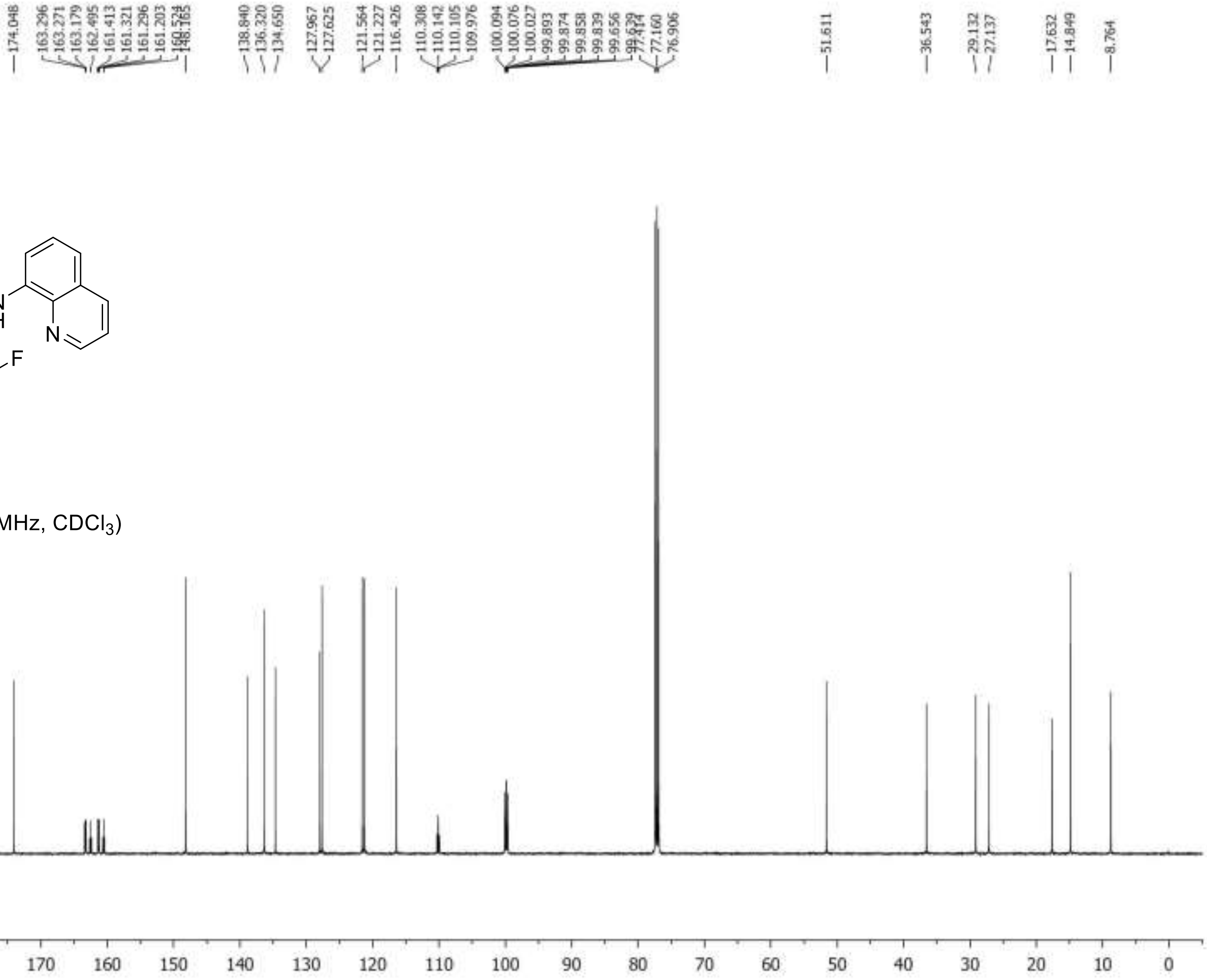

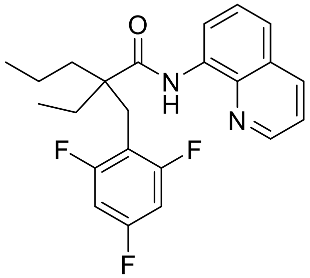

3j

^{19}F NMR (471 MHz, $CDCl_3$)

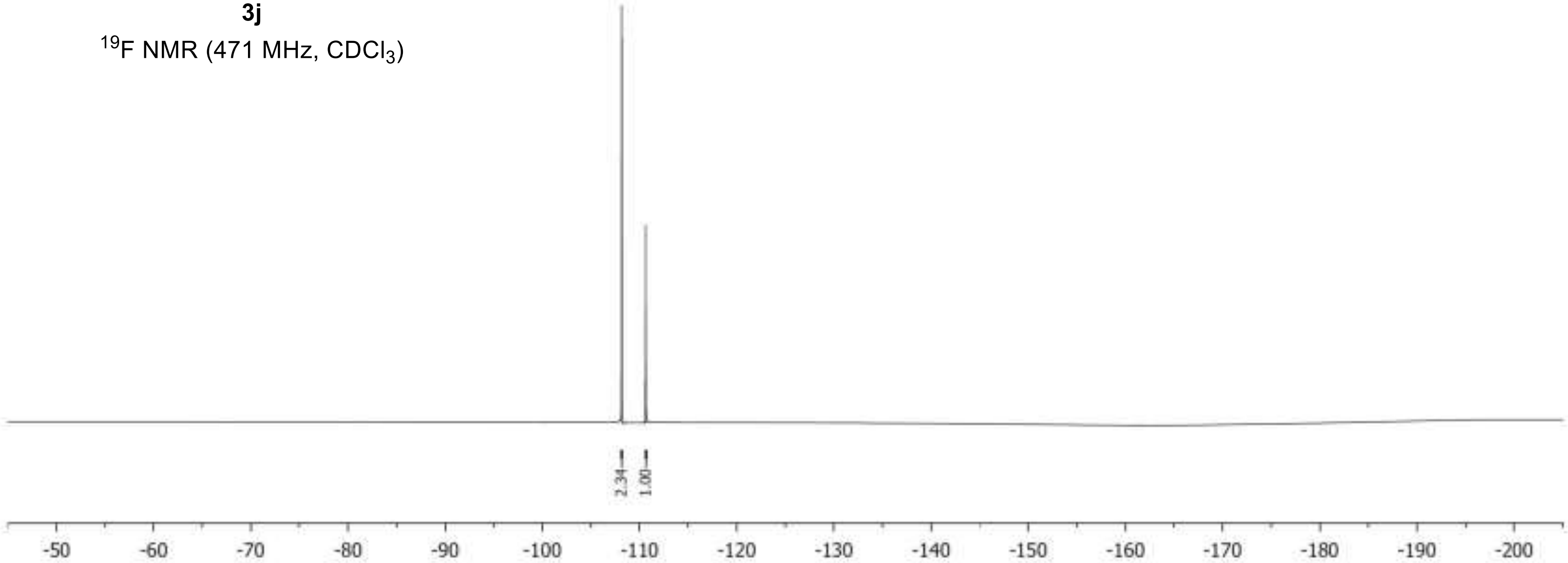

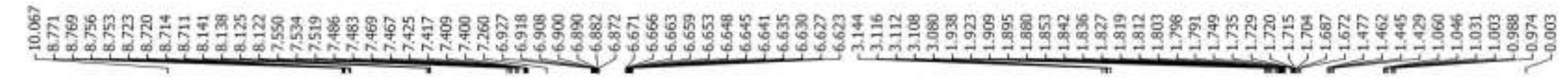

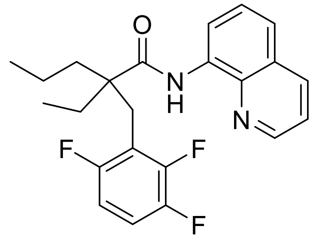

3k

^{1}H NMR (500 MHz, $CDCl_3$)

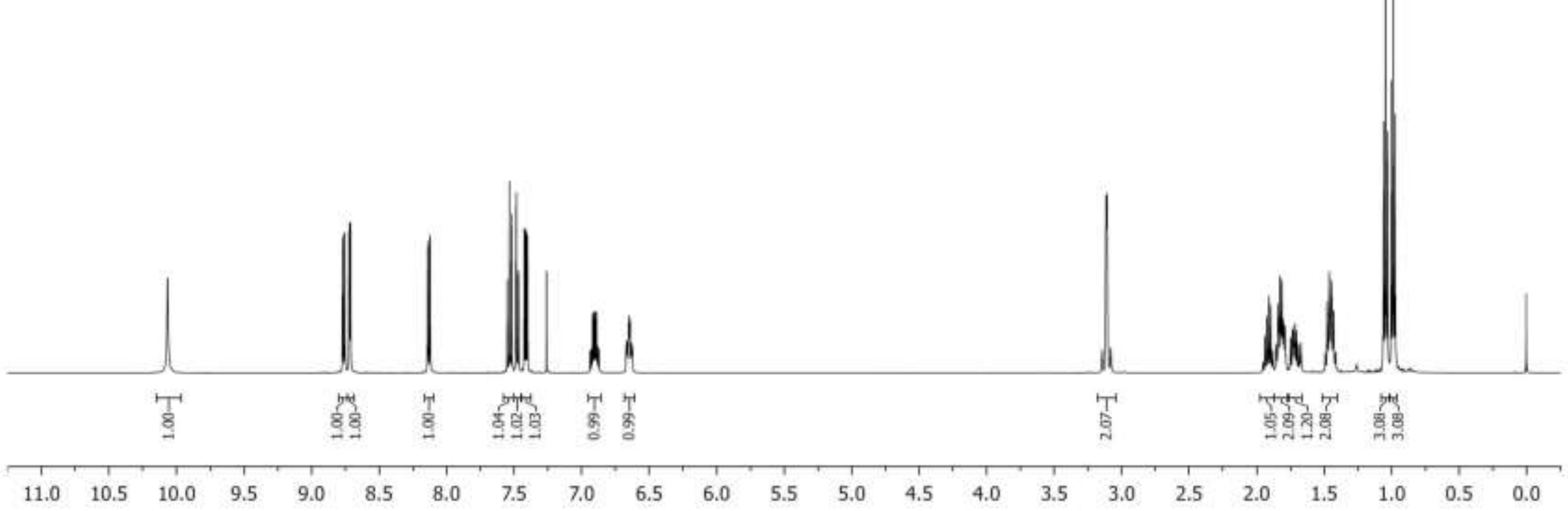

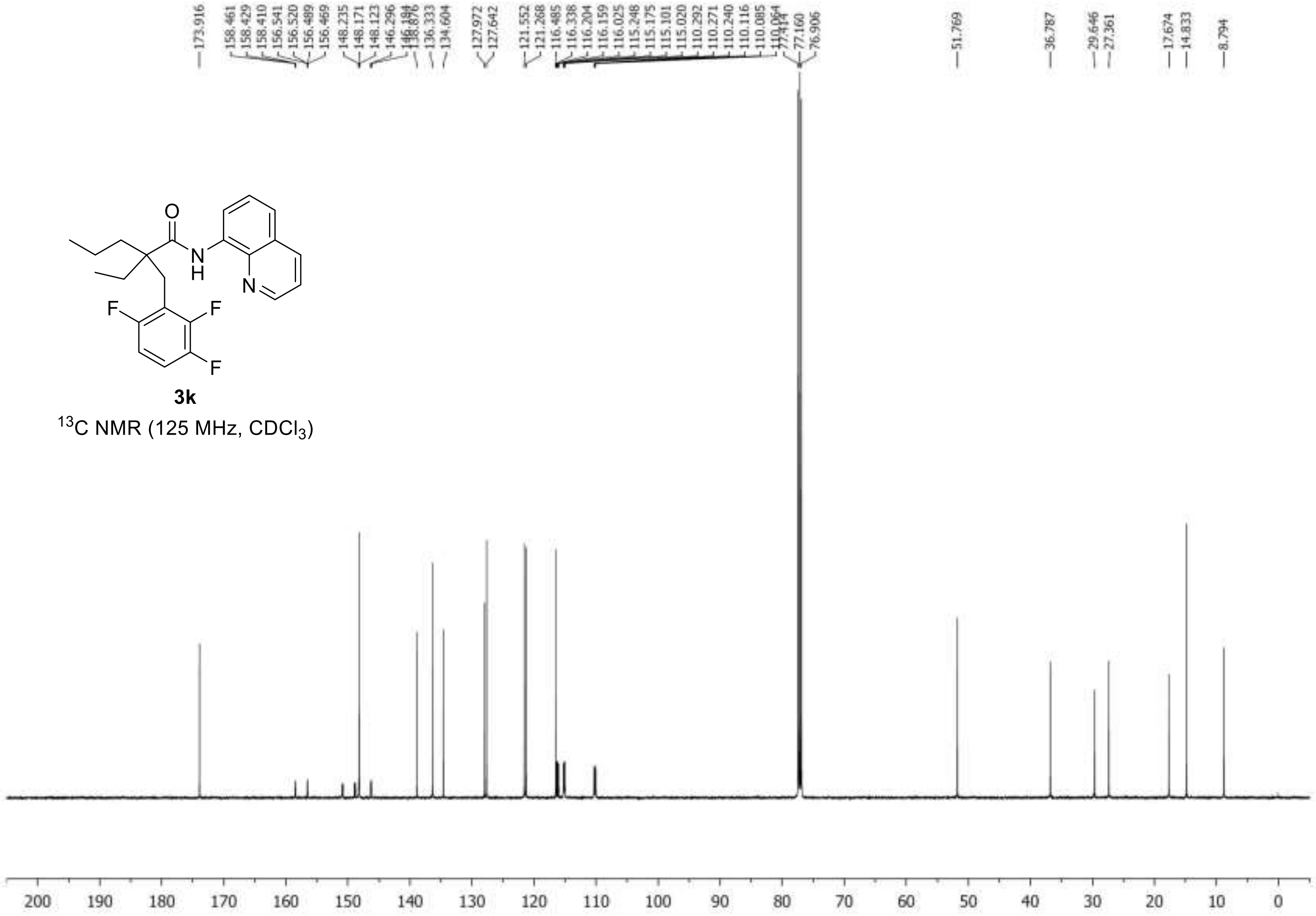

3k

^{13}C NMR (125 MHz, $CDCl_3$)

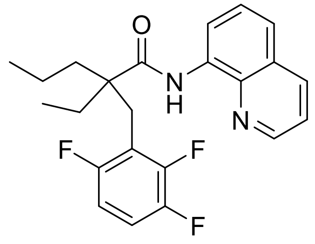

3k

^{19}F NMR (471 MHz, $CDCl_3$)

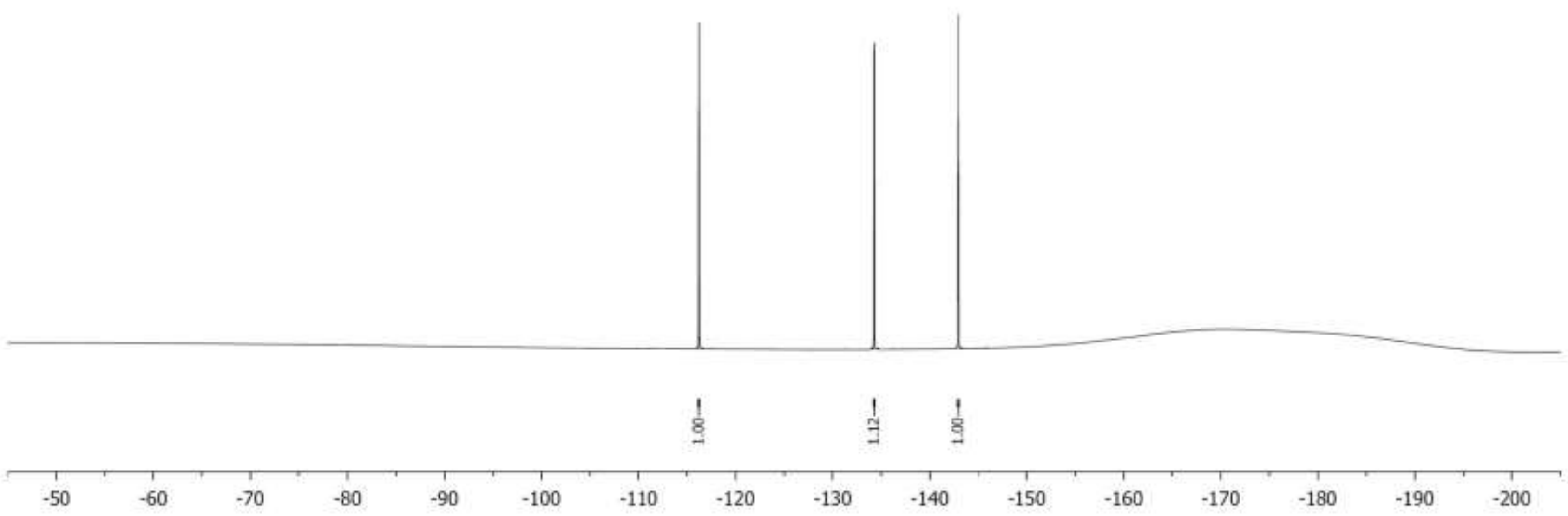

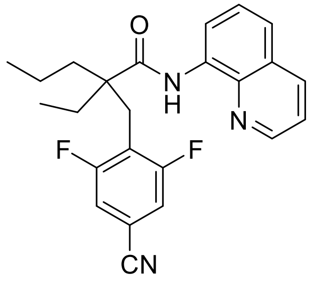

3l

^1H NMR (500 MHz, $CDCl_3$)

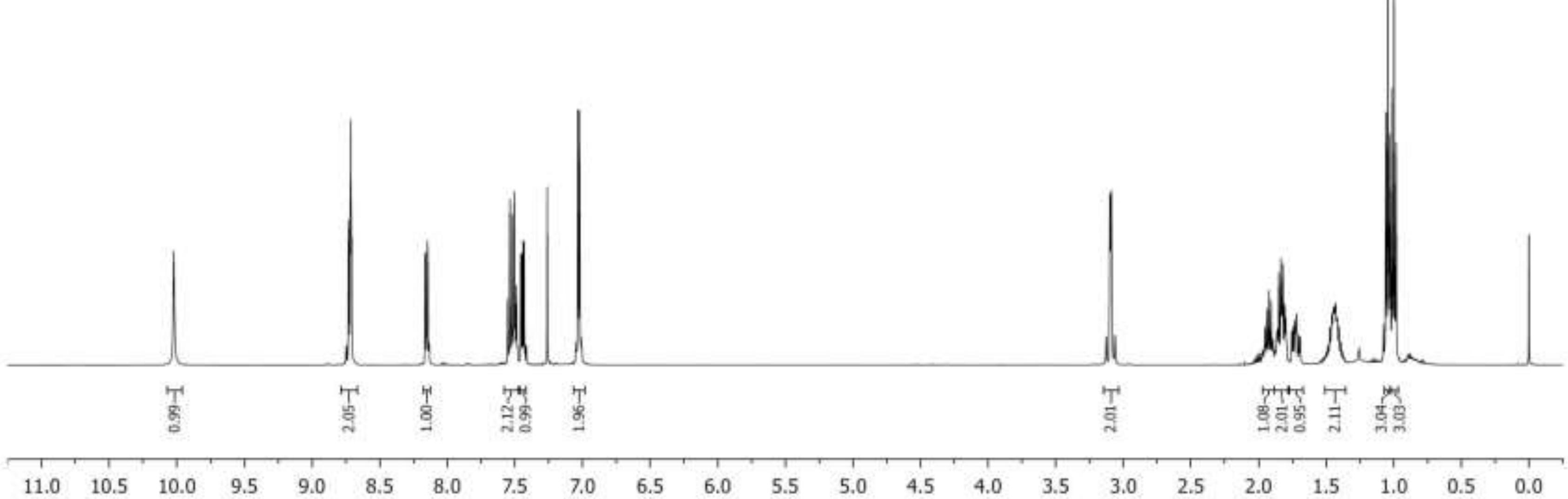

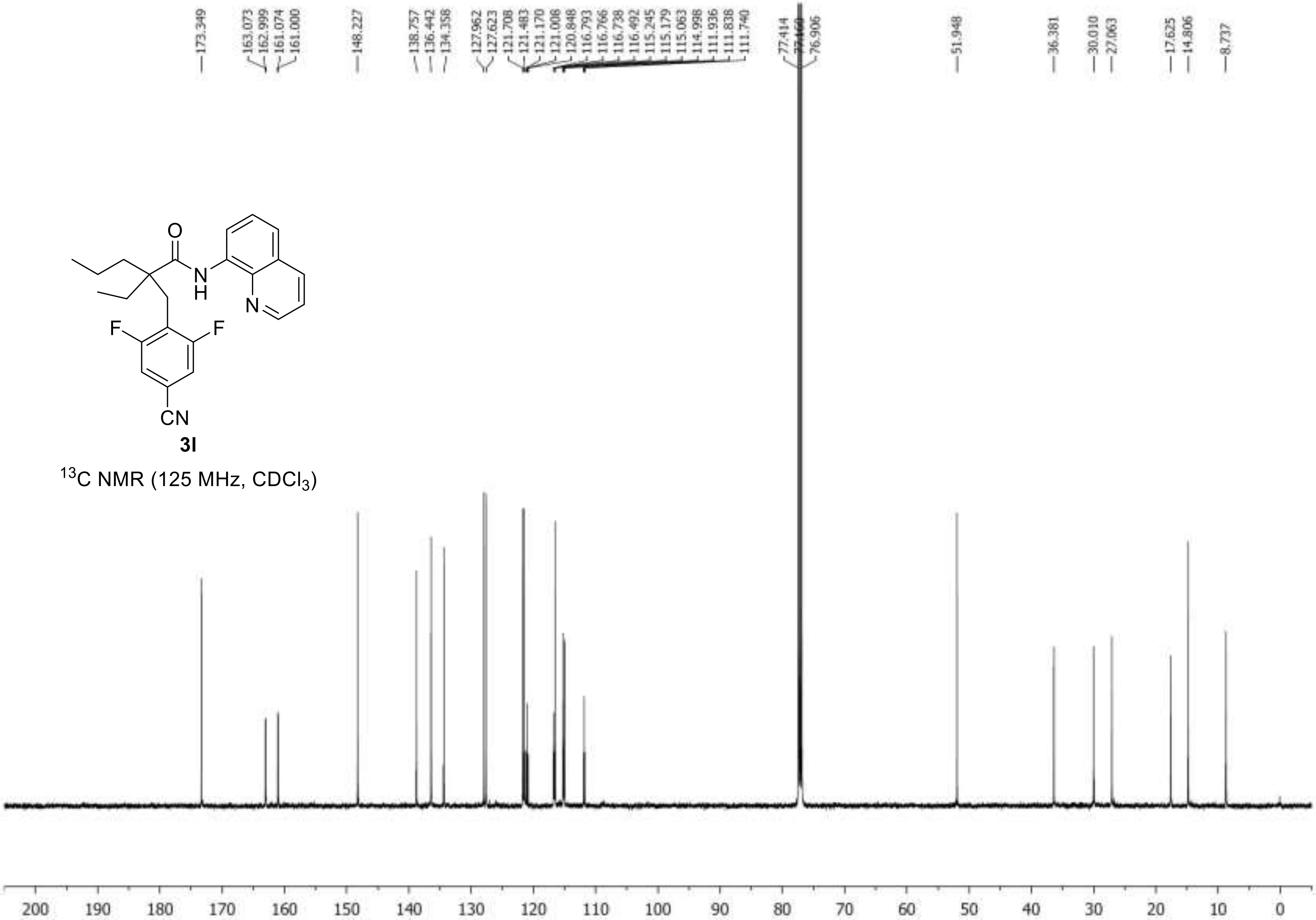

3l

^{13}C NMR (125 MHz, $CDCl_3$)

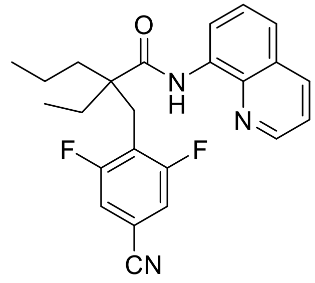

3l

^{19}F NMR (471 MHz, $CDCl_3$)

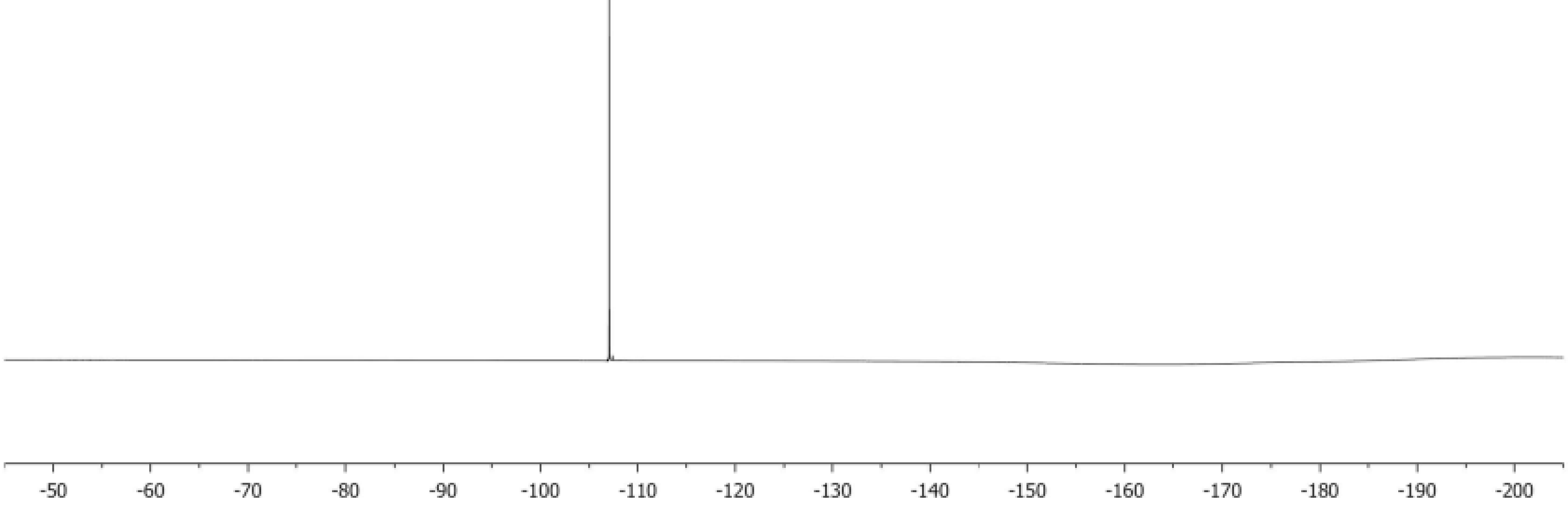

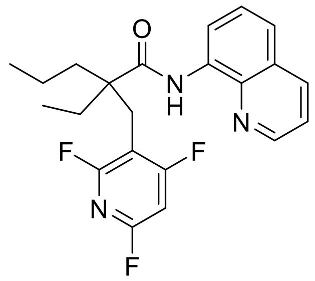

3m

^{1}H NMR (500 MHz, $CDCl_3$)

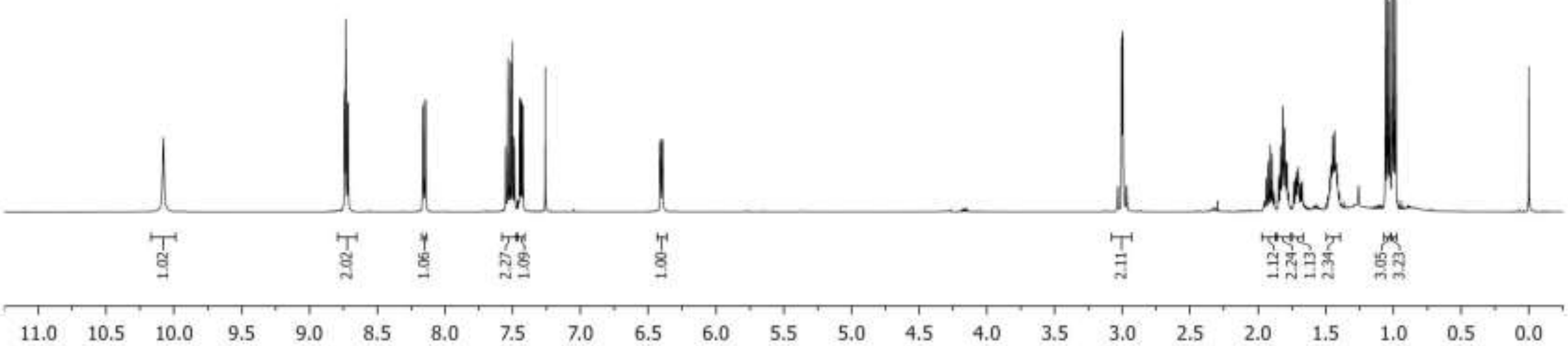

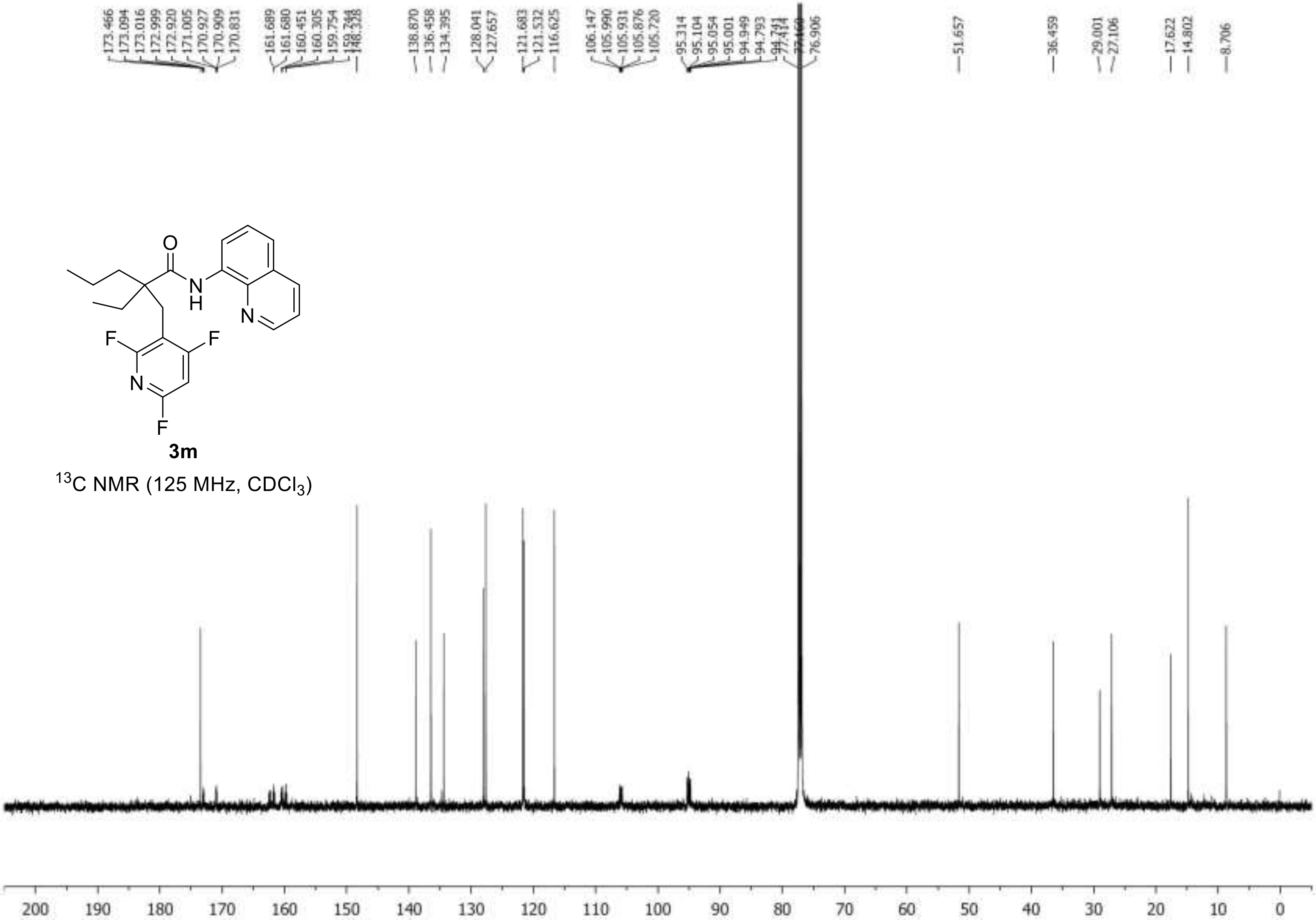

3m

^{13}C NMR (125 MHz, $CDCl_3$)

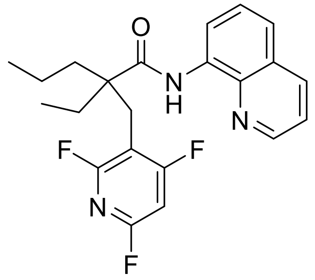

3m

^{19}F NMR (471 MHz, $CDCl_3$)

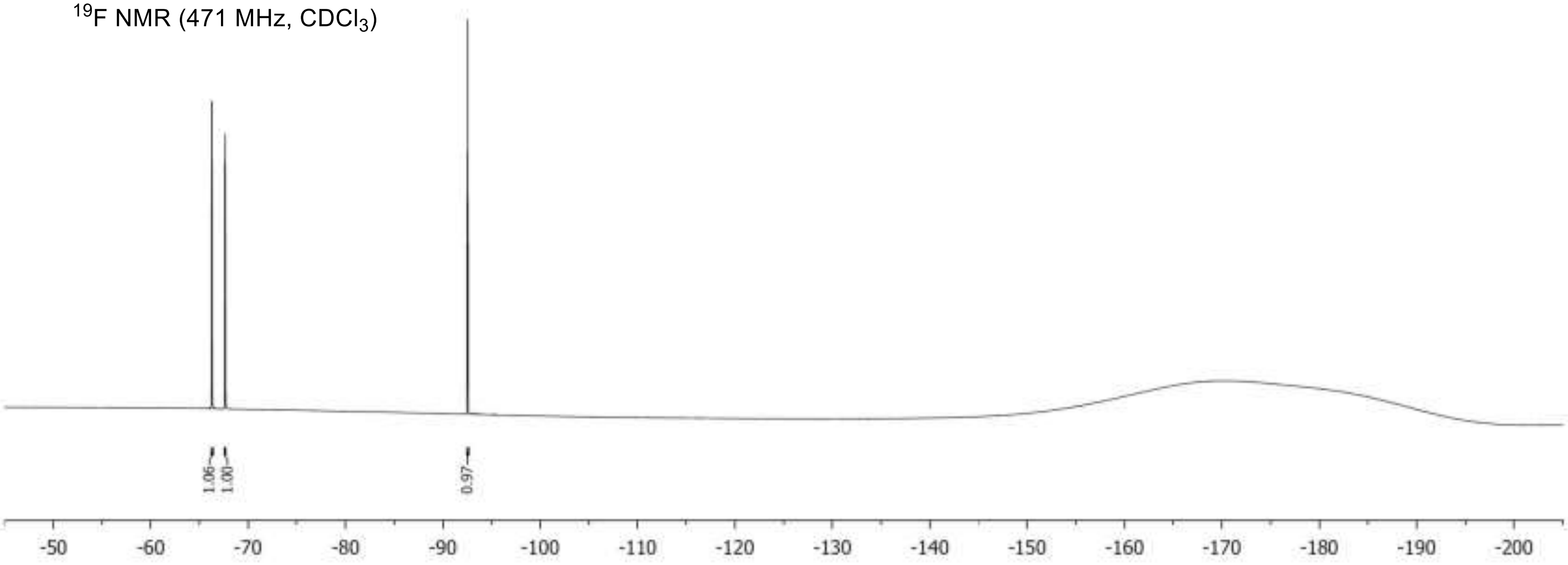

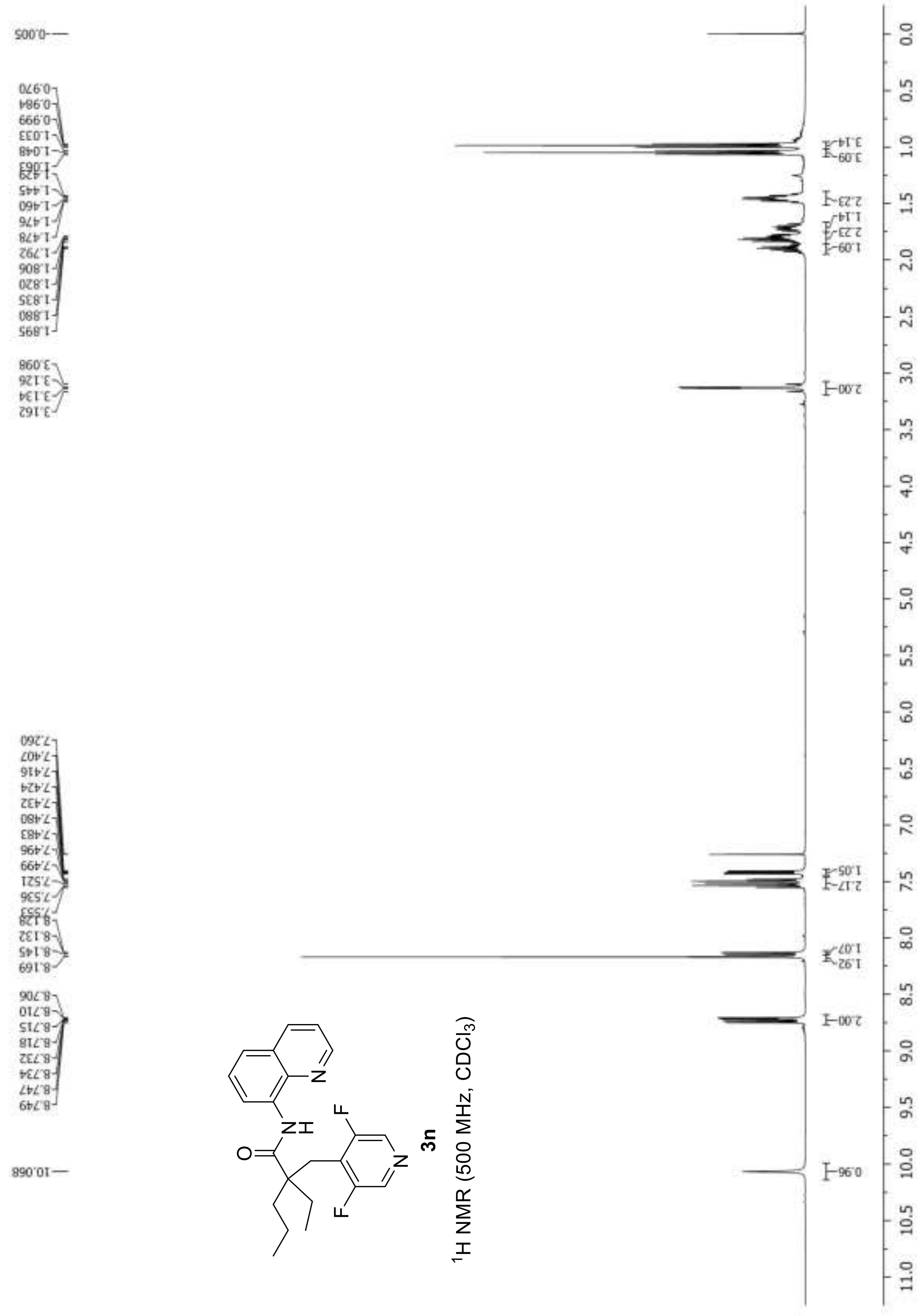

3n

^1H NMR (500 MHz, $CDCl_3$)

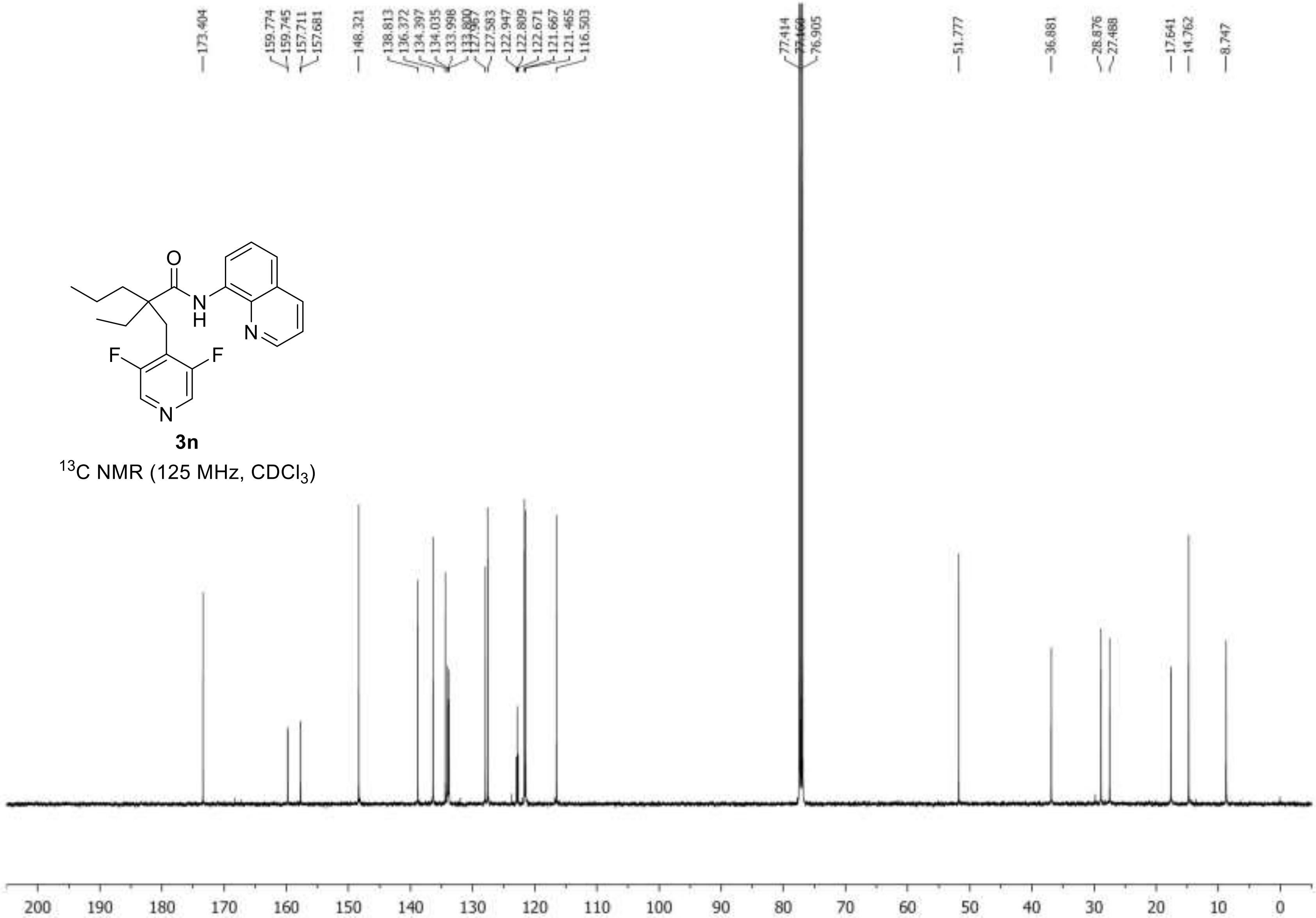

3n

^{13}C NMR (125 MHz, $CDCl_3$)

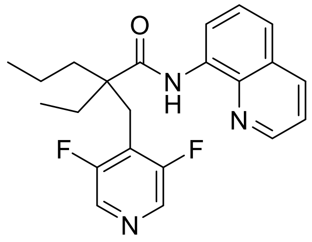

3n

^{19}F NMR (471 MHz, $CDCl_3$)

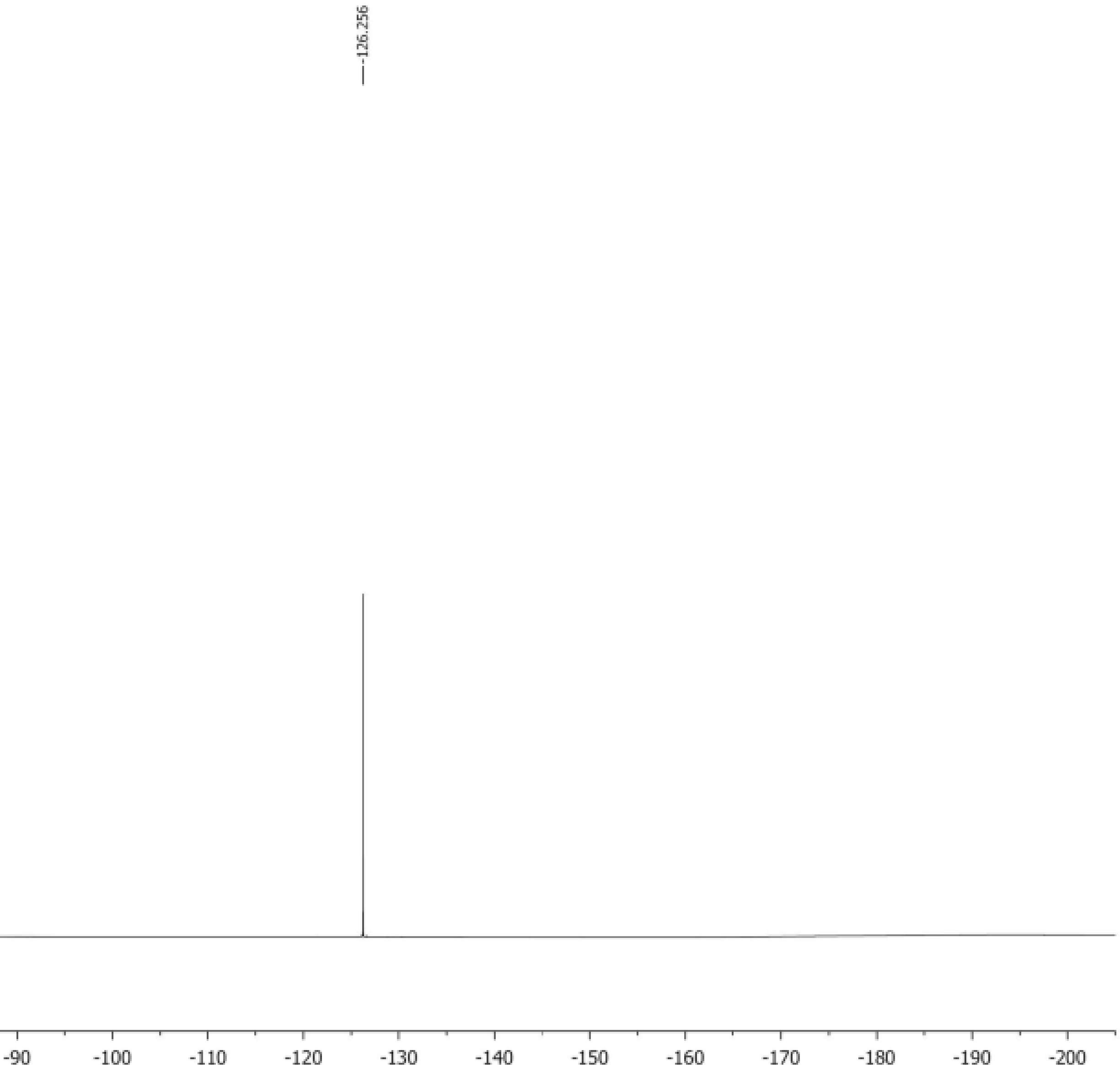

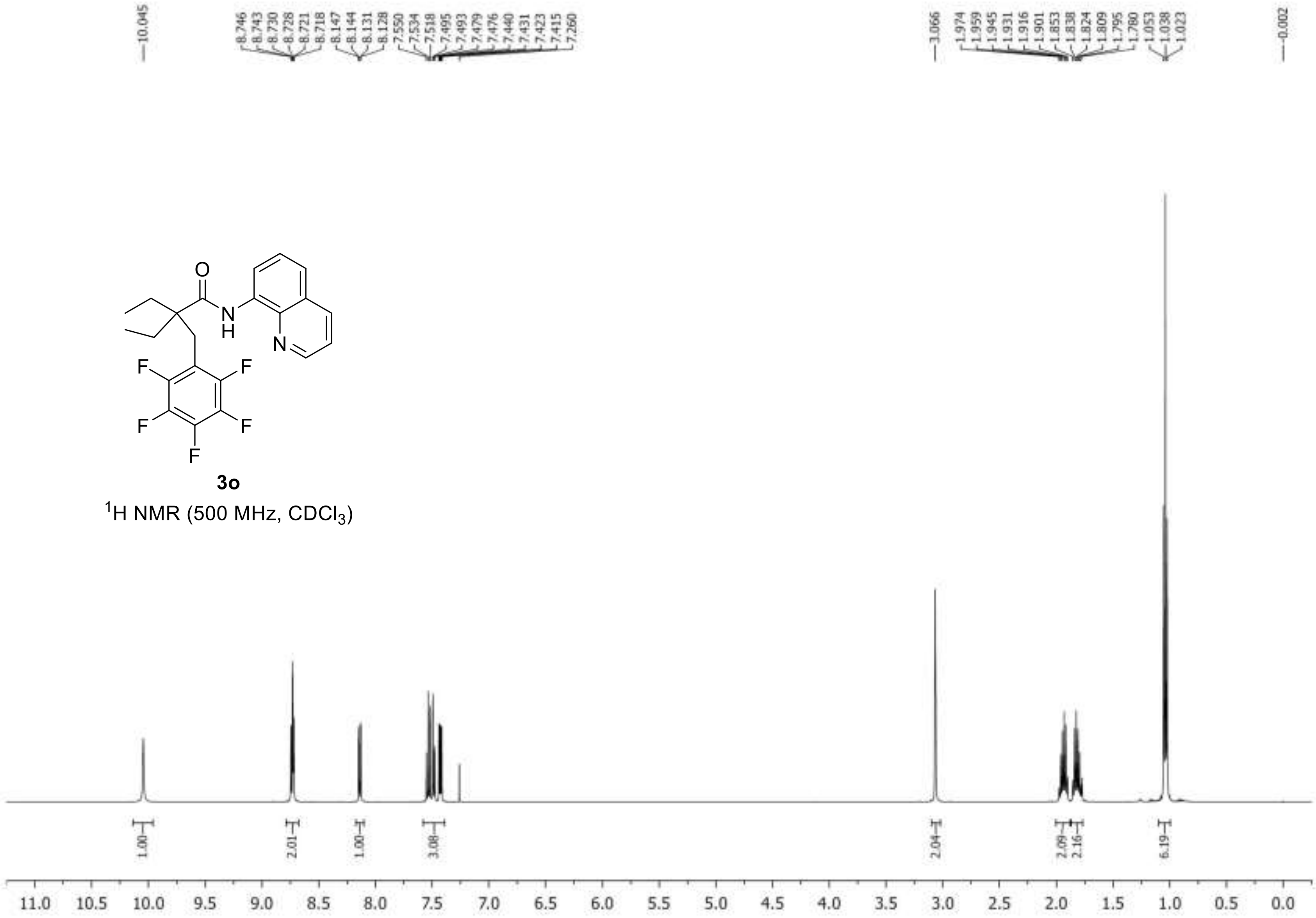

3o

^{1}H NMR (500 MHz, $CDCl_3$)

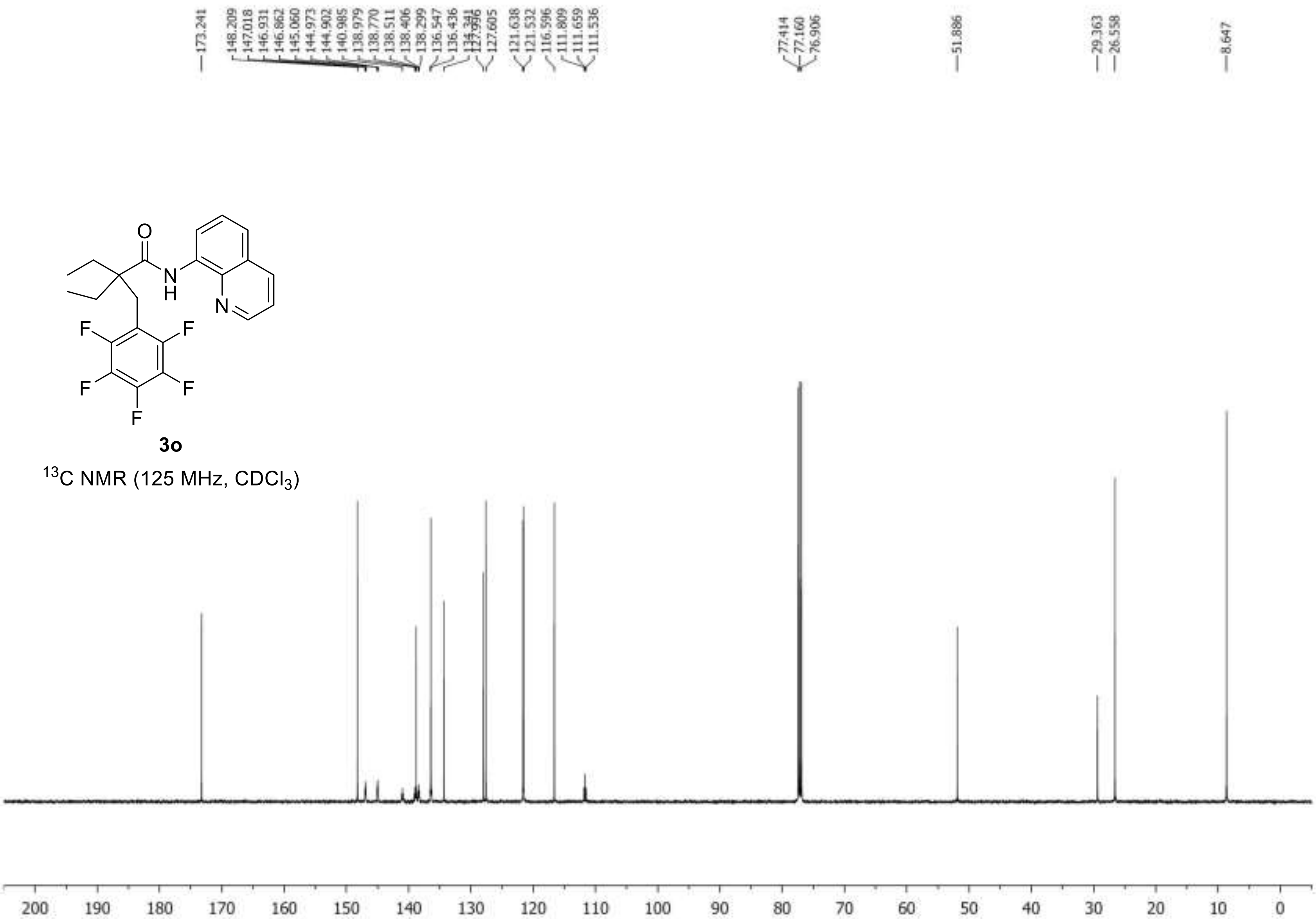

3o

^{13}C NMR (125 MHz, $CDCl_3$)

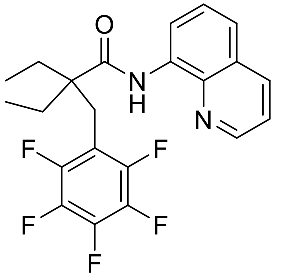

3o

^{19}F NMR (471 MHz, $CDCl_3$)

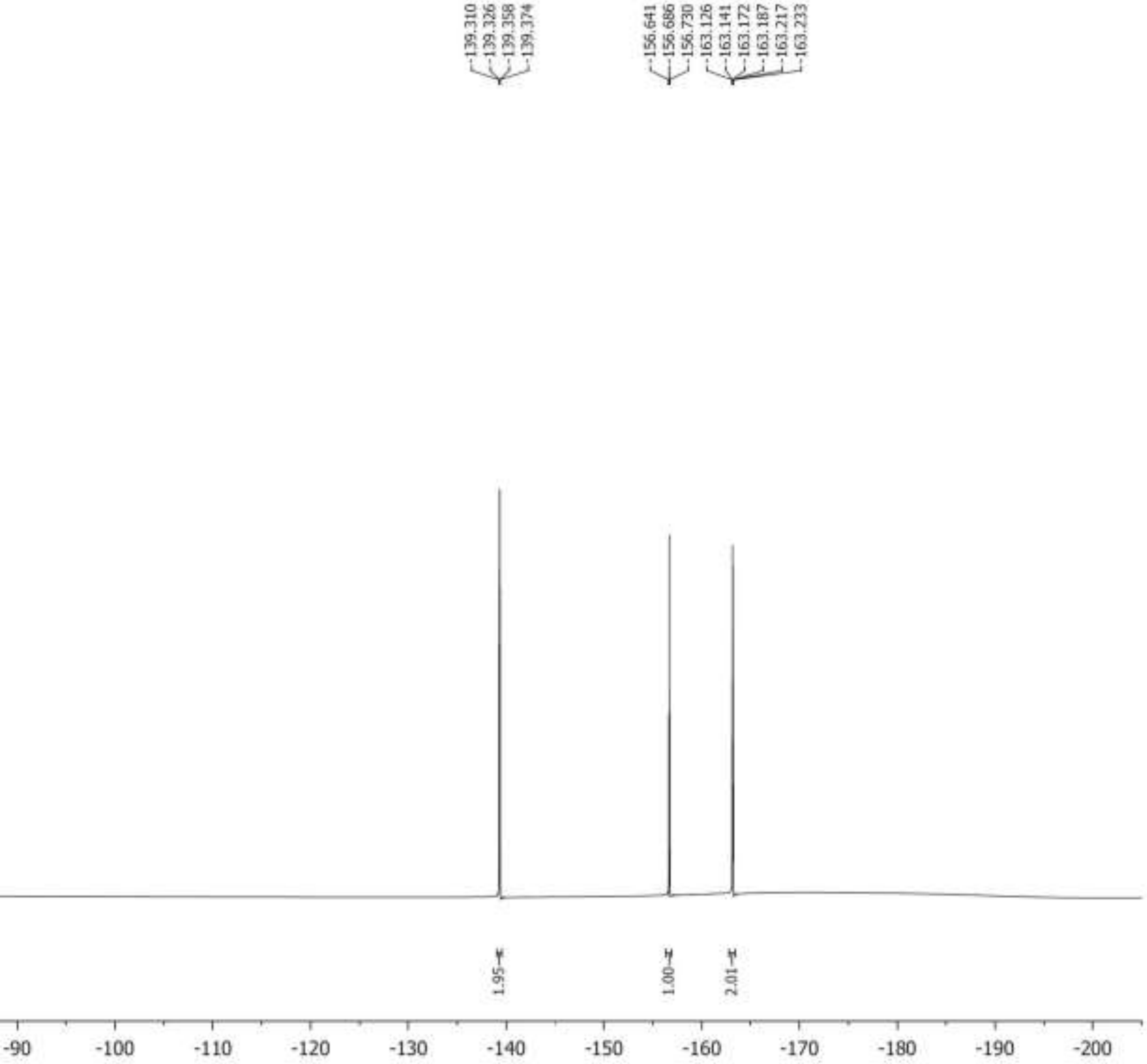

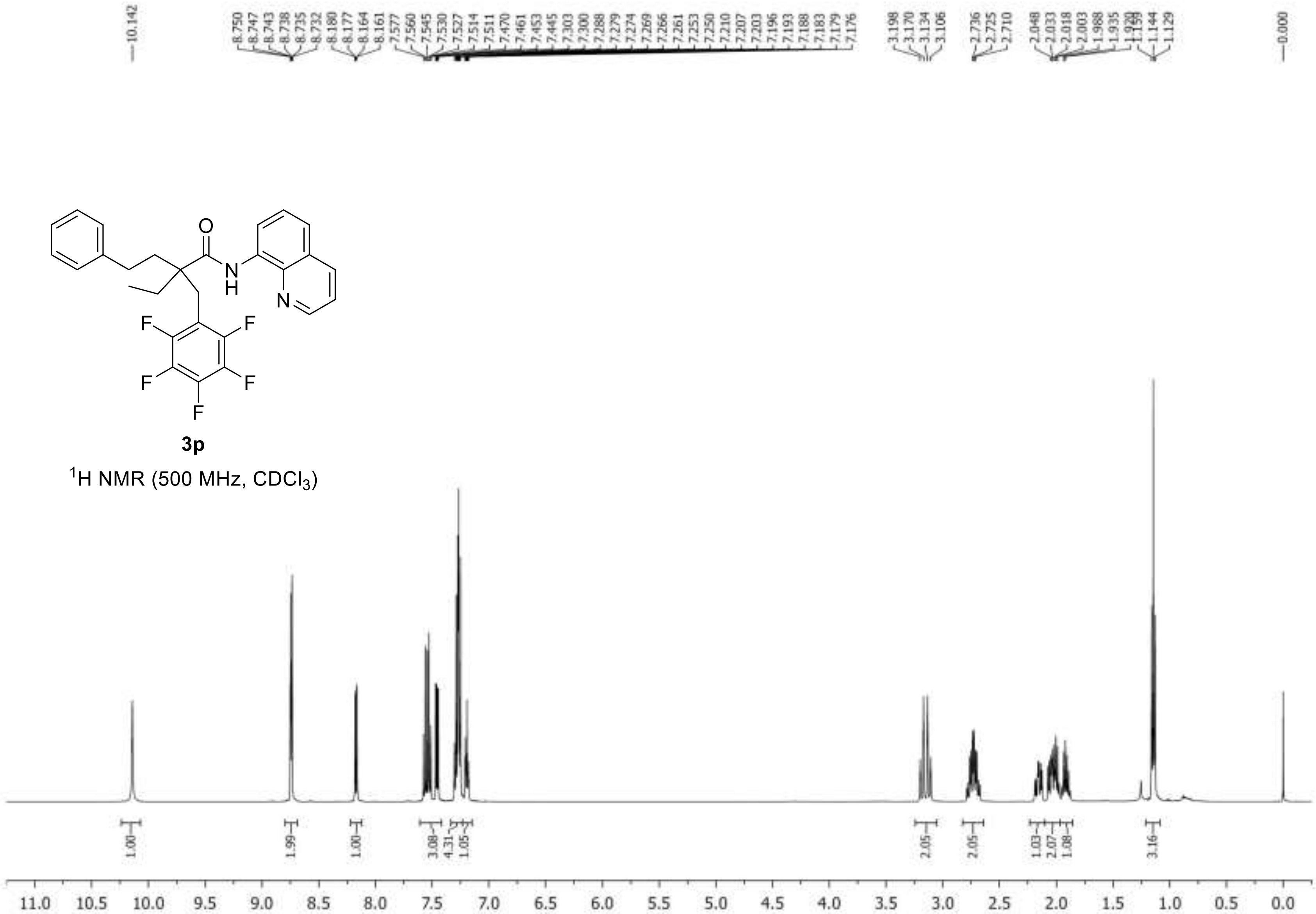

3p

^{1}H NMR (500 MHz, $CDCl_3$)

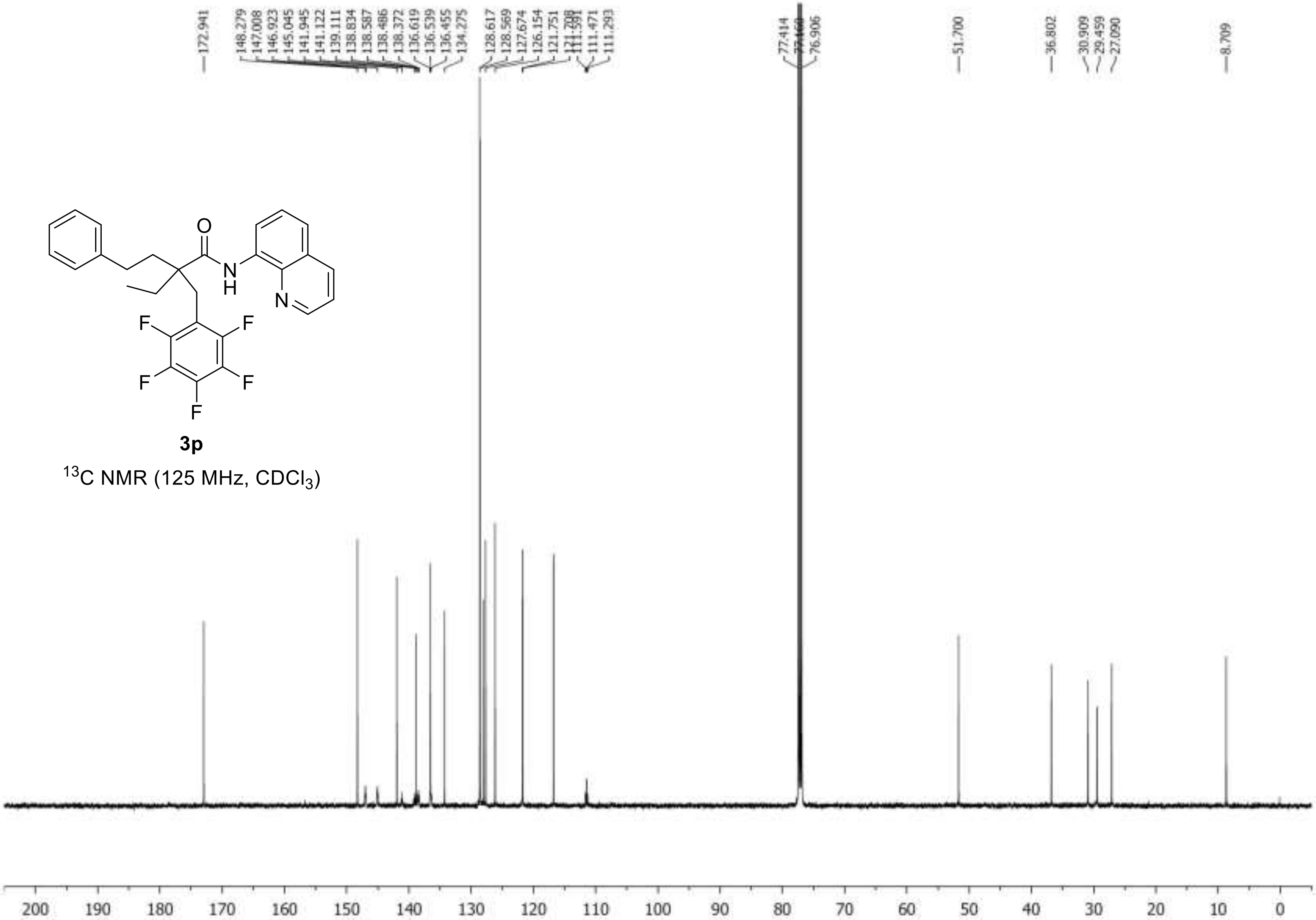

3p

^{13}C NMR (125 MHz, $CDCl_3$)

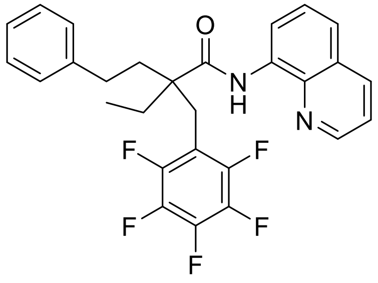

3p

^{19}F NMR (471 MHz, $CDCl_3$)

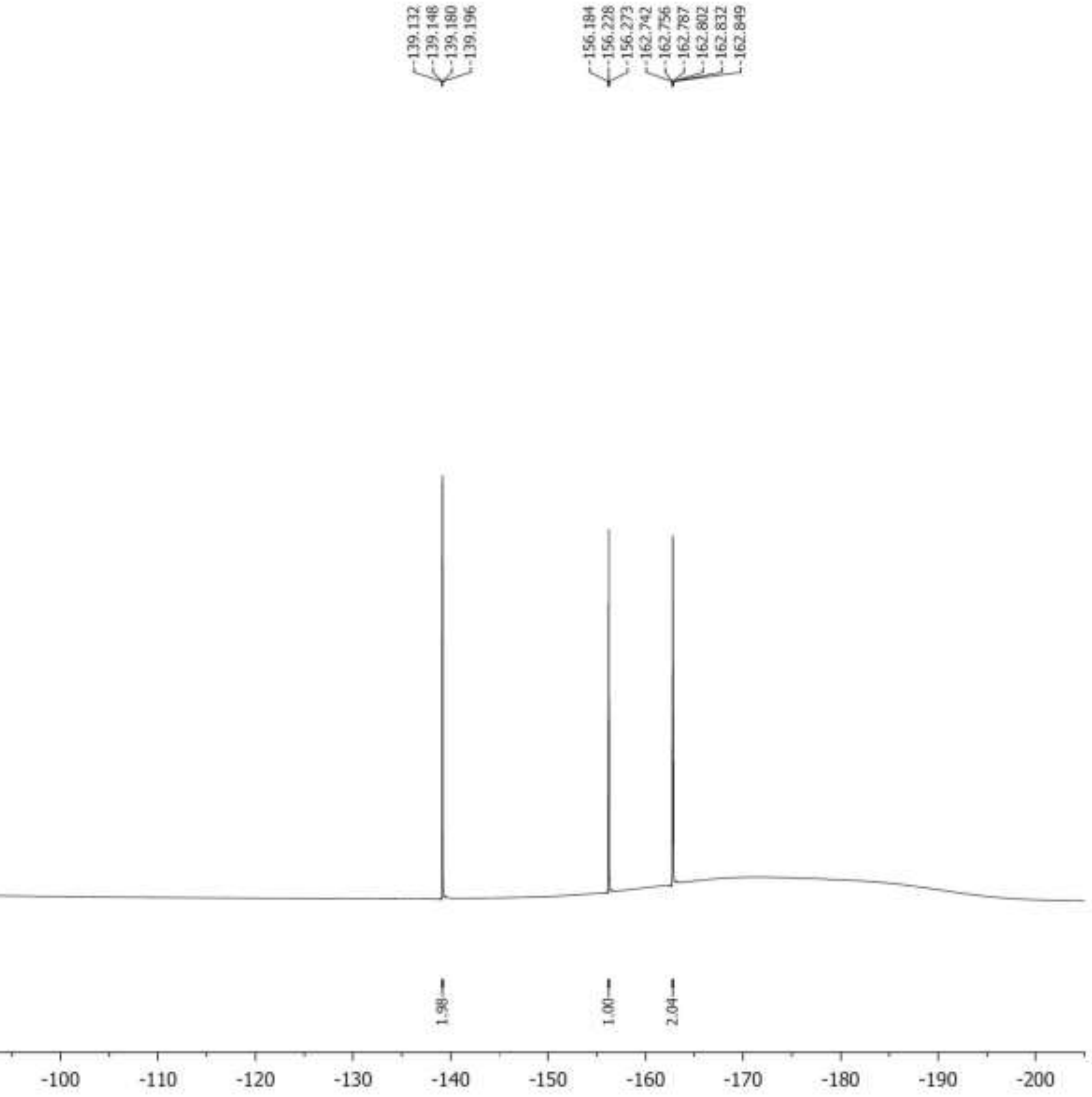

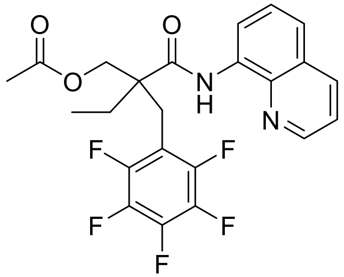

3q

^{1}H NMR (500 MHz, $CDCl_3$)

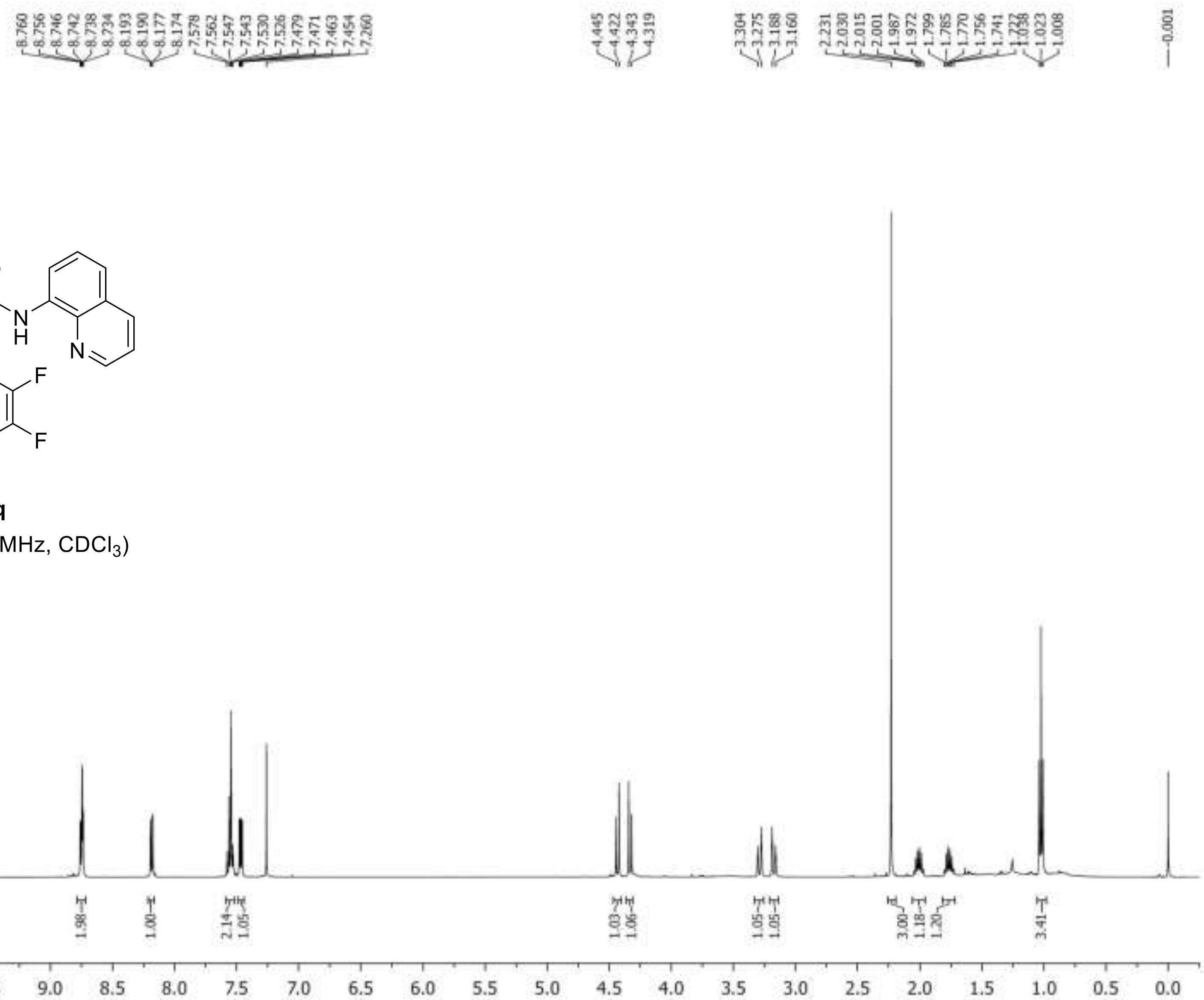

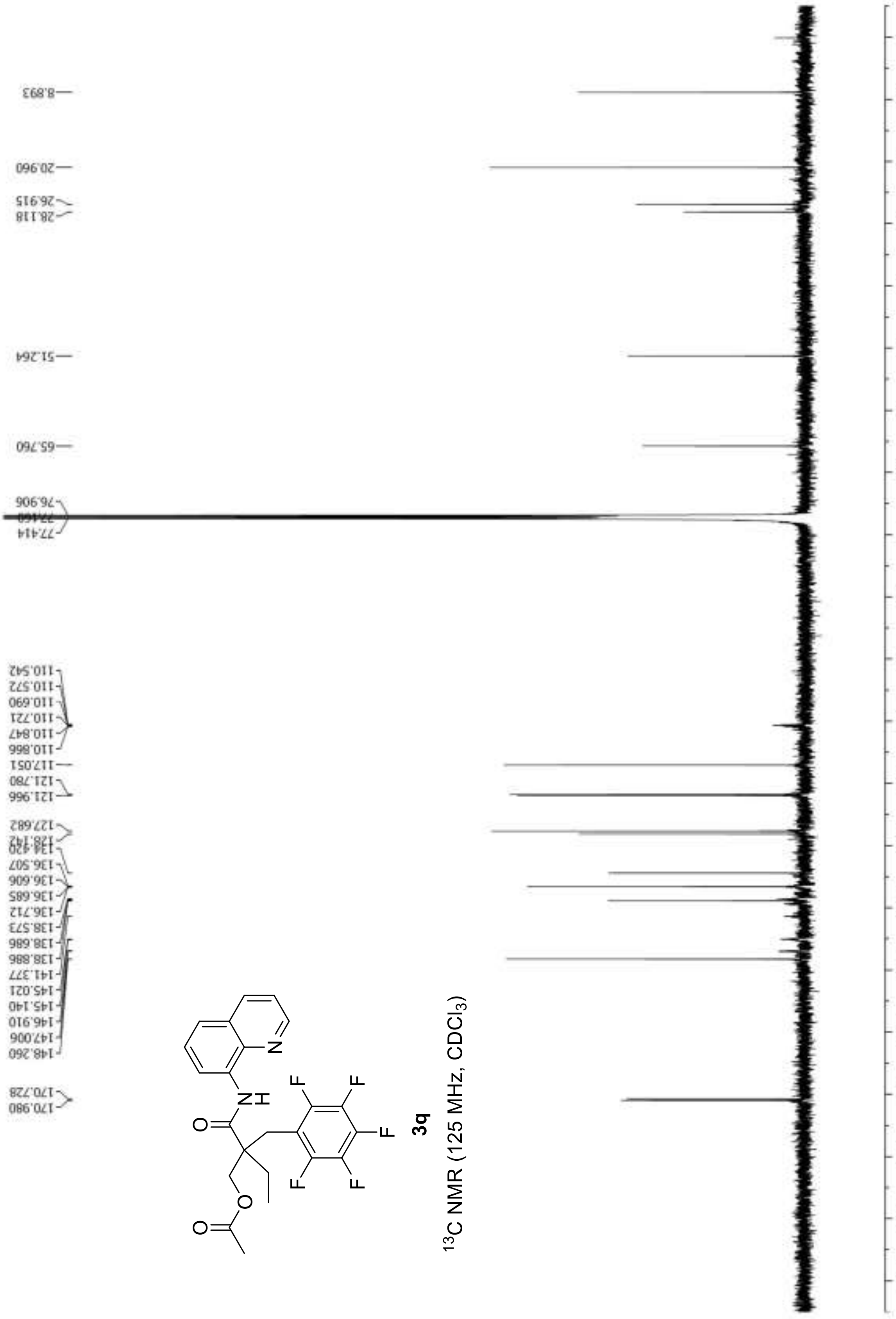

3q

^{13}C NMR (125 MHz, $CDCl_3$)

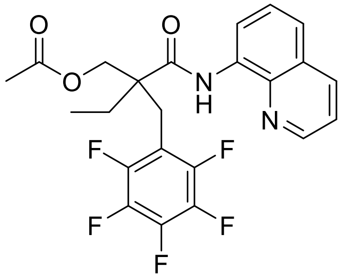

3q

^{19}F NMR (471 MHz, $CDCl_3$)

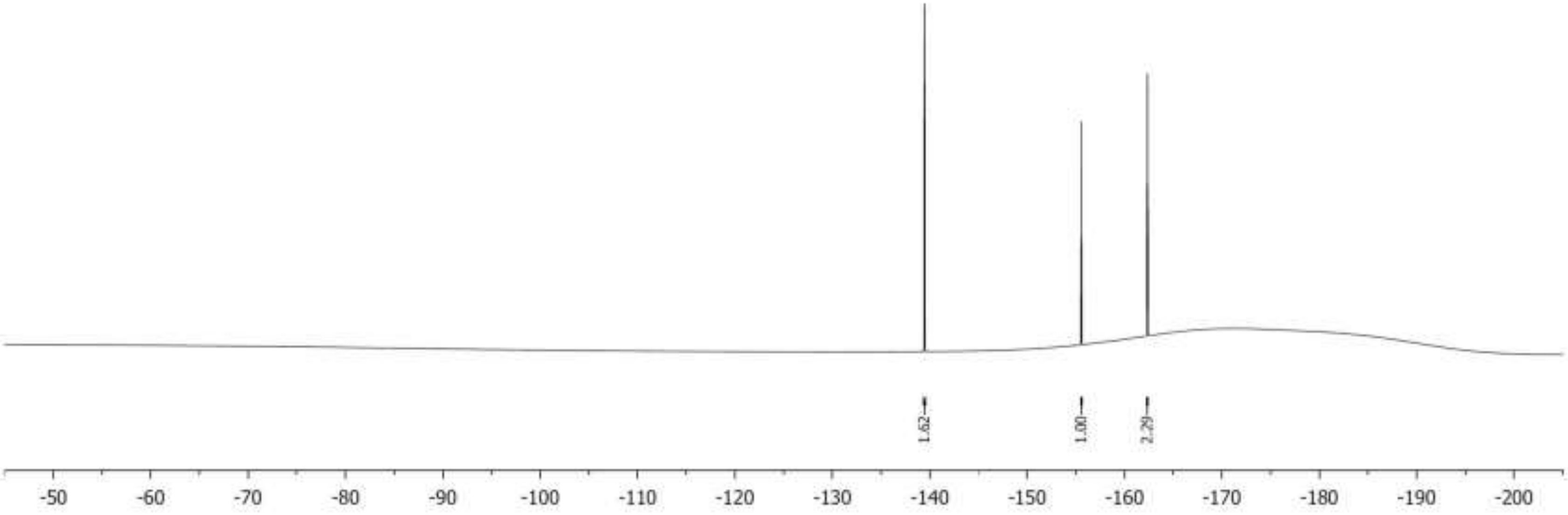

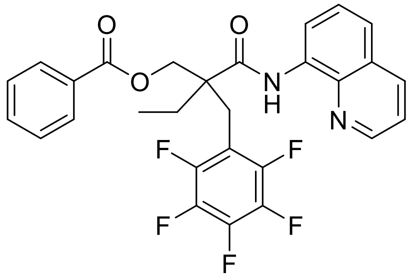

3r

^{1}H NMR (500 MHz, $CDCl_3$)

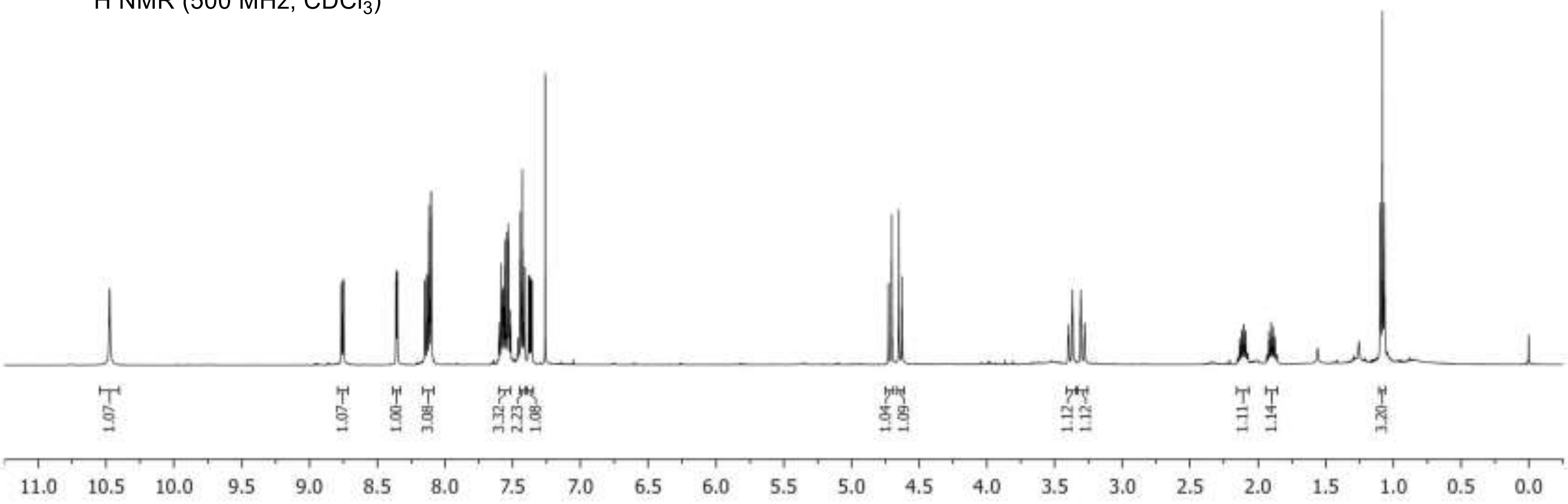

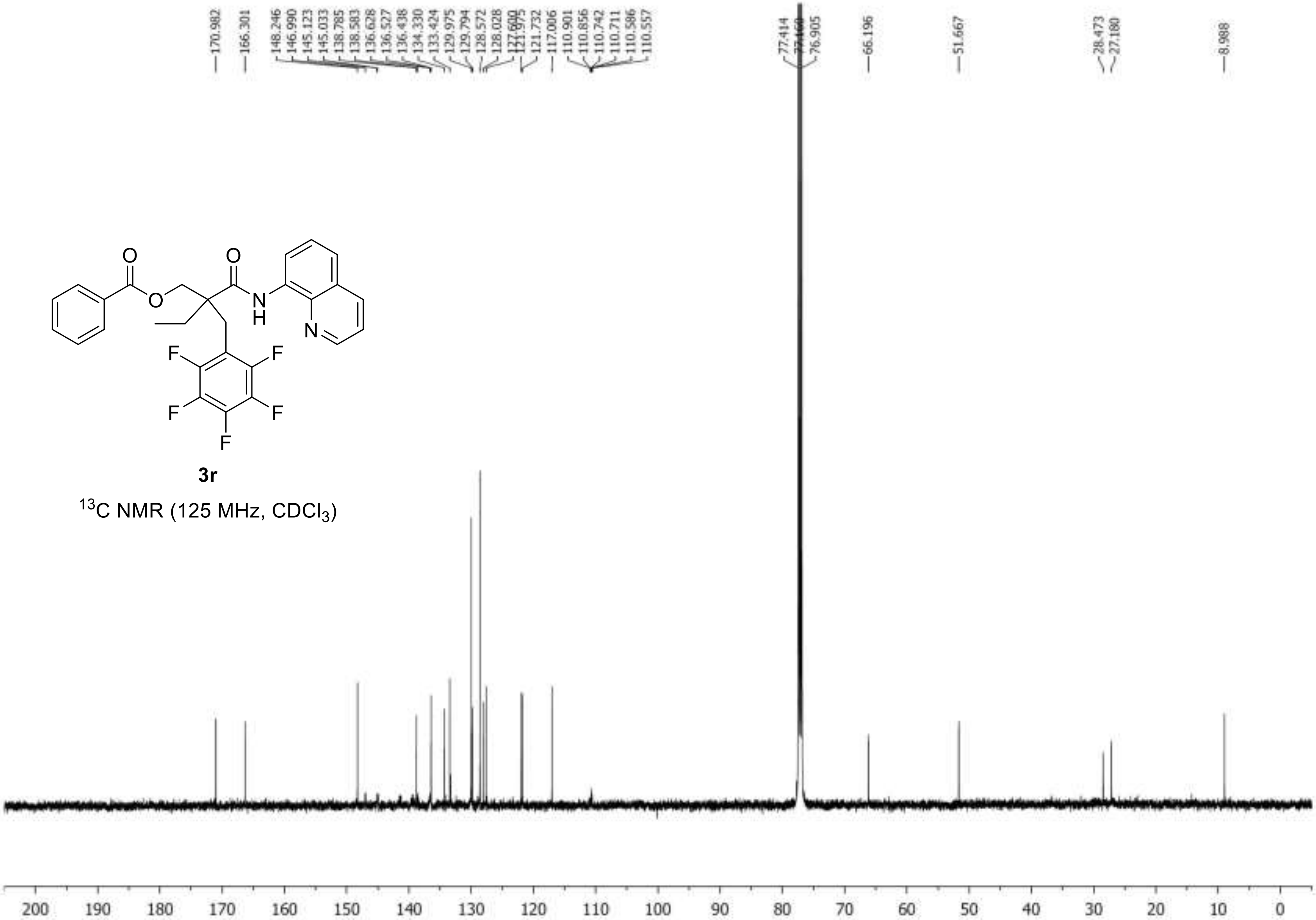

3r

^{13}C NMR (125 MHz, $CDCl_3$)

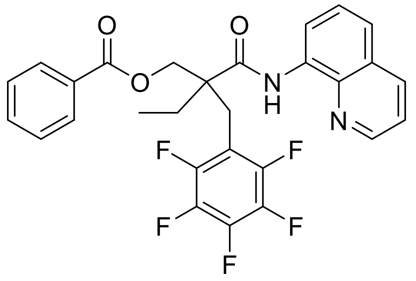

3r

^{19}F NMR (471 MHz, $CDCl_3$)

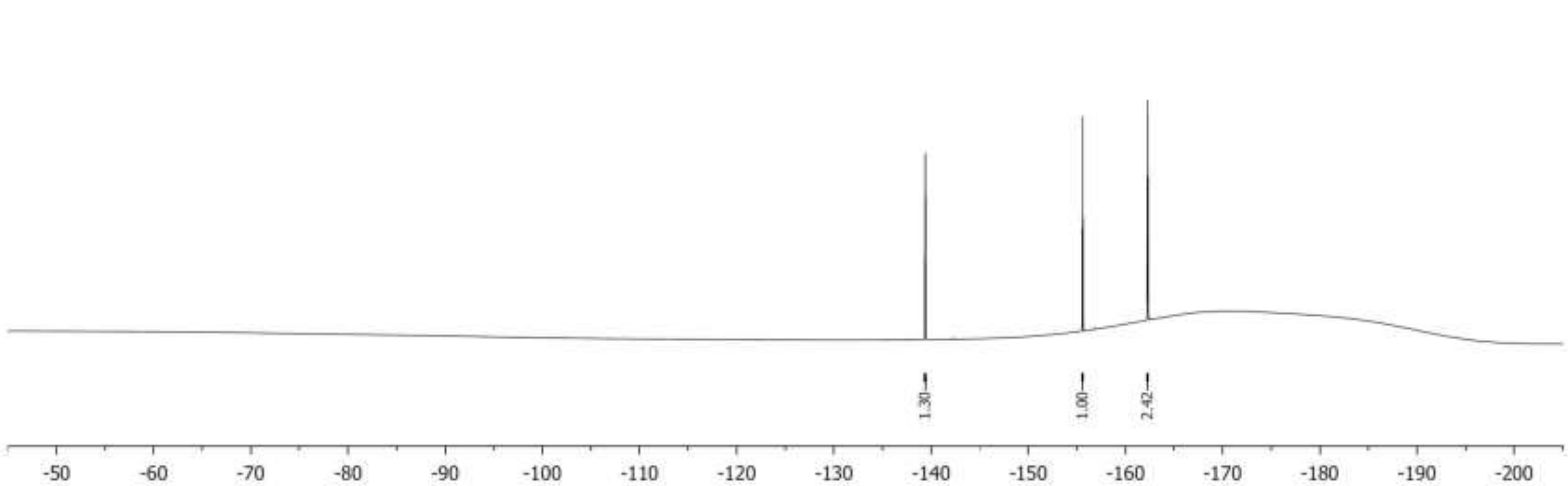

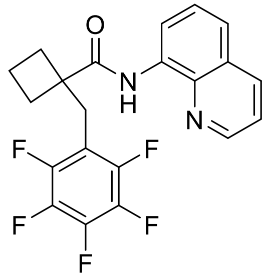

3s

^{1}H NMR (500 MHz, $CDCl_3$)

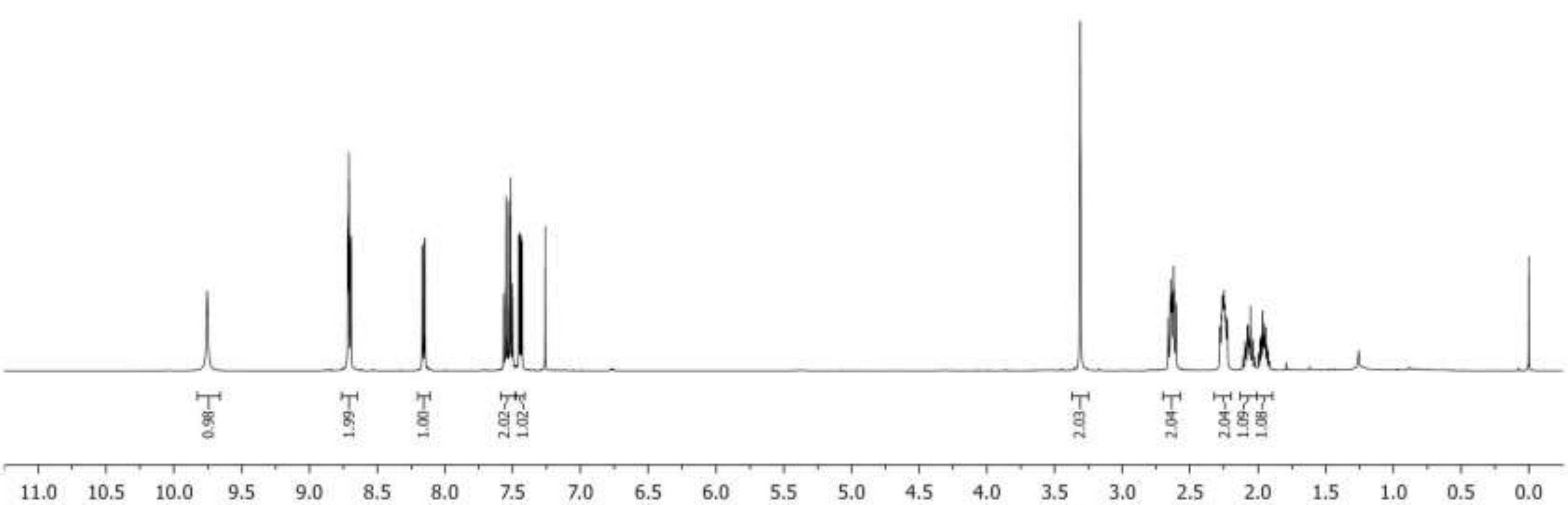

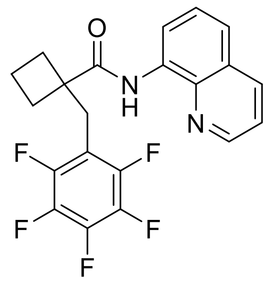

3s

^{13}C NMR (125 MHz, $CDCl_3$)

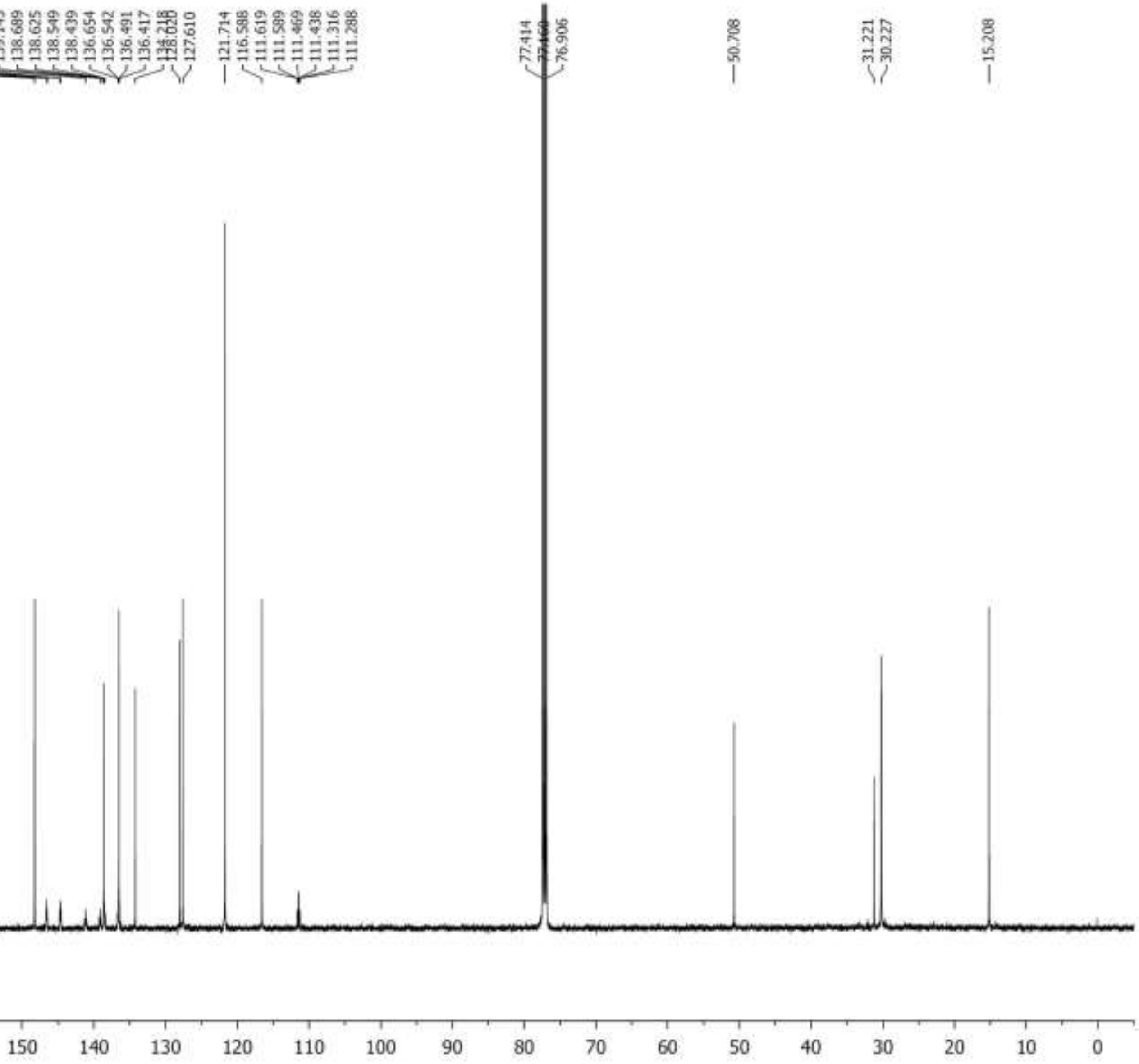

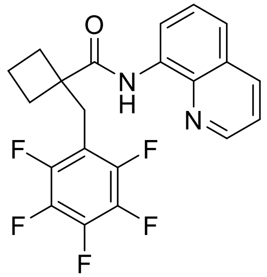

3s

^{19}F NMR (471 MHz, $CDCl_3$)

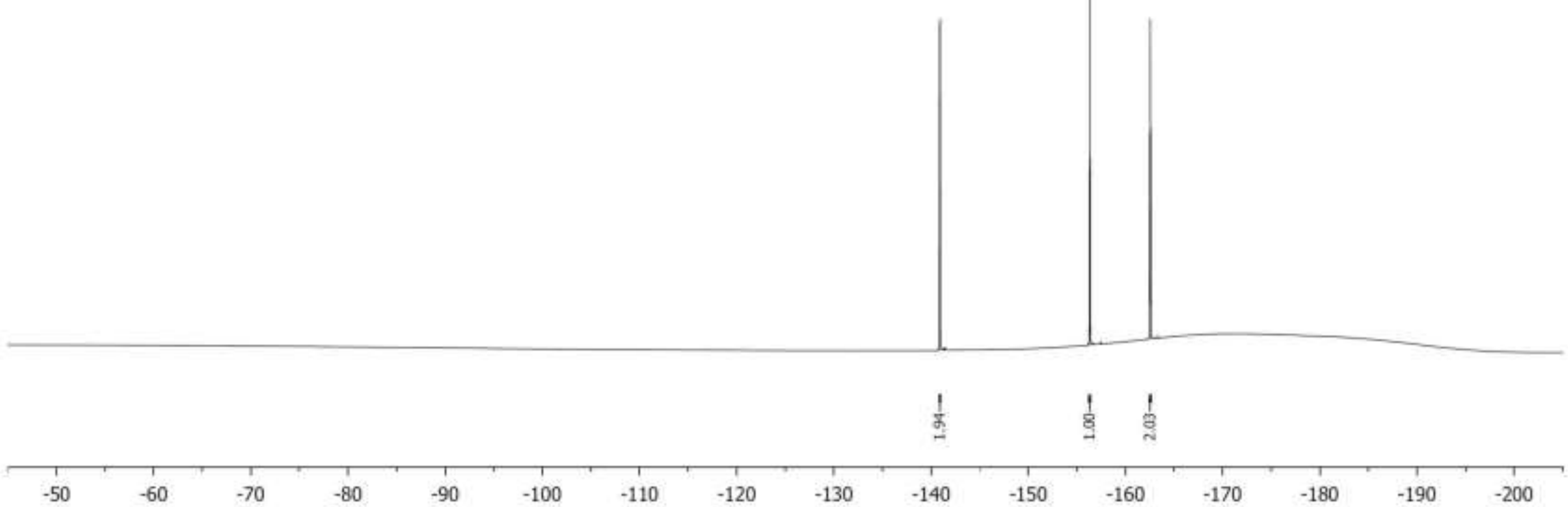

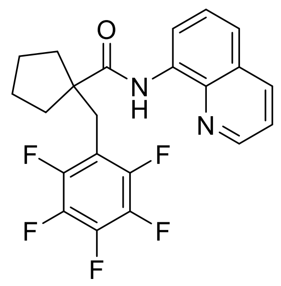

3t

^{1}H NMR (500 MHz, $CDCl_3$)

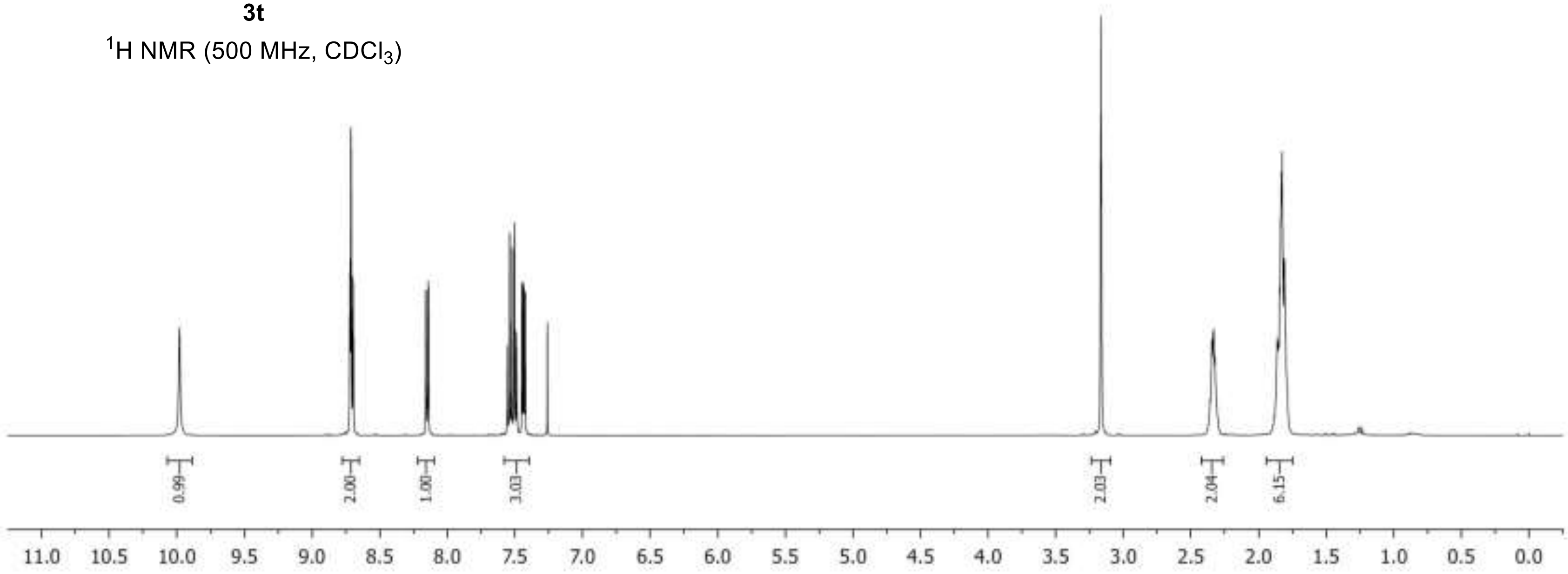

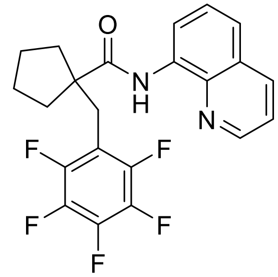

3t

^{13}C NMR (125 MHz, $CDCl_3$)

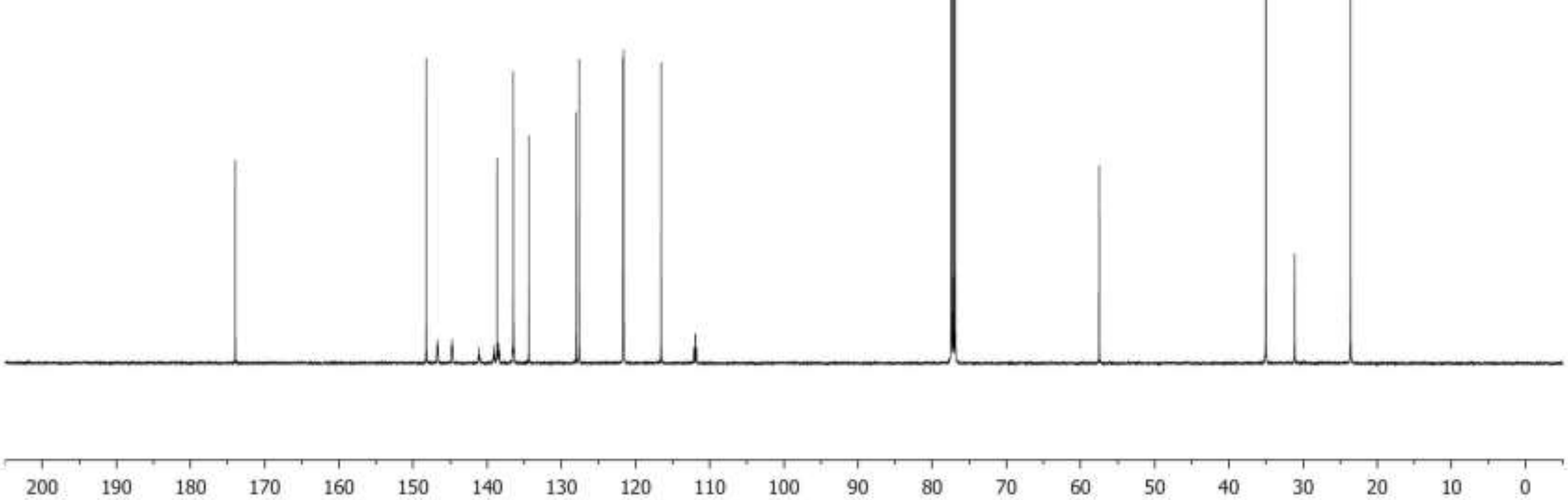

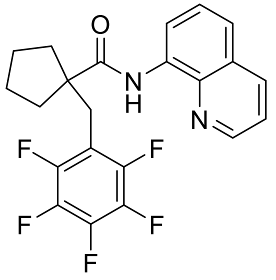

3t

^{19}F NMR (471 MHz, $CDCl_3$)

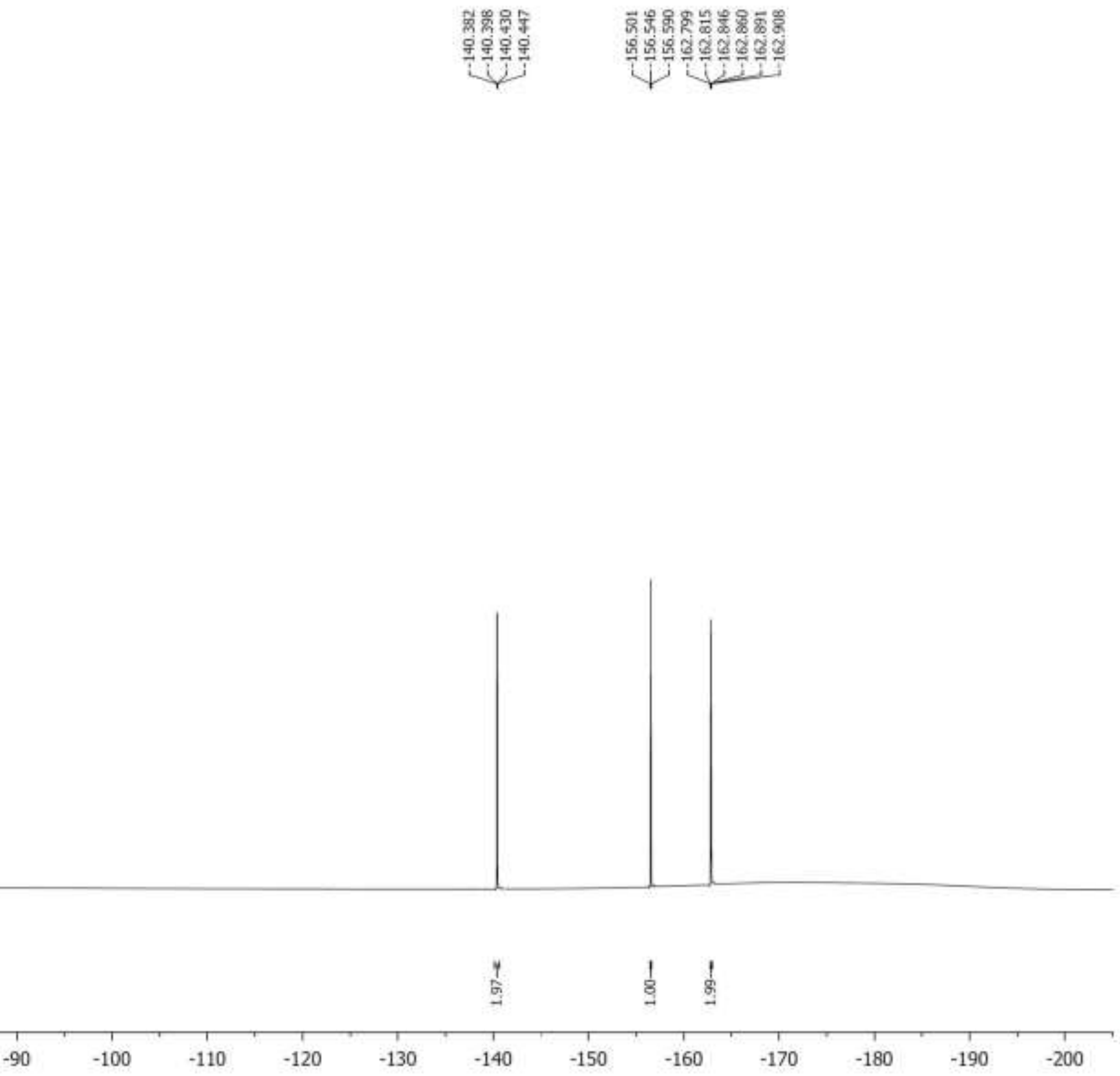

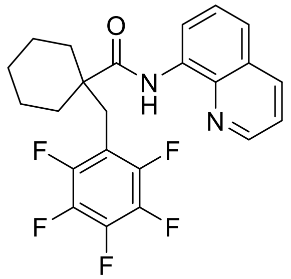

3u

^{1}H NMR (500 MHz, $CDCl_3$)

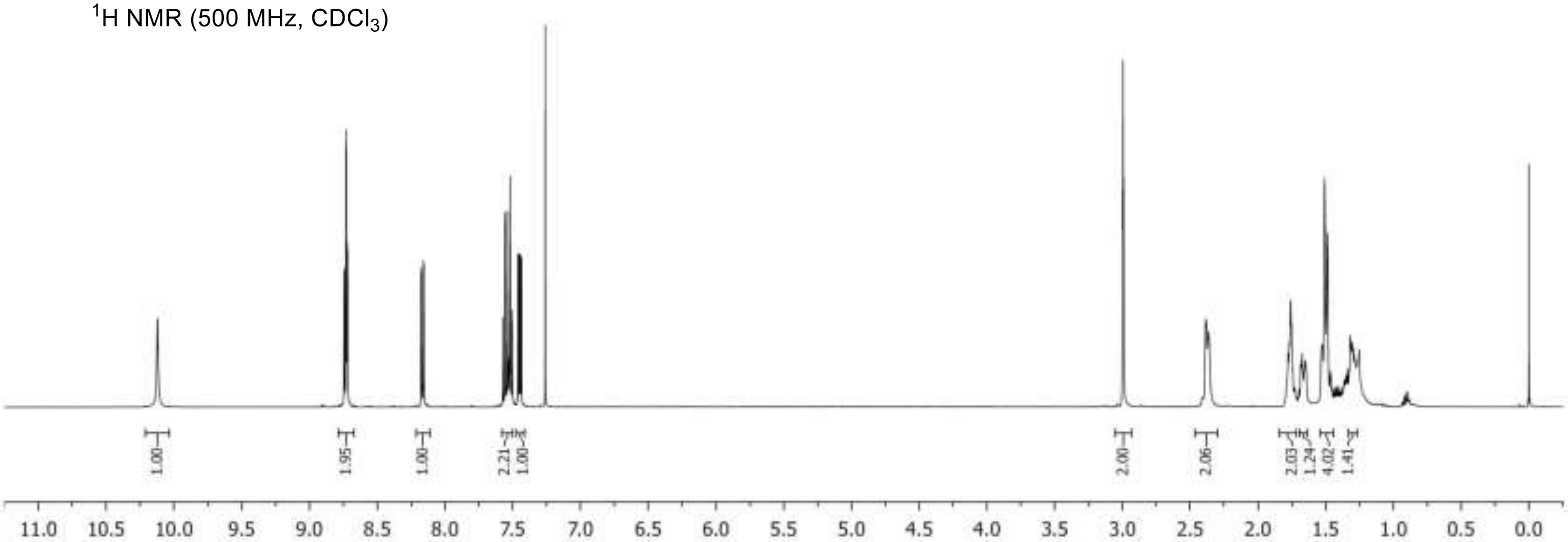

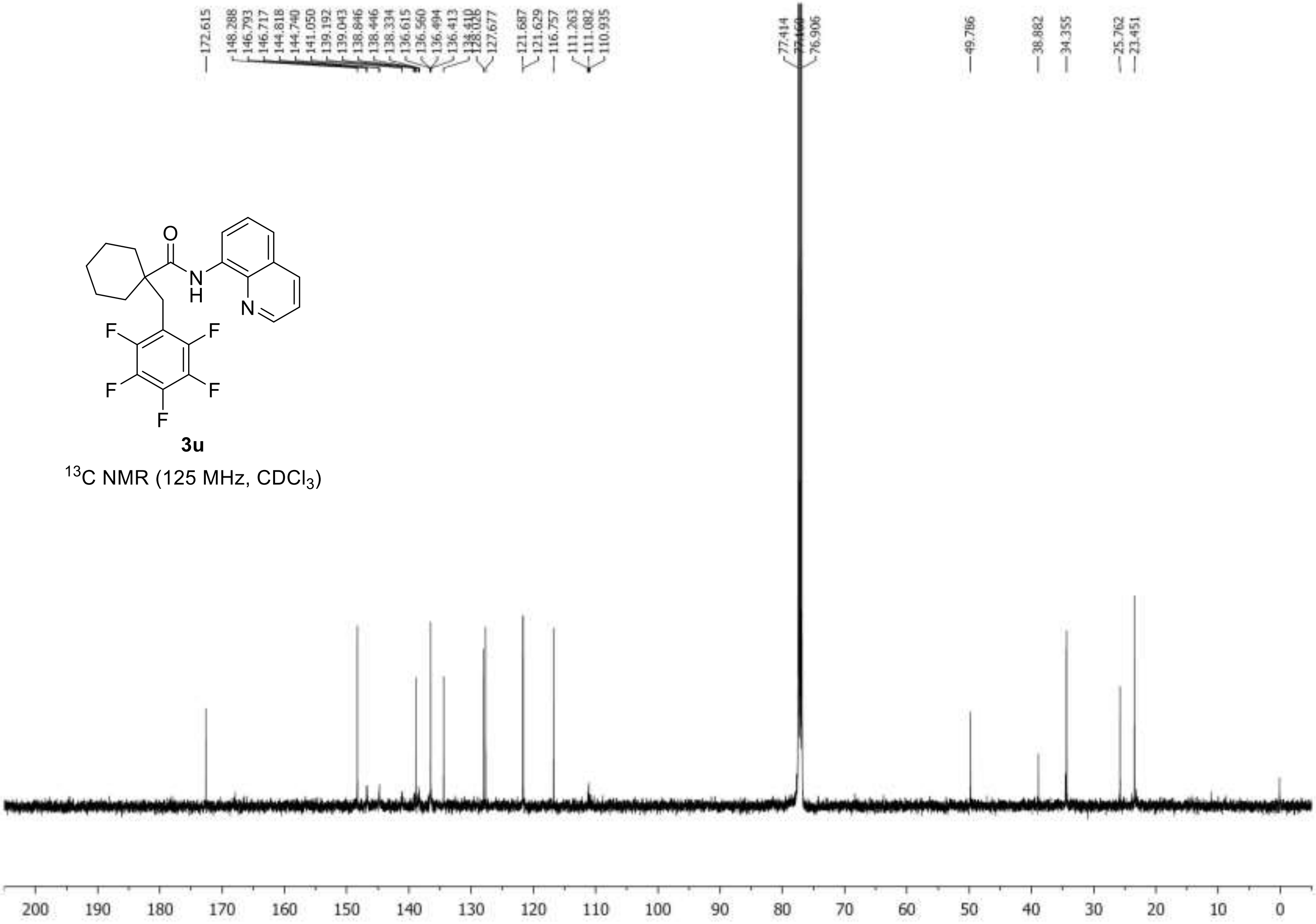

3u

^{13}C NMR (125 MHz, $CDCl_3$)

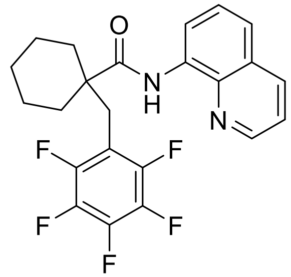

3u

^{19}F NMR (471 MHz, $CDCl_3$)

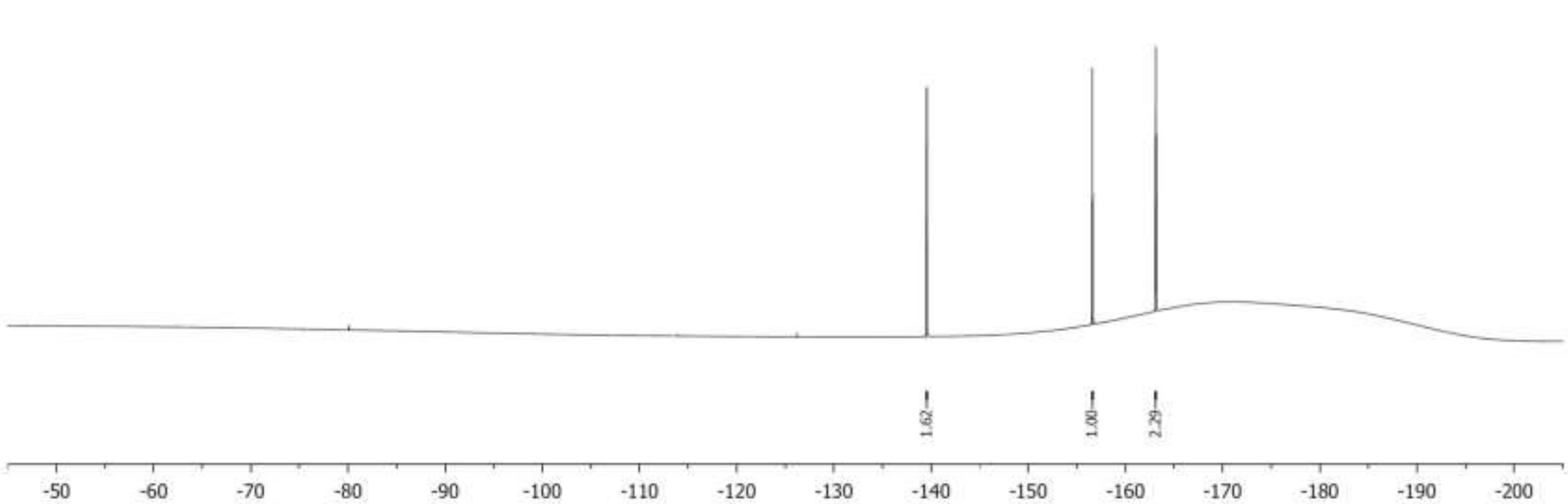

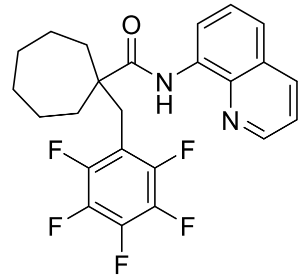

3v

^1H NMR (500 MHz, $CDCl_3$)

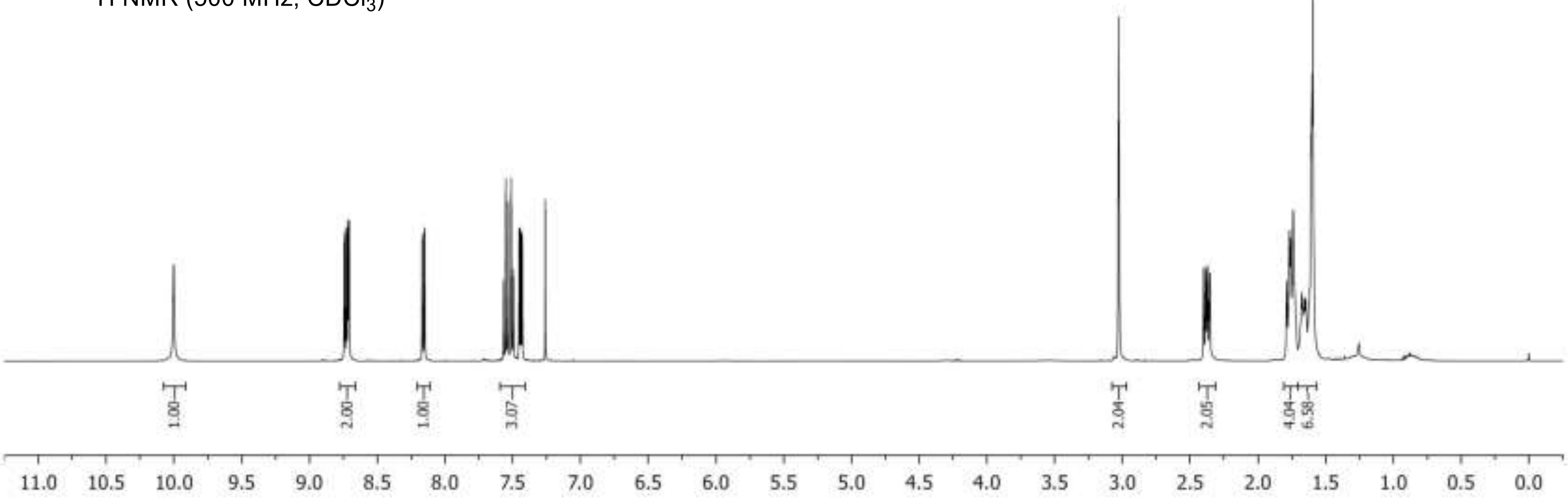

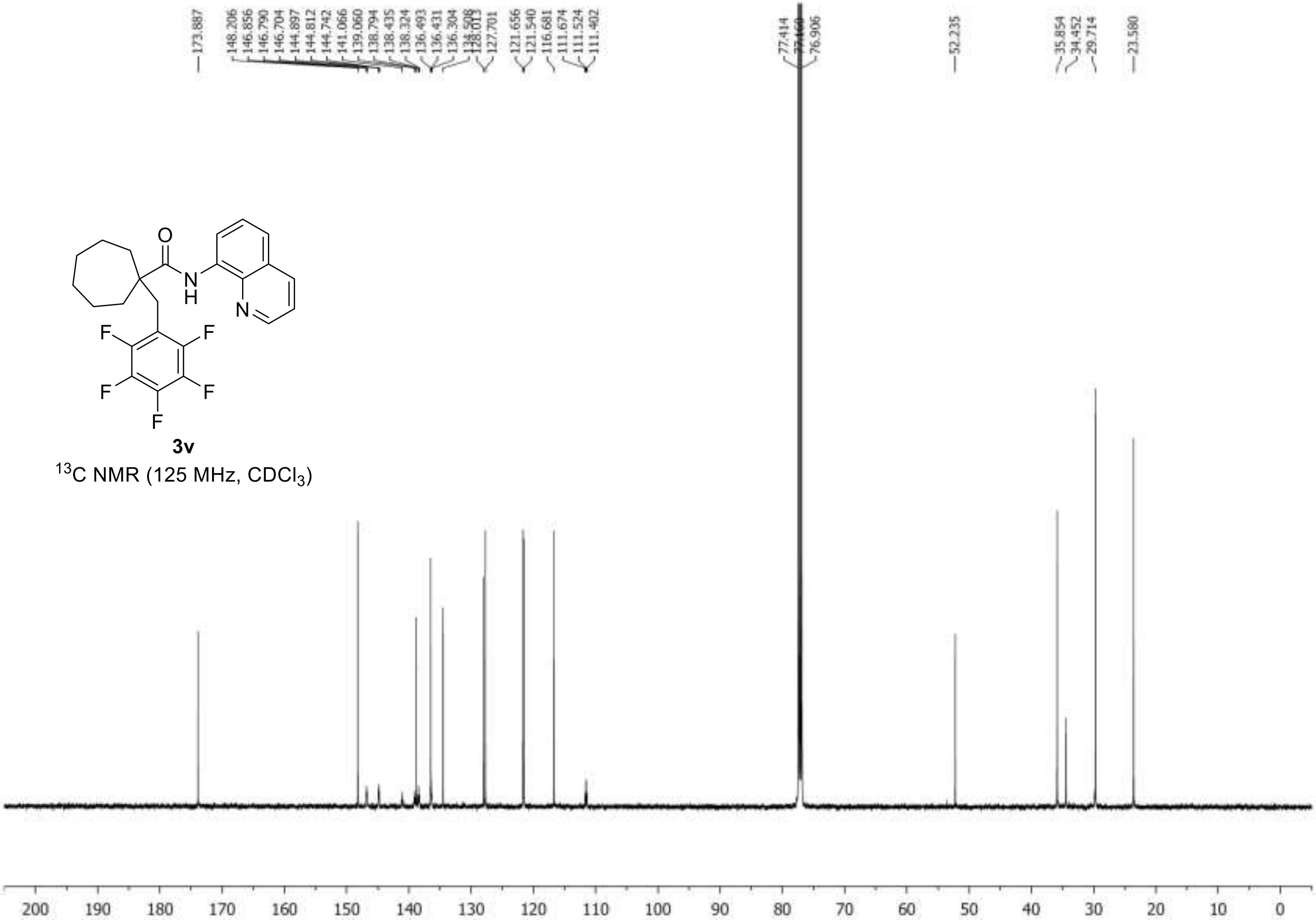

3v

^{13}C NMR (125 MHz, $CDCl_3$)

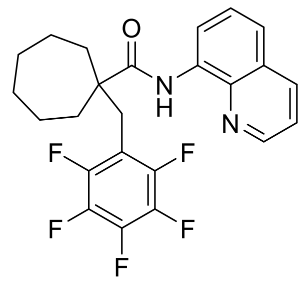

3v

^{19}F NMR (471 MHz, $CDCl_3$)

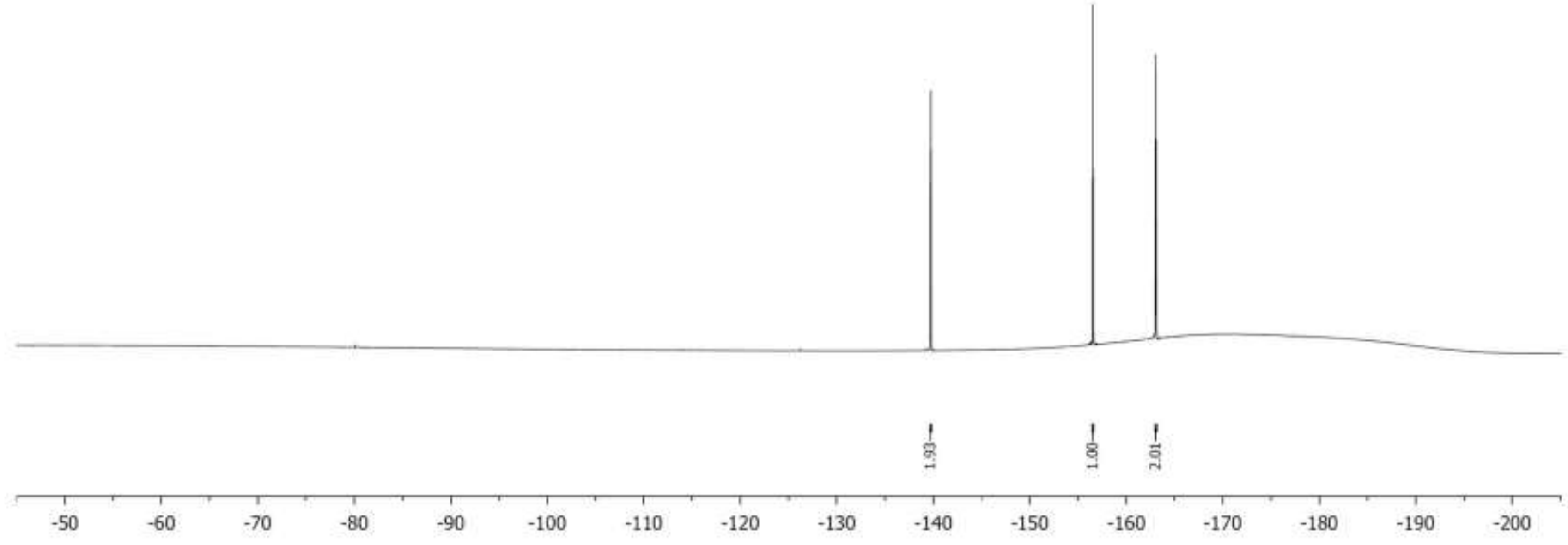

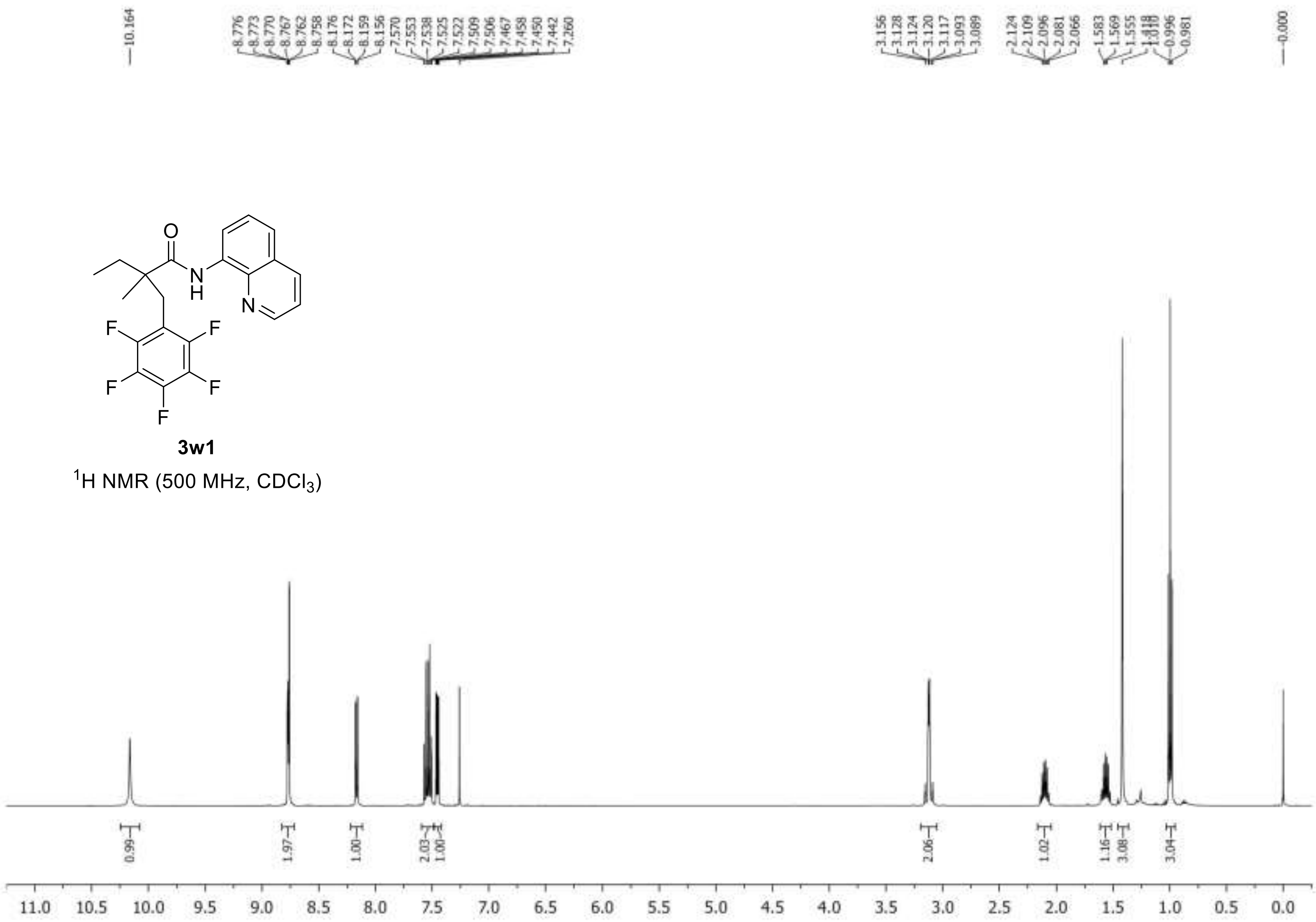

3w1

^{1}H NMR (500 MHz, $CDCl_3$)

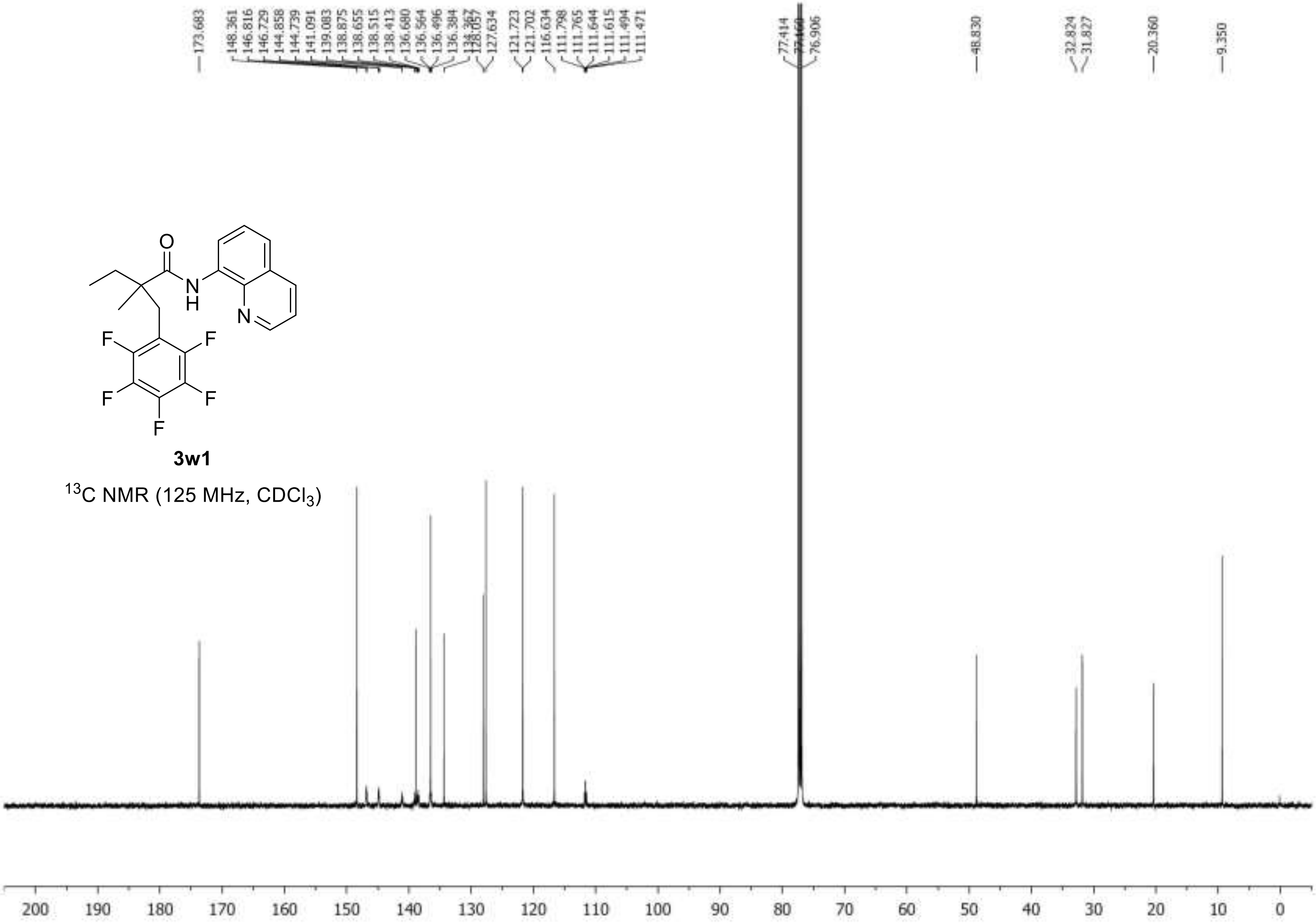

3w1

^{13}C NMR (125 MHz, $CDCl_3$)

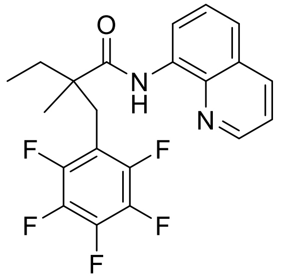

3w1

^{19}F NMR (471 MHz, CDCl$_3$)

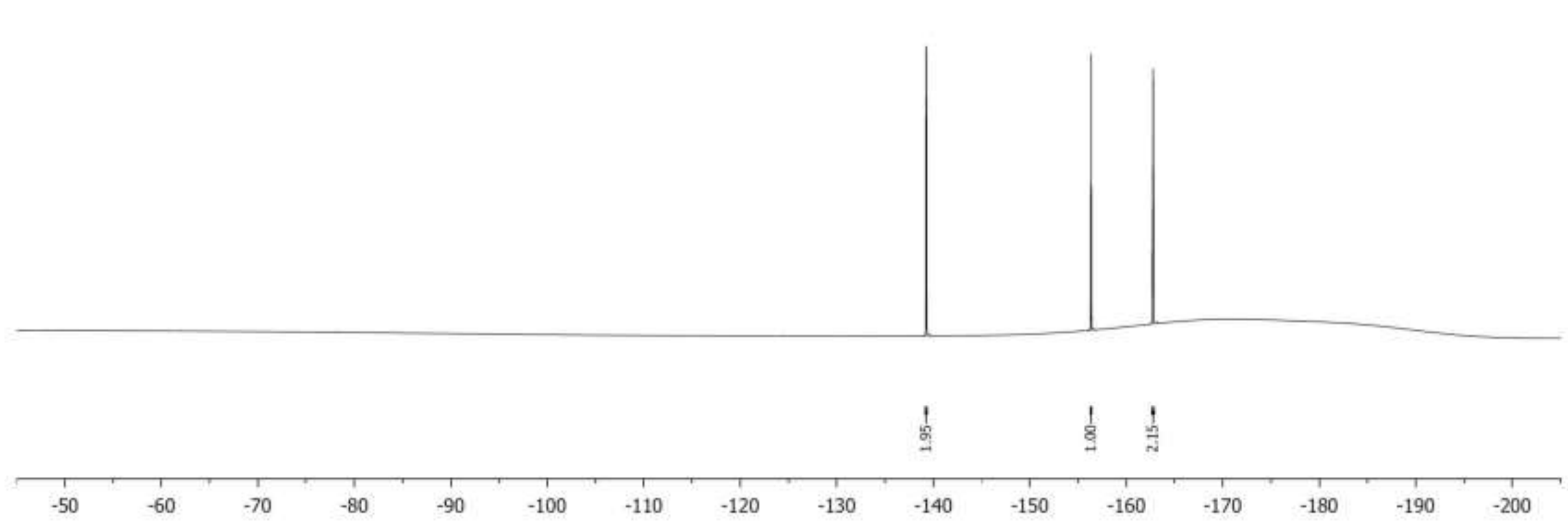

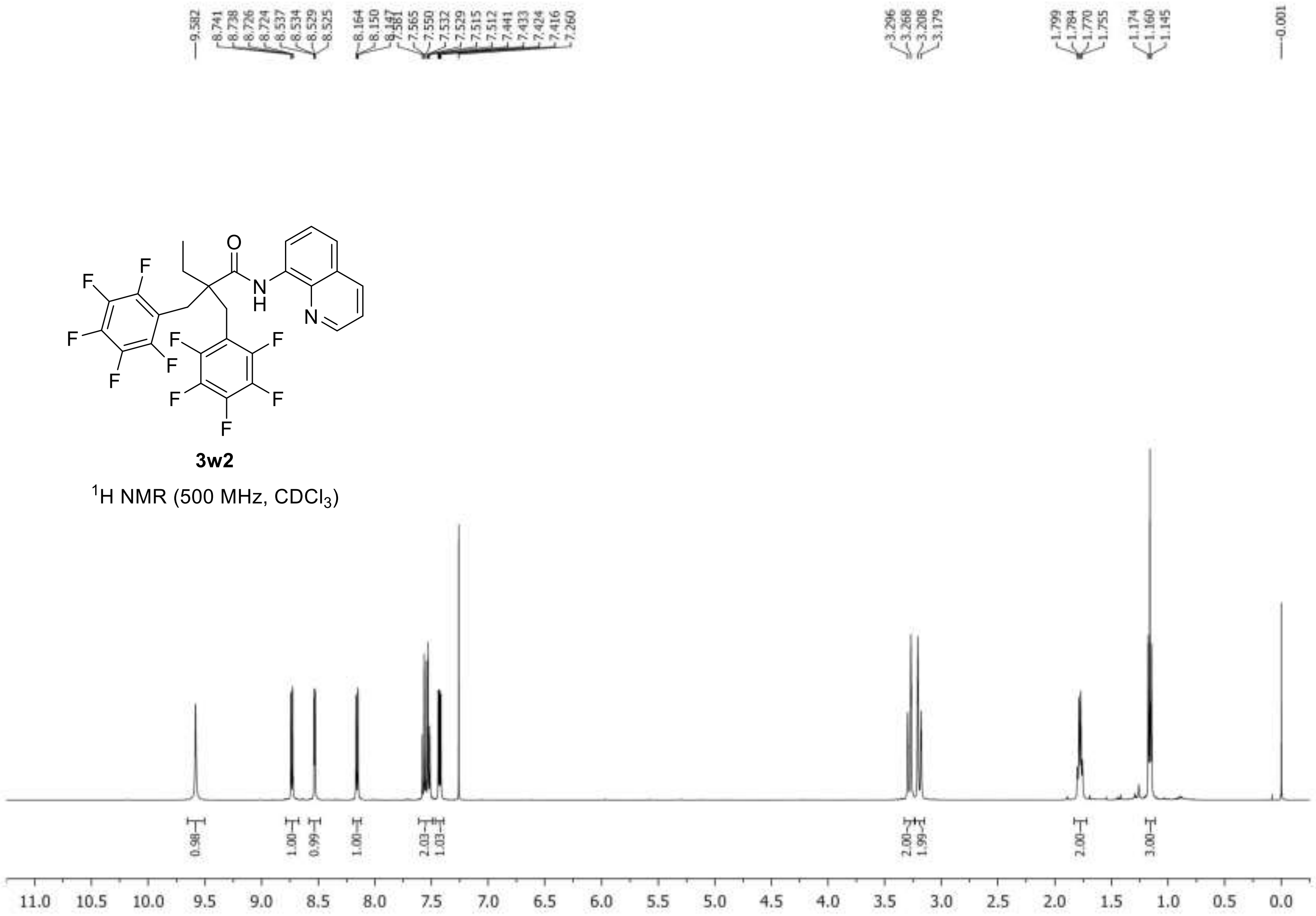

3w2

^{1}H NMR (500 MHz, $CDCl_3$)

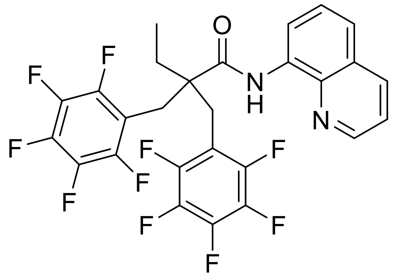

3w2

^{13}C NMR (125 MHz, $CDCl_3$)

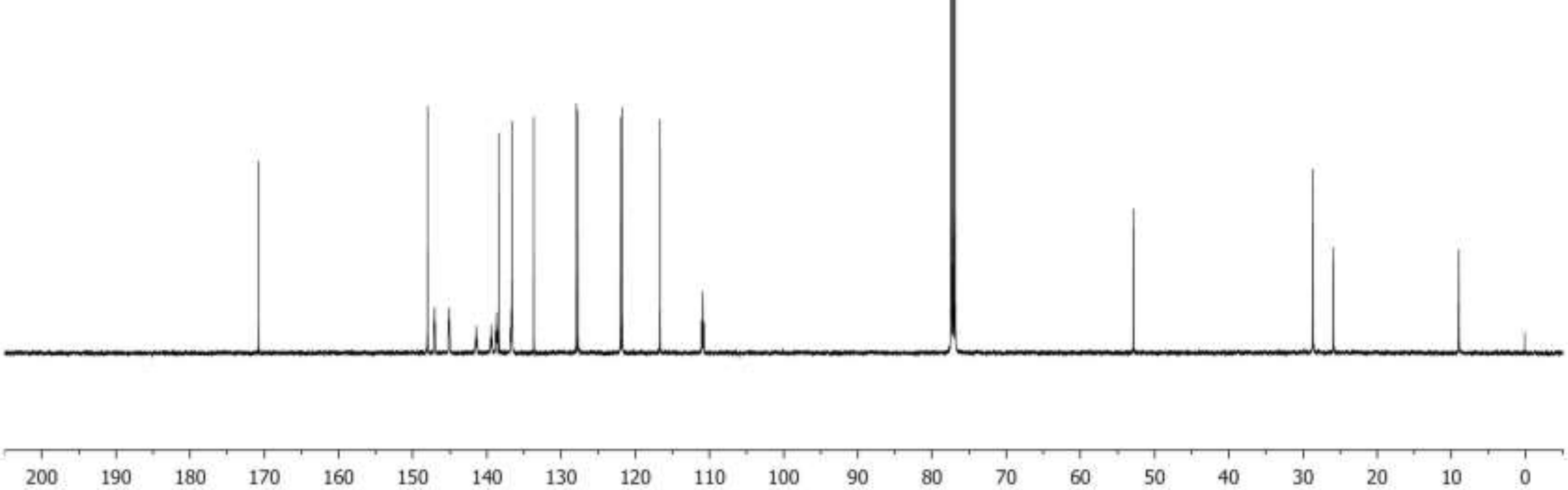

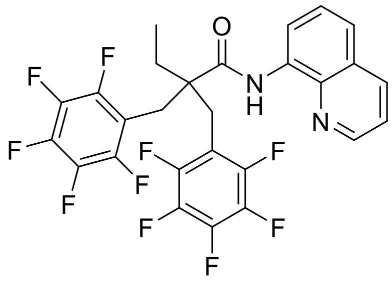

3w2

^{19}F NMR (471 MHz, $CDCl_3$)

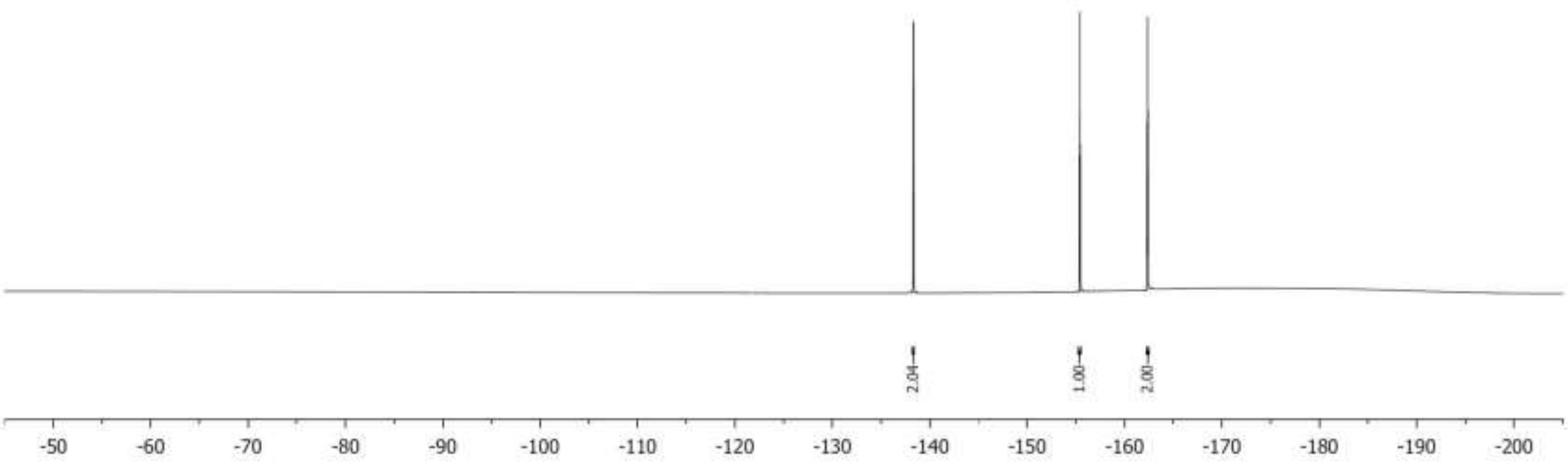

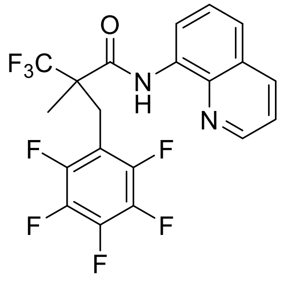

3x1

^{1}H NMR (500 MHz, $CDCl_3$)

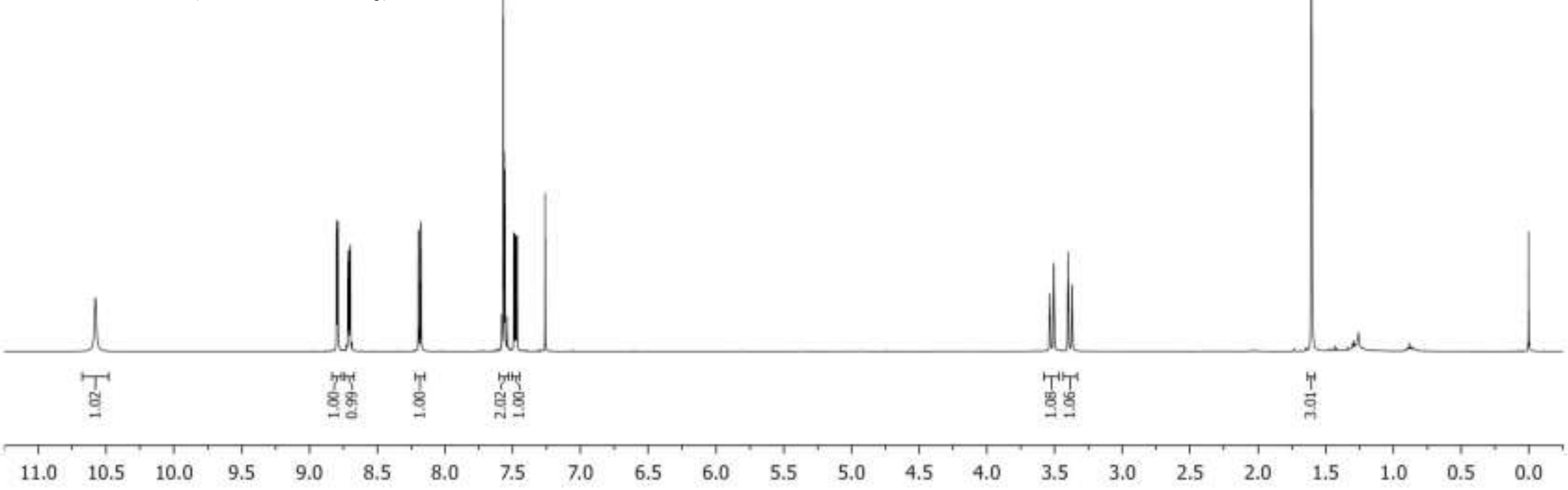

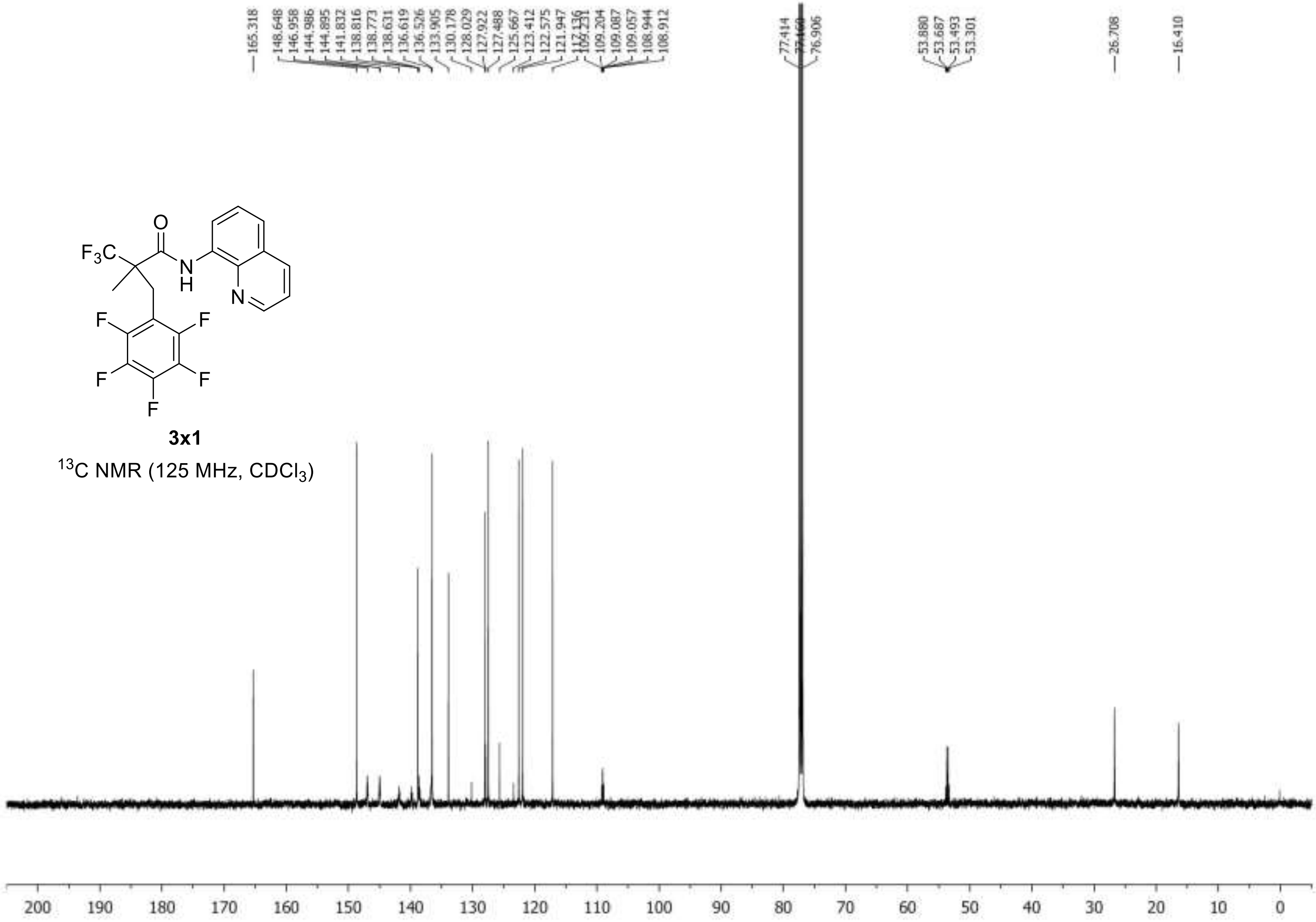

3x1

^{13}C NMR (125 MHz, $CDCl_3$)

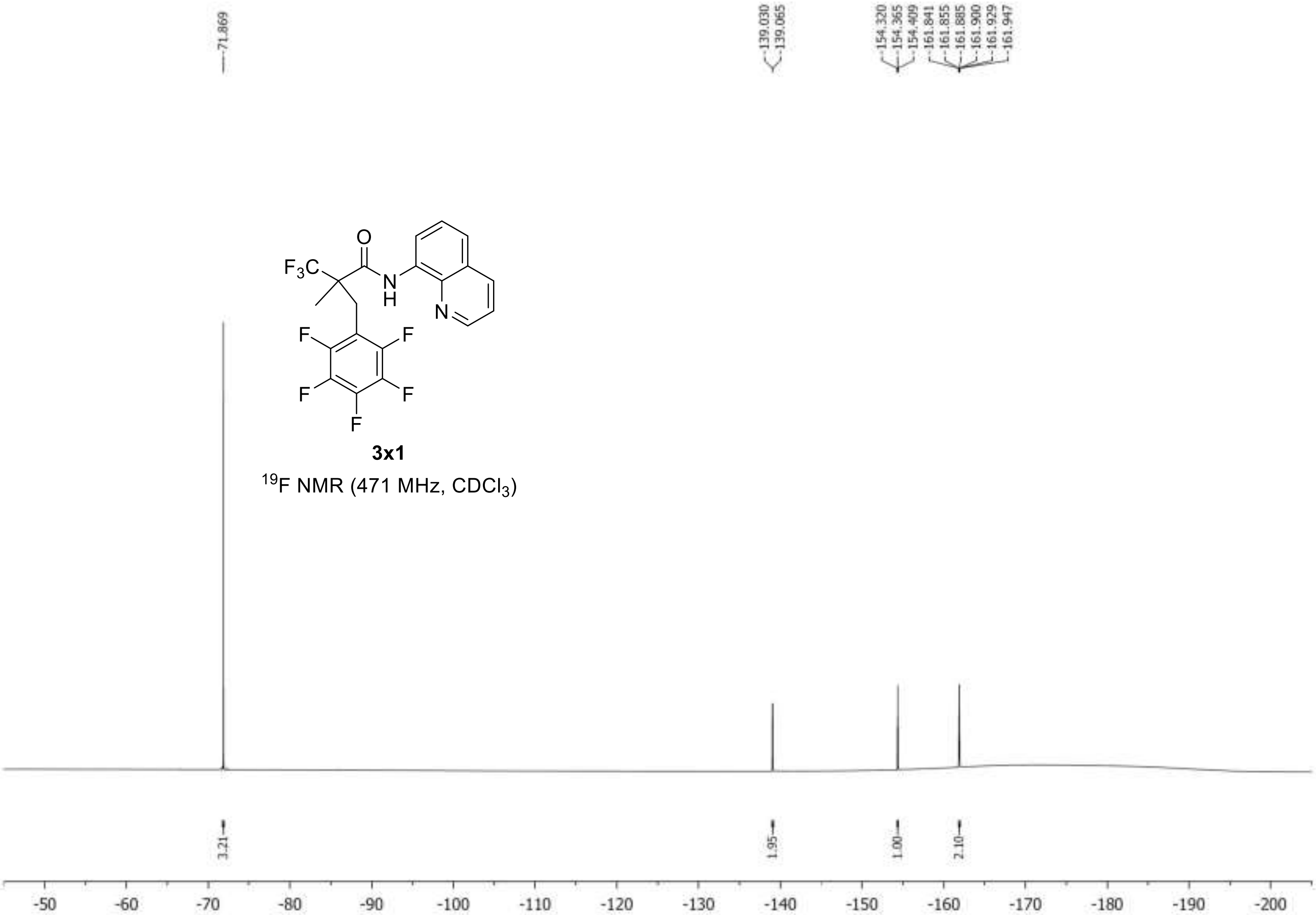

3x1

^{19}F NMR (471 MHz, $CDCl_3$)

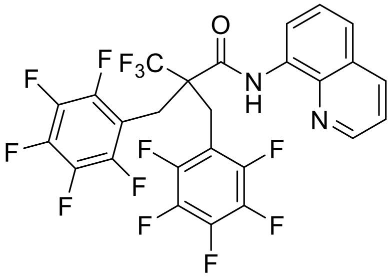

3x2

^{1}H NMR (500 MHz, $CDCl_3$)

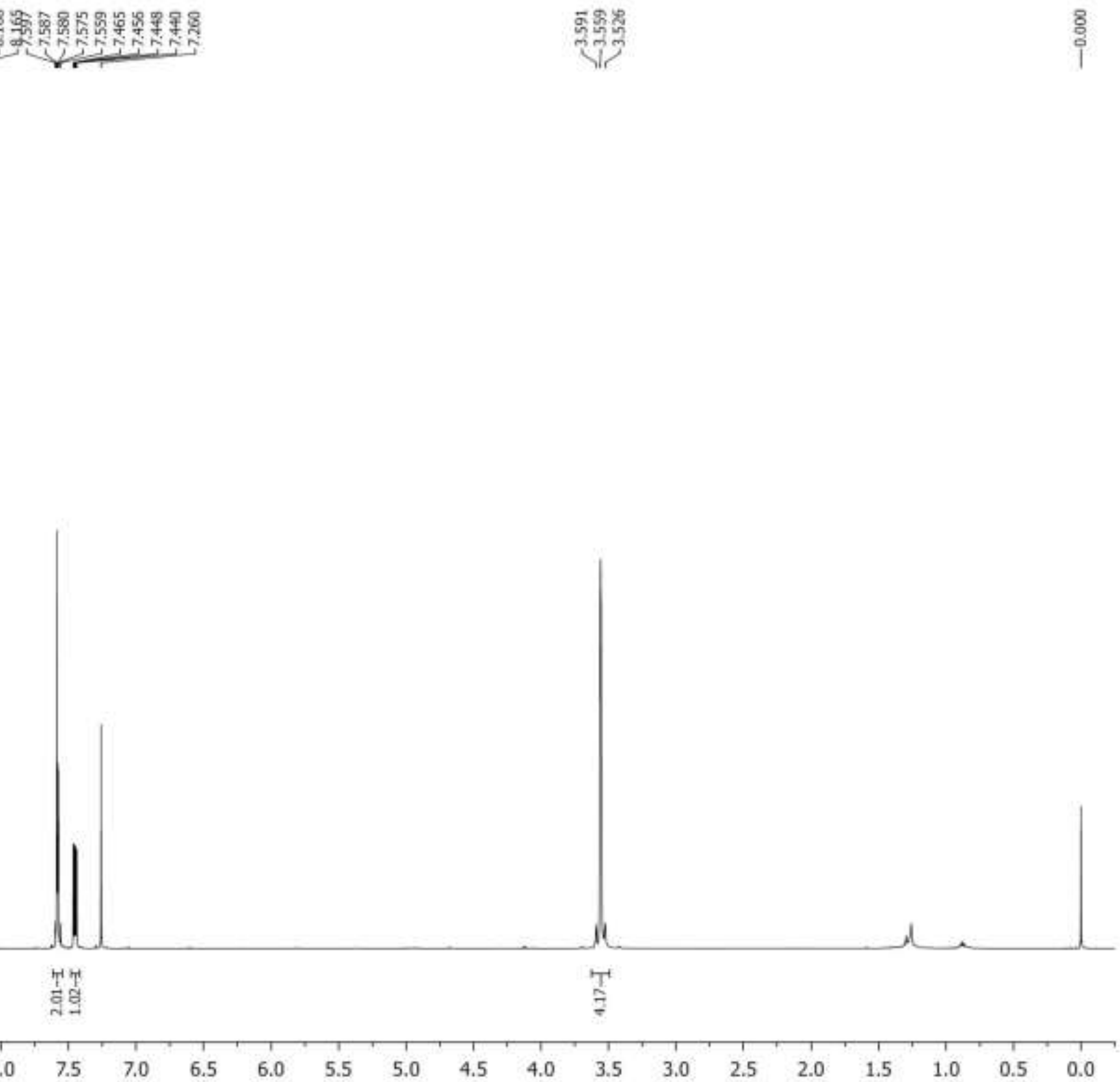

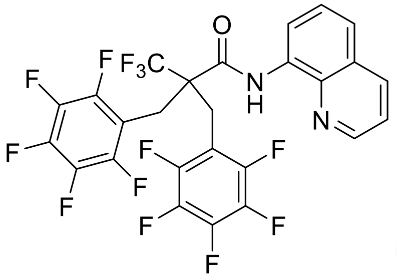

3x2

^{13}C NMR (125 MHz, $CDCl_3$)

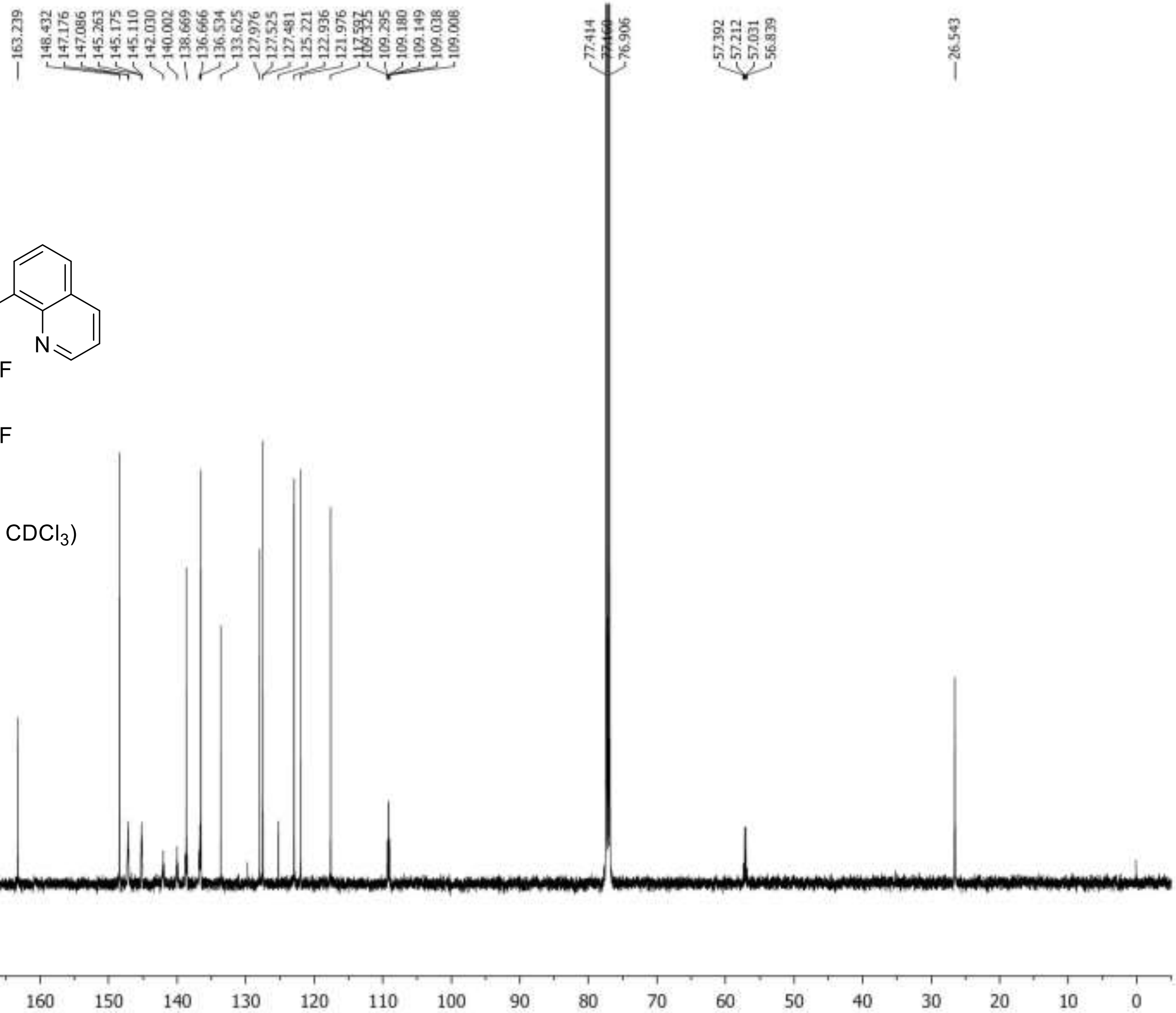

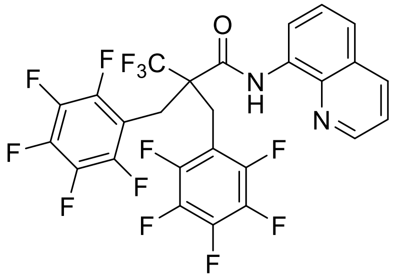

3x2

^{19}F NMR (471 MHz, $CDCl_3$)

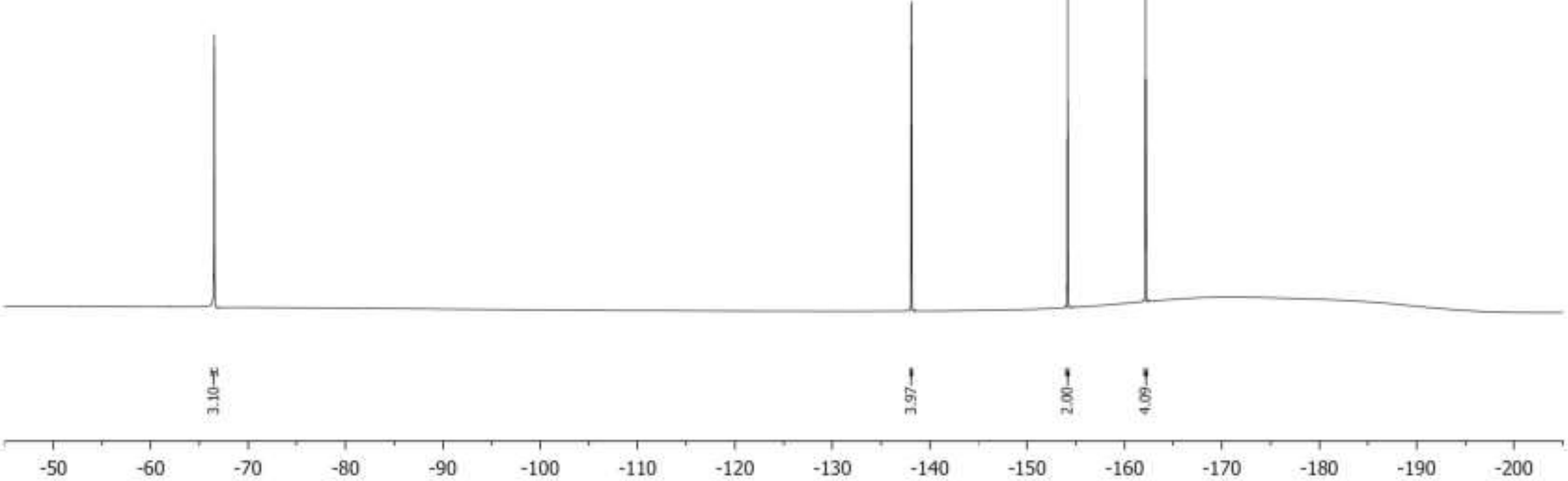

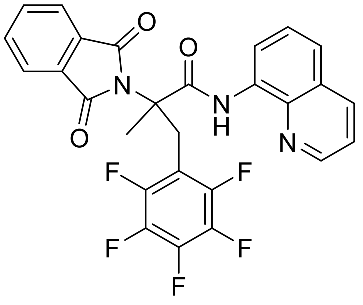

3y

^{1}H NMR (500 MHz, $CDCl_3$)

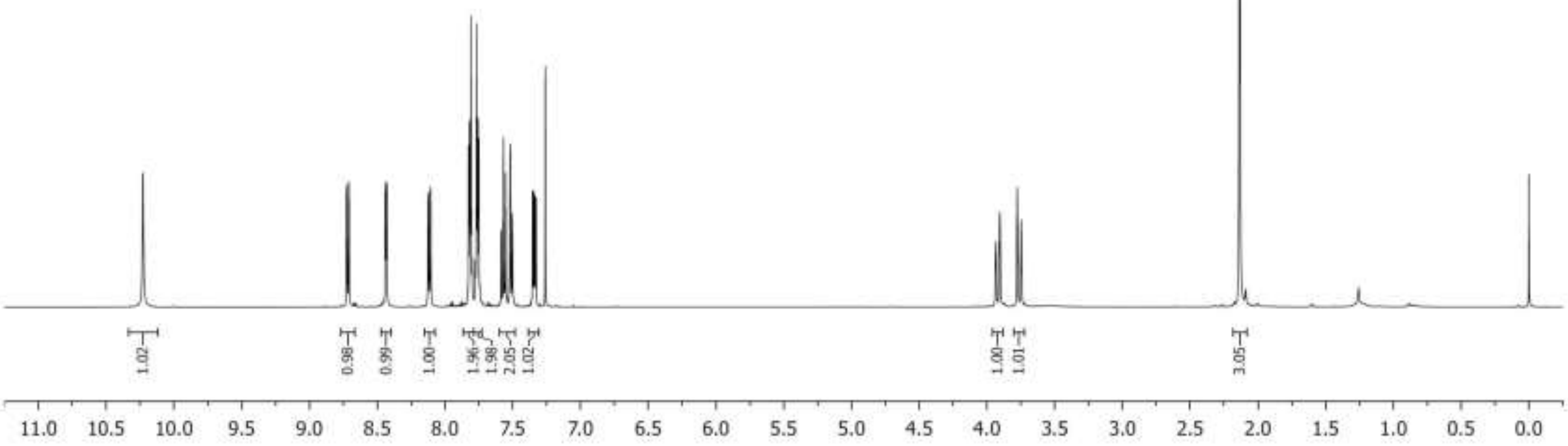

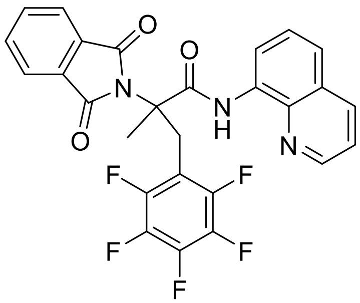

3y

^{13}C NMR (125 MHz, $CDCl_3$)

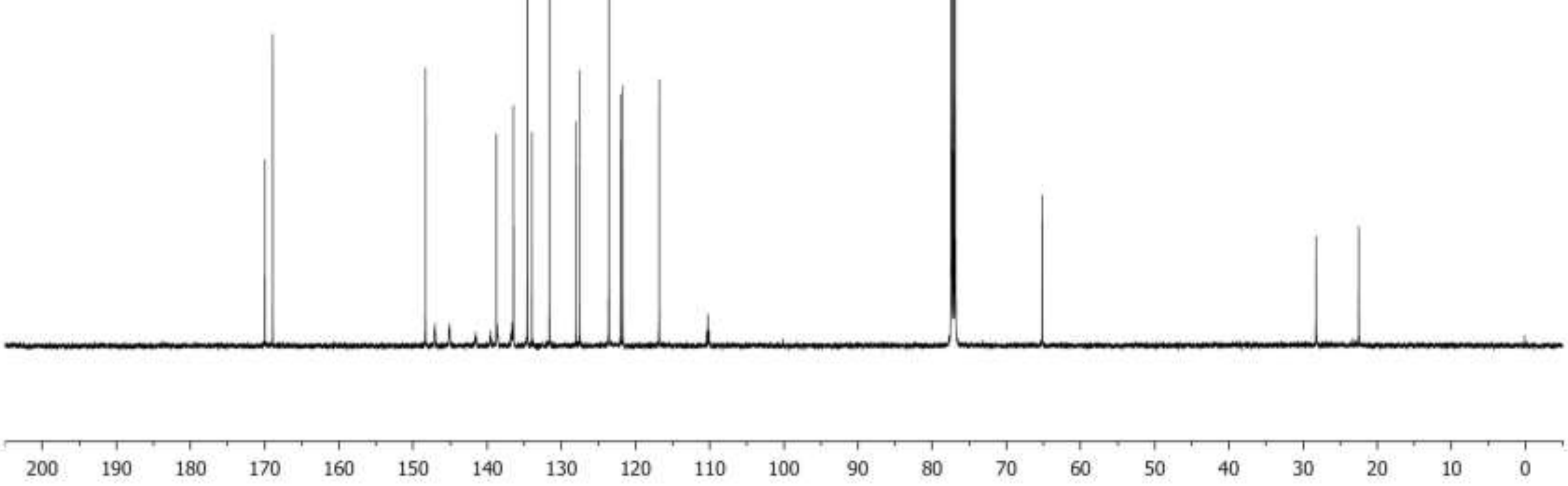

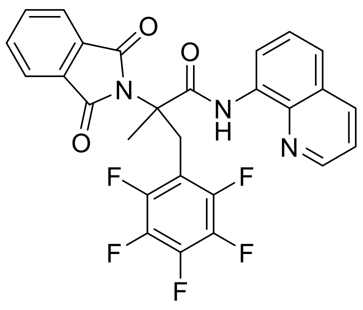

3y

^{19}F NMR (471 MHz, $CDCl_3$)

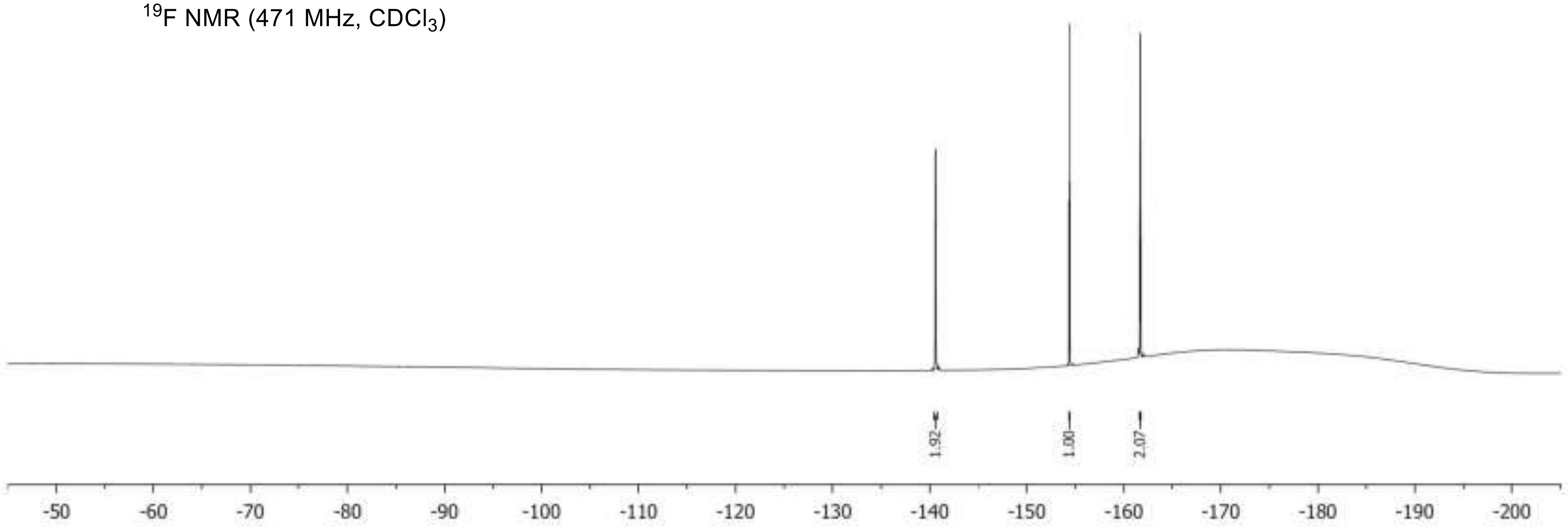

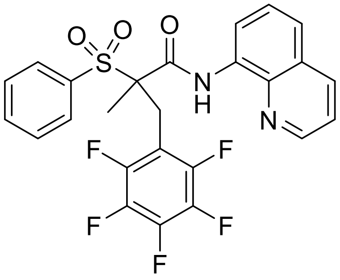

3z

^1H NMR (500 MHz, $CDCl_3$)

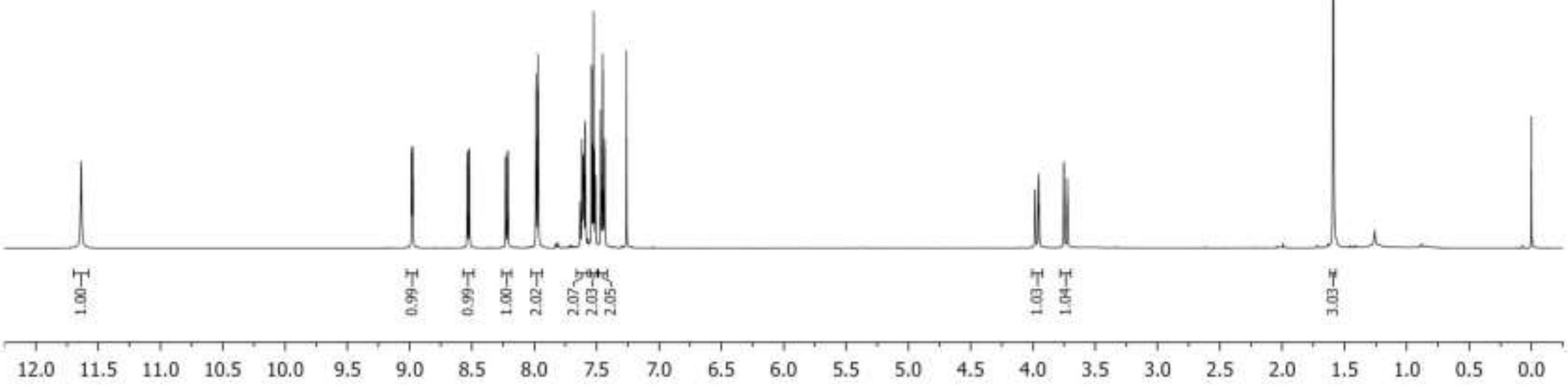

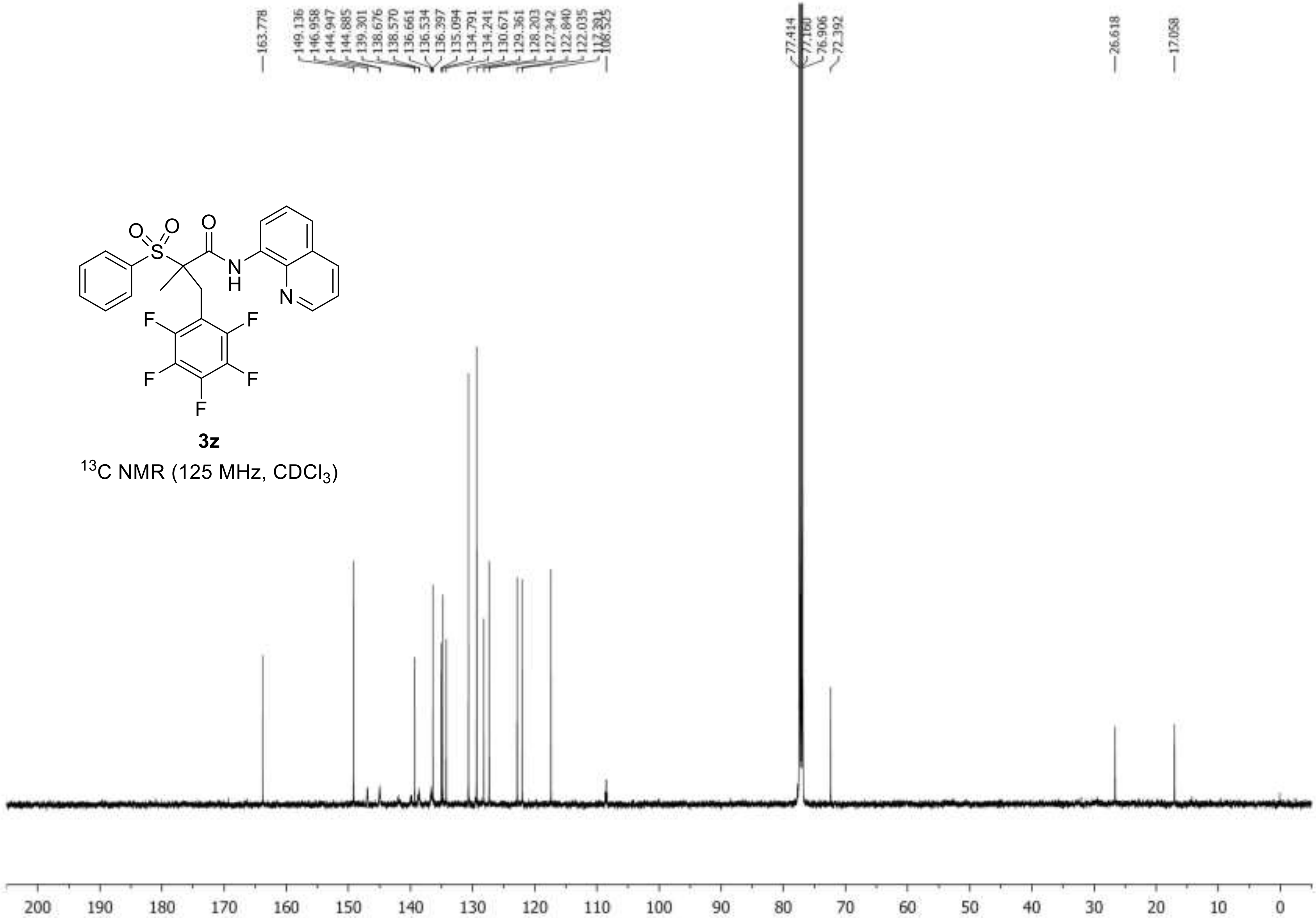

3z

^{13}C NMR (125 MHz, $CDCl_3$)

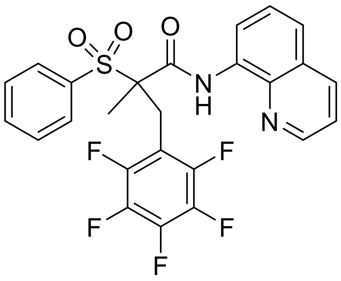

3z

^{19}F NMR (471 MHz, $CDCl_3$)

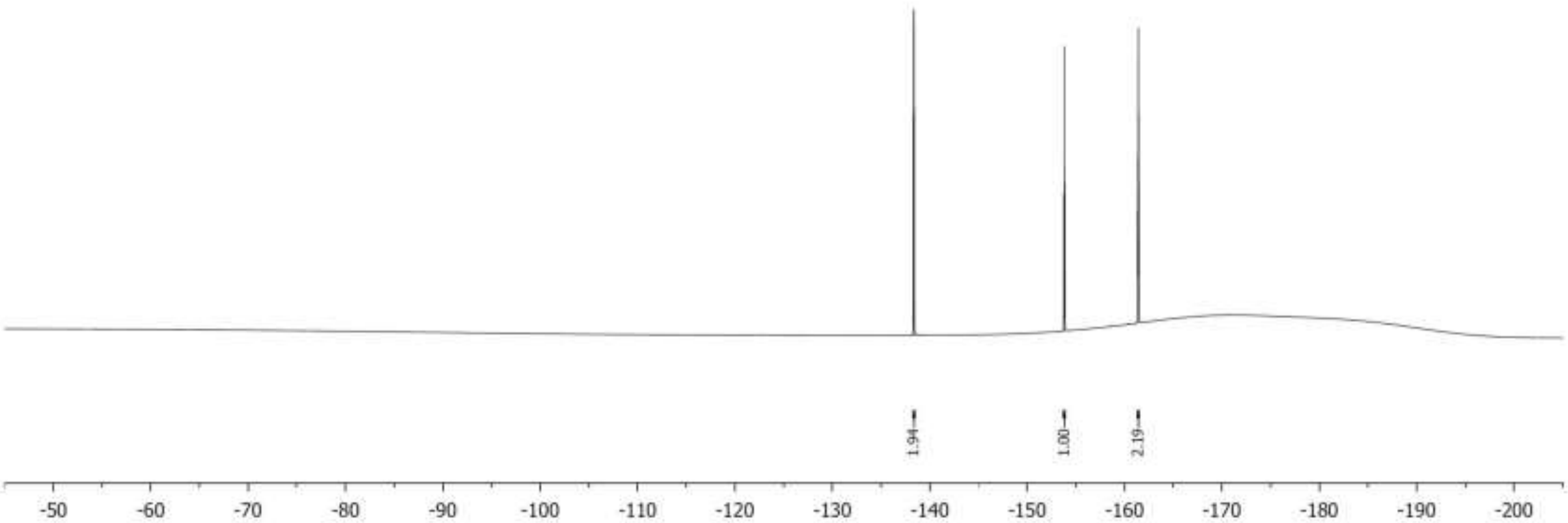

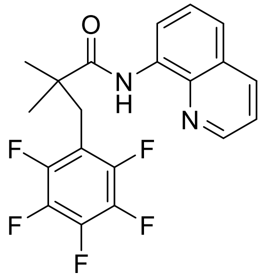

3aa1

^{1}H NMR (500 MHz, $CDCl_3$)

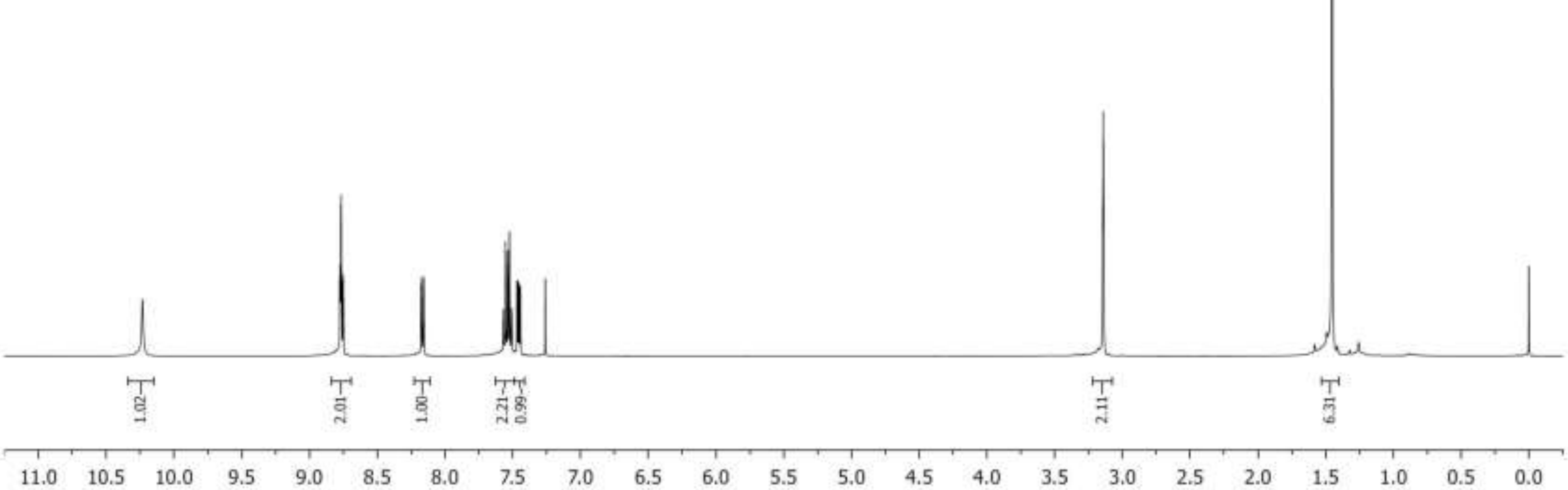

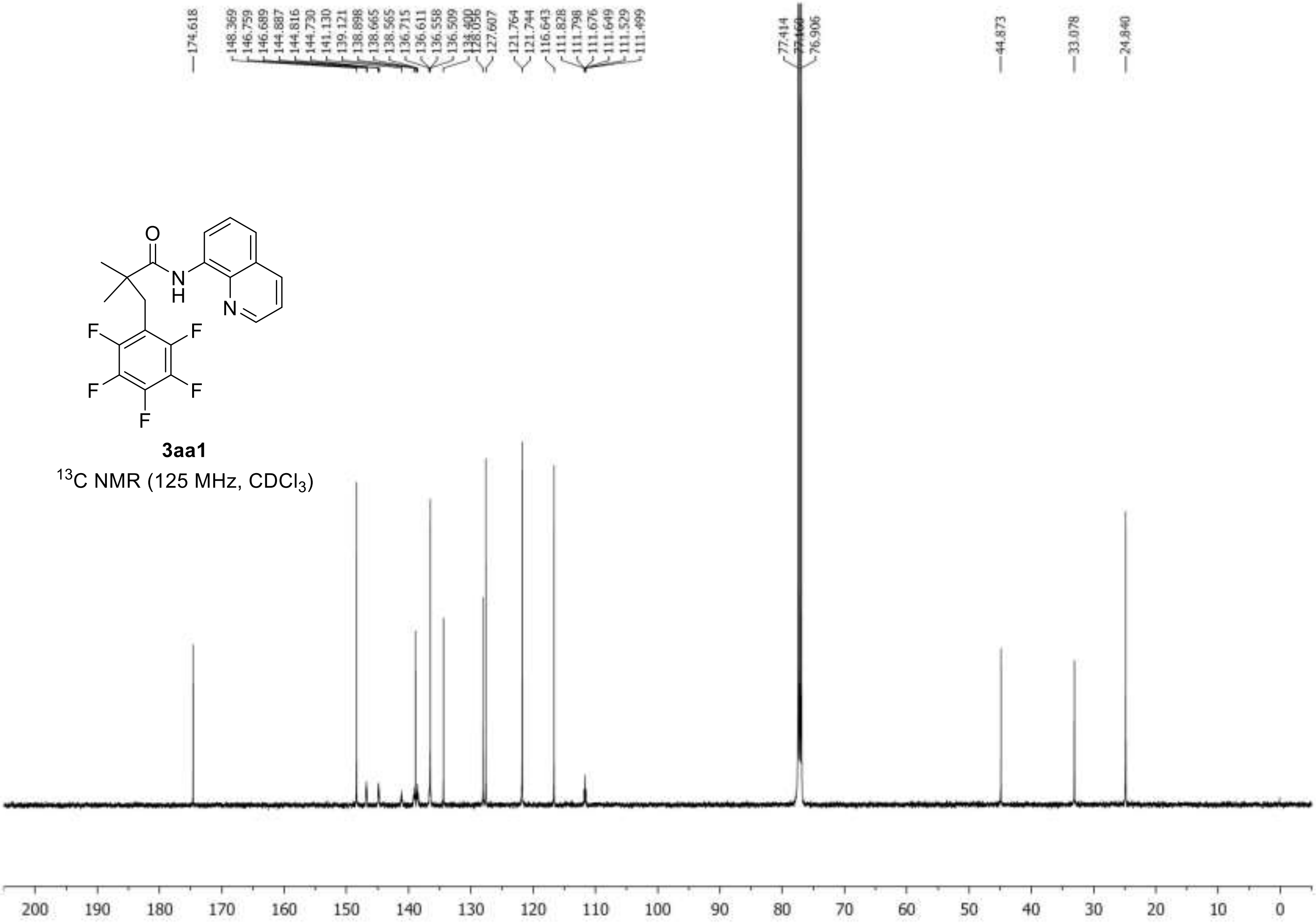

3aa1

^{13}C NMR (125 MHz, $CDCl_3$)

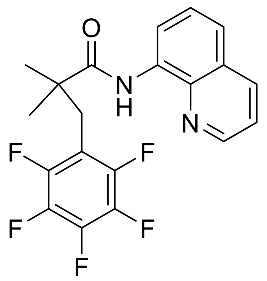

3aa1

^{19}F NMR (471 MHz, $CDCl_3$)

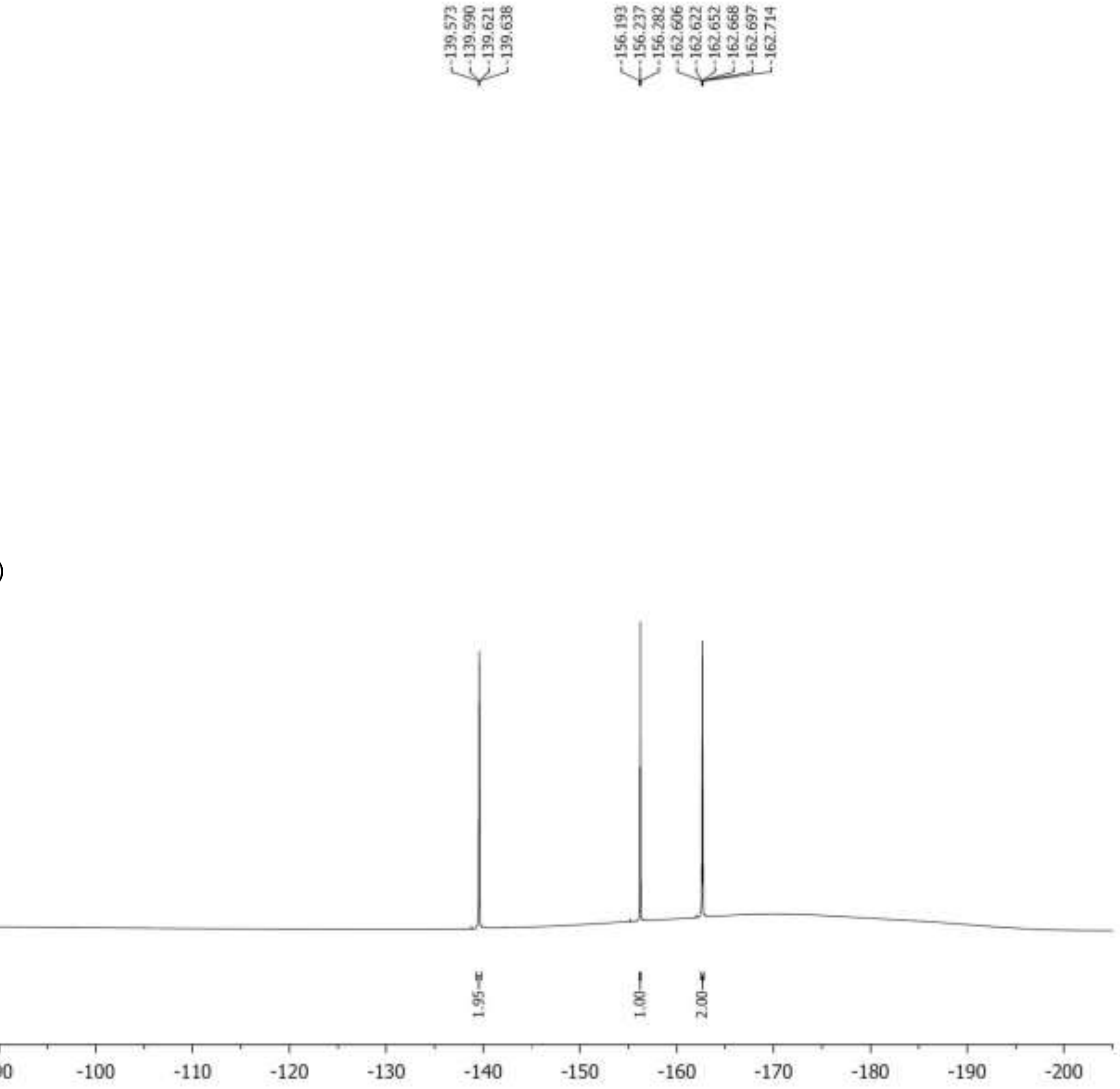

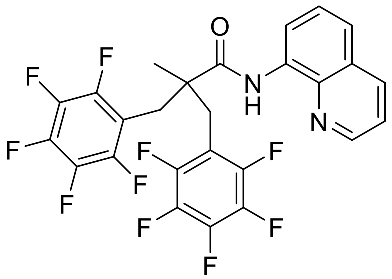

3aa2

^{1}H NMR (500 MHz, $CDCl_3$)

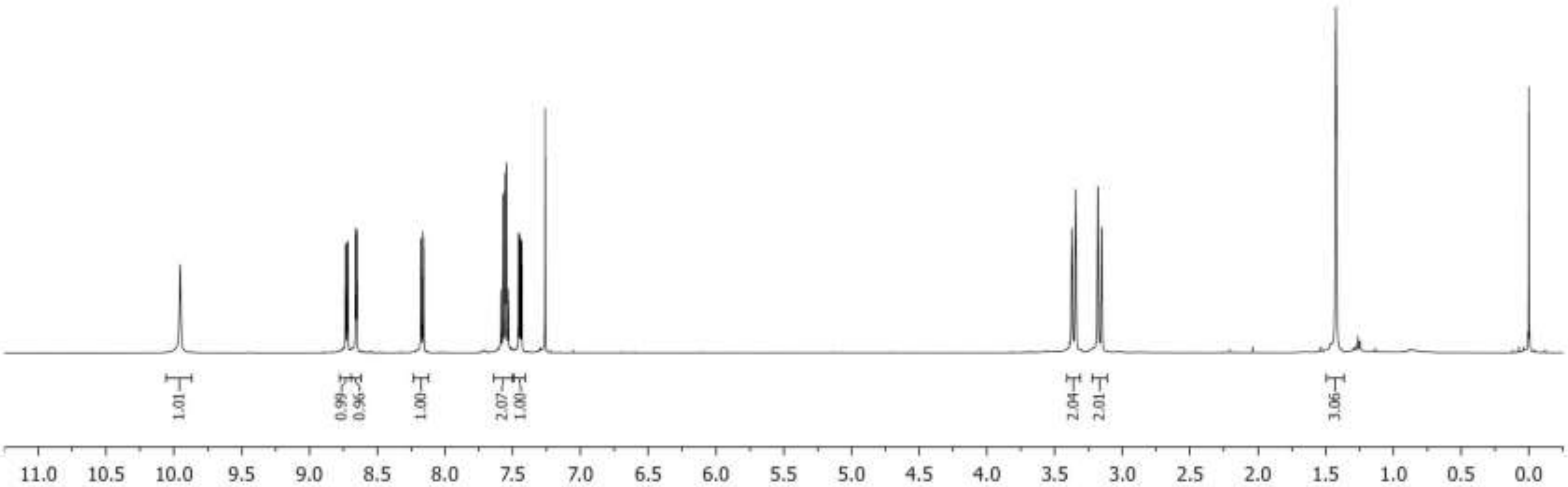

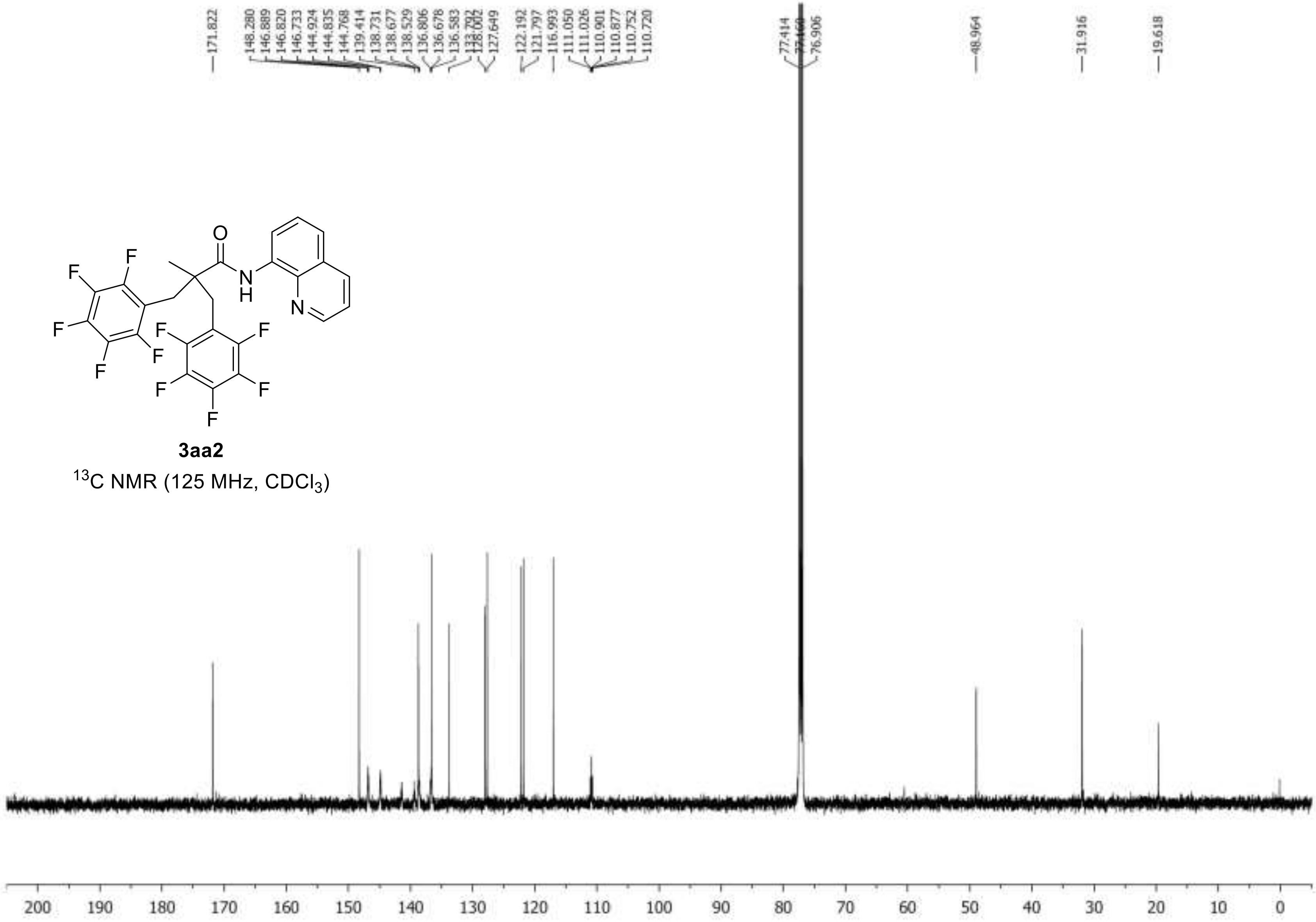

3aa2

^{13}C NMR (125 MHz, $CDCl_3$)

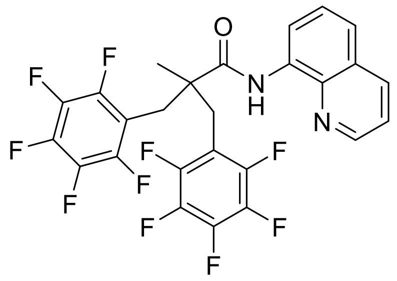

3aa2

^{19}F NMR (471 MHz, $CDCl_3$)

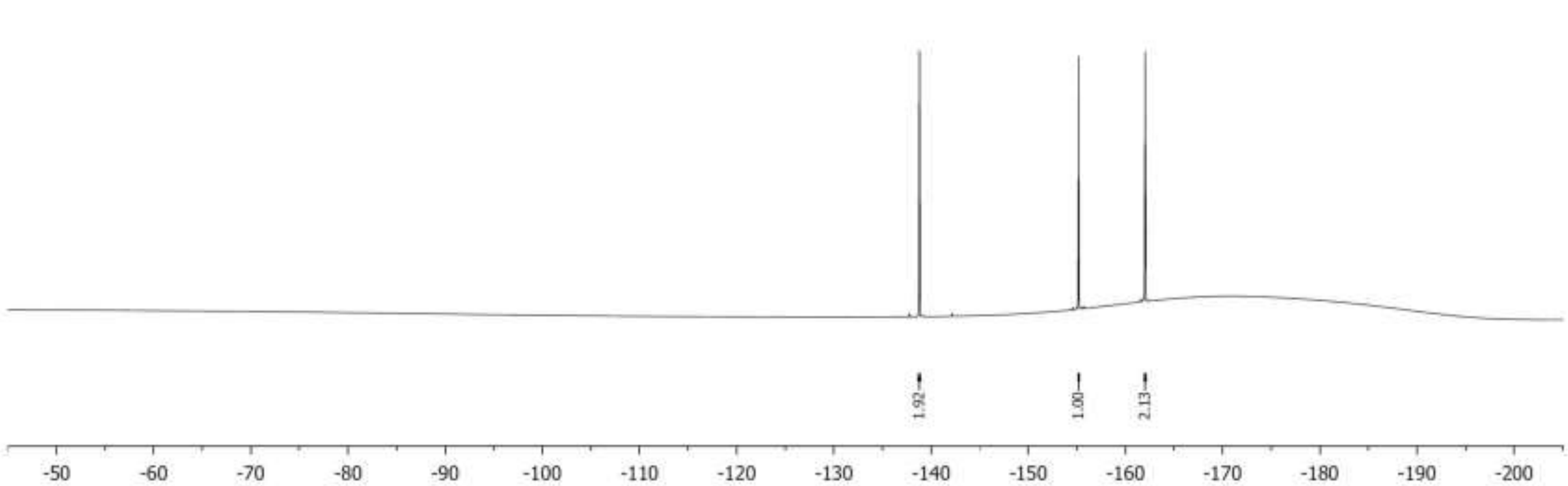

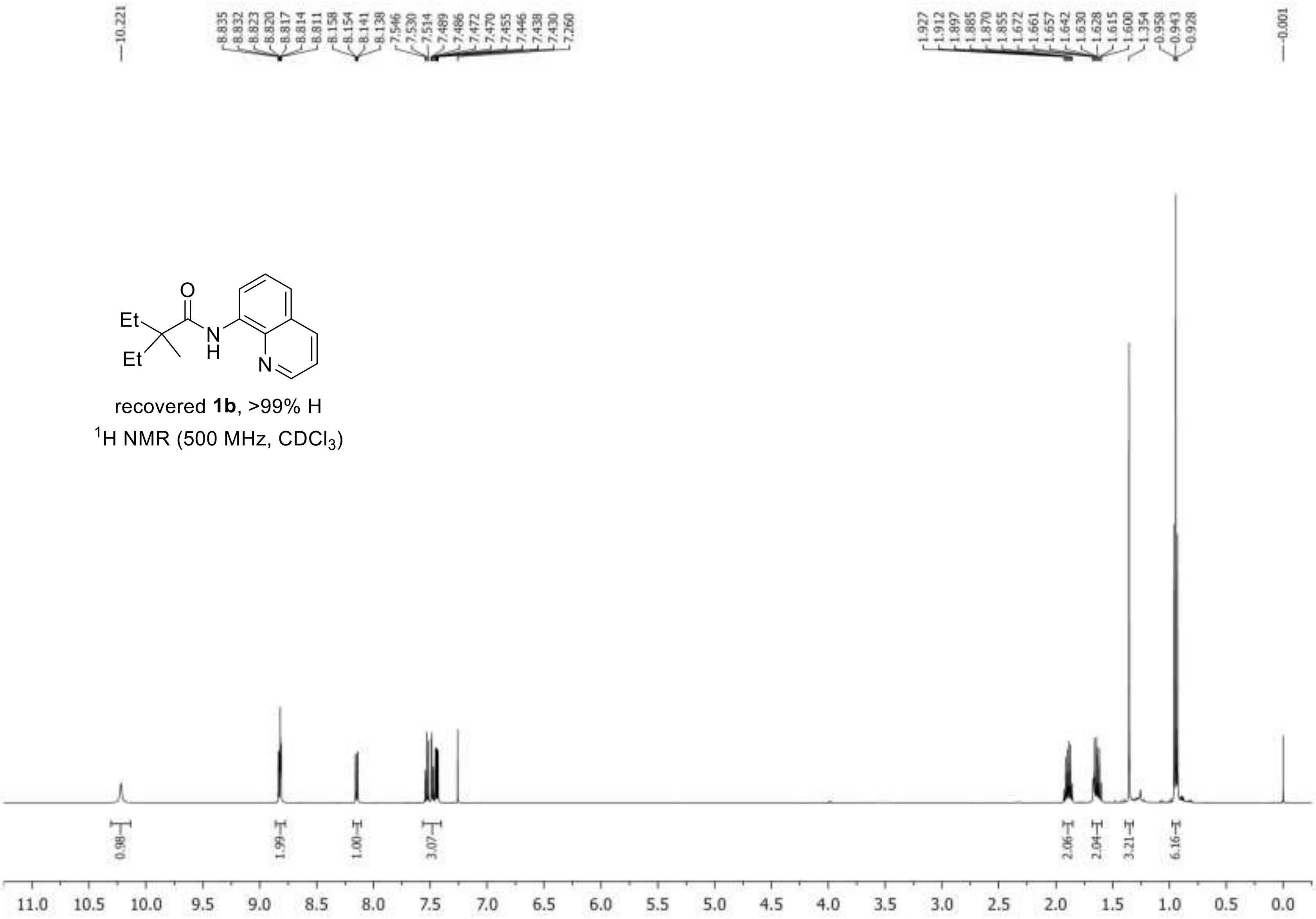

10.221
8.835
8.832
8.823
8.820
8.817
8.814
8.811
8.158
8.154
8.141
8.138
7.546
7.530
7.514
7.489
7.486
7.472
7.470
7.455
7.446
7.438
7.430
7.260
1.927
1.912
1.897
1.885
1.870
1.855
1.672
1.661
1.657
1.642
1.630
1.628
1.615
1.600
1.354
0.958
0.943
0.928
-0.001
O
Et
Et
N
H
N
recovered **1b**, >99% H
^{1}H NMR (500 MHz, $CDCl_3$)
0.98
1.99
1.00
3.07
2.06
2.04
3.21
6.16
11.0
10.5
10.0
9.5
9.0
8.5
8.0
7.5
7.0
6.5
6.0
5.5
5.0
4.5
4.0
3.5
3.0
2.5
2.0
1.5
1.0
0.5
0.0

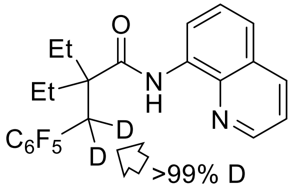

[D_2]-**3o**

^{1}H NMR (500 MHz, $CDCl_3$)

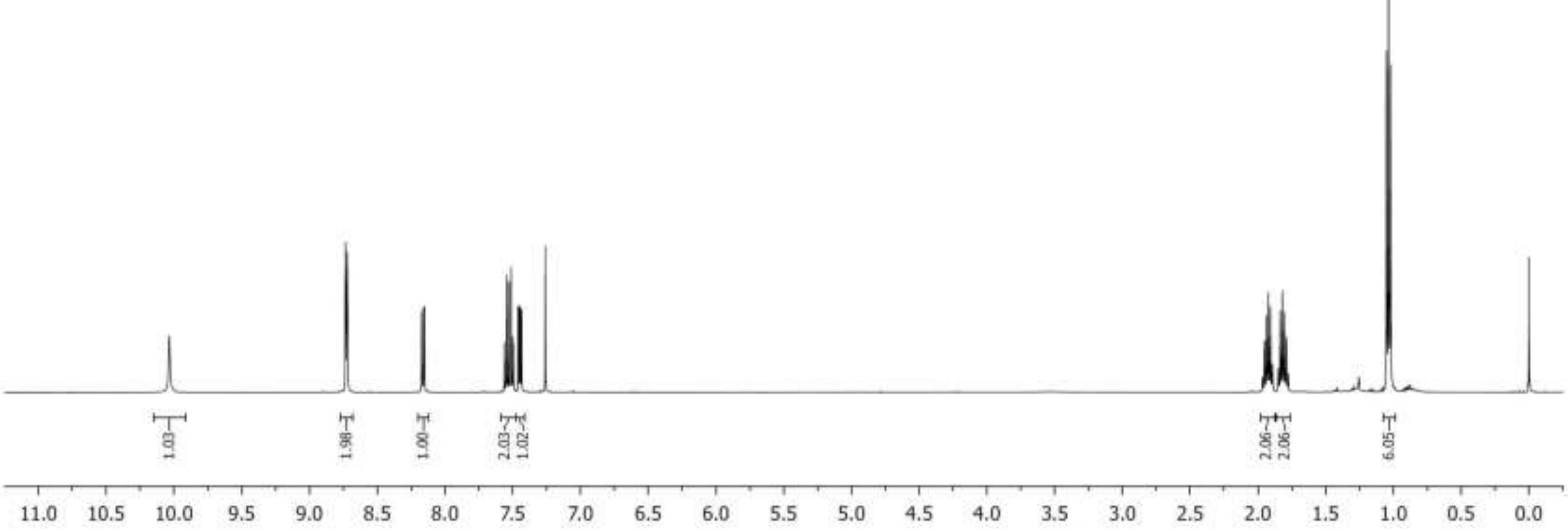

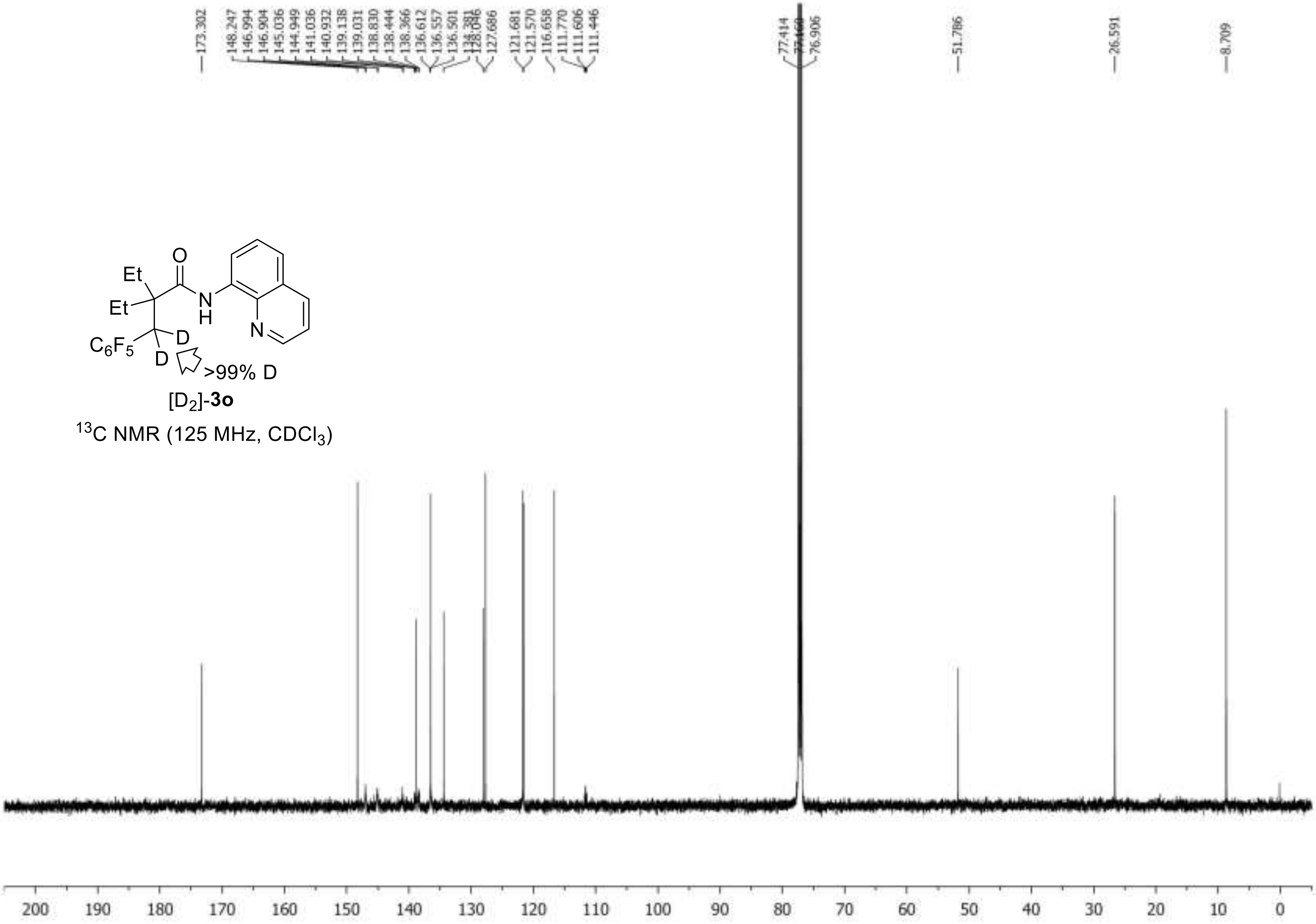

[D$_2$]-**3o**

^{13}C NMR (125 MHz, $CDCl_3$)

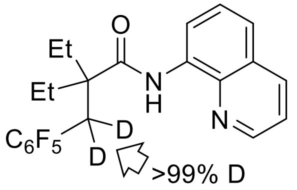

[D$_2$]-**3o**

^{19}F NMR (471 MHz, $CDCl_3$)

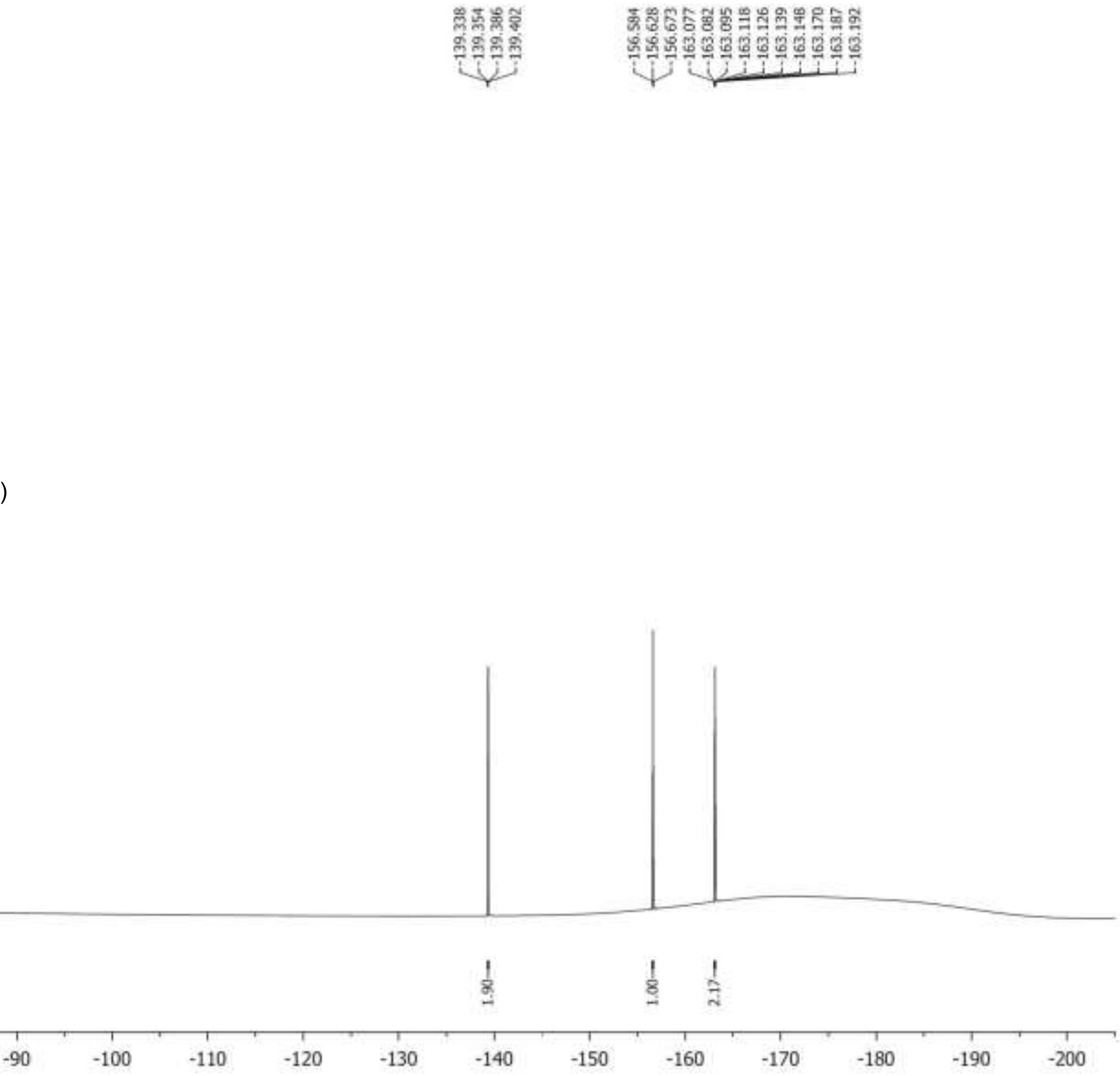

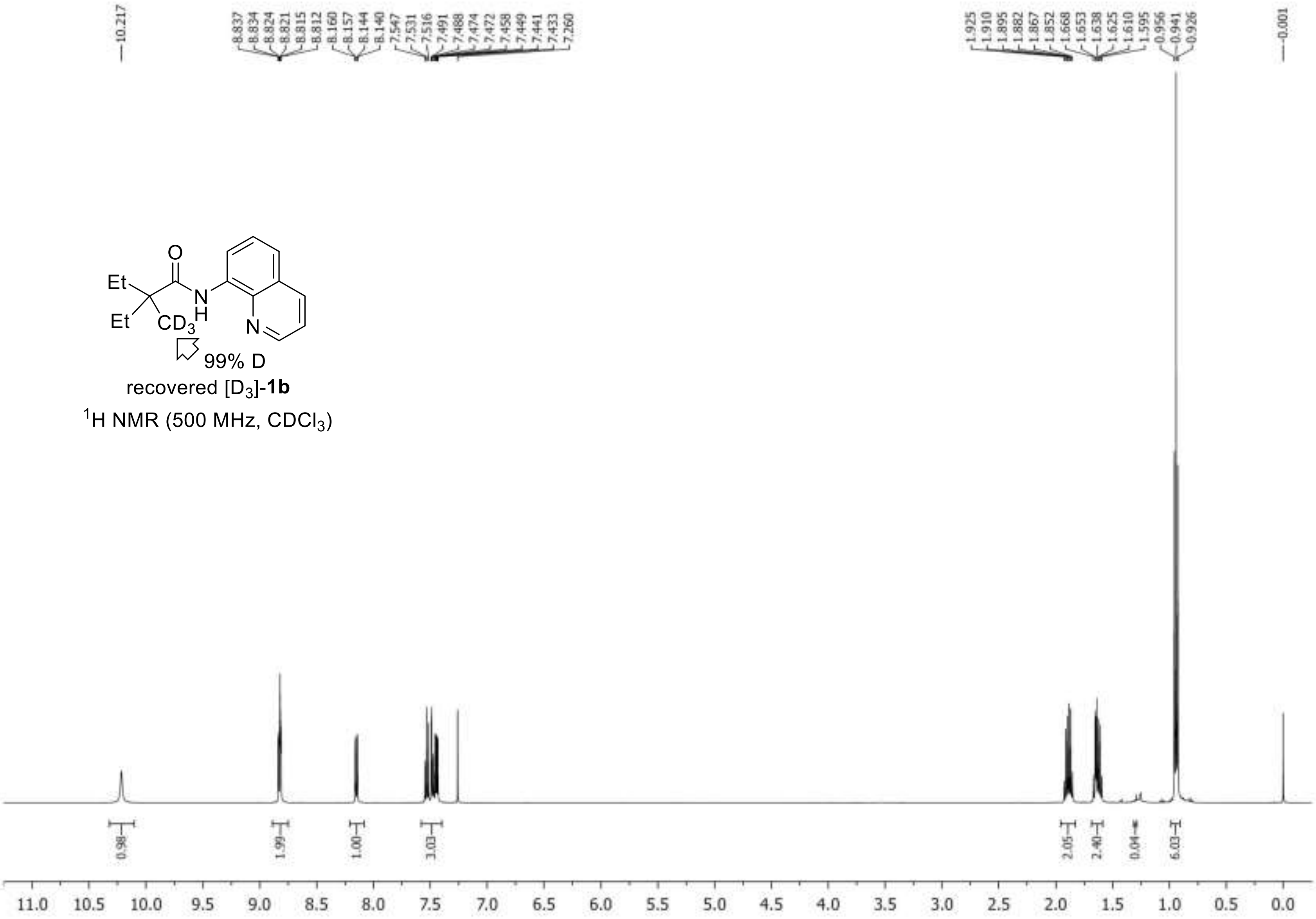
10.217
8.837 8.834 8.824 8.821 8.815 8.812 8.160 8.157 8.144 8.140 7.547 7.531 7.516 7.491 7.488 7.474 7.472 7.458 7.449 7.441 7.433 7.260
1.925 1.910 1.895 1.882 1.867 1.852 1.668 1.653 1.638 1.625 1.610 1.595 0.956 0.941 0.926
-0.001
Et
Et
O
N
H
CD3
N
99% D
recovered [D3]-1b
1H NMR (500 MHz, CDCl3)
0.98
1.99
1.00
3.03
2.05
2.40
0.04
6.03
11.0 10.5 10.0 9.5 9.0 8.5 8.0 7.5 7.0 6.5 6.0 5.5 5.0 4.5 4.0 3.5 3.0 2.5 2.0 1.5 1.0 0.5 0.0
